# A community approach to the Neotropical ticks-hosts interactions

**DOI:** 10.1038/s41598-020-66400-3

**Published:** 2020-06-09

**Authors:** Agustín Estrada-Peña, Santiago Nava, Evelina Tarragona, José de la Fuente, Alberto A. Guglielmone

**Affiliations:** 1 Department of Animal Pathology. Faculty of Veterinary Medicine, Zaragoza, Spain; 2 Instituto Agroalimentario de Aragón (IA2), Zaragoza, Spain; 3 INTA, Rafaela, Santa Fe, Argentina; 4 SaBio, Instituto de Investigación en Recursos Cinegéticos (IREC-CSIC-UCLM-JCCM), Ronda de Toledo s/n, 13005 Ciudad Real, Spain; 5 Department of Veterinary Pathobiology, Center for Veterinary Health Sciences, Oklahoma State University, Stillwater, OK 74078 USA

**Keywords:** Community ecology, Ecological epidemiology, Ecological networks, Evolutionary ecology

## Abstract

The relationships between ticks and hosts are relevant to capture the ecological background driving the evolution of these parasites. We used a set of 4,764 records of ticks of the genera *Amblyomma*, *Ixodes*, and *Haemaphysalis* and their hosts in the Neotropics to approach the tick-host relationships using a network-based construct. The network identified 9 clusters of interacting hosts and ticks partially connected by 22 tick species that switch their host range according to their life cycle stage. These links among clusters do not confer an extra resilience to the network following removal of hosts and subsequent cascade extinctions of ticks: the robustness of the network slightly changed when these inter-clusters links are considered. Phylogenetic clustering of ticks to hosts at cluster level was not significant (p > 0.15) but if examined individually 63 tick species/stages (59%) displayed such clustering, suggesting that their hosts have a related phylogenetic background. We interpreted these results under an ecological perspective in which ticks could track its environmental niche associating to vertebrates that would maximize tick survival under the range of abiotic traits. We encourage these integrated analyses to capture the patterns of circulation of tick-transmitted pathogens, a topic still unaddressed in the Neotropical region.

## Introduction

The relationships between hosts and parasites are commonly defined in terms of simple taxonomic units instead of communities of interacting organisms. Long-lasting associations among vertebrates and parasites should be realized as an interacting community^1^. Ticks and vertebrates constitute associations casted by specific interactions among the parasites and a range of vertebrates, under the directory forces of the environmental traits^1,2^. Studies exist summarizing the finely tuned relationships between tick species and the environment, the importance of key hosts in the tick life cycle, or the prominent role of some hosts in the circulation of some pathogens^3^. Pioneering efforts produced lists of ticks and vertebrate hosts of the African fauna^4^ or the realized environmental niche of ticks in the Palearctic region^5^. These studies resulted in partial pictures of the community of ticks and vertebrates. Further studies expanded that initial view by demonstrating that phylogenetically close species of African ticks are segregated along the niches of environmental variables, a finding interpreted as the best strategy to avoid the competition between closely related taxa^6^. Other groups of ectoparasites of wild vertebrates such as fleas and mites have been deeply studied at the community level^7–9^, demonstrating details about the phylogenetic and environmental drivers of diversity of these ectoparasites.

Ticks interact with their hosts in complex ways that can be understood only at a large spatial scale and with the adequate management of a large number of *bona fide* records, covering large biogeographical regions^10,11^. The interest on the ticks of the Neotropical region was historically restricted to species affecting livestock because the economic burden of the transmitted pathogens. However, a large body of knowledge on ticks of wild vertebrates accumulated in the last years^12,13^. Tools coming from network theory have demonstrated to be a valuable approach to characterize the community interactions and their variations in space and time^12^. The field of network theory is prolific in studies about the structure of food-web systems^14^ or the interactions between plant and pollinators^15^. The application of network theory to the ecology and epidemiology of ticks and vertebrate hosts is an emerging field and seems to be a promising way to reveal the largely ignored concept of community behind these interactions^16^.

This study aims to synthesize the ecological relationships between ticks and their wild hosts in the Neotropical region as a community of interacting organisms, aiming to move beyond the emphasis on particular interactions. We aimed to know if communities of Neotropical tick taxa are restricted by specific phylogenetic traits of vertebrates. The study focusses on the factors shaping the community of vertebrates and ticks in a large and diverse region and how they circulate in nature.

## Results

### Neotropical ticks form clustered networks with their hosts

The ticks of wild vertebrates in the Neotropical region form a network of multiple relationships, built over a set of 4,764 *bona fide* records of species of ticks with specific details of the host (ticks recorded on domestic animals were removed). After elimination of the tick species that were below a critical number of records, the network included 52 species of ticks of the family Ixodidae (34 species of *Amblyomma*, 2 of *Haemaphysalis* and 16 of *Ixodes*). The available information included a total of 143 combined tick species and stages (larva, nymph, adult). The network included a set of 384 genera of vertebrates, arranged in 106 families and 35 orders. The phylogenetic tree of the genera of hosts for which information is available, and on which calculations of phylogenetic diversity of ticks are based, is provided as Supplementary Fig. S1.

**Figure 1:**
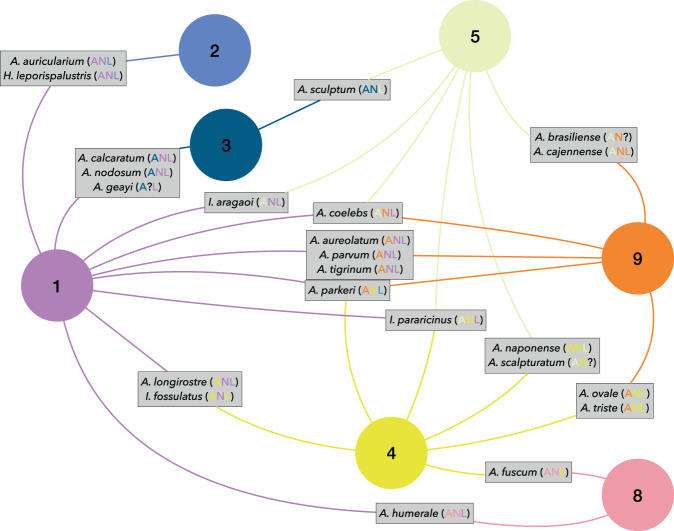
A schematic representation of the clusters of the network of Neotropical ticks and their hosts. In the scheme, circles are clusters of interacting organisms (species/stages of ticks and hosts). The numbers are consecutive and are used as the name of the cluster. The colors are random and match the colors in the complete network of Supplementary Fig. S2. The small squares include species of ticks that switch the cluster (and thus hosts) according to the stage of the life cycle (A: adult, N: nymph; L: larva). The colors of each capital letter naming the stages are those of the clusters in which they are integrated. Lines link the clusters with the same colors. Clusters 6 and 7 are not included because they only have one species of tick. The complete network is available complete as Supplementary Fig. S2.

The Louvaine algorithm summarized the interactions among ticks and vertebrates into 9 clusters built around the taxonomic clusters of hosts (Fig. 1 and Supplementary Fig. S2). We will refer as “cluster” to each group of ticks and hosts that interact more strongly among them than with members of other communities. The network was loosely nested: the index of nestedness is 16.2, in the range 0–100 (minimum: 9; maximum: 32, for the different clusters). The network does not consist of isolated clusters but is reinforced by many interconnections among clusters by both ticks and hosts (i.e. ticks exploiting hosts of different clusters or vertebrates parasitized by tick species of different clusters). However not every tick species is linked to the same cluster of hosts for every life cycle stage: some ticks switch the range of hosts according to its developmental stage. Therefore, the tick species showing this trait completely change their host range among stages, and in some cases switch their strategy of endophilic-exophilic behavior (living inside the host shelter or questing in the vegetation). Figure 1 summarizes the tick-derived relationships among the clusters and the species of ticks that switch the group of hosts. The complete network with every species/stage of ticks and genera of hosts is displayed in Supplementary Fig. S2. To note that this switching of clusters according to the tick stage is mainly observed in the genus *Amblyomma*: 19 species (55% of the *Amblyomma* fauna included in the study) switch the membership to different clusters of hosts according to the life cycle stage. The most commonly recorded strategy is the use of a cluster of hosts by immature ticks (larva and nymph) while adults switch to a different cluster of hosts. However, two species of *Amblyomma* (*A. coelebs*, *A. parkeri*) use a different cluster of hosts for each life cycle stage. Only 3 species of *Ixodes* (18%) switch the cluster of hosts according to the life cycle stage.

Supplementary Fig. S2 complements Fig. 1 and displays the complete set of interactions among ticks and vertebrates. The numbering of the clusters of ticks and hosts is consecutive and random. Community 1 includes 181 genera of birds (35 Families, 11 Orders) and their associated ticks. The only tick with adults represented in this group of hosts is *Ixodes auritulus*. The remaining 18 species have only immatures associated with these vertebrates. There are only 3 species of ticks associated to hosts in the cluster 2, recorded on Cingulata, Lagomorpha, and in a smaller degree on Tetraodontiformes and Tinamiformes. Cluster 3 represents a small group of 8 tick species mainly associated to Pilosa. Cluster 4 is a large assemblage of immature and adult ticks that are associated to Rodentia and Didelphiomorphia (72 genera in 17 Families). Cluster 5 includes ticks associated mainly to 17 genera of Artiodactyla. Clusters 6 and 7 are separated from the main component of the network (see Supplementary Fig. S2), because they are represented by ticks restricted to a small range of hosts, not shared by other ticks in the network. All the stages of *I. paranaensis* (cluster 6) are restricted to two genera of Apodidae (Aves) and *I. neuquenensis* (cluster 7) is restricted to the genus *Dromiciops*, a marsupial. No other ticks have been reported on these hosts. The ticks belonging to cluster 8 are associated to Anura, Squamata and Testudines (i.e. amphibians and reptiles). Ticks in the cluster 9 are mainly associated to Carnivora.

**Figure 2:**
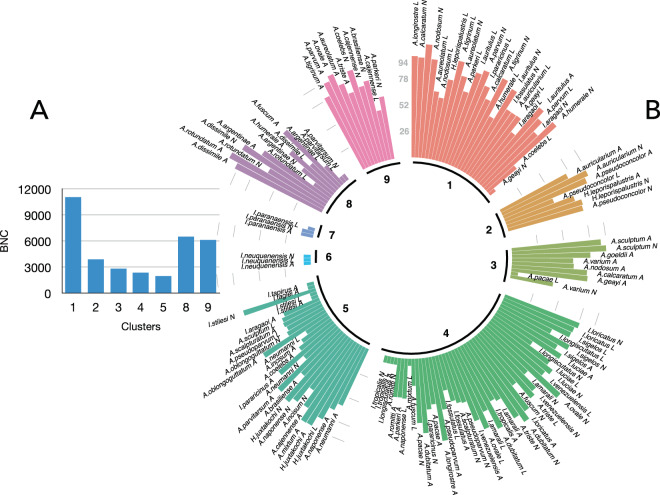
The values of centrality (BNC) of the ticks in the network. (**A**) Averaged values of centrality of the ticks belonging to the clusters detected in the network. Clusters 6 and 7 are not included. (**B**) The values of BNC for each species of tick separated by cluster (inner circle). The value displayed was scaled to [log(BNC + 1) × 10] to avoid zero values and allow enough amplitude as to be mapped in the histograms. Each histogram is labelled with the species and the stage of the tick (A: adult, N: nymph, L: larva). Abbreviations for the genera of ticks are A (*Amblyomma*), H (*Haemaphysalis*) and I (*Ixodes*).

Centrality is used as a measure to capture the importance of each organism in the context of the network. The values of betweenness centrality (BNC) for every network-derived cluster, as well as for individual tick species/stages are included in Fig. 2A,B. This index indicates how a tick links different genera of hosts, giving a measure of the relative importance of the tick in the context of the complete network. Clusters have largely variable values of tick’s BNC (Fig. 2A). Highest values were observed for ticks populating the clusters 1 (Aves), 8 (amphibians and reptiles) and 9 (Carnivora). These results derived from the number of hosts genera on which ticks have been recorded: a tick linked to more vertebrates will have a greater BNC. Clusters 1, 8, and 9 included the highest number of hosts recorded in the complete network, and ticks in these clusters have been reported on several genera of hosts of the same cluster, contributing to these higher values of BNC. Figure 2B displays the BNC values for each tick species/stage with an explicit mention to the membership cluster, demonstrating the importance of each parasite in the context of the complete network. Both *I. paranaensis* and *I. neuquenensis* have the smallest values of BNC because they are not linked to the main component of the network and are restricted to their own communities. Both exophilic and endophilic tick species have similar values of BNC (4807 and 4940, respectively).

### Neotropical ticks show variable clustering to the phylogenetic traits of hosts

**Figure 3:**
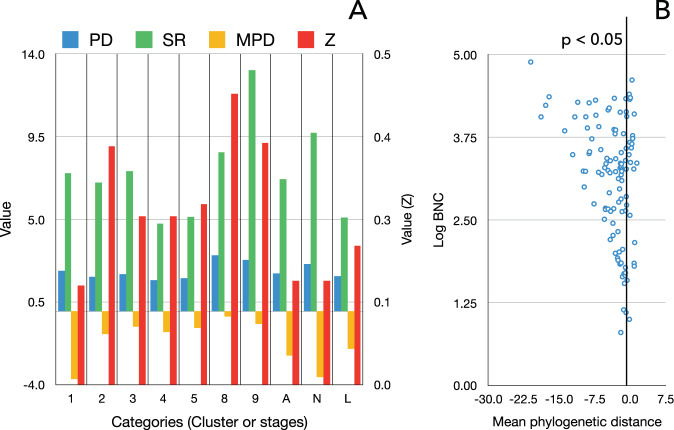
Values of phylogenetic clustering of the Neotropical ticks. (**A**) The values of Faith’s phylogenetic diversity (PD), host genera richness (SR), mean pairwise distance on the phylogenetic tree (MPD), and significance of MPD (Z) for all the species of ticks belonging to each cluster in the network (but 6 and 7). Also included are the indexes grouped according to the stages of the tick’s life cycle (A: adult, N: nymph, L: larva). Values of Z read in the right axis of the chart. (**B**) The plot of log(BNC) against MPD for each species of tick separately. Values at left of the black vertical line have a significant MPD (p < 0.05).

A quantitative analysis of the specialization of the Neotropical ticks along the phylogeny of hosts was made using measures of community phylogenetic structure. The mean pairwise distance (MPD) calculates the average phylogenetic distance among the genera of hosts used by each species of tick, either individually or along the clusters detected in the network. The value of MPD is compared with the observed phylogenetic relatedness produced by null models of community randomization, therefore providing a measure corrected by the size of sample (the number of available records of each tick). This index provides an estimation about how specialized or generalist a tick is regarding the phylogenetic distance of the complete range of the recorded genera of hosts. A complementary index is the Faith’s phylogenetic distance (PD). The phylogenetic specialization of ticks on hosts genera is included in Fig. 3A. Ticks belonging to clusters 6 and 7 could not be included because they lack a minimum variety of hosts to derive MPD. Phylogenetic clustering of clusters (averaged for the species and stages of ticks in each community) is low, with non-significant values. Both the tick species in the community 1 (birds) and the nymphal stages considered together for every species display the highest, but not significant, phylogenetic specialization. Considered as an entity, clusters do not specialize around phylogenetic communities of hosts.

These results changed drastically after an individual analysis of each tick species, since 63 species/stages of ticks (out of 109 species/stages, 59%) showed phylogenetic clustering to hosts with p < 0.05 (Supplementary Table S1 and Fig. S3). Differences in the degree of phylogenetic clustering between exophilic and endophilic ticks, stages or communities were not observed (p = 0.67). These results suggest that even if these species are parasites of a large number of vertebrates, most of their hosts have a closely related phylogenetic background. These results showed that the ticks in each cluster are essentially host-generalist, but each cluster may consist of species with low or high MPD, independently of the stage and the cluster considered.

An inverse relationship between the MPD and BNC was noted along tick species (Fig. 3B). As the phylogenetic clustering increase (in the chart, the lower values of MPD) BNC increases. These are ticks that link genera of hosts that otherwise would be not connected, resulting in a high BNC (which is a relative index that considers the complete network). This result reinforces the notion that ticks with high BNC may be either generalists that use a wide range of hosts or restricted to a narrow range of vertebrates with a common phylogenetic background. This relationship occurred independently of the group of hosts or the tick stage.

### Extra-cluster connections slightly affect the resilience of the network

**Figure 4:**
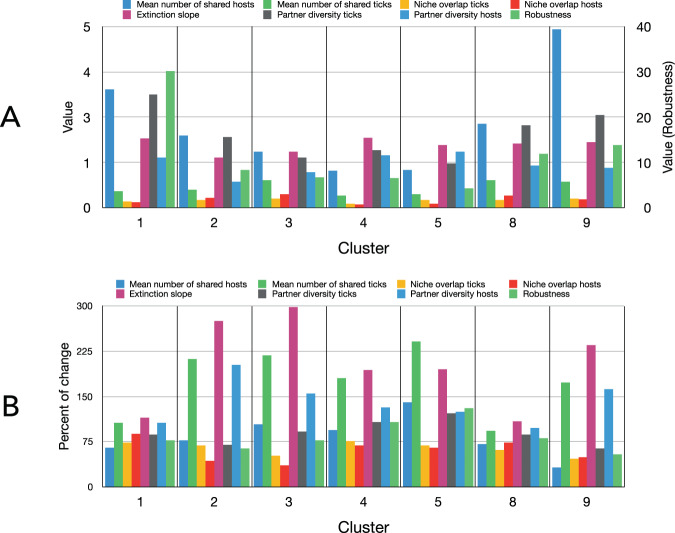
Tick-host partnership indexes, biotic niche overlap, and robustness of the clusters of the network. (**A**) Values for some indexes obtained for the combinations of tick’s species/stage and hosts in each cluster including the extinction slope and robustness (i.e. two indexes of elasticity of the network of ticks to the cascading extinction events after removal of hosts). Values of robustness read in the right axis of the chart. Higher values of the extinction slope mean for higher resistance to extinction. Only the links among ticks and hosts within each cluster are considered in the chart. (**B**) The percent of change of the indexes above after inclusion in calculations of every link among communities (i.e. ticks switching hosts in consecutive life stages or hosts parasitized by ticks of different communities). Values below 100 mean a decrease of the index; values above 100 mean an increase of the index.

Complex systems may display a high degree of tolerance against errors but result vulnerable to attacks (i.e. to the removal of a few nodes that play a vital role in maintaining the network’s connectivity). We subjected the network to a removal of hosts and examined its robustness aiming to compare differences between attacks to the isolated clusters or including the many links connecting clusters. The removal of critical nodes is expected to progressively disintegrate the network in a cascading scenario. Some indexes obtained from the iterative removal of hosts produced an overview of the response of each cluster to attacks (see Material and Methods for the complete description of the indexes). Nonetheless, clusters of interacting ticks and hosts are not closed compartments (but species included in clusters 5 and 6). Therefore, we run these analyses twice, first on each cluster separately, then on each cluster including the links recorded with the ticks or hosts belonging to other communities (Fig. 4). The aim was to demonstrate if the resilience of the clusters is affected by these extra links.

Each cluster has a variable number of shared hosts per tick or shared ticks per host, giving also variable values of niche overlap (in terms of biotic niche, or host’s overlap) and partner diversity. Both robustness to removal of nodes and extinction slope, two indexes related to network resilience, were highest in community 1 (Aves) probably because the large number of shared hosts by the ticks in this cluster: 3.5 hosts/tick (Fig. 4A). To note that the meaning of the extinction slope is inverse to the vulnerability of the cluster. The inclusion in calculations of the connections linking each cluster with others slightly changed the extinction slope but decreased the robustness (Fig. 4B). The procedure resulted in an increase of the extinction rate (a decrease of the extinction slope) and dramatically decreased robustness in most clusters. In conclusion, the life cycle traits of individual tick species/stages and the resulting switching of clusters according to the use of different ranges of hosts do not affect the robustness of the network.

## Discussion

We proposed a synthesis of the ecology of ticks and wild vertebrate hosts in the Neotropics that considers the community of interacting organisms. More than half species/stages of Neotropical ticks seem to have strict phylogenetic relationships with hosts, while the rest are generalists. The complete framework provides significant information of the relative importance of the biotic interactions between tick species and different groups of vertebrates.

Our findings suggest that several tick species in the target region phylogenetically cluster around some hosts, demonstrating a strict specificity for host taxa. In several instances, the same tick species belongs to two different clusters, where one of the parasitic stages (i.e. adults) is clustered around some hosts and the others (i.e. immature) to another group of hosts. This should not be interpreted as an ancient linkage of ticks along hosts lines, since a significant evolutionary event produced a deep and relatively recent modification in the mammal faunal composition in the Neotropics: the Great American Biotic Interchange (GABI). During this major event, occurred about 2.5 My., many mammal species had dispersed between North and South America^17,18^. Prior to this event, the South American mammalian fauna, (i.e. all the large and small carnivores, which were marsupials, or the large grazing herbivorous) was completely different to the current mammalian composition^18^. Many clades of large and medium-sized mammals became extinct and replaced by other taxa spreading from the Northern Hemisphere^17,18^. Because some extant tick species from South America (e.g. *Amblyomma* species) were already differentiated before GABI^19,20^ it can be assumed that they formerly used a range of vertebrates that are now extinct, then adapting to a completely different host spectrum without a close evolutionary relationship with the early fauna. This is an example of a rapid adaptation to invading clades of vertebrates, the community of Neotropical ticks being thus extremely flexible adapting to these new hosts, a conclusion that precludes a strict phylogenetic adaptation of the ticks to ancestral hosts clades. Two species, *I. paranaensis* and *I. neuquenensis*, represent an unusual pattern, because they are linked to a small group of hosts, and therefore they probably exhibit a strict monoxenous behavior towards these vertebrates.

The results obtained are strongly suggestive of the use of hosts that outline the set of environmental traits that ticks could tolerate. The phylogenetic clustering of ticks with vertebrates would not derive from a strict biotic dependence but would result from the adaptation to environmental conditions. The detected clusters are thus formed by tick species occupying a similar ecological niche. For example, *A. pseudoconcolor*, *A. auricularium* and *H. leporispalustris* belong to different genera and use different hosts (Xenarthra and Lagomorpha) but all of them are nidicolous ticks associated to mammals with nidicolous habits. The same applies to exophilic and ambushing tick genera, all of them parasites of different families of large herbivorous. Other than two large clusters displaying the community of parasites of birds, reptiles or amphibians, the construct resolved the ticks/stages into two clearly defined branches: endophilic and exophilic ticks/stages. The strategy of host selection driven by environmental constraints would be enhanced by the switch of groups of hosts, immatures being parasites of a lineage of hosts, adults using a different group of vertebrates^21^ or the swap of endophilic and exophilic behavior according to the life stage. The switch of hosts and/or questing strategy according to the stage of tick’s life cycle could maximize the survival of the ticks under variable environmental conditions (i.e. birds or rodents, living in nests or burrows increasing tick survival) together with the dissemination of adult stages through the use of large animals. To note, the reported spatial range of the ticks is always smaller than the realized host’s range. This may derive from a lack of surveillance in some regions, or because other, not yet captured traits, would result in the confinement of the tick’s spatial range as a subset of the hosts distribution. The topic deserves further research.

This study also confirmed the proposed relationships of the sigmodontine rodents (part of Cricetidae) with South American Ixodidae^22^. It has been proposed that the *Ixodes*-Sigmodontinae relationship evolved from a South American tick ancestor parasite of Didelphidae. Such cluster of *Ixodes* and rodents is of interest, since Cricetidae has an origin independent of rodent ancestors introduced from Africa^23^. It is postulated that sigmodotine rodents entered Southern America not later than 5 mya^24^ with a very diverse radiation. Our study demonstrated that four species, *Ixodes amarali*, *I. loricatus*, *I. luciae*, and *I. sigelos*, have been recorded as belonging to the same cluster, paralleling previous conclusions based on DNA systematics and morphological features^22^. Molecular studies suggested that both *I. taglei* and *I. stilesi* should belong to a second clade and the network placed them in a different cluster. *Ixodes neuquenensis* (established in South-Western South America) is considered a relatively new species that radiated from *Ixodes* parasites of Sigmodontinae invaders in southern South America, and uses its exclusive host *Dromiciops gliroides*, placed in a separated cluster. The network could not separate species of tick parasites of either Rodentia or Didelphiomorphia in different clusters. This seems to be derived from the fact that several species/stages of ticks in this cluster have been recorded on both orders of vertebrates.

It has been reported that compartmentalization in food-webs increases its persistence^25^. The concept was already proposed by Robert May in 1972, who stated that the stability of a complex system would increase if species are arranged in compartments^26^. Our results support the theoretical framework: the network of Neotropical ticks and wild hosts is highly compartmentalized. Resilience to disturbance was found in this study to be slightly affected by the links among organisms. This supports that the relationships of ticks and hosts seem to be originated as features optimizing the survival and spread of each species, and most probably to avoid host overlap. This may be a fertile field of research, since it has been demonstrated that close species of Tropical ticks do not share the same environmental traits, most probably to avoid the overlap on hosts^27^.

The networks construct has a potential for examining the spread of tick-borne pathogens through the interacting vertebrates and ticks. Some pathogens of importance in human health, like *Rickettsia* spp., have been widely recorded^28–31^. A probable lack of strict molecular dependence allows these bacteria to unrestrictedly move among groups of vertebrates using different tick species, therefore being recorded from vertebrates that are phylogenetically widely separated. Graph theory could provide the framework to explore the relationships between ticks and hosts that promote and support the circulation of pathogens. To note that some vertebrates may act as super-spreaders of ticks, since they act as a “catch-all” for ticks of different clusters allowing them to circulate in the gradient of environmental traits. Most of these super-spreaders are vertebrates that colonize large regions^31^. This is relevant because these vertebrates may reach high local density in sub-urban habitats with an impact on human health.

This study explored the trophic relationships among ticks and hosts in Neotropics, using the largest available dataset of records in the region. The framework managed to display the modularity of the interplaying organisms, matching previous results based on molecular data and showing unexpected behaviors in a highly compartmentalized construct. The network clearly displayed five large groups of vertebrates interacting with ticks, which we interpreted as the background of the ecological strategies of ticks and not a true physiological dependence. We suggest the need to move beyond the particular interactions between ticks and parasites, which may be of local nature, adopting the community perspective.

## Methods

### Raw data

This study was based on a literature survey covering the records of ticks in the Neotropical region on wild hosts, from Mexico to the southern cone. The survey was carried out and assessed for reliability and accuracy by two of the co-authors (see author’s contributions). The complete raw data includes 77 species of ticks of the genera *Amblyomma*, *Haemaphysalis* and *Ixodes*, reported from 398 genera of hosts, with a total of 4,764 records with indication of the stage of the tick (larva, nymph, or adult). The three stages were sometimes reported in the same locality/host and in the same bibliographical reference, thus providing a total of 7,869 pairs of relationships tick/stage and host. Other genera of ticks in the region are mainly parasites of livestock (i.e. *Rhipicephalus microplus*) or pets (i.e. *Rhipicephalus sanguineus* s.l.) and were not included in the main database, since it has been demonstrated that their consideration deeply alters the balance of the relationships between ticks and hosts. The complete set of data is available in DataDryad at 10.5061/dryad.860473k.

### Building networks of interacting ticks and hosts

We used an approach previously reported in which records of ticks on vertebrates are converted into a graph structure^12^. A graph (or network) is composed by nodes (ticks or vertebrates). Edges among nodes (links) reflect the relationships among parasites and hosts. The strength of the association (i.e. the weighted number of times a tick has been reported on a given host) is the weight of the link. The use of networks allows the calculation of ecologically relevant indexes related with the relationships among ticks and vertebrates, namely modularity, centrality and nestedness. Most calculations were done with the software Gephi v0.92 (www.gephi.org, last accessed November 2018) but some results were obtained in the R programming environment^32^.

Strictly speaking, modularity is a measure of the organization of taxa in subsets of species that interact more frequently among them than with the other taxa of the network, forming clusters of interactions, considered as groups of tightly interacting organisms. The number of clusters, the organization of species and the links among nodes in different clusters are important clues to understand the relationships of the Neotropical ticks and their hosts. We calculated the modularity and thence the clusters using the Louvaine algorithm available in Gephi. A system of interacting organisms is said to be nested when the clusters that have a few items in them (species with few interactions) are a subset of the items of clusters with more items. We used the measure of nestedness as available in the package “bipartite”^33^ for R.

### Evaluating the behavior of the network

It became evident from the main dataset of records, that several species of ticks switch the strategy of questing for hosts according to the stage of the life cycle: some species may be endophilic (living inside the host shelter) in their immature stages, or exophilic (questing for host in the vegetation) at the adult stage. These ticks switch the group of hosts (and therefore the cluster of the network to which they belong) according to the life cycle stages. Other than the ecological advantages that such strategy might have for each species (related to environmental traits or to the relevance of the food source) we evaluated how it impacted the persistence of the network, like a higher robustness to the removal of hosts. It commonly happens that the removal of one or several nodes has catastrophic consequences for the rest of the network, resulting in the disconnection of some nodes from the main component (i.e. the complete set of interacting clusters).

We evaluated the features of the complete network and the different clusters using several indexes. Indexes were calculated for (i) the individual clusters alone, excluding the edges linking different them and (ii) for the members of each cluster and the further links to other clusters (by either ticks to hosts or hosts to ticks) that relate some nodes among clusters. In the first step, we consider each cluster as an isolated group of interacting organisms; in the second, the switch of hosts is included. In our application, the interest is focused to capture the meaning of these relationships among clusters for the complete network, and not for individual species.

The following indexes were calculated in “bipartite” for R: *mean number of shared hosts* and *mean number of shared ticks* (an expression of how many genera of hosts are shared by the same species/stage of tick, or how many species/stages of ticks are shared by the same genus of vertebrate), the *niche overlap* of both ticks and hosts (mean similarity in interaction pattern between species of the same level, either ticks or hosts), the *extinction slope* (the exponent of an hyperbolic model fitting the effects of the removal of hosts on the number of secondary extinctions of ticks; a higher value means a lower extinction rate), the *partner diversity* for ticks or hosts (i.e. the weighted mean Shannon diversity of the number of interactions for the species of that level), and the robustness (this is identical to exp[“partner diversity”], i.e. the version of diversity proposed in^34^). As mentioned, calculations were done twice with the different configurations of the clusters, and the percent of change between both analyses, calculated.

### Clustering of ticks in the phylogenetic tree of the hosts genera

Since the number of species of vertebrates recorded in the final dataset is high, potentially introducing noise of difficult interpretation, we opted to use the genera of hosts for evaluating phylogenetic associations among ticks and hosts. We used the package “rotl”^35^ for R to query the website of the project “Open Tree of Life” (TOL at http://tree.opentreeoflife.org) and download a synthetic tree of hosts genera. However, the complete list of hosts genera in the dataset of ticks-hosts relationships was not available in TOL and some of the genera were not included in further calculations. The total number of hosts genera became reduced to 185, and the resulting phylogenetic tree obtained from TOL is available as Supplementary Fig. S1.

Measures of phylogenetic clustering were obtained with the mean pairwise distance (MPD) in the package “picante”^36^ for R. The measure is an estimation of the clustering of ticks to specific portions of the phylogenetic tree of hosts. It is an estimation of the amount of genetic variability that is shared by the hosts that the tick exploits. The purpose of calculating MPD is to demonstrate the hypothesis of segregation of the Neotropical ticks along to particular groups of vertebrates: a significant association with a portion of the phylogenetic tree of hosts would mean for a more or less strict association of the tick with these vertebrates. It is necessary to stress that if a tick species/stage uses a large number of hosts mostly belonging to close evolutive branches of the phylogenetic tree, values will be significant for clustering. However, it the tick uses a few hosts belonging to essentially different vertebrate clades, MPD will be not significant. The Faith’s index of phylogenetic diversity (PD) was also used. It represents the phylogenetic analogue of taxon richness and is expressed as the number of tree units which a species/stage of tick exploits^37^. In this application, PD is an evaluation of biodiversity in the branches of the phylogenetic tree of hosts exploited by ticks; MPD is an estimation of phylogeny-based diversity since it sums the distances among every vertebrates of the tree used by ticks and compares with the total length of the branches in the tree.

## Supplementary information

Supplementary Information.

Supplementary Information 2.

## Data Availability

The datasets analyzed during the current study are available at 10.5061/dryad.860473k. The data generated during the current study are included in this published article (and its Supplementary Information files).
