# Supplementary material for: A community approach to the Neotropical ticks-hosts interactions
Source: Sci Rep. 2020 Jun 9;10:9269. doi: 10.1038/s41598-020-66400-3 (PMC7283479; doi:10.1038/s41598-020-66400-3)
Supplement: Supplementary file 1 — Supplementary Information. [file 41598_2020_66400_MOESM1_ESM.zip › Supplementary Material Legend.pdf]

A community approach to the Neotropical ticks-hosts interactions

Agustín Estrada-Peña [1*], Santiago Nava [2], Evelina Tarragona [2], José de la Fuente [3,4], Alberto A. Guglielmone [2].

[1] Department of Animal Pathology. Faculty of Veterinary Medicine. Zaragoza (Spain)

[2] INTA, Rafaela, Santa Fe, Argentina.

[3] SaBio, Instituto de Investigación en Recursos Cinegéticos (IREC-CSIC-UCLM-JCCM), Ronda de Toledo s/n, 13005 Ciudad Real, Spain.

[4] Department of Veterinary Pathobiology, Center for Veterinary Health Sciences, Oklahoma State University, Stillwater, OK 74078, USA.

* Correspondence: Agustín Estrada-Peña. Department of Animal Pathology. Faculty of Veterinary Medicine. Miguel Servet, 177. 50013-Zaragoza (Spain); e-mail: aestrada@unizar.es

Supplementary Figure S1. The phylogenetic tree of the genera of hosts of Neotropical ticks for which available data exist in "The Open Tree of Life".

Supplementary Figure S2. The network of relationships among species of ticks and genera of hosts in the Neotropical region. Circles (nodes) are the organisms that interact through links (curved lines). They conform communities (colors) that are sets of organisms that interact more among themselves (the community) than with the rest of nodes. Color of lines follow the color of clusters, and the width of links is proportional to the intensity of interactions between nodes. Labels of both ticks species/stages and genera of vertebrates are included.

Supplementary Figure S3. A set of 125 phylogenetic trees of the hosts recorded for each species/stage of tick. The phylogenetic tree was generated and downloaded from The Open Tree of Life, and includes a total of 185 genera of vertebrates. Blue dots mean the hosts on which a species/stage of tick has been recorded. The size of the dot is proportional to the number of times the species/stage has been recorded on that host. Labels are in black are for exophilic ticks, labels in green are for endophilic ticks, labels in red are for genera of vertebrate hosts.

Supplementary Table S1. Indexes of phylogenetic clustering and centrality for every species and stage of Neotropical ticks that were included in the study. The columns include the species of ticks and its stage, the number of Genera of vertebrates in which it has been recorded, the observed mean pairwise distance with the hosts in the phylogenetic tree (mpd.obs), the mean pairwise distance observed in 100 null models (mpd.rand.mean) the ratio of the observed mean pairwise distance compared with the obtained null model (mpd.obs.z), the probability that the species/stage has phylogenetic clustering with the hosts (mpd.obs.p), the value of centrality (BNC), the community of the network to which the species/stage belongs (Community) and its status as exophilic (EXO), endophilic (ENDO) or unknown (?). Low values (negative) of mpd.obs.z and values of $p < 0.05$ are indicative of phylogenetic clustering
