# Supplementary material for: A community approach to the Neotropical ticks-hosts interactions
Source: Sci Rep. 2020 Jun 9;10:9269. doi: 10.1038/s41598-020-66400-3 (PMC7283479; doi:10.1038/s41598-020-66400-3)
Supplement: Supplementary file 1 — Supplementary Information. [file 41598_2020_66400_MOESM1_ESM.zip › Supplementary Table S1.pdf]

Supplementary Table S1

Species of ticks	Genera	mpd.obs	mpd.rand.mean	mpd.obs.z	mpd.obs.p	BNC	Community	Status
**Amblyomma argentinae (Adult)**	9	1.276	1.315	-0.229	0.25	3082.22	8	ENDO
**Amblyomma argentinae (Larva)**	2	1.251	1.345	-0.144	0.435	10.01	8	ENDO
**Amblyomma argentinae (Nymph)**	3	0.909	1.380	-1.294	0.17	48.90	8	ENDO
**Amblyomma aureolatum (Adult)**	17	0.601	1.336	-8.092	0.01	5349.79	9	EXO
**Amblyomma aureolatum (Larva)**	6	0.517	1.331	-4.391	0.01	818.42	1	ENDO
**Amblyomma aureolatum (Nymph)**	8	1.150	1.352	-1.392	0.11	6399.94	1	ENDO
**Amblyomma auricularium (Adult)**	12	0.465	1.360	-9.888	0.01	1739.65	2	ENDO
**Amblyomma auricularium (Larva)**	7	1.244	1.346	-0.809	0.18	1869.95	1	ENDO
**Amblyomma auricularium (Nymph)**	18	1.293	1.339	-0.708	0.21	11685.06	2	ENDO
**Amblyomma brasiliense (Adult)**	6	0.413	1.366	-5.430	0.01	325.90	5	EXO
**Amblyomma brasiliense (Nymph)**	15	0.686	1.351	-8.561	0.01	3415.00	9	EXO
**Amblyomma cajennense (Adult)**	11	0.490	1.339	-8.661	0.01	3209.92	5	EXO
**Amblyomma cajennense (Larva)**	2	1.978	1.293	0.971	0.64	144.81	9	EXO
**Amblyomma cajennense (Nymph)**	8	1.030	1.334	-2.045	0.05	2284.71	9	EXO
**Amblyomma calcaratum (Adult)**	6	0.460	1.342	-4.666	0.01	1780.66	3	EXO
**Amblyomma calcaratum (Larva)**	8	0.884	1.335	-3.064	0.03	1708.24	1	EXO
**Amblyomma calcaratum (Nymph)**	25	0.846	1.332	-7.300	0.01	20521.70	1	EXO
**Amblyomma coelebs (Adult)**	3	0.488	1.335	-2.135	0.05	68.61	5	EXO
**Amblyomma coelebs (Larva)**	2	0.894	1.295	-0.642	0.32	38.91	1	EXO
**Amblyomma coelebs (Nymph)**	17	0.964	1.343	-4.644	0.01	14618.67	9	EXO
**Amblyomma dissimile (Adult)**	23	1.004	1.329	-4.411	0.01	15659.60	8	ENDO
**Amblyomma dissimile (Larva)**	1	NA	NA	NA	NA	34.94	8	ENDO
**Amblyomma dissimile (Nymph)**	18	1.347	1.350	-0.046	0.41	21202.85	8	ENDO
**Amblyomma dubitatum (Adult)**	8	0.390	1.345	-6.671	0.01	1670.49	4	EXO
**Amblyomma dubitatum (Larva)**	15	0.871	1.347	-6.602	0.01	8177.16	4	EXO
**Amblyomma dubitatum (Nymph)**	25	0.597	1.350	-17.841	0.01	17179.56	4	EXO
**Amblyomma fuscum (Adult)**	4	1.771	1.346	1.428	0.99	2300.37	8	EXO
**Amblyomma fuscum (Larva)**	2	0.715	1.276	-0.846	0.28	12.66	4	ENDO
**Amblyomma fuscum (Nymph)**	6	0.465	1.351	-4.647	0.01	463.96	4	ENDO
**Amblyomma geayi (Adult)**	2	0.011	1.334	-1.885	0.01	1968.99	3	EXO
**Amblyomma geayi (Larva)**	8	1.092	1.347	-2.112	0.05	1951.89	1	ENDO
**Amblyomma geayi (Nymph)**	1	NA	NA	NA	NA	0.00	1	ENDO
**Amblyomma goeldii (Adult)**	5	1.403	1.386	0.082	0.43	2352.83	3	EXO
**Amblyomma humerale (Adult)**	6	1.318	1.325	-0.028	0.38	374.06	8	ENDO
**Amblyomma humerale (Larva)**	4	0.231	1.327	-4.105	0.01	428.93	1	ENDO
**Amblyomma humerale (Nymph)**	14	1.404	1.336	0.881	0.87	12706.68	1	ENDO
**Amblyomma incisum (Adult)**	2	0.570	1.397	-1.197	0.16	34.88	5	EXO
**Amblyomma incisum (Nymph)**	7	0.328	1.384	-7.627	0.01	556.81	5	EXO
**Amblyomma longirostre (Adult)**	10	1.388	1.346	0.384	0.57	4494.48	4	EXO
**Amblyomma longirostre (Larva)**	36	0.545	1.337	-17.114	0.01	23125.45	1	ENDO
**Amblyomma longirostre (Nymph)**	59	0.725	1.336	-21.003	0.01	77550.42	1	ENDO
**Amblyomma mixtum (Adult)**	10	0.676	1.355	-7.229	0.01	12182.38	5	EXO
**Amblyomma mixtum (Larva)**	1	NA	NA	NA	NA	0.00	4	EXO
**Amblyomma naponense (Adult)**	9	0.415	1.359	-9.691	0.01	1002.90	5	EXO
**Amblyomma naponense (Larva)**	2	0.570	1.236	-0.974	0.155	50.52	4	EXO
**Amblyomma naponense (Nymph)**	11	0.718	1.338	-6.185	0.01	3677.10	5	EXO
**Amblyomma neumanni (Adult)**	7	0.334	1.369	-5.217	0.02	478.97	5	EXO
**Amblyomma neumanni (Larva)**	2	0.324	1.348	-1.362	0.19	13.94	5	EXO

**Amblyomma neumanni (Nymph)**	4	0.460	1.302	-2.652	0.02	84.65	5	EXO
**Amblyomma nodosum (Adult)**	4	0.061	1.374	-5.195	0.01	457.42	3	EXO
**Amblyomma nodosum (Larva)**	6	0.506	1.299	-3.452	0.01	471.47	1	EXO
**Amblyomma nodosum (Nymph)**	25	0.726	1.329	-8.655	0.01	18729.95	1	EXO
**Amblyomma oblongoguttatum (Adult)**	6	0.904	1.304	-1.787	0.06	1829.00	5	EXO
**Amblyomma oblongoguttatum (Nymph)**	3	0.488	1.363	-2.549	0.03	78.26	5	EXO
**Amblyomma ovale (Adult)**	23	0.649	1.347	-11.067	0.01	19111.21	9	EXO
**Amblyomma ovale (Larva)**	7	1.335	1.346	-0.069	0.37	4741.28	4	ENDO
**Amblyomma ovale (Nymph)**	15	1.084	1.350	-3.318	0.02	21946.08	4	EXO
**Amblyomma pacae (Adult)**	3	0.469	1.385	-2.291	0.06	104.40	4	ENDO
**Amblyomma pacae (Larva)**	0	NA	NA	NA	NA	0.00	3	ENDO
**Amblyomma pacae (Nymph)**	3	0.521	1.384	-2.412	0.03	591.34	4	ENDO
**Amblyomma parkeri (Adult)**	1	NA	NA	NA	NA	46.18	4	EXO
**Amblyomma parkeri (Larva)**	10	0.535	1.338	-5.971	0.01	1548.18	1	ENDO
**Amblyomma parkeri (Nymph)**	2	0.715	1.209	-0.733	0.34	532.94	9	ENDO
**Amblyomma parvitarsum (Adult)**	0	NA	NA	NA	NA	4943.56	5	ENDO
**Amblyomma parvitarsum (Larva)**	0	NA	NA	NA	NA	0.00	8	ENDO
**Amblyomma parvitarsum (Nymph)**	0	NA	NA	NA	NA	0.00	8	ENDO
**Amblyomma parvum (Adult)**	24	0.410	1.353	-18.821	0.01	11486.69	9	EXO
**Amblyomma parvum (Larva)**	5	1.464	1.342	0.531	0.69	5980.59	1	ENDO
**Amblyomma parvum (Nymph)**	25	1.294	1.337	-0.899	0.14	21646.90	1	ENDO
**Amblyomma pseudoconcolor (Adult)**	10	0.405	1.354	-9.016	0.01	7814.71	2	ENDO
**Amblyomma pseudoconcolor (Larva)**	1	NA	NA	NA	NA	178.01	2	ENDO
**Amblyomma pseudoconcolor (Nymph)**	3	0.700	1.349	-1.818	0.1	2227.59	2	ENDO
**Amblyomma pseudoparvum (Adult)**	4	0.547	1.327	-2.566	0.03	211.95	4	ENDO
**Amblyomma pseudoparvum (Larva)**	2	0.570	1.255	-0.977	0.18	60.79	5	ENDO
**Amblyomma pseudoparvum (Nymph)**	4	0.333	1.351	-3.603	0.01	185.73	4	ENDO
**Amblyomma romitti (Adult)**	2	0.715	1.430	-1.050	0.22	46.29	4	?
**Amblyomma romitti (Nymph)**	1	NA	NA	NA	NA	0.00	4	?
**Amblyomma rotundatum (Adult)**	19	1.364	1.330	0.410	0.64	41472.47	8	ENDO
**Amblyomma rotundatum (Larva)**	2	0.011	1.282	-1.957	0.01	6.33	8	ENDO
**Amblyomma rotundatum (Nymph)**	8	1.146	1.313	-0.808	0.14	717.66	8	ENDO
**Amblyomma scalpturatum (Adult)**	3	0.637	1.367	-1.898	0.095	70.91	5	EXO
**Amblyomma scalpturatum (Nymph)**	4	0.317	1.370	-4.195	0.01	160.59	4	EXO
**Amblyomma sculptum (Adult)**	19	0.479	1.352	-13.818	0.01	7080.65	3	EXO
**Amblyomma sculptum (Larva)**	2	1.978	1.424	0.880	0.57	63.65	5	EXO
**Amblyomma sculptum (Nymph)**	21	0.648	1.354	-11.688	0.01	11479.98	3	EXO
**Amblyomma tigrinum (Adult)**	11	0.254	1.342	-12.125	0.01	3081.66	9	EXO
**Amblyomma tigrinum (Larva)**	6	1.274	1.312	-0.182	0.31	25316.96	1	ENDO
**Amblyomma tigrinum (Nymph)**	6	1.347	1.335	0.065	0.4	22414.67	1	ENDO
**Amblyomma triste (Adult)**	12	0.357	1.340	-9.413	0.01	1727.23	9	EXO
**Amblyomma triste (Larva)**	7	1.219	1.343	-0.830	0.17	3841.34	4	ENDO
**Amblyomma triste (Nymph)**	10	1.047	1.361	-3.052	0.02	7279.75	4	ENDO
**Amblyomma varium (Adult)**	2	0.011	1.253	-1.701	0.01	425.02	3	EXO
**Amblyomma varium (Nymph)**	2	1.978	1.417	0.853	0.85	70.48	3	ENDO
**Haemaphysalis juxtakochi (Adult)**	11	0.586	1.360	-9.355	0.01	11516.87	5	EXO
**Haemaphysalis juxtakochi (Larva)**	5	1.404	1.345	0.272	0.54	3905.98	5	EXO
**Haemaphysalis juxtakochi (Nymph)**	8	0.816	1.329	-3.172	0.02	6371.24	5	EXO
**Haemaphysalis leporispalustris (Adult)**	3	0.454	1.372	-2.467	0.03	1340.33	2	ENDO
**Haemaphysalis leporispalustris (Larva)**	3	0.521	1.320	-1.866	0.13	3319.27	1	ENDO
**Haemaphysalis leporispalustris (Nymph)**	2	0.715	1.327	-0.954	0.25	2517.12	2	ENDO
**Ixodes amarali (Adult)**	2	0.034	1.344	-1.906	0.01	660.12	4	ENDO

**Ixodes amarali (Larva)**	3	0.499	1.349	-2.226	0.06	938.03	4	ENDO
**Ixodes amarali (Nymph)**	2	0.715	1.347	-0.930	0.28	438.89	4	ENDO
**Ixodes aragaoi (Adult)**	2	0.324	1.382	-1.615	0.09	44.05	5	EXO
**Ixodes aragaoi (Larva)**	0	NA	NA	NA	NA	387.59	1	EXO
**Ixodes aragaoi (Nymph)**	3	0.667	1.355	-1.759	0.12	1732.13	1	EXO
**Ixodes auritulus (Adult)**	1	NA	NA	NA	NA	14937.75	1	EXO
**Ixodes auritulus (Larva)**	5	0.745	1.309	-2.087	0.08	13514.66	1	EXO
**Ixodes auritulus (Nymph)**	6	1.144	1.327	-0.803	0.18	13696.21	1	EXO
**Ixodes fossulatus (Adult)**	2	0.212	1.292	-1.567	0.09	114.17	4	ENDO
**Ixodes fossulatus (Larva)**	1	NA	NA	NA	NA	32.29	4	ENDO
**Ixodes fossulatus (Nymph)**	4	1.566	1.317	0.780	0.89	1874.08	1	ENDO
**Ixodes longiscutatus (Adult)**	1	NA	NA	NA	NA	0.00	4	ENDO
**Ixodes longiscutatus (Larva)**	3	0.164	1.393	-3.632	0.01	283.75	4	ENDO
**Ixodes longiscutatus (Nymph)**	3	0.164	1.333	-3.110	0.01	99.71	4	ENDO
**Ixodes loricatus (Adult)**	6	0.560	1.335	-3.403	0.01	7407.20	4	EXO
**Ixodes loricatus (Larva)**	6	0.444	1.355	-5.359	0.01	1957.82	4	EXO
**Ixodes loricatus (Nymph)**	6	0.444	1.349	-4.905	0.01	2715.88	4	EXO
**Ixodes luciae (Adult)**	5	0.302	1.335	-5.092	0.01	2251.75	4	ENDO
**Ixodes luciae (Larva)**	2	0.034	1.323	-1.765	0.025	1252.85	4	ENDO
**Ixodes luciae (Nymph)**	4	0.577	1.369	-3.040	0.03	2677.90	4	ENDO
**Ixodes neuquenensis (Adult)**	0	NA	NA	NA	NA	0.00	6	ENDO
**Ixodes neuquenensis (Larva)**	0	NA	NA	NA	NA	0.00	6	ENDO
**Ixodes neuquenensis (Nymph)**	0	NA	NA	NA	NA	0.00	6	ENDO
**Ixodes paranaensis (Adult)**	0	NA	NA	NA	NA	1.33	7	?
**Ixodes paranaensis (Larva)**	0	NA	NA	NA	NA	0.00	7	?
**Ixodes paranaensis (Nymph)**	0	NA	NA	NA	NA	1.33	7	?
**Ixodes pararicinus (Adult)**	2	0.101	1.351	-1.881	0.05	1532.25	5	EXO
**Ixodes pararicinus (Larva)**	3	1.557	1.330	0.546	0.68	5413.19	1	EXO
**Ixodes pararicinus (Nymph)**	0	NA	NA	NA	NA	163.74	4	EXO
**Ixodes sigelos (Adult)**	1	NA	NA	NA	NA	973.64	4	ENDO
**Ixodes sigelos (Larva)**	1	NA	NA	NA	NA	1805.47	4	ENDO
**Ixodes sigelos (Nymph)**	1	NA	NA	NA	NA	2804.21	4	ENDO
**Ixodes stilesi (Adult)**	0	NA	NA	NA	NA	0.00	5	?
**Ixodes stilesi (Larva)**	0	NA	NA	NA	NA	0.00	5	?
**Ixodes stilesi (Nymph)**	1	NA	NA	NA	NA	1816.12	5	?
**Ixodes taglei (Adult)**	0	NA	NA	NA	NA	0.00	5	?
**Ixodes tapirus (Adult)**	1	NA	NA	NA	NA	0.00	5	ENDO
**Ixodes tropicalis (Adult)**	0	NA	NA	NA	NA	502.26	4	ENDO
**Ixodes tropicalis (Larva)**	0	NA	NA	NA	NA	0.00	4	ENDO
**Ixodes tropicalis (Nymph)**	0	NA	NA	NA	NA	0.00	4	ENDO
**Ixodes venezuelensis (Adult)**	1	NA	NA	NA	NA	1584.15	4	ENDO
**Ixodes venezuelensis (Larva)**	5	0.488	1.363	-4.251	0.01	1640.29	4	ENDO
**Ixodes venezuelensis (Nymph)**	5	0.460	1.350	-4.170	0.01	2499.10	4	ENDO
