# Supplementary figures and images for: A community approach to the Neotropical ticks-hosts interactions
Source: Sci Rep. 2020 Jun 9;10:9269. doi: 10.1038/s41598-020-66400-3 (PMC7283479; doi:10.1038/s41598-020-66400-3)

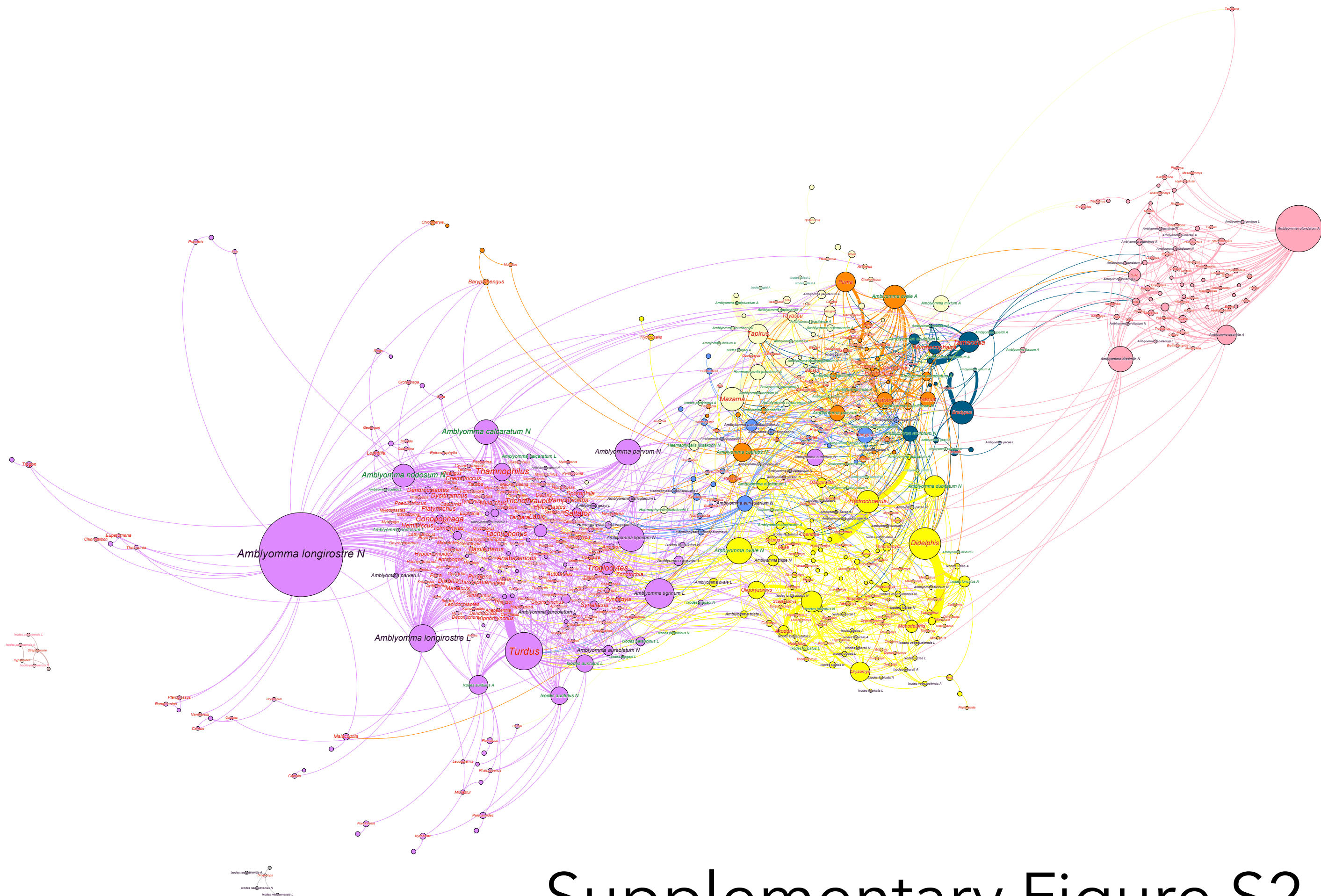

Supplement: Supplementary file 1 — Supplementary Information. [file 41598_2020_66400_MOESM1_ESM.zip › Supplementary Figure S2.pdf]
