# Supplementary material for: A community approach to the Neotropical ticks-hosts interactions
Source: Sci Rep. 2020 Jun 9;10:9269. doi: 10.1038/s41598-020-66400-3 (PMC7283479; doi:10.1038/s41598-020-66400-3)

Amblyomma argentinae adult

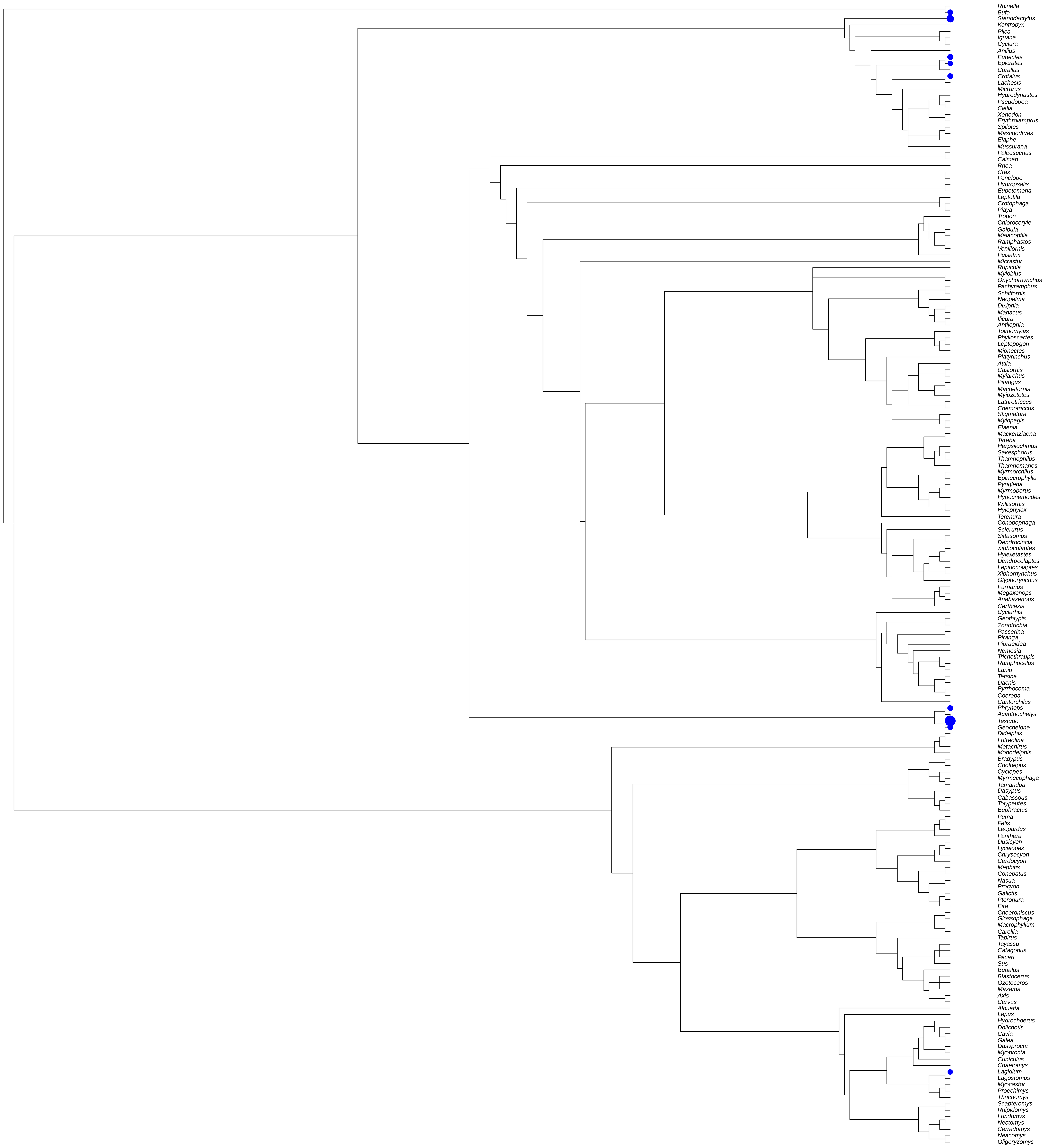

Amblyomma argentinae larva

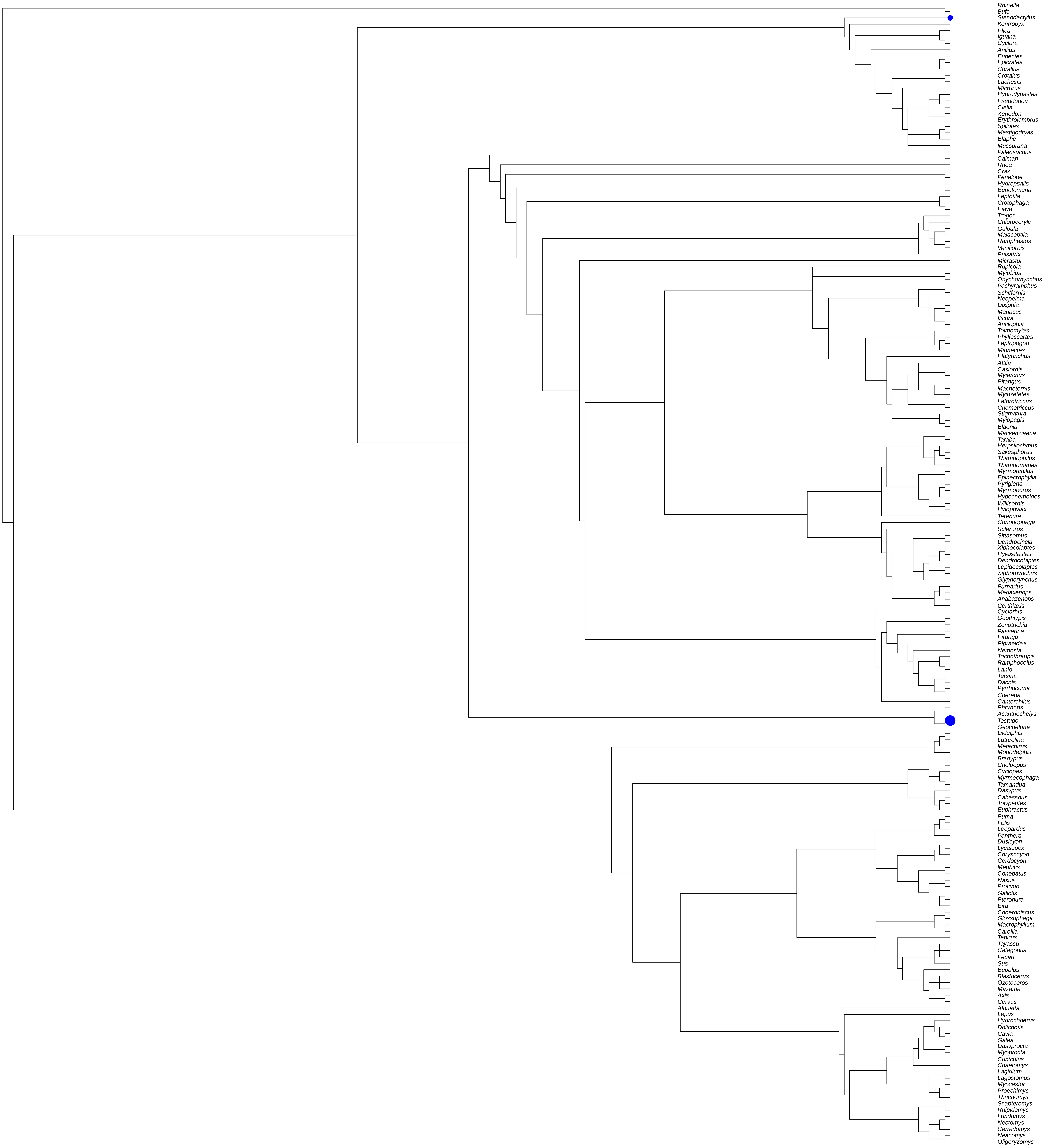

Amblyomma argentinae nymph

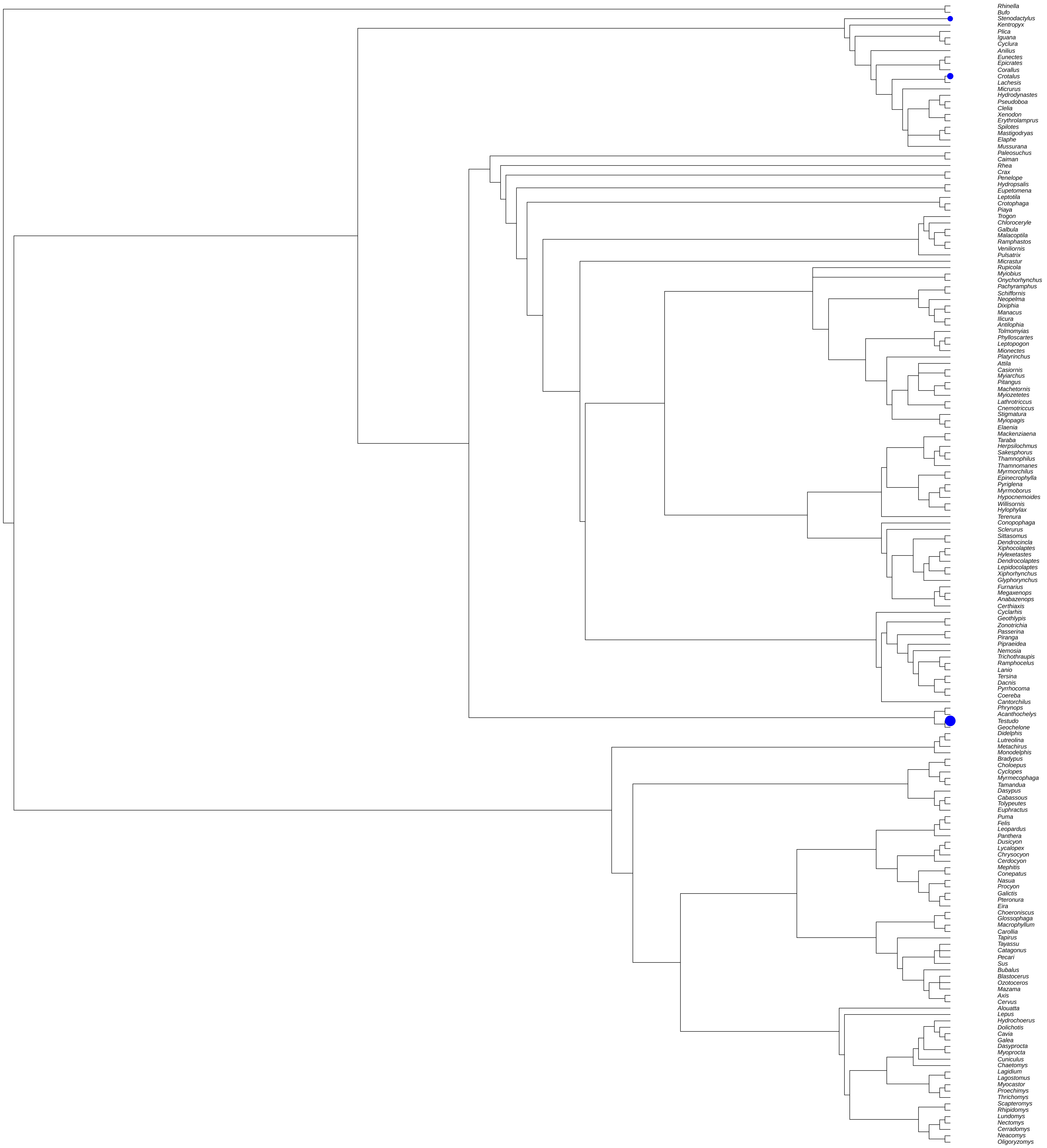

Amblyomma aureolatum adult

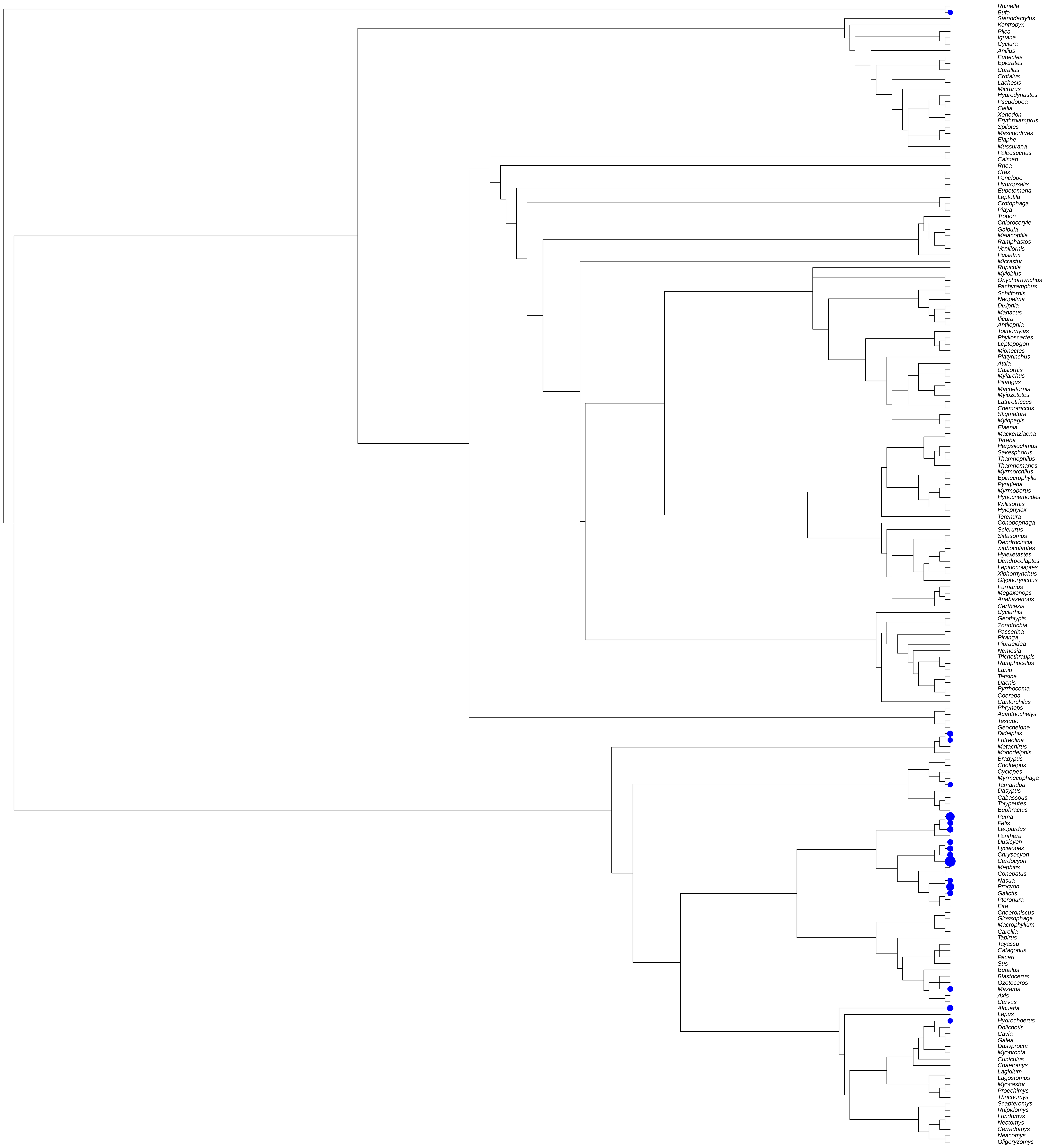

Amblyomma aureolatum larva

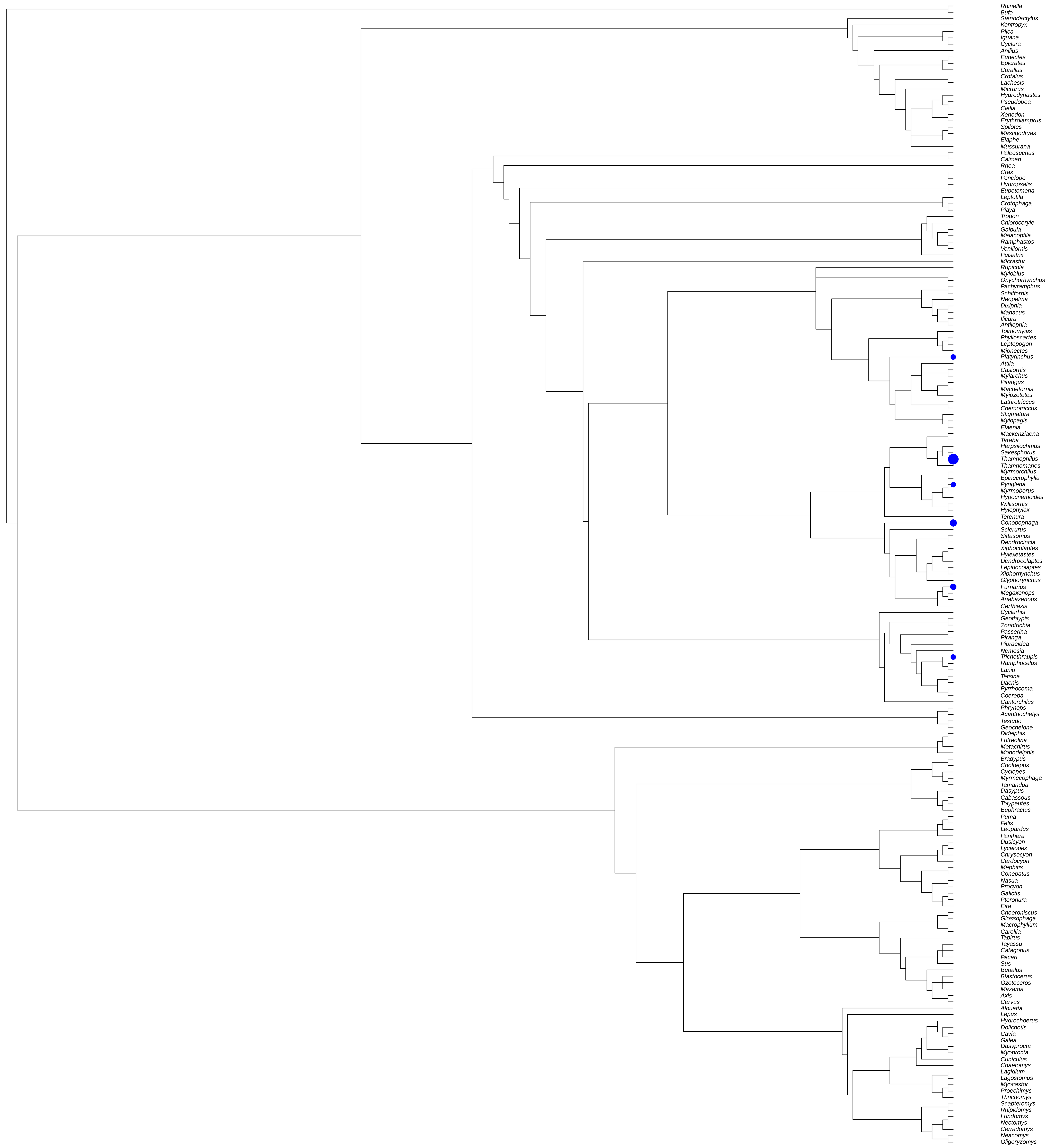

Amblyomma aureolatum nymph

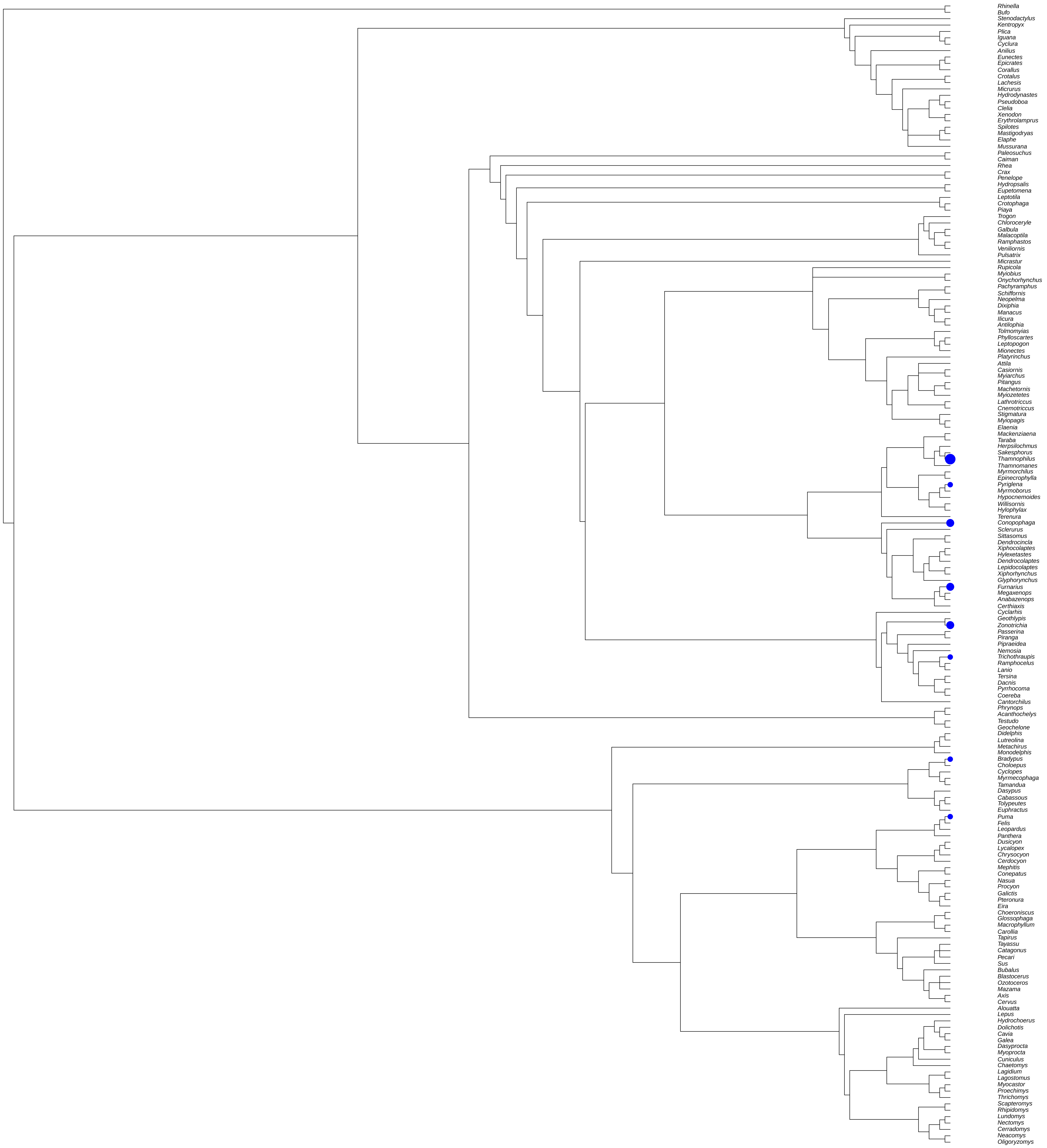

Amblyomma auricularium adult

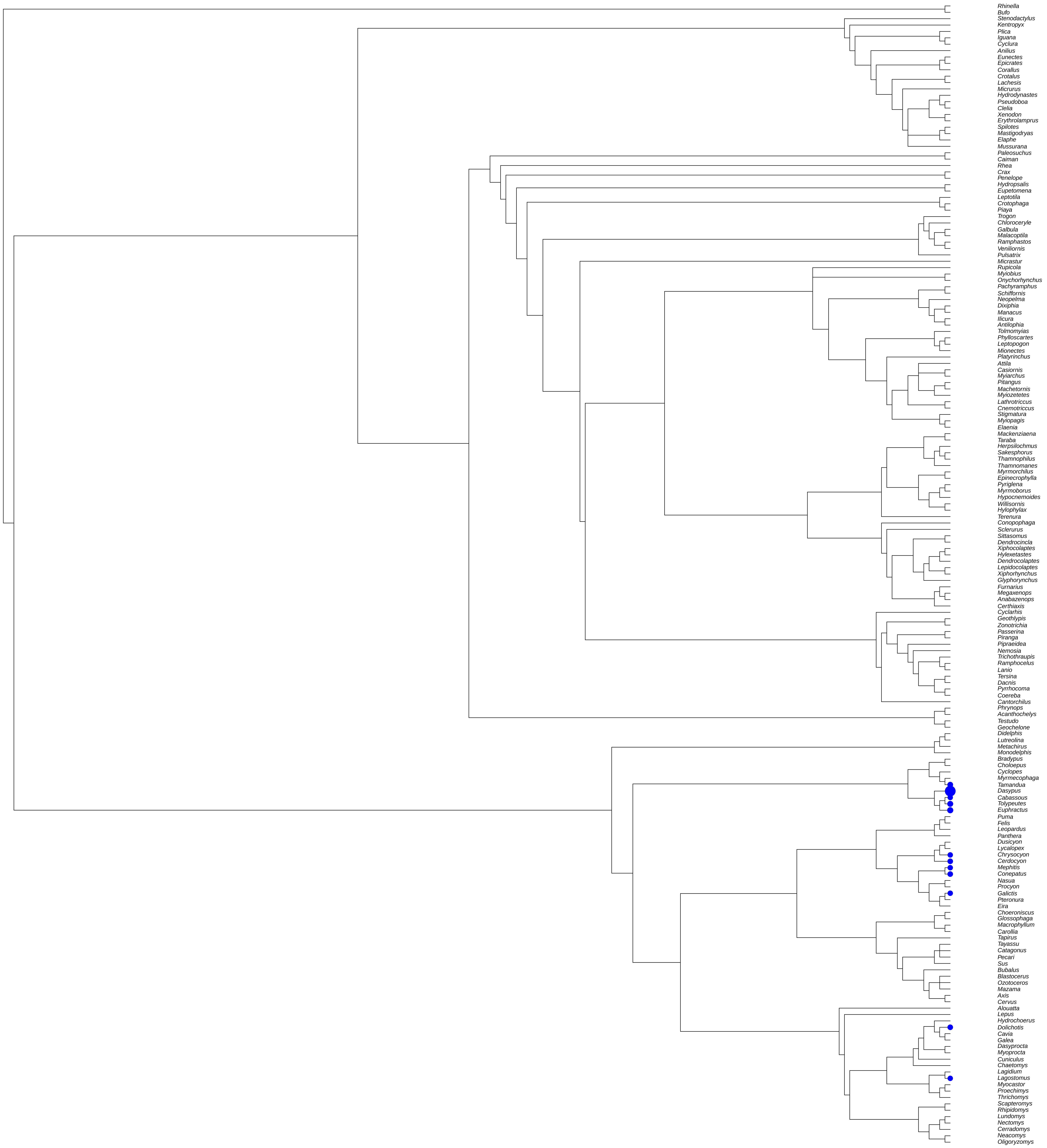

Amblyomma auricularium larva

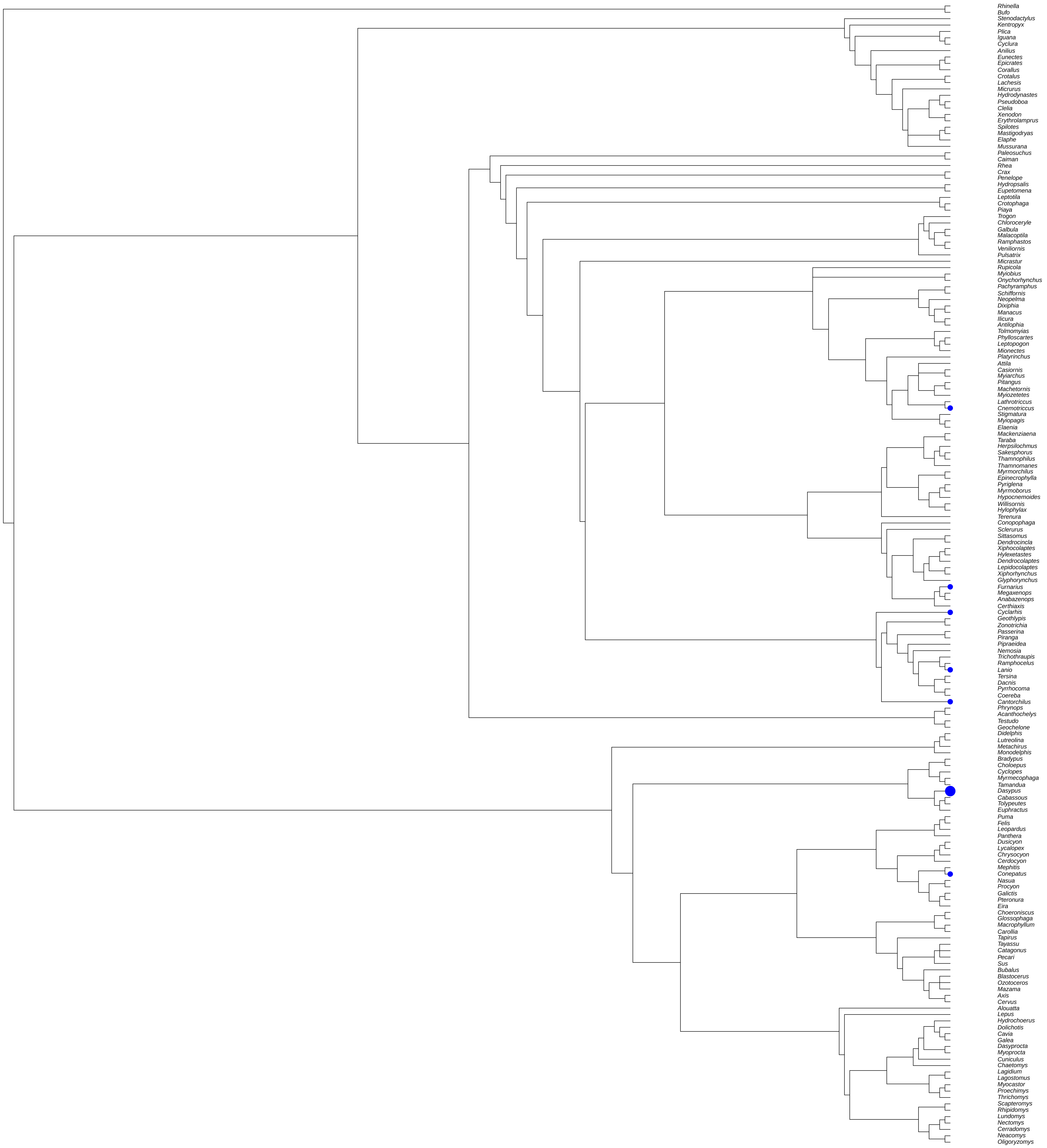

Amblyomma auricularium nymph

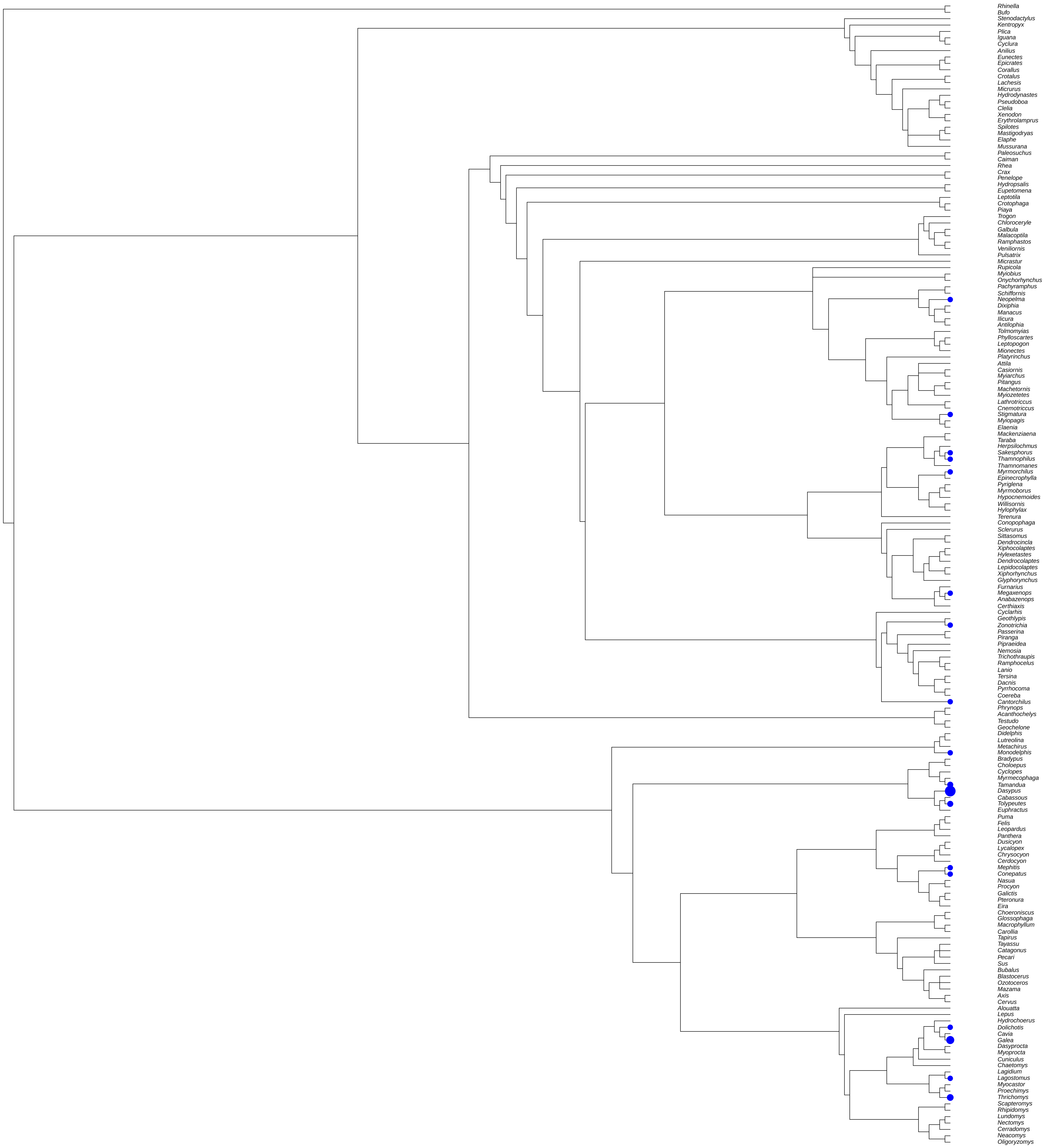

Amblyomma brasiliense adult

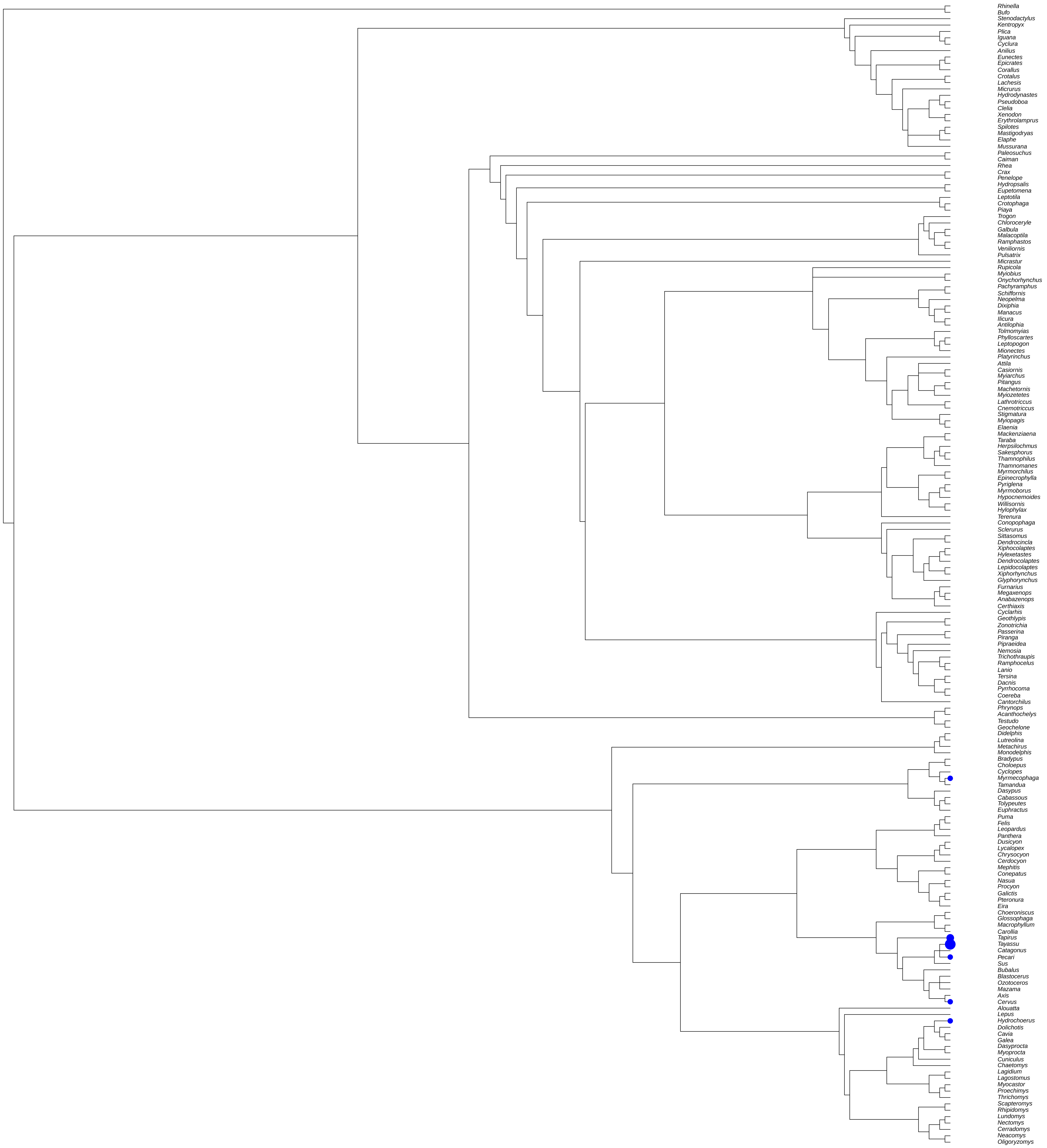

Amblyomma brasiliense nymph

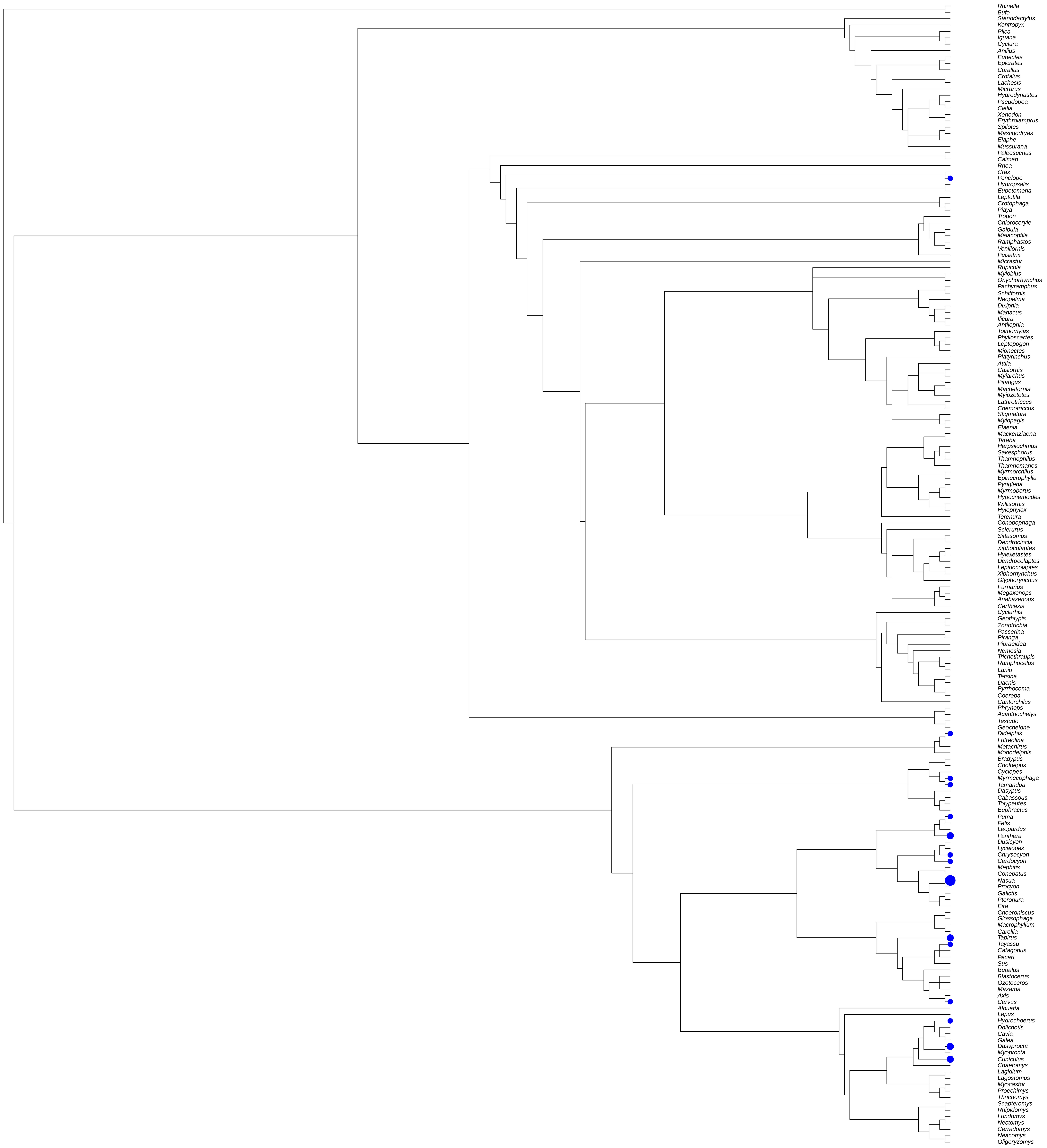

Amblyomma cajennense adult

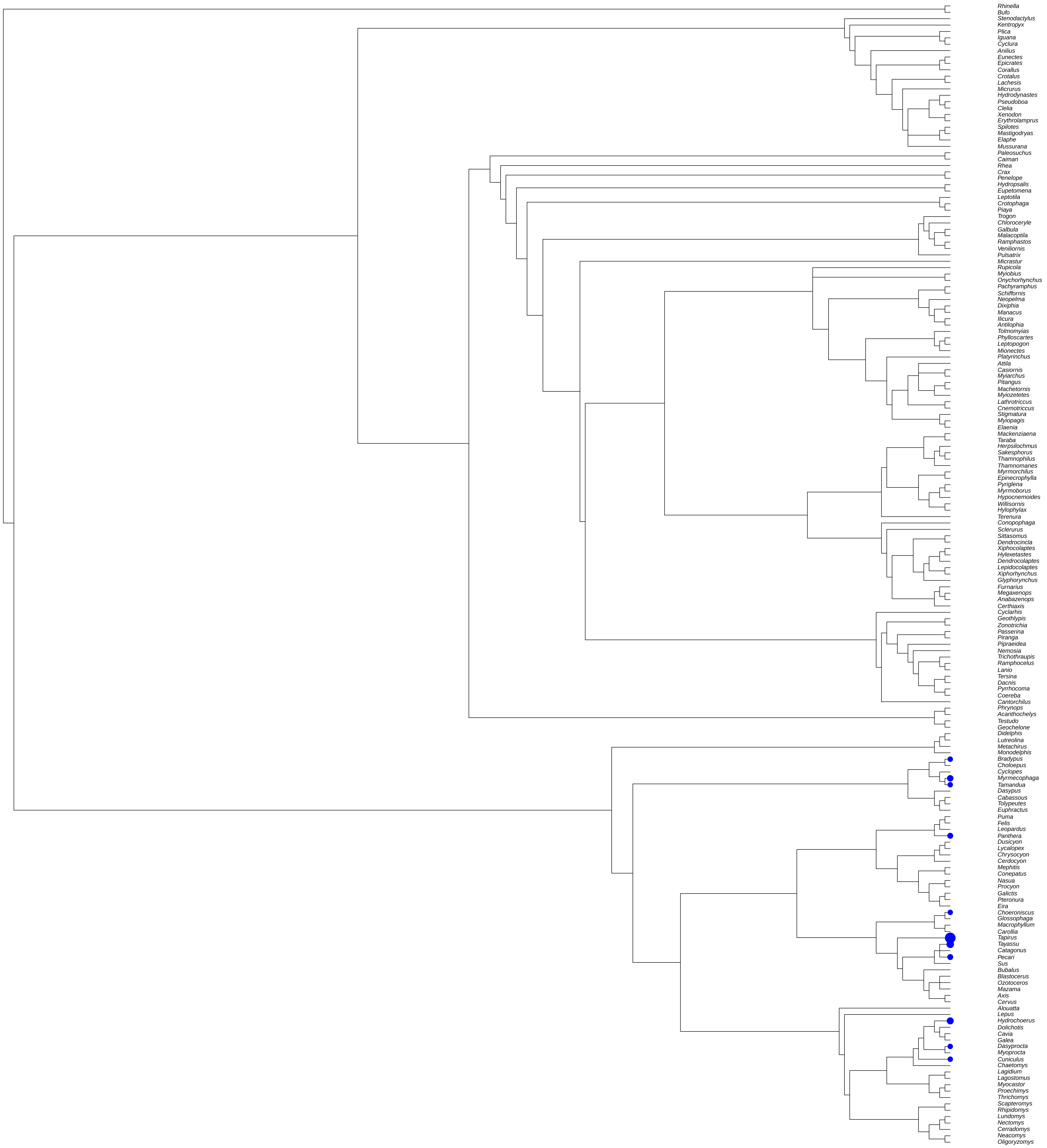

Amblyomma cajennense larva

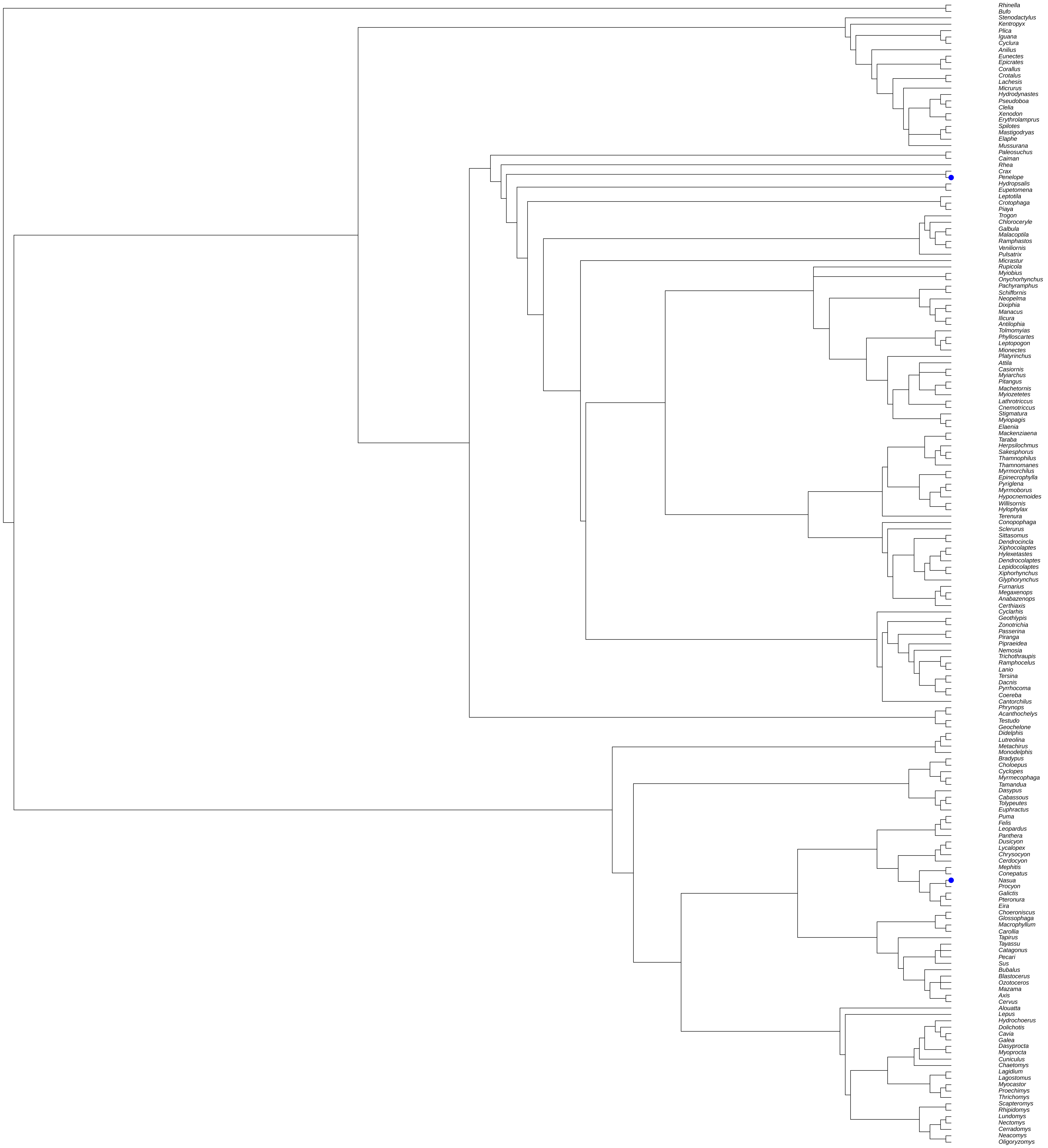

Amblyomma cajennense nymph

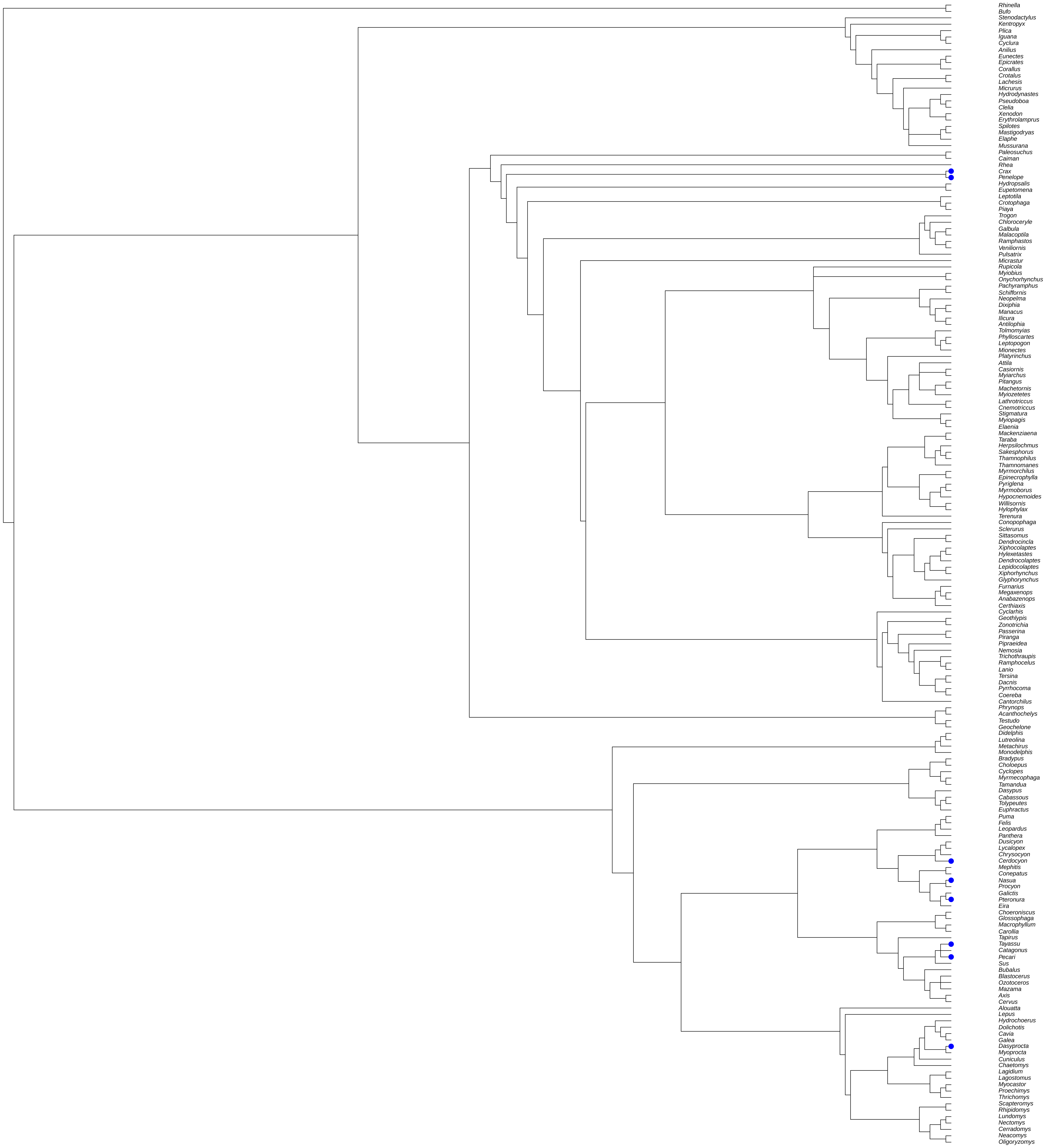

Amblyomma calcaratum adult

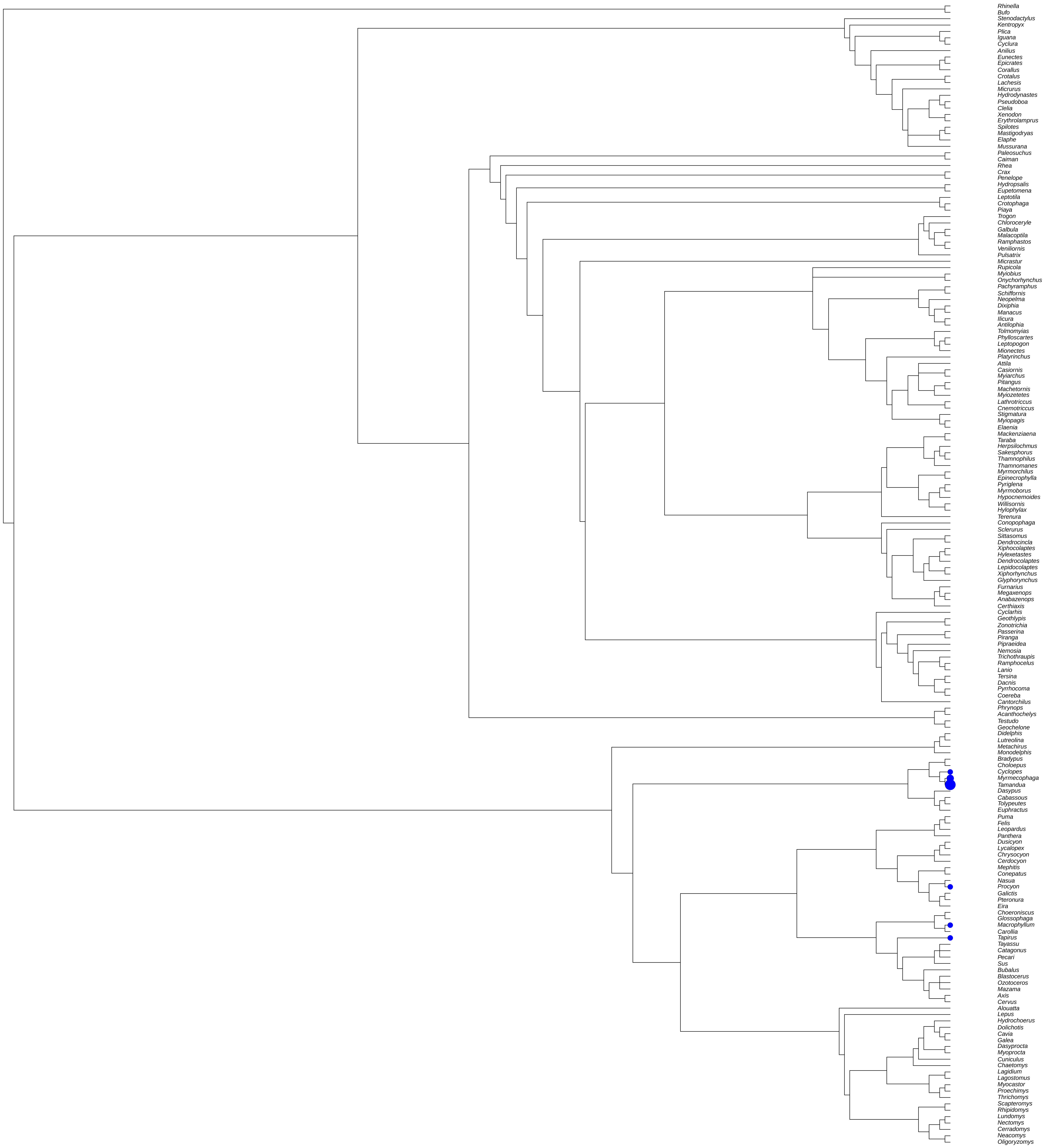

Amblyomma calcaratum larva

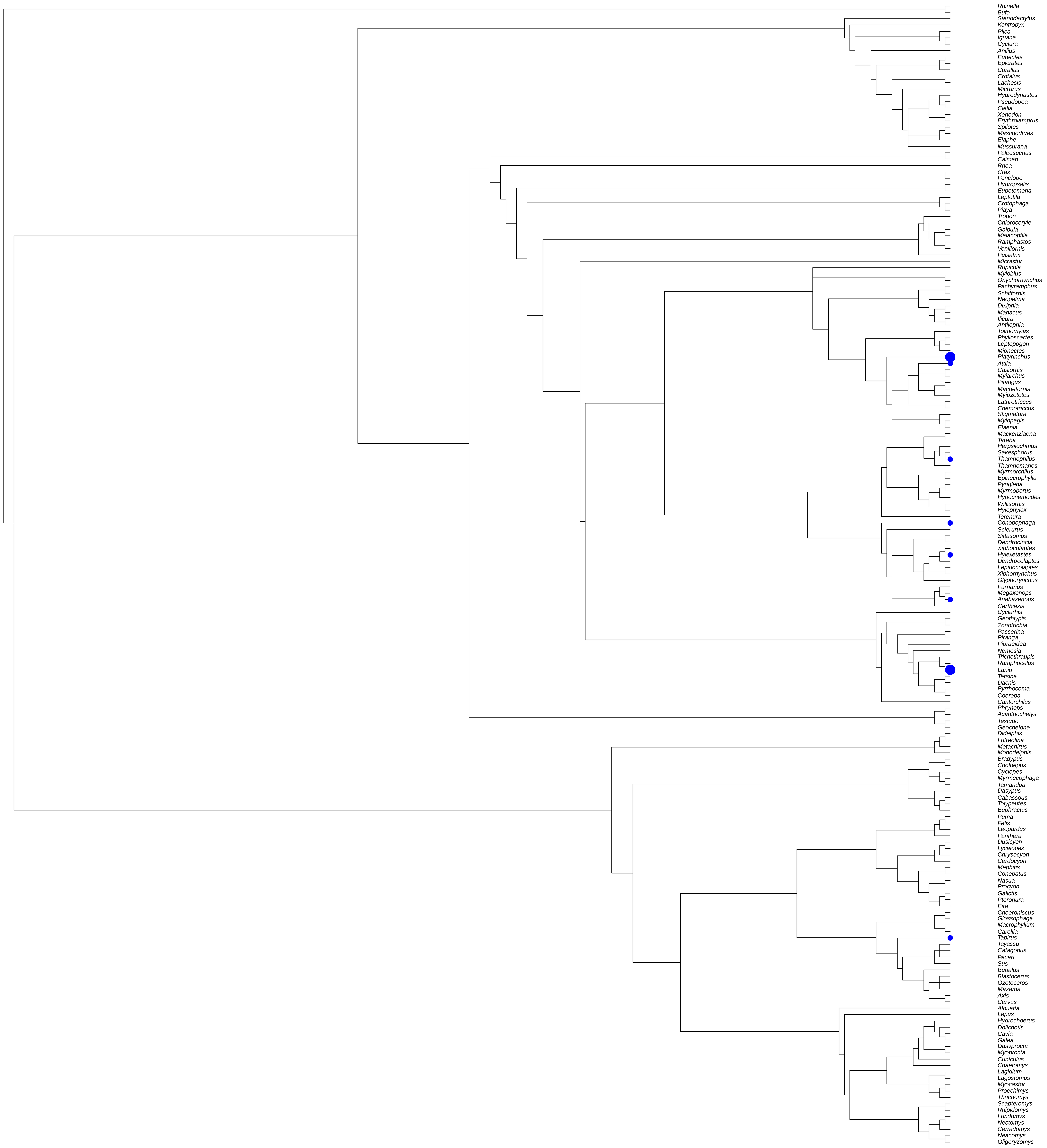

Amblyomma calcaratum nymph

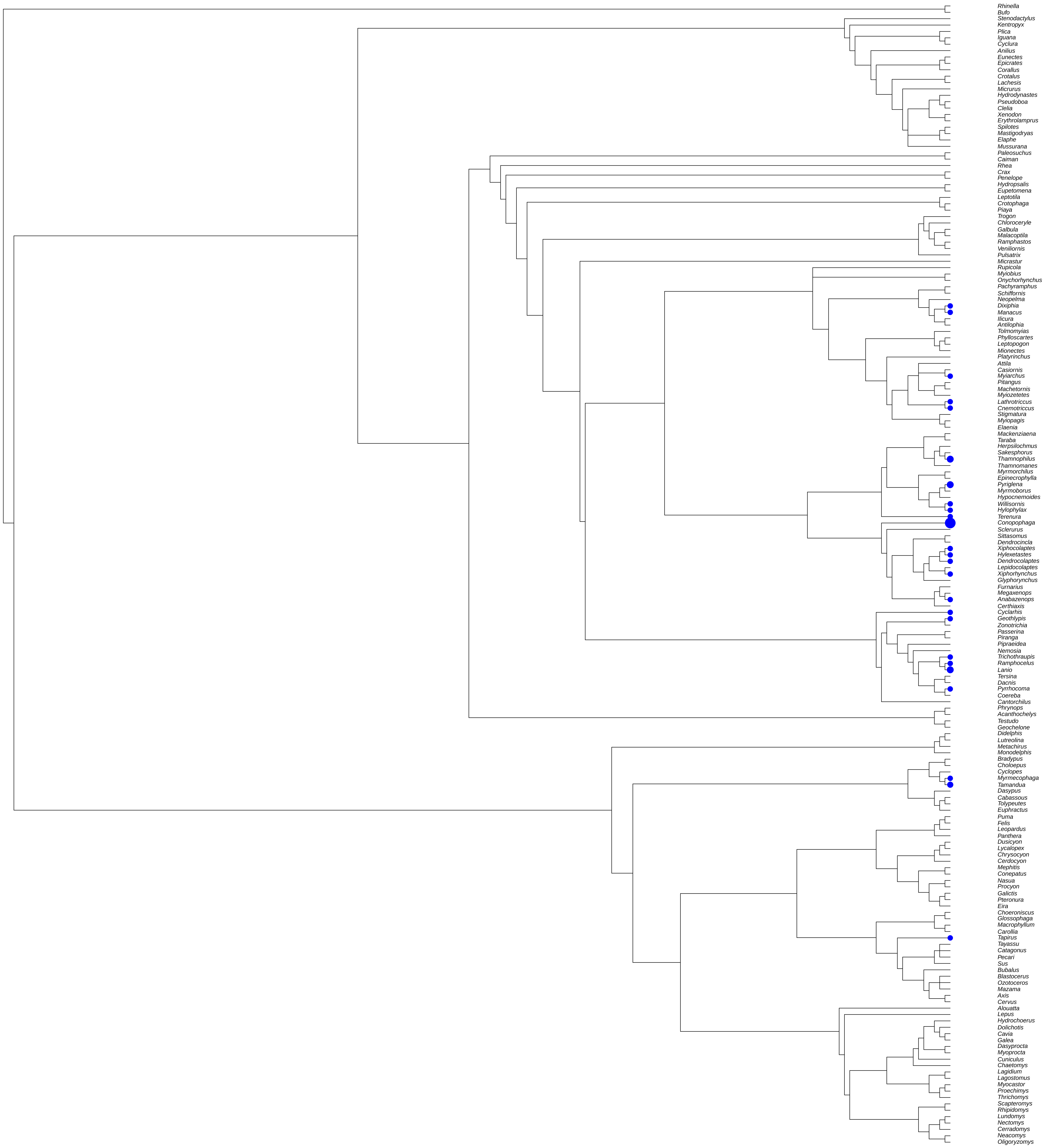

Amblyomma coelebs adult

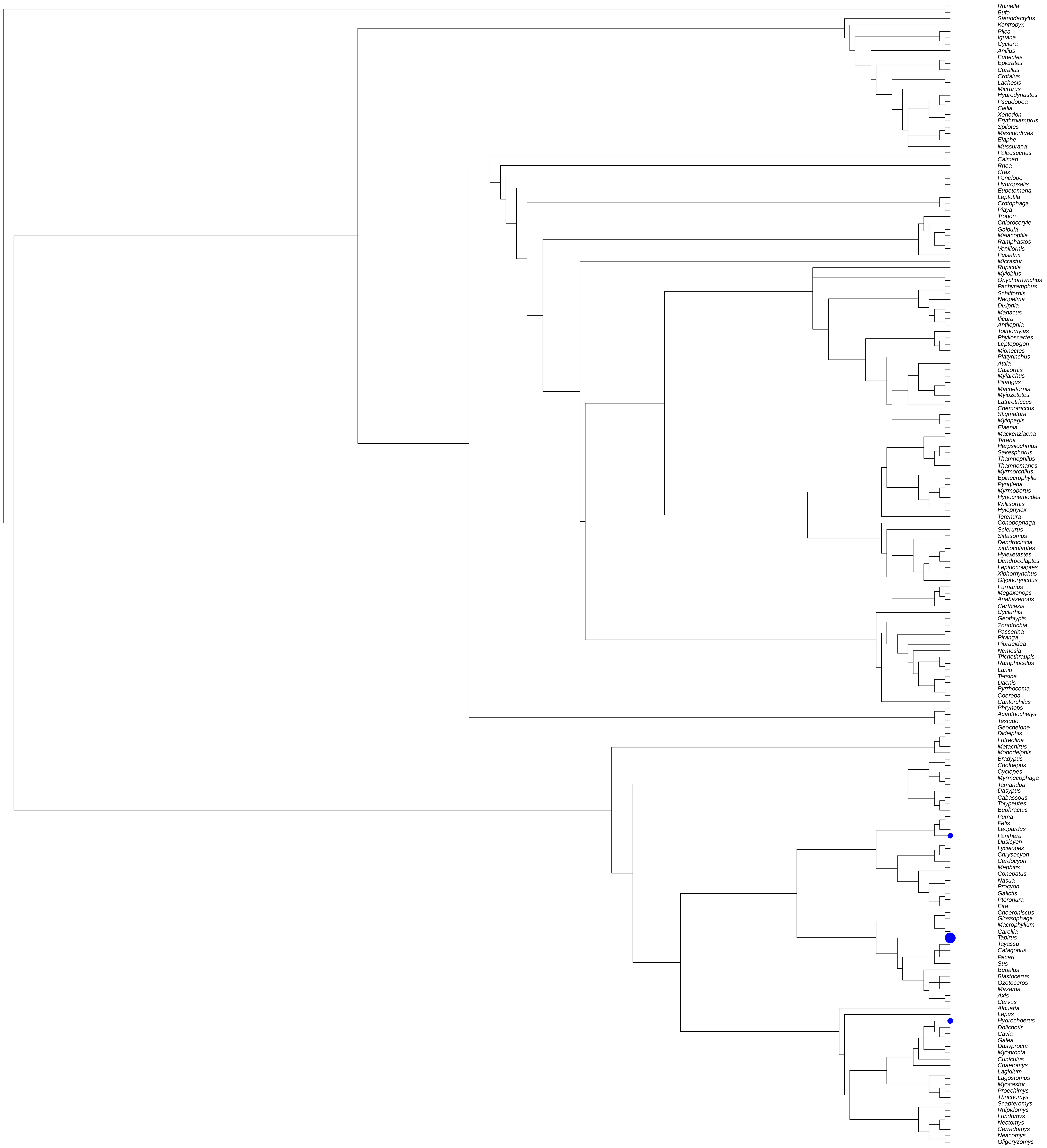

Amblyomma coelebs larva

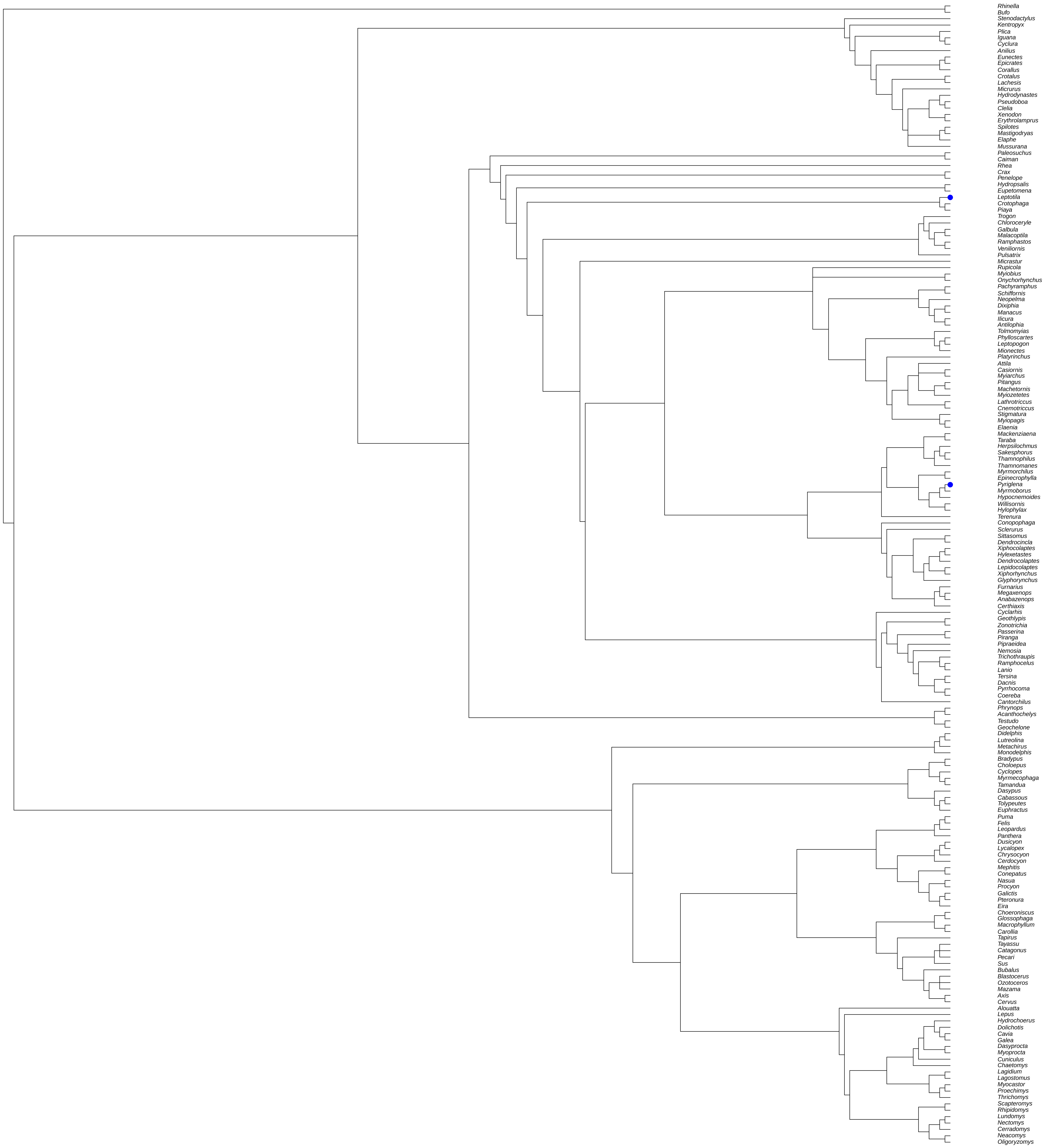

Amblyomma coelebs nymph

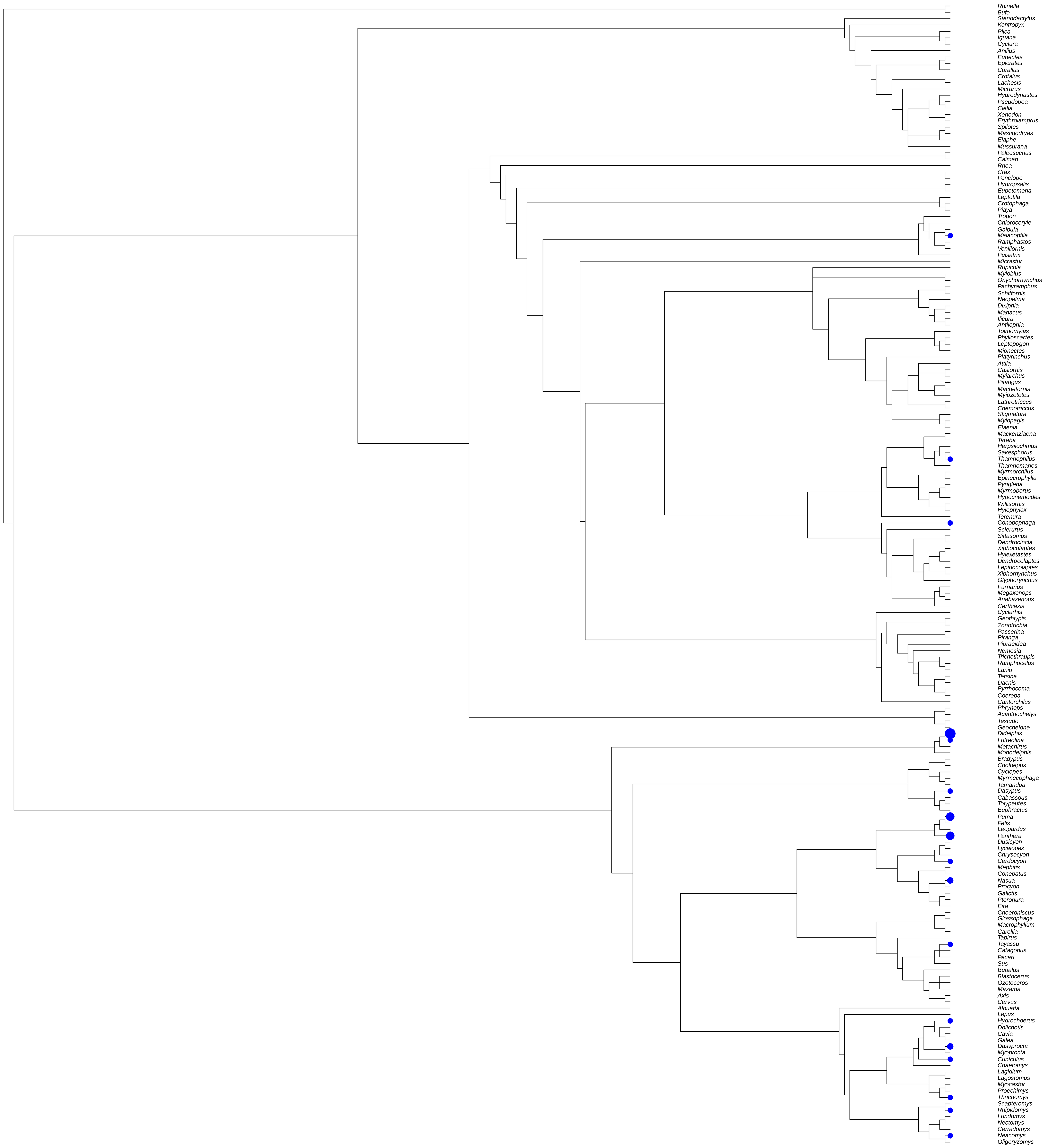

Amblyomma dissimile adult

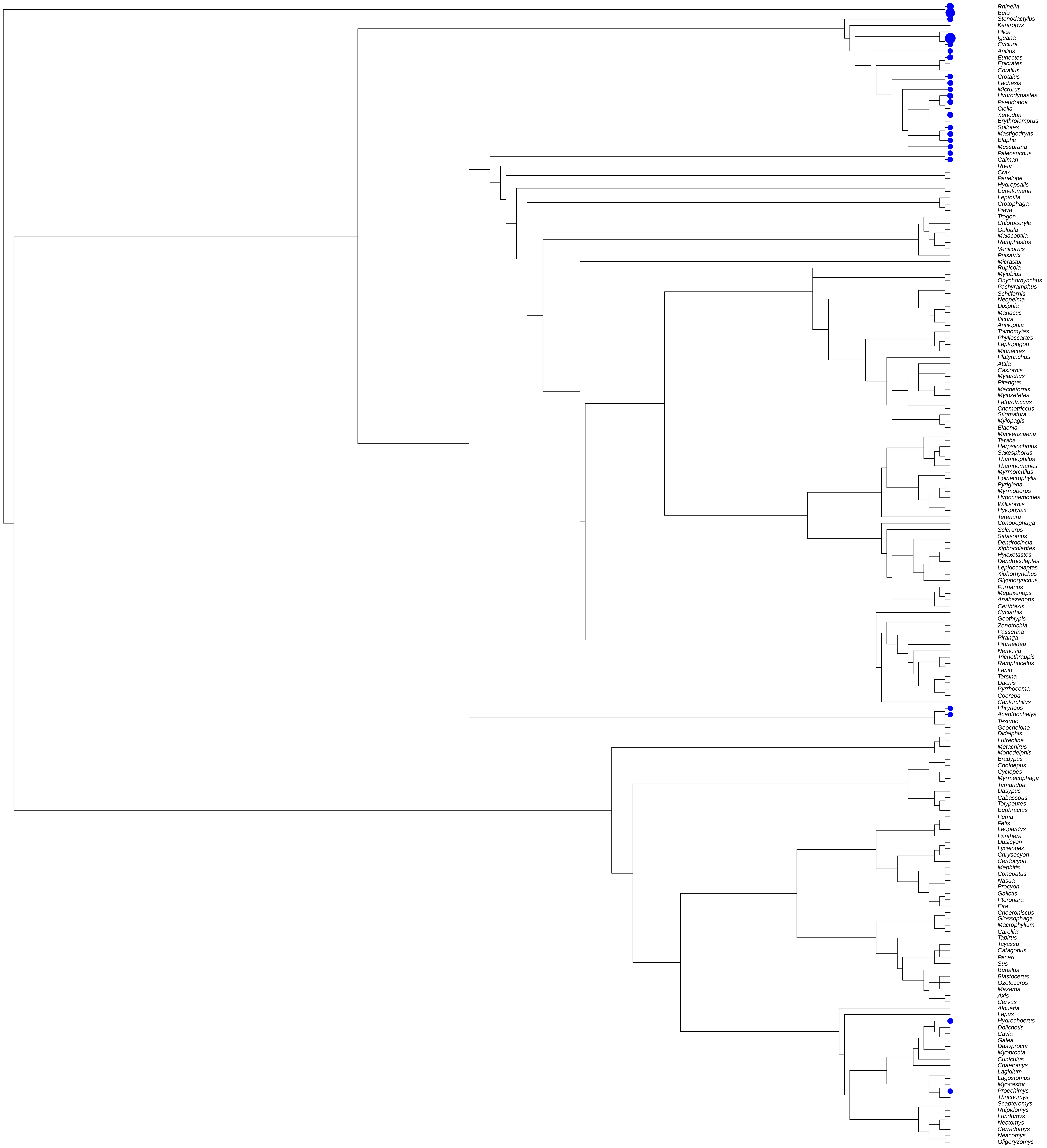

Amblyomma dissimile larva

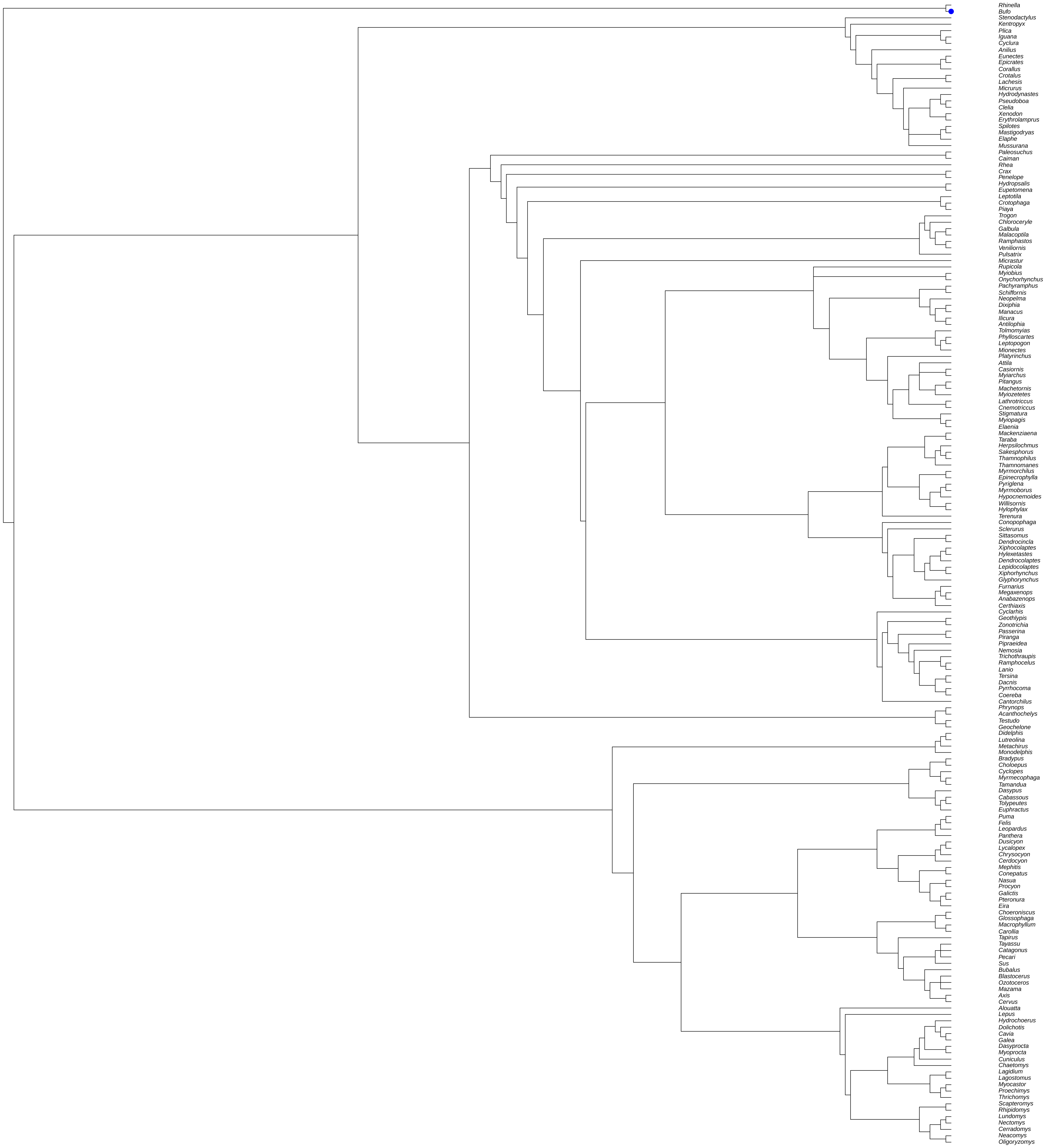

Amblyomma dissimile nymph

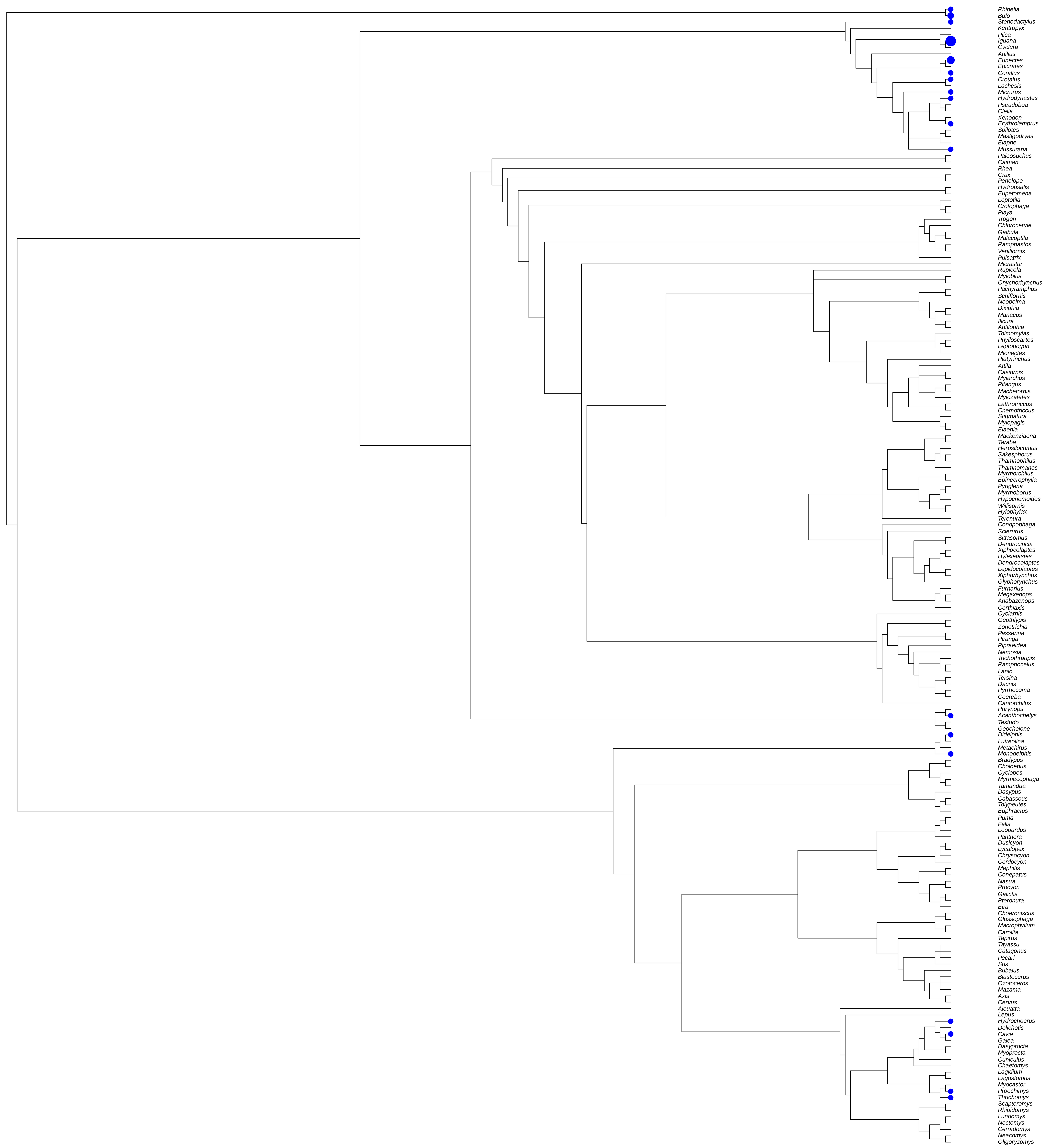

Amblyomma dubitatum adult

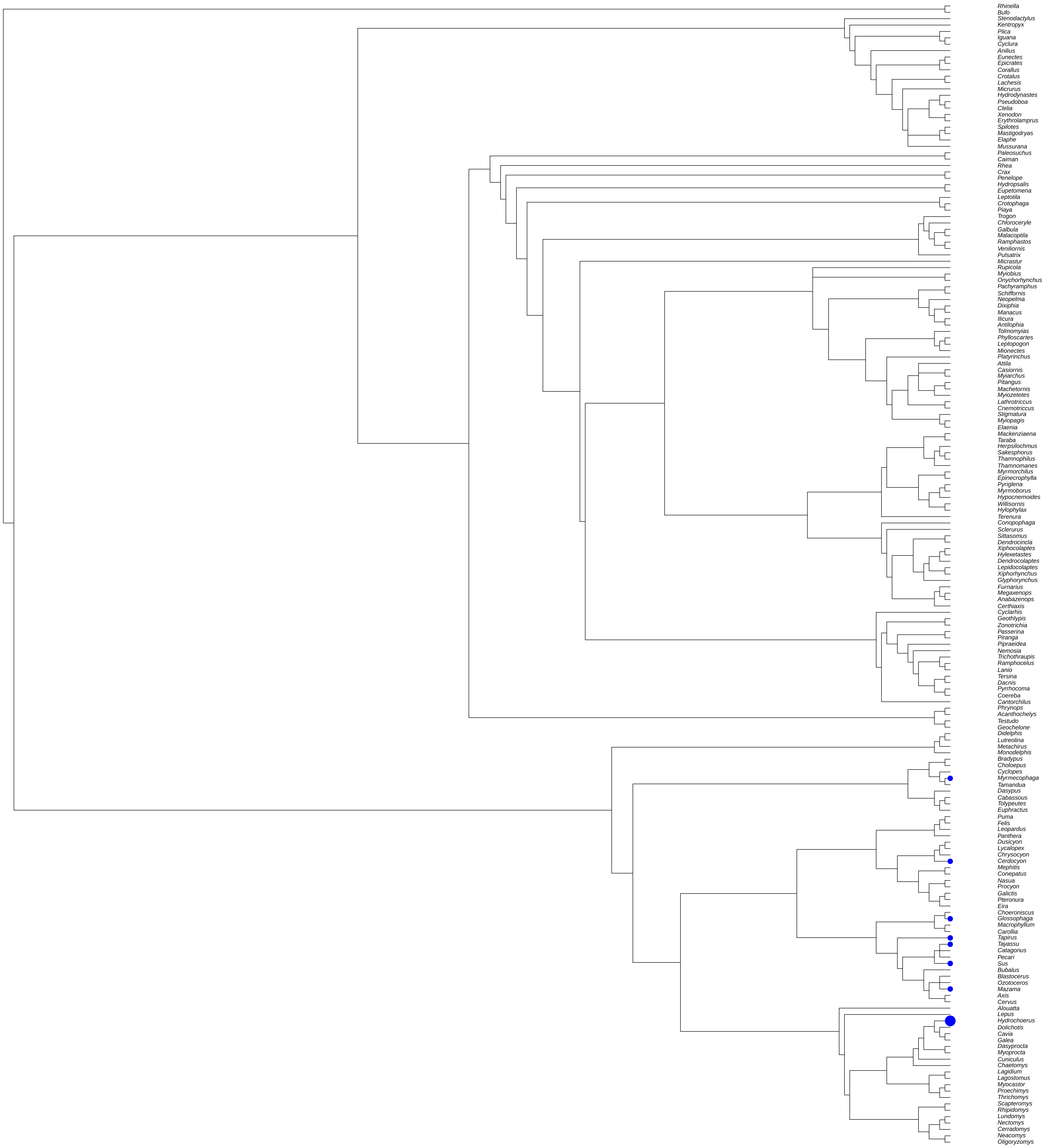

Amblyomma dubitatum larva

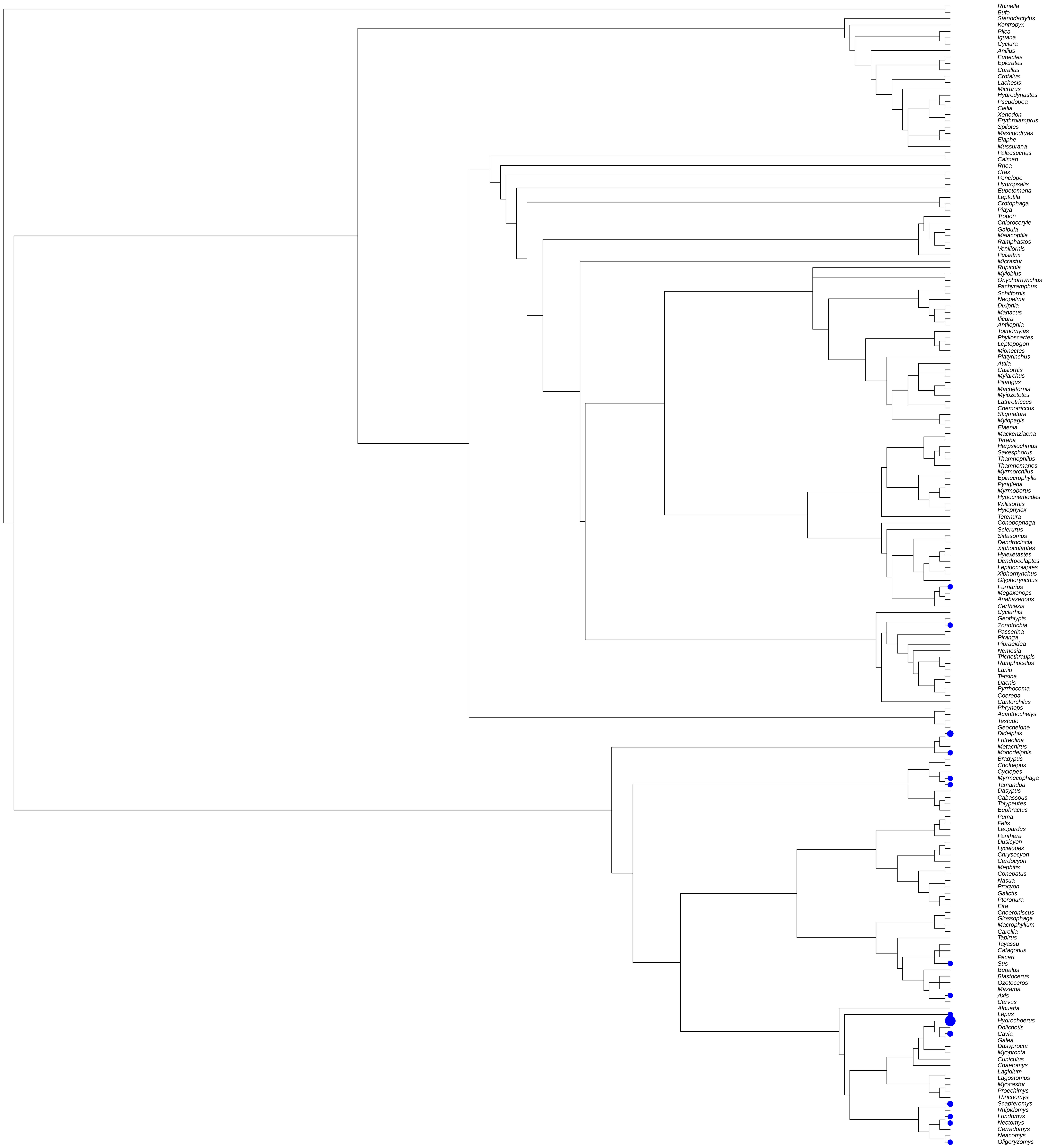

Amblyomma dubitatum nymph

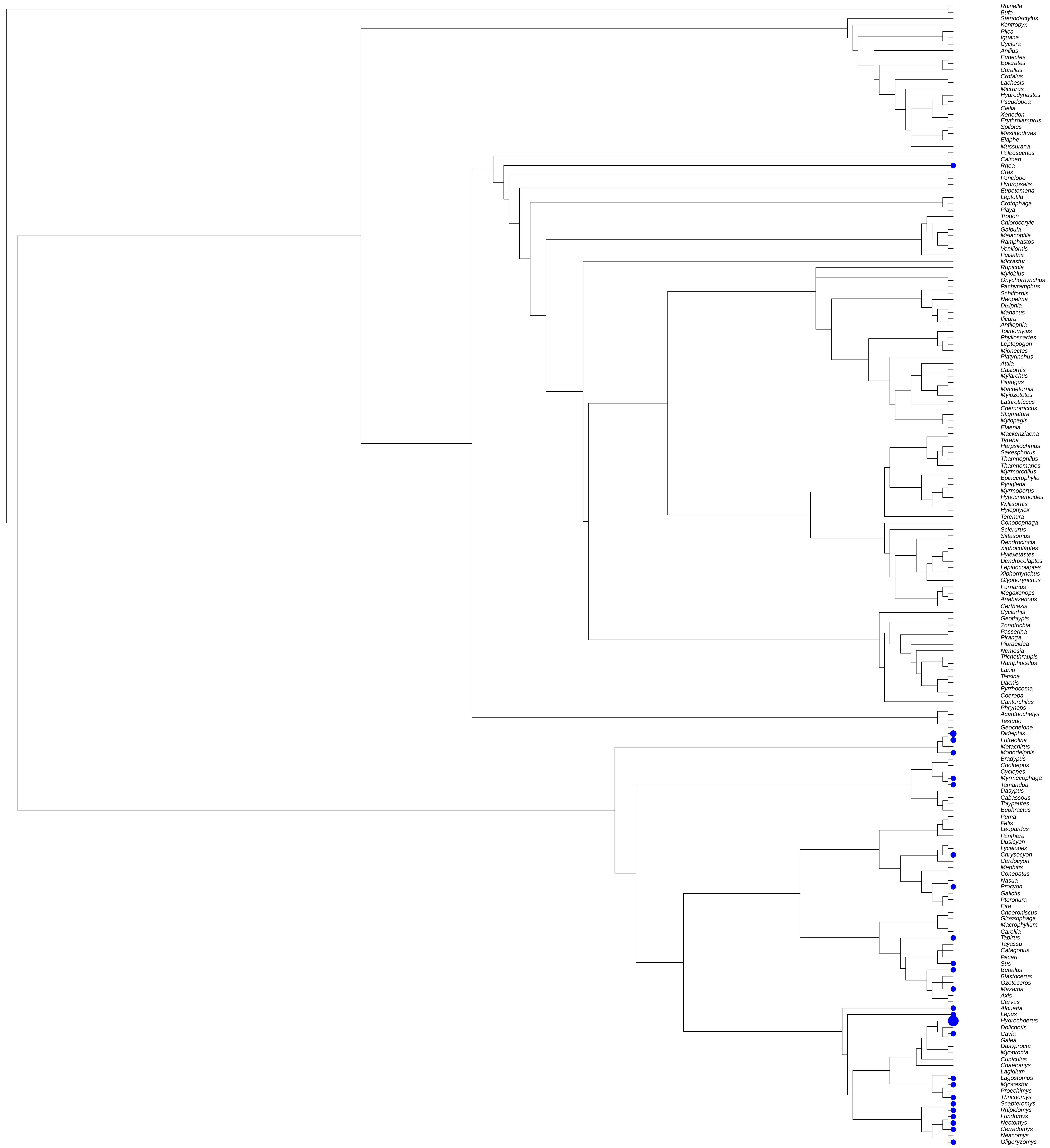

Amblyomma fuscum adult

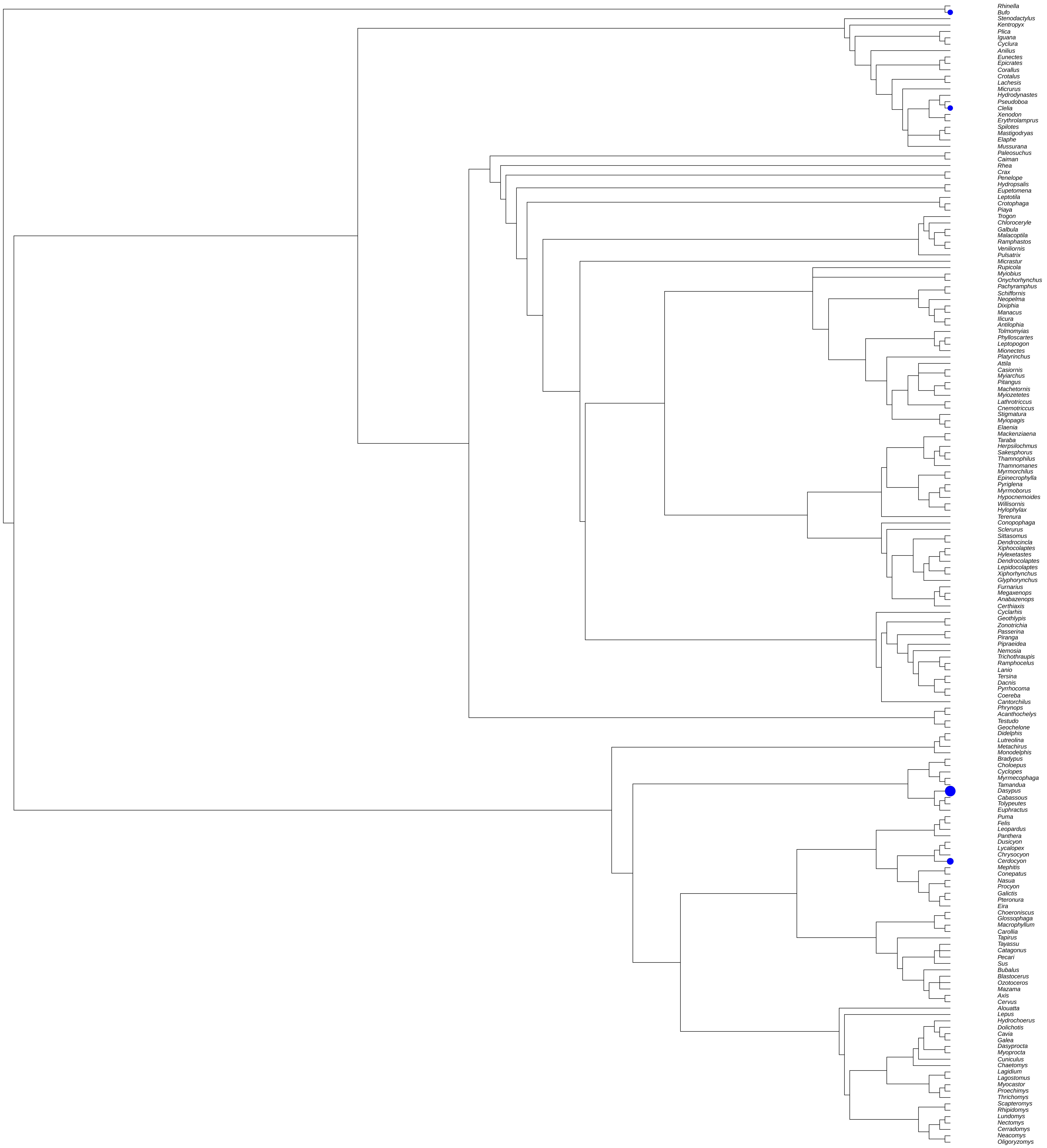

Amblyomma fuscum larva

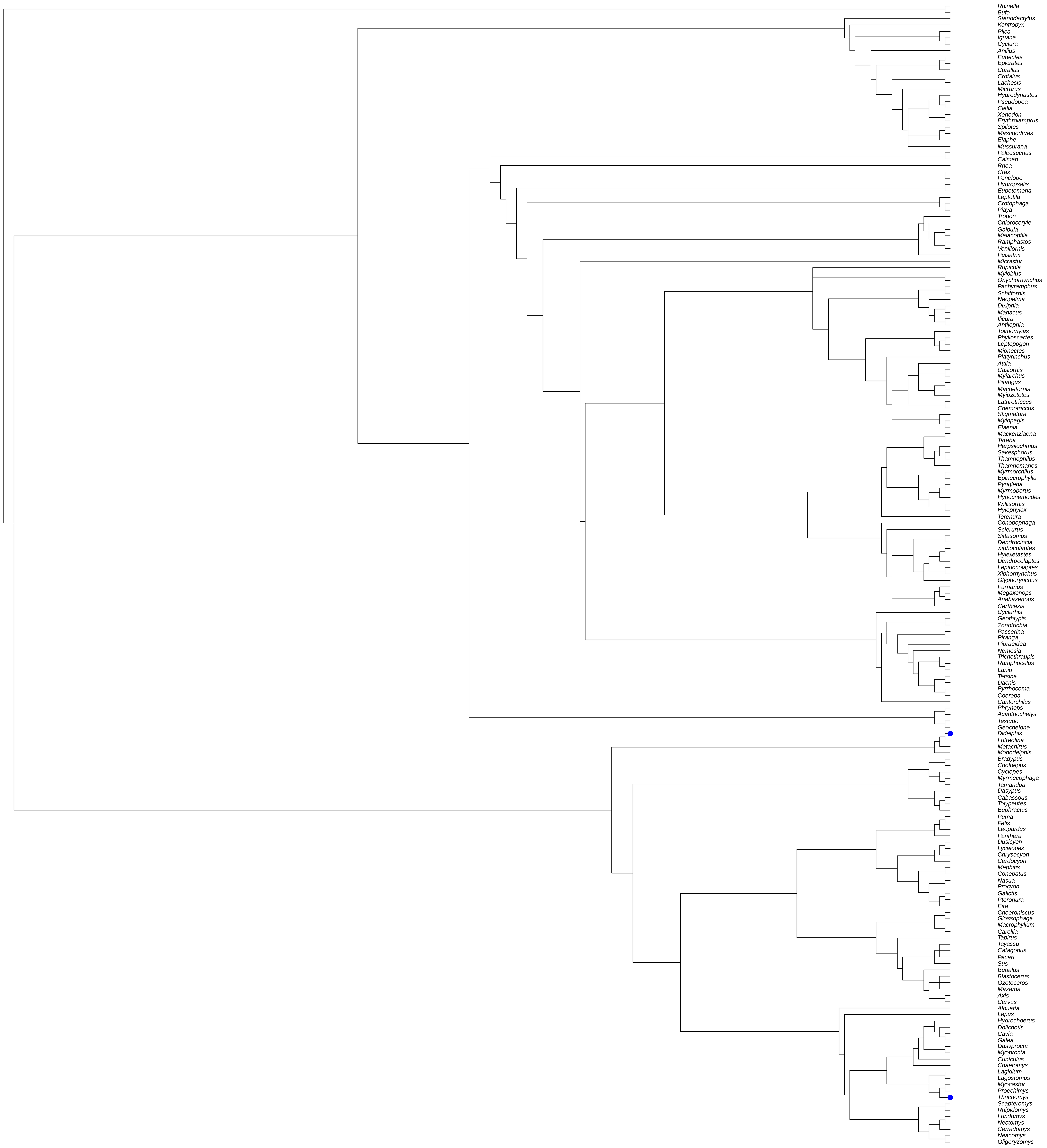

Amblyomma fuscum nymph

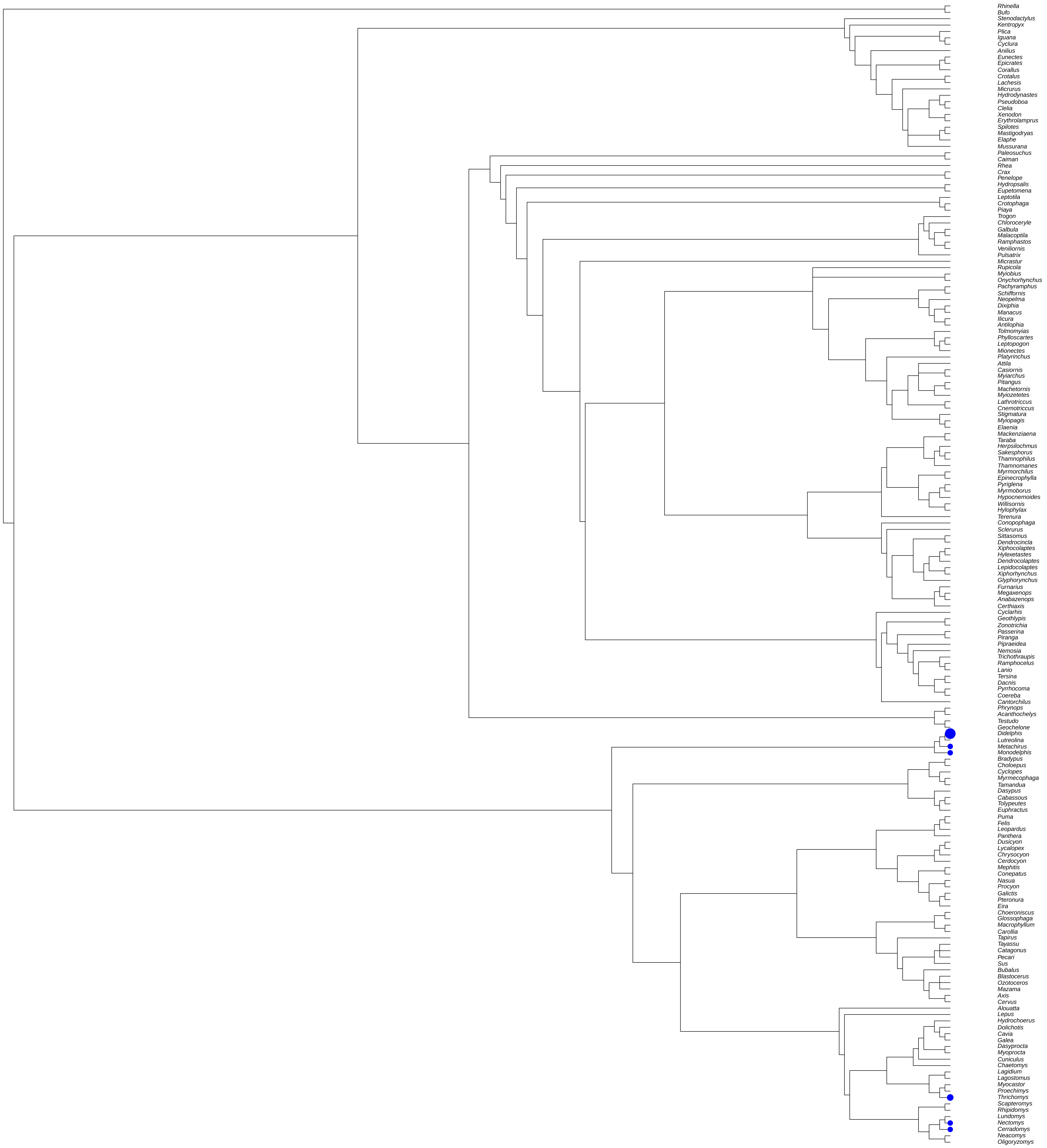

Amblyomma geayi adult

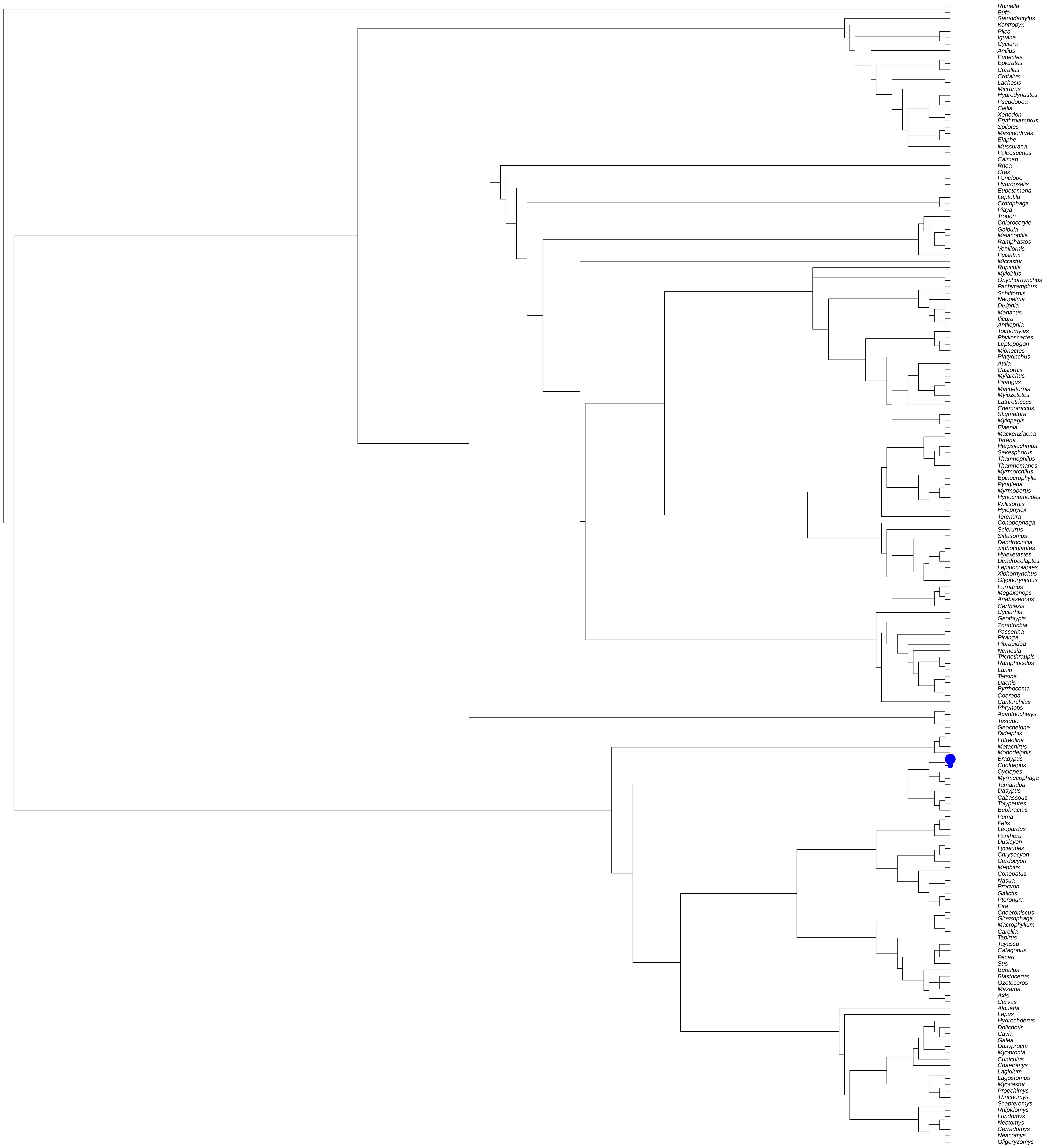

Amblyomma geayi larva

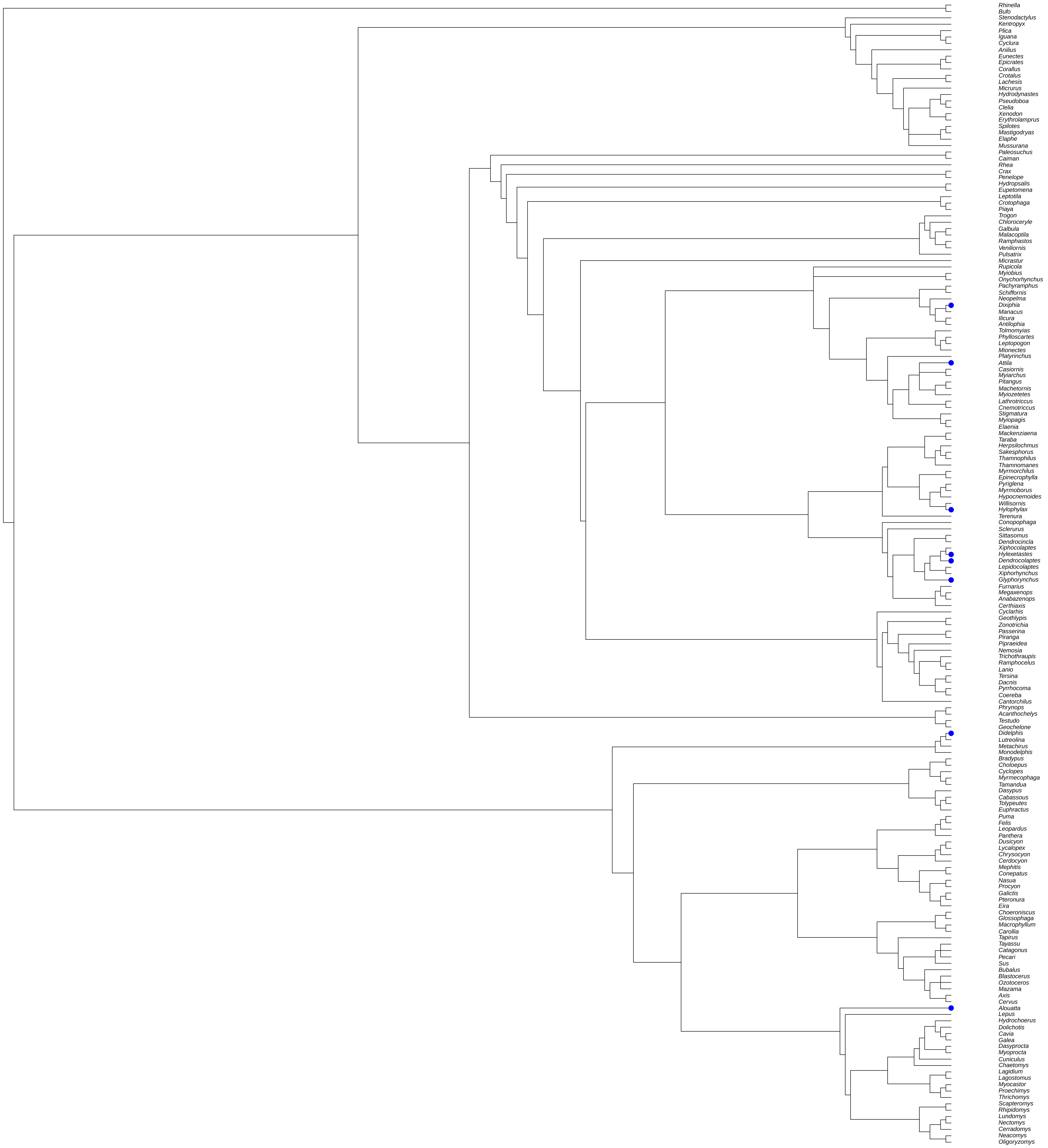

Amblyomma geayi nymph

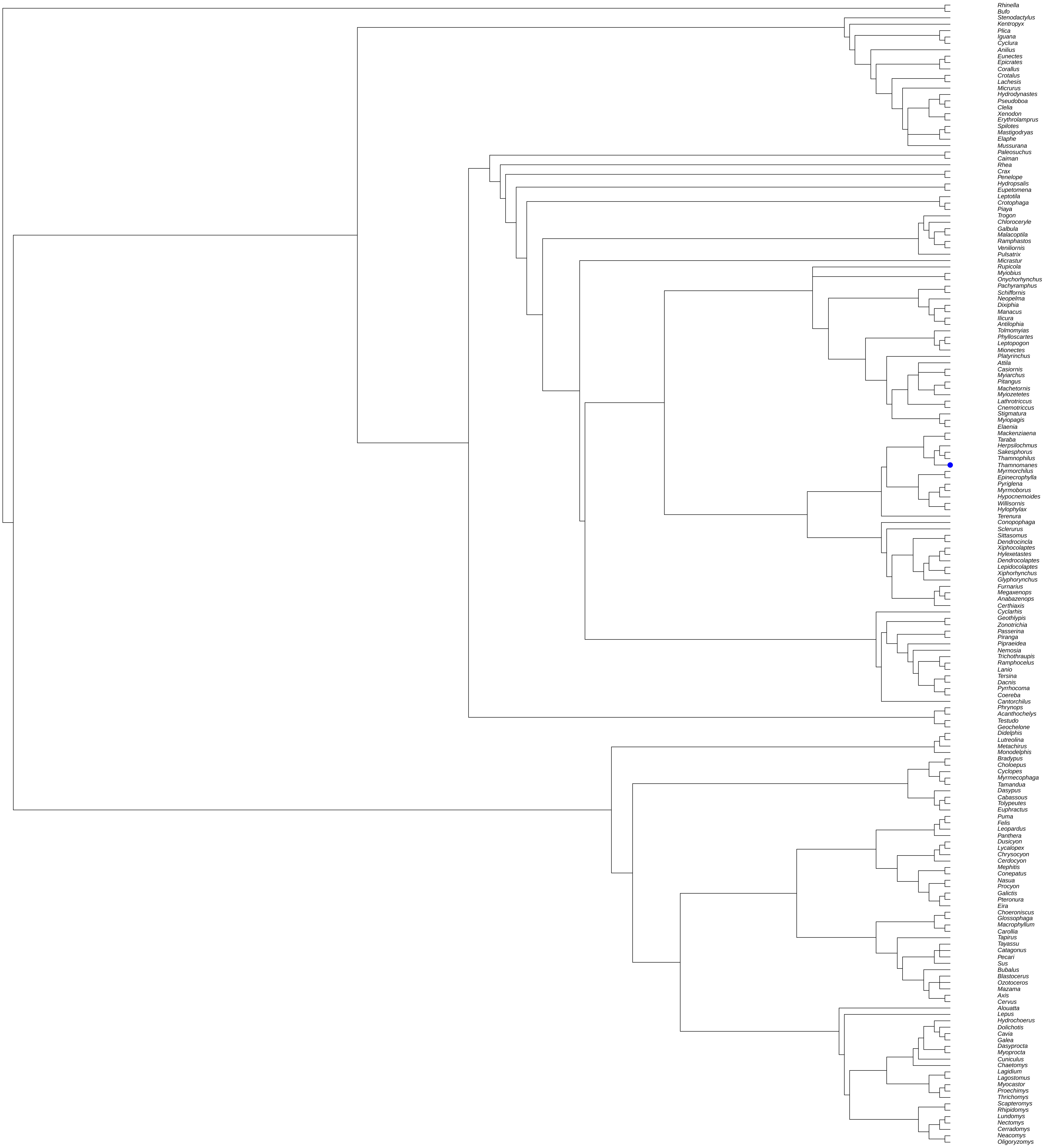

Amblyomma goeldii adult

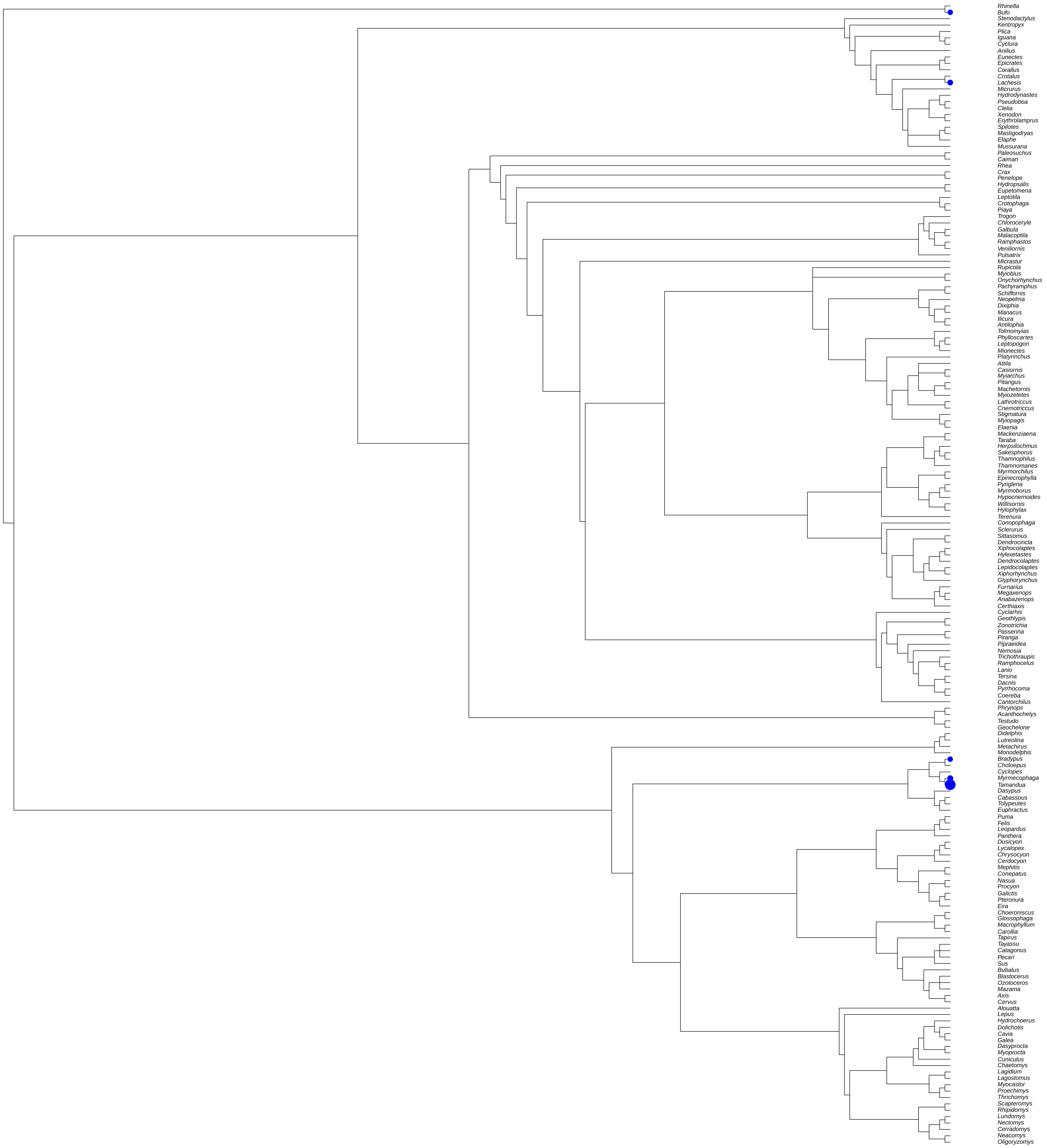

Amblyomma humerale adult

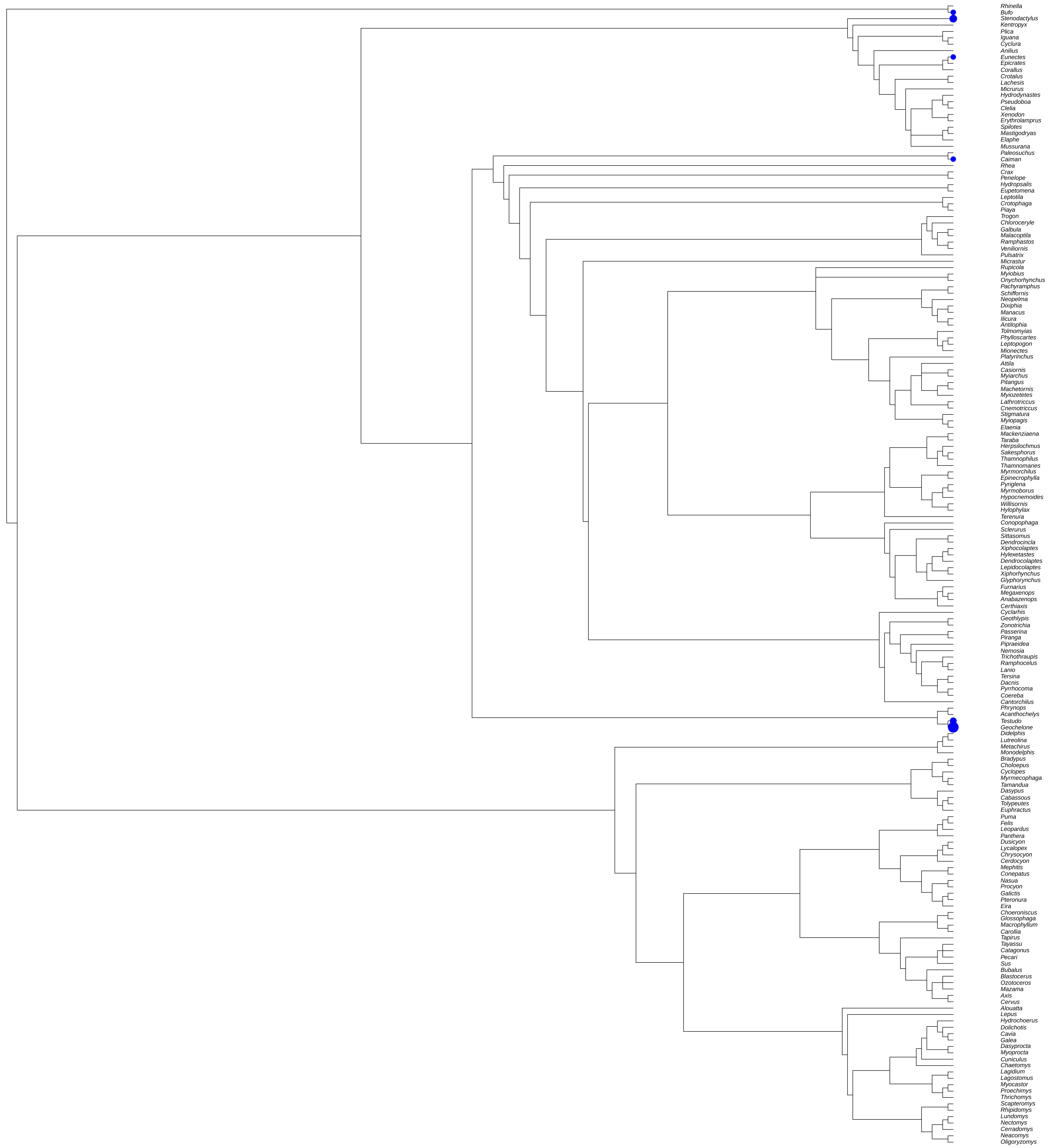

Amblyomma humerale larva

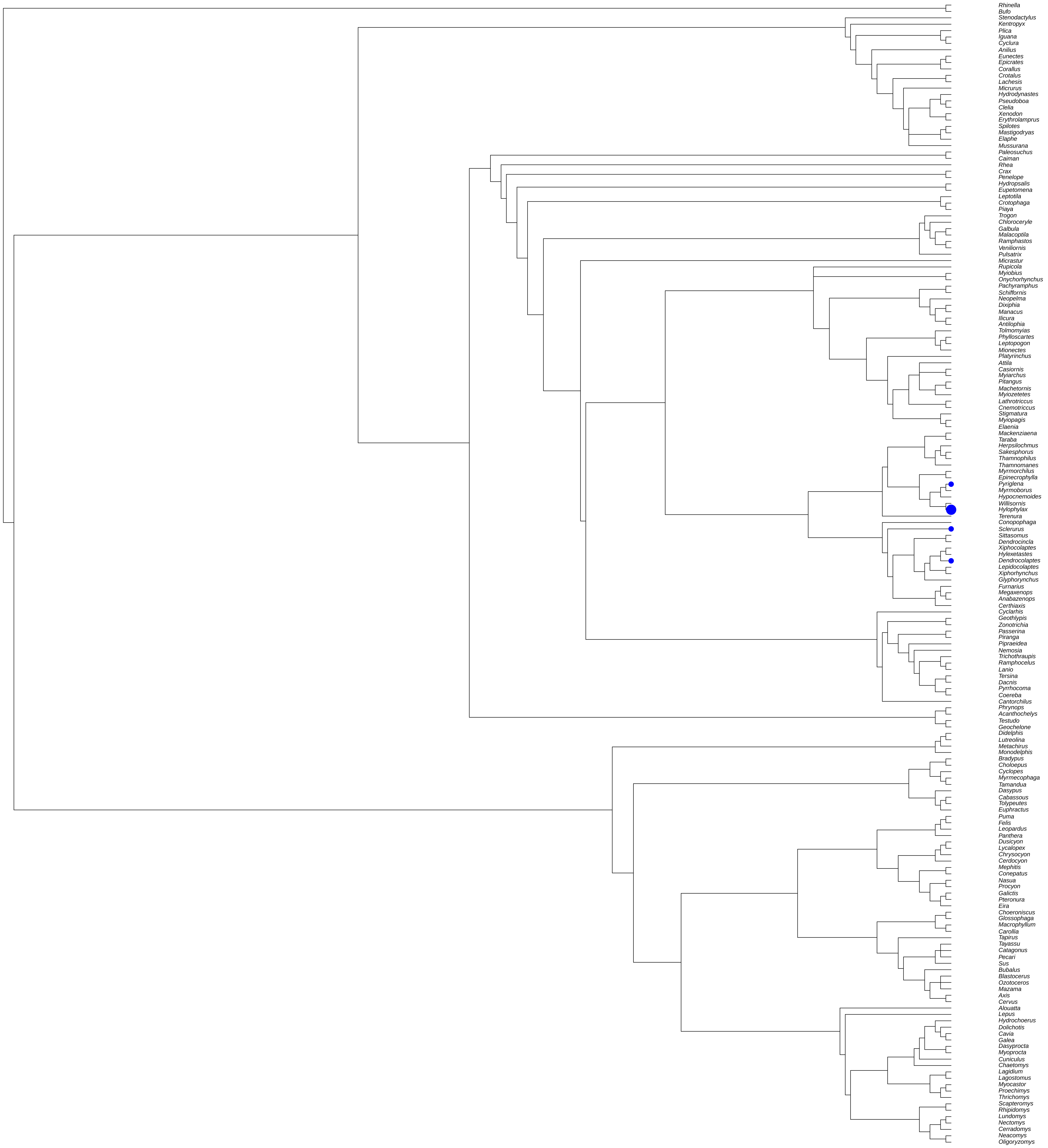

Amblyomma humerale nymph

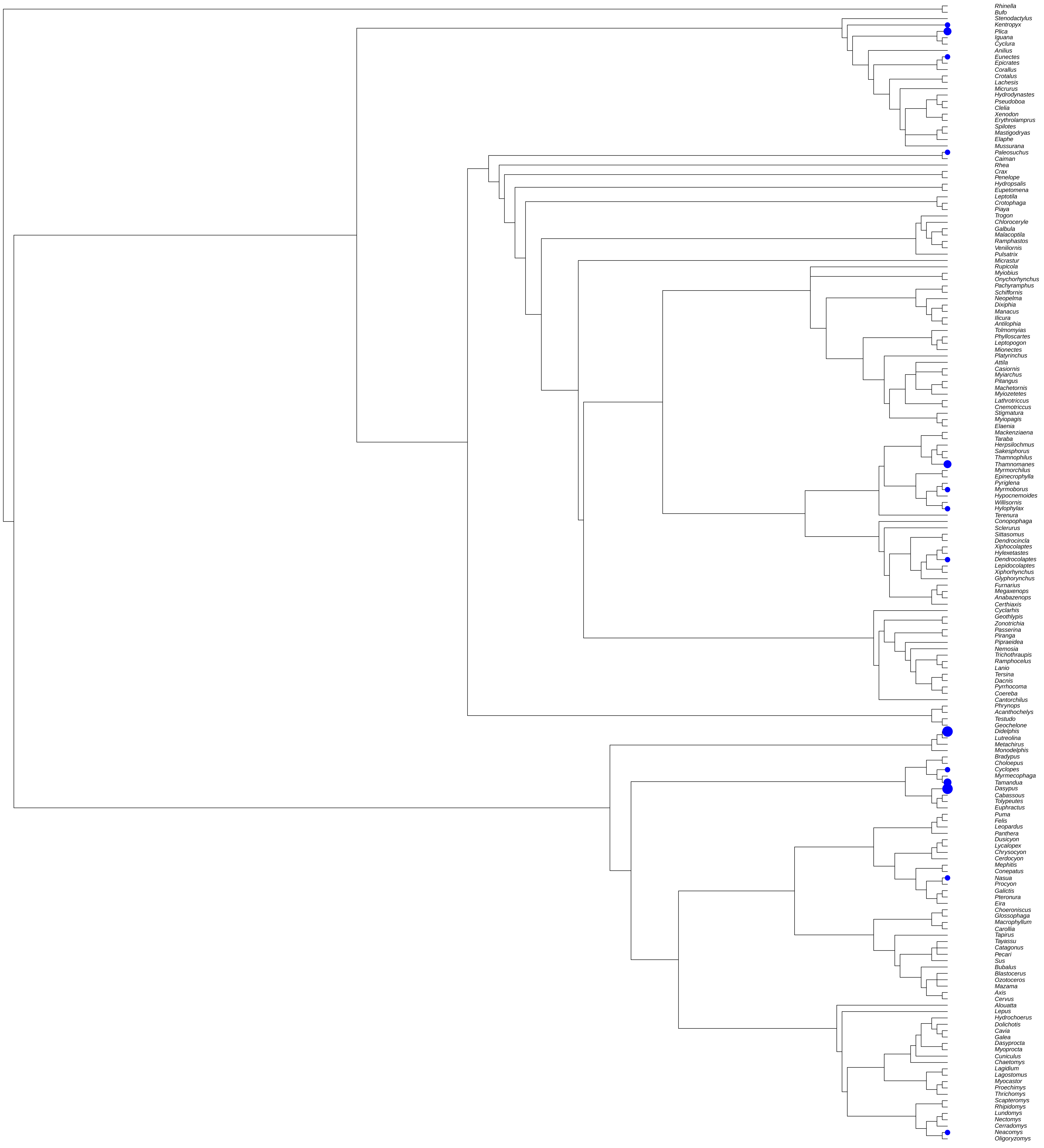

Amblyomma incisum adult

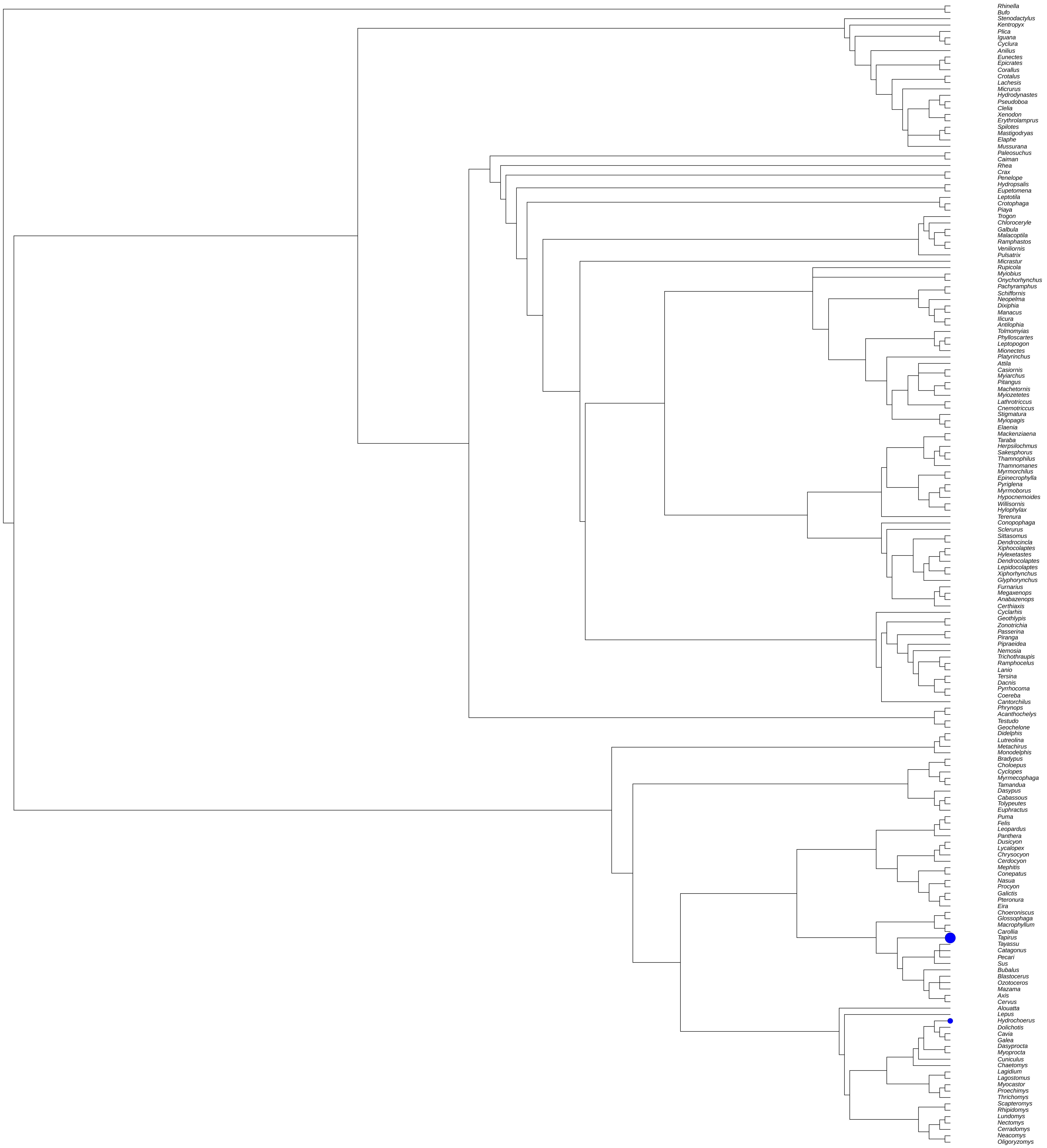

Amblyomma incisum nymph

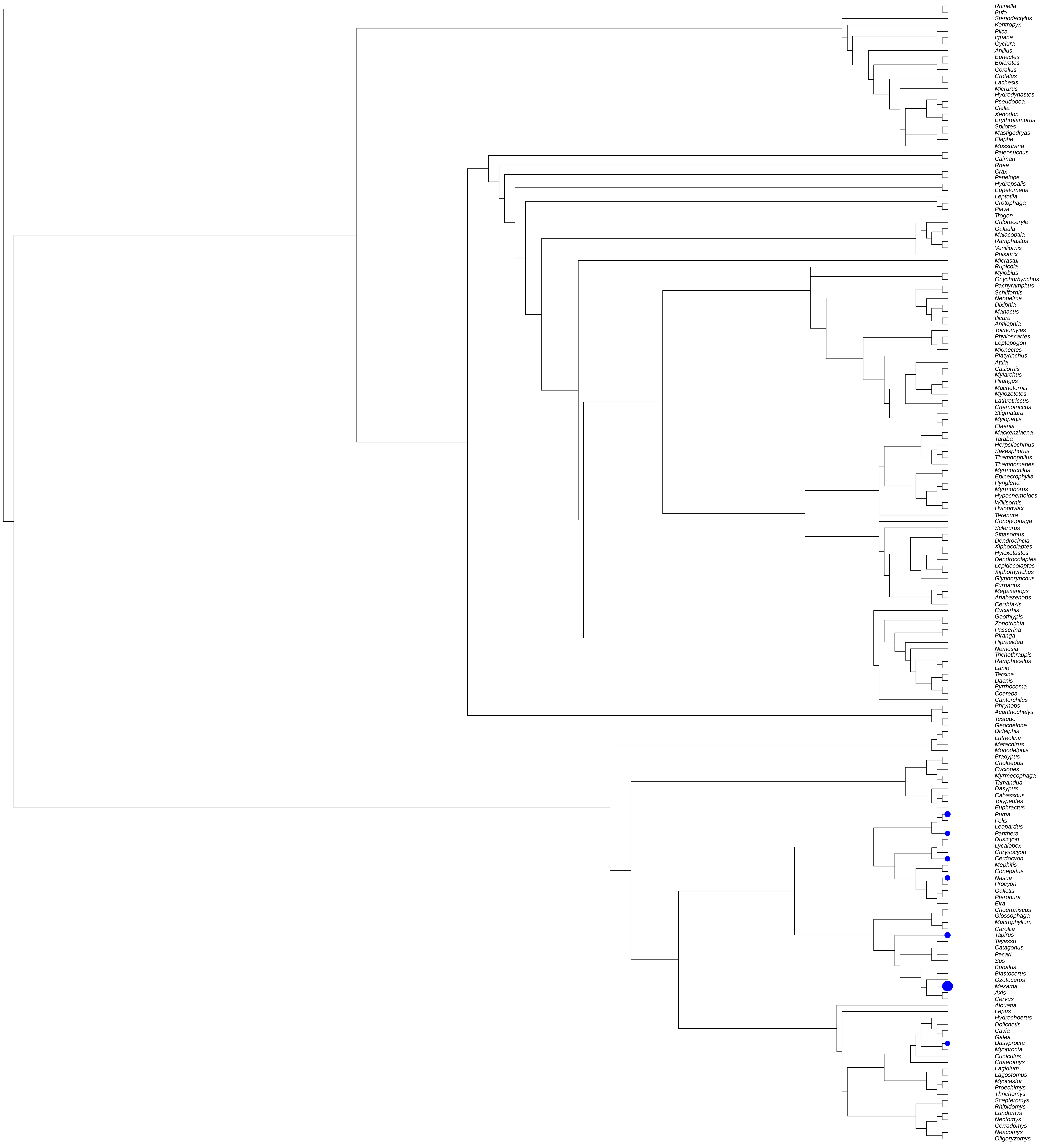

Amblyomma longirostre adult

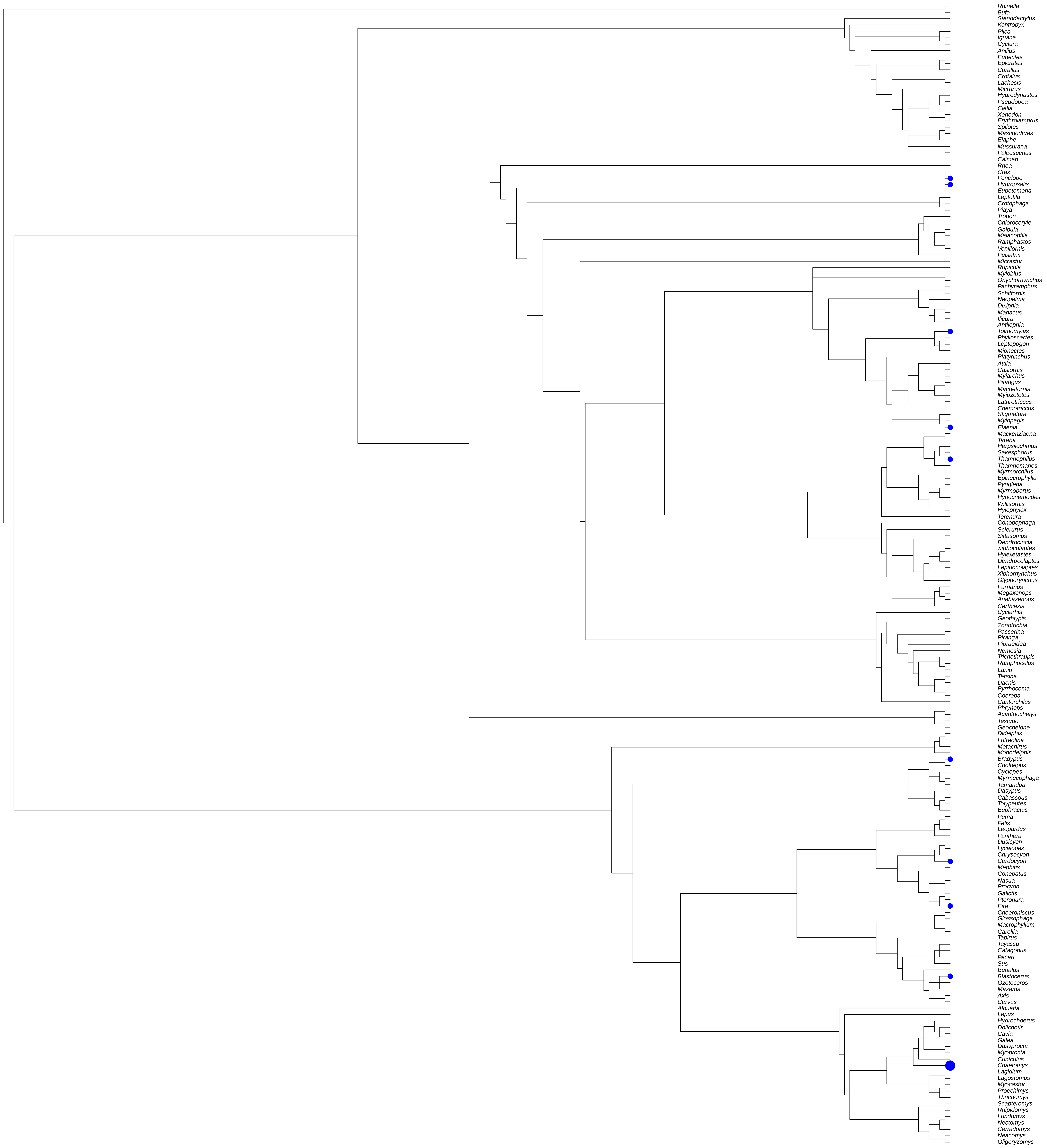

Amblyomma longirostre larva

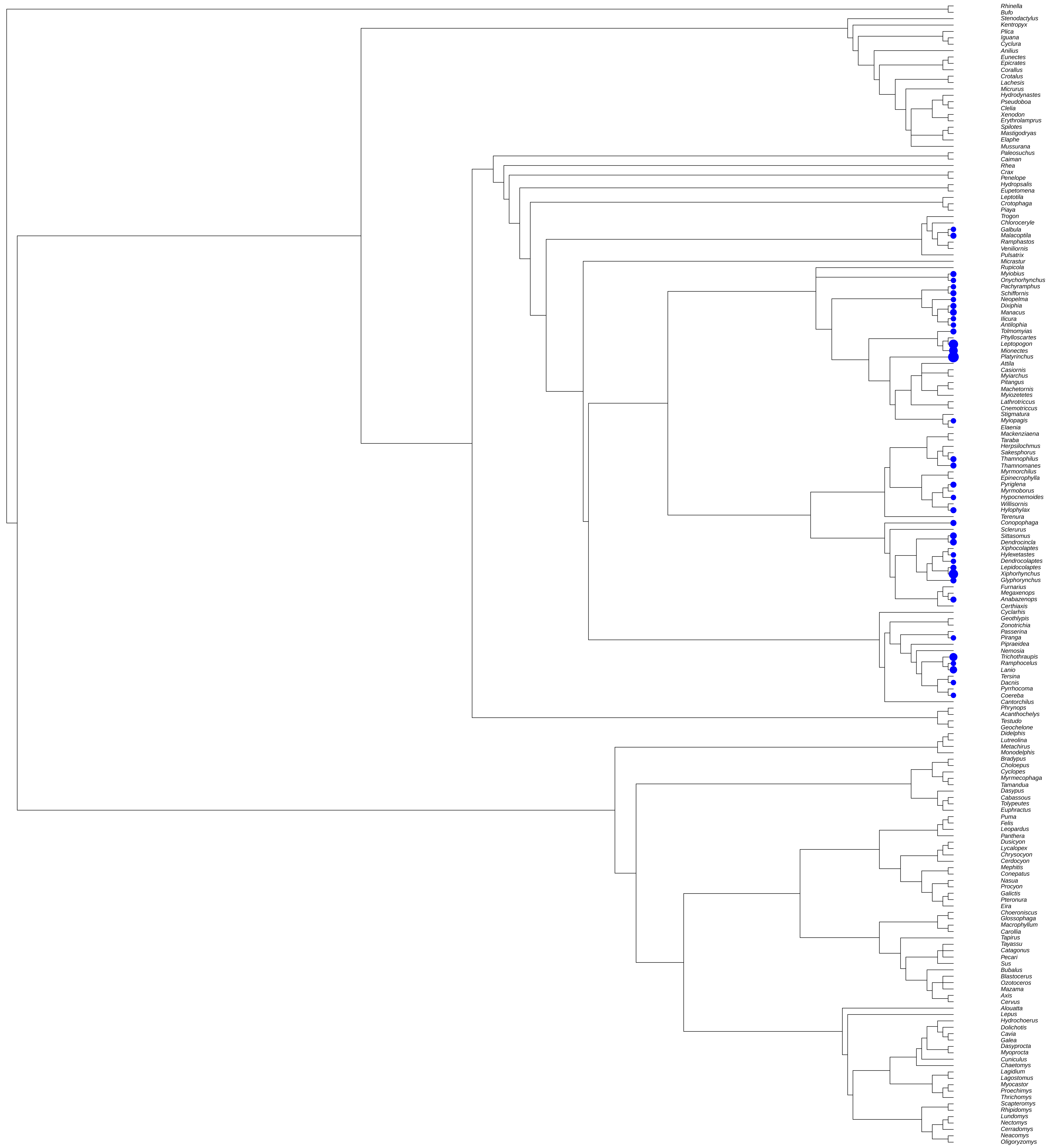

Amblyomma longirostre nymph

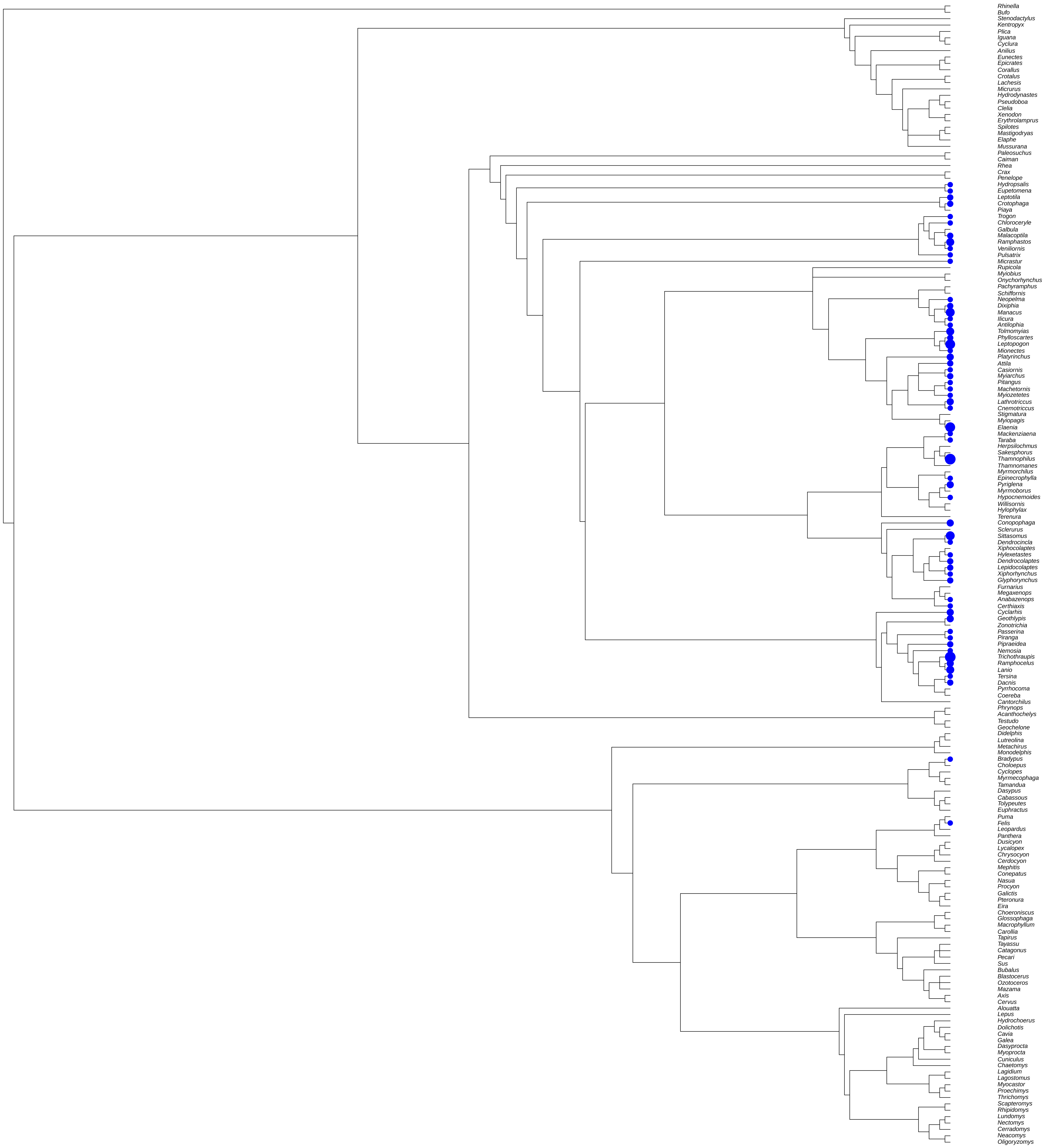

Amblyomma mixtum adult

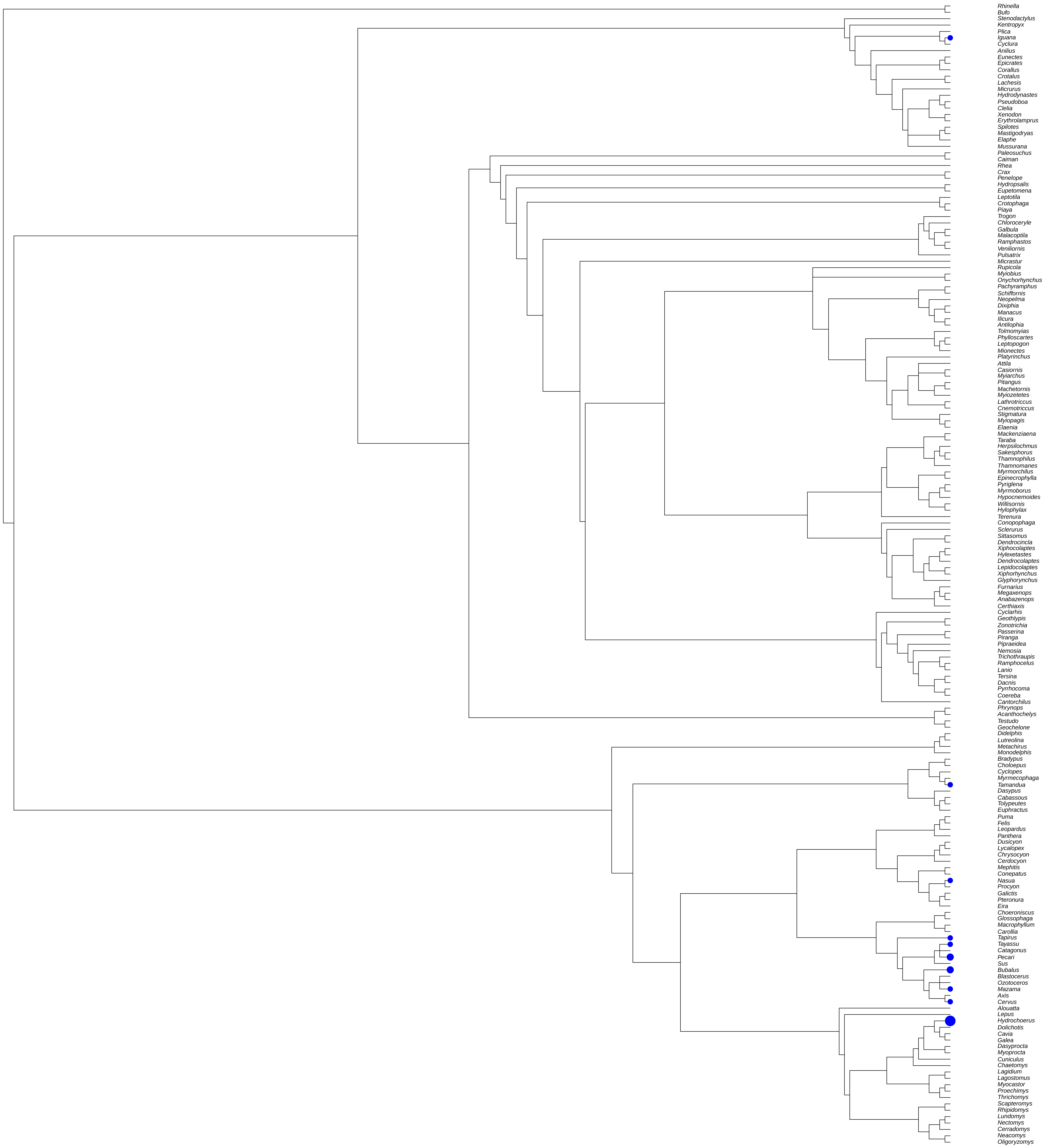

Amblyomma mixtum larva

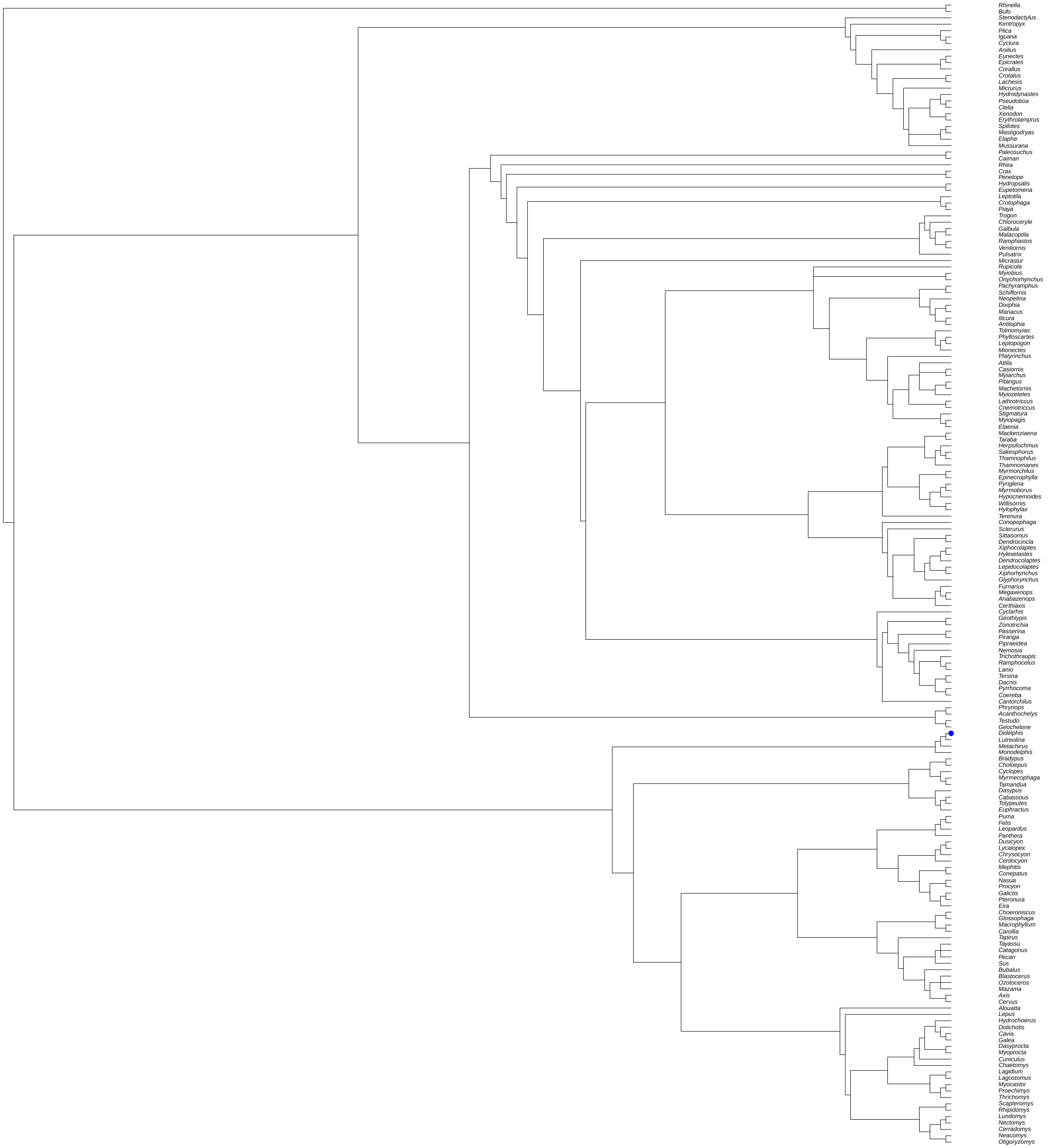

Amblyomma naponense adult

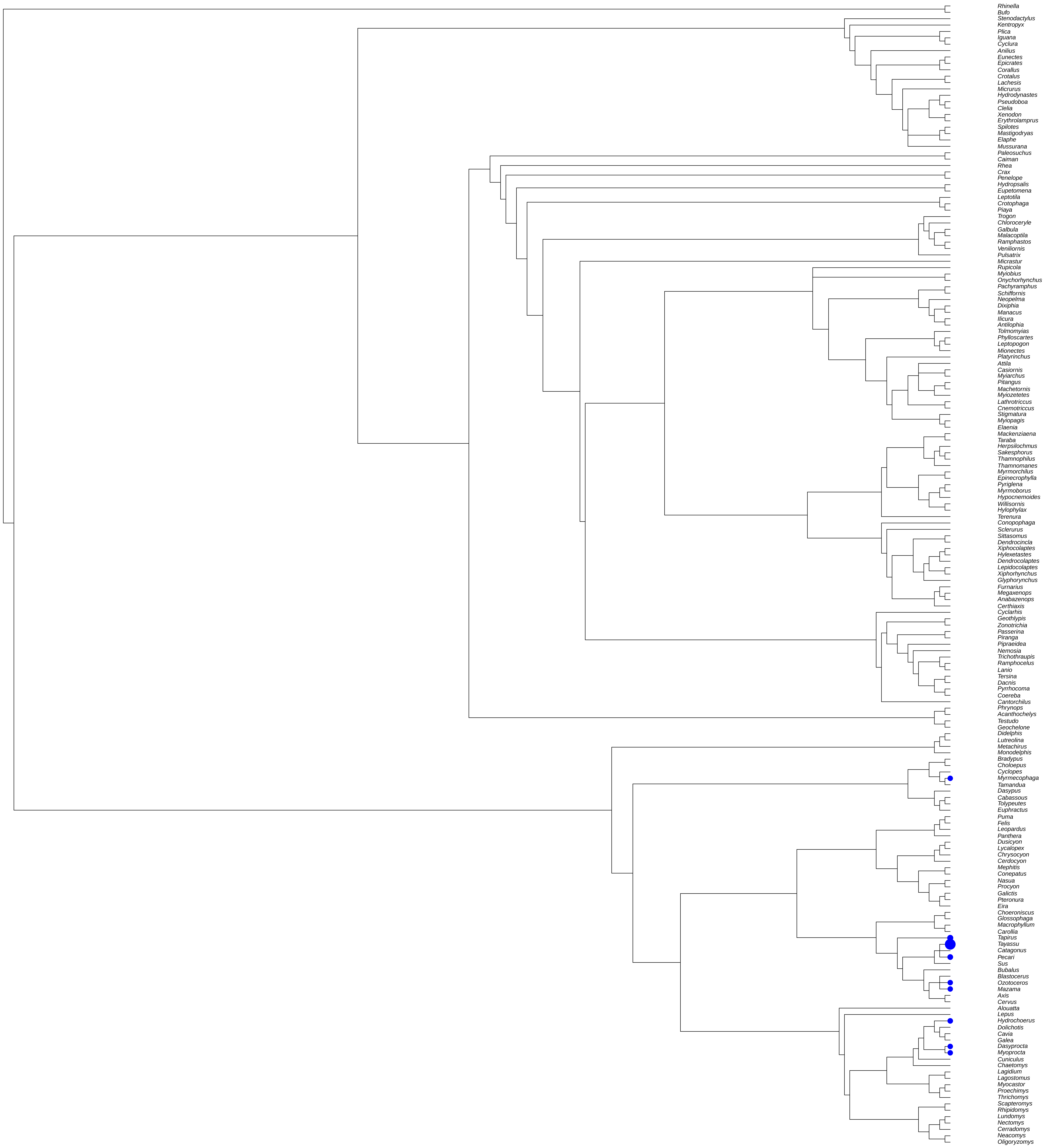

Amblyomma naponense larva

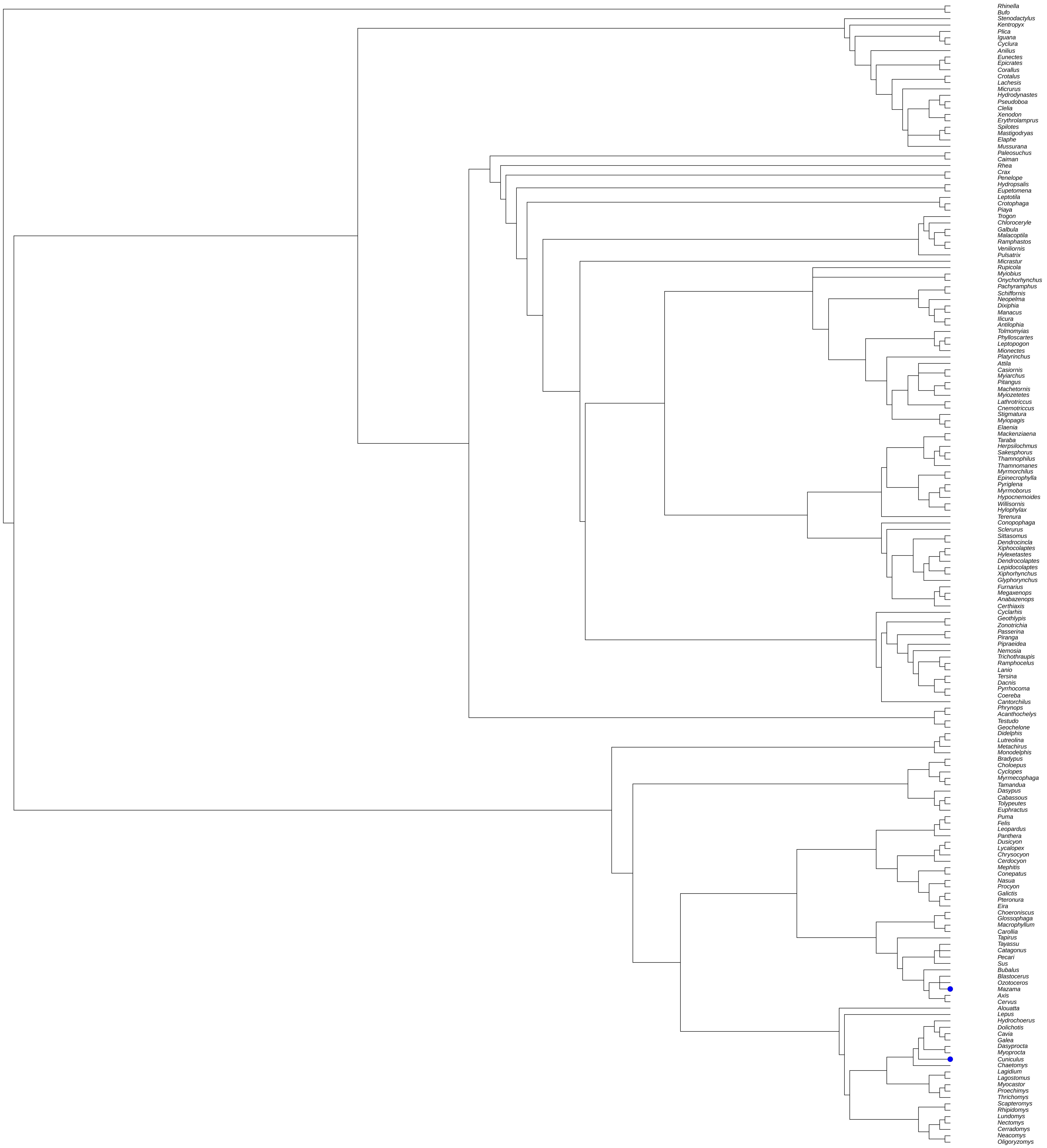

Amblyomma naponense nymph

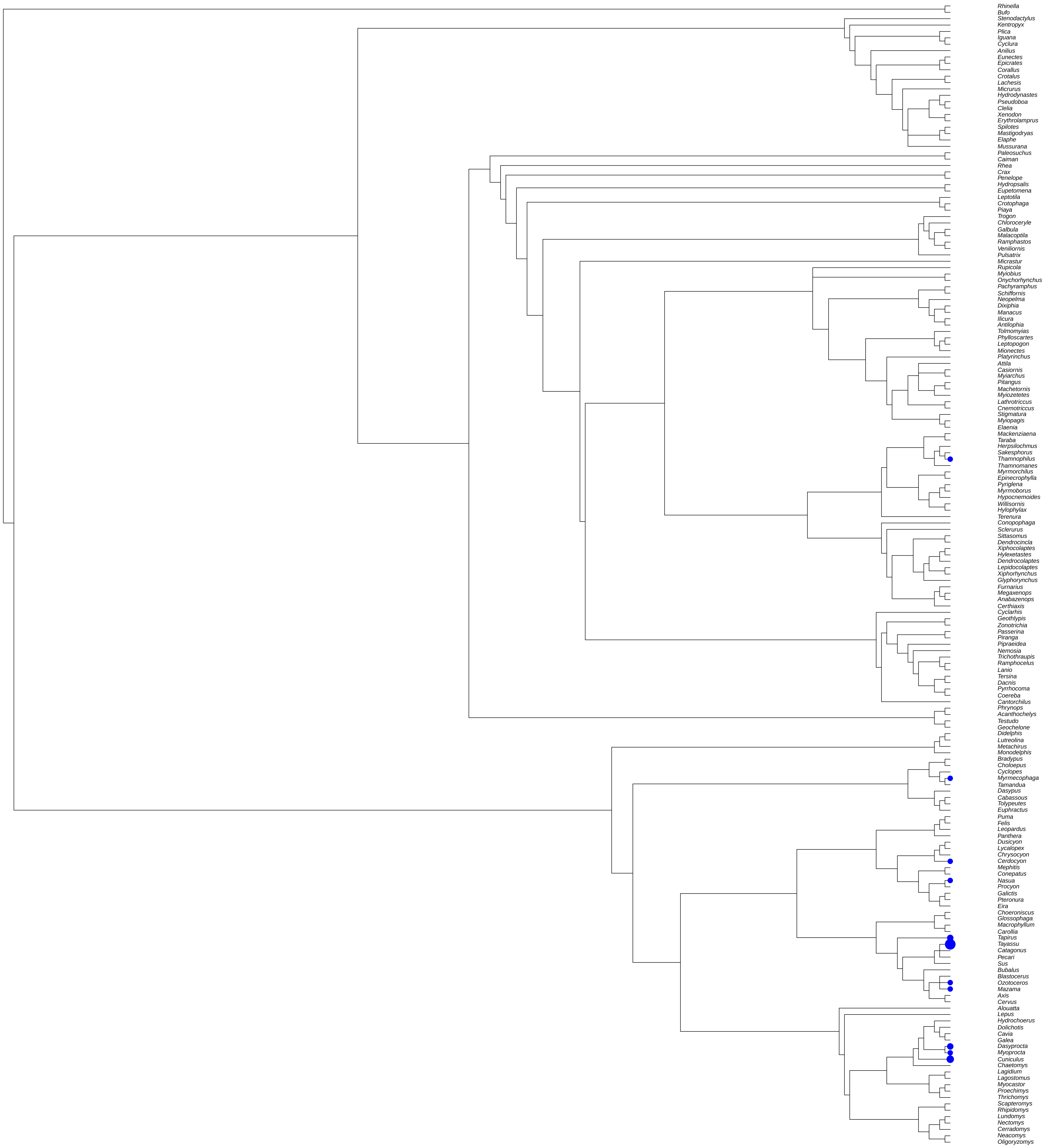

Amblyomma neumanni adult

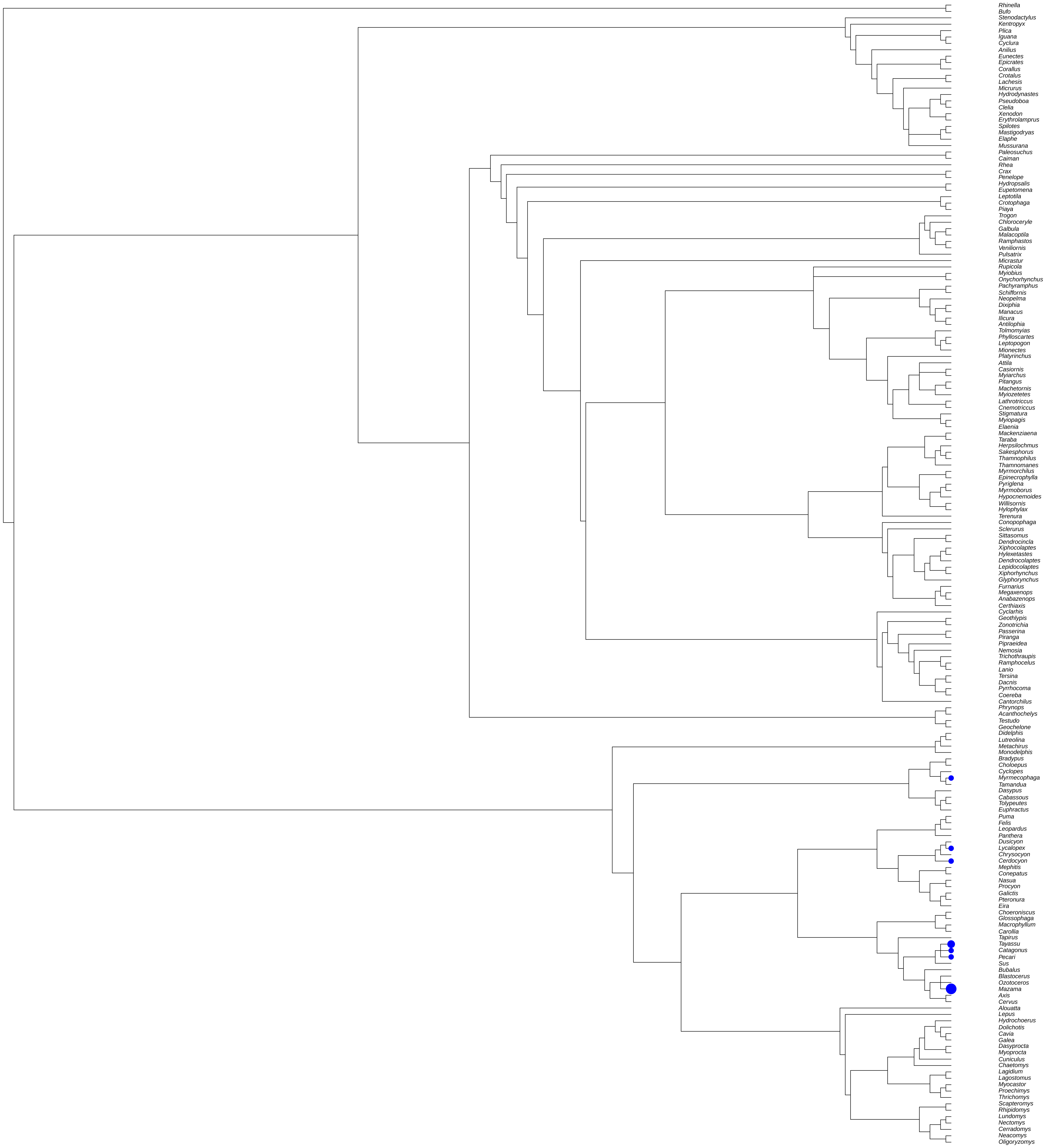

Amblyomma neumanni larva

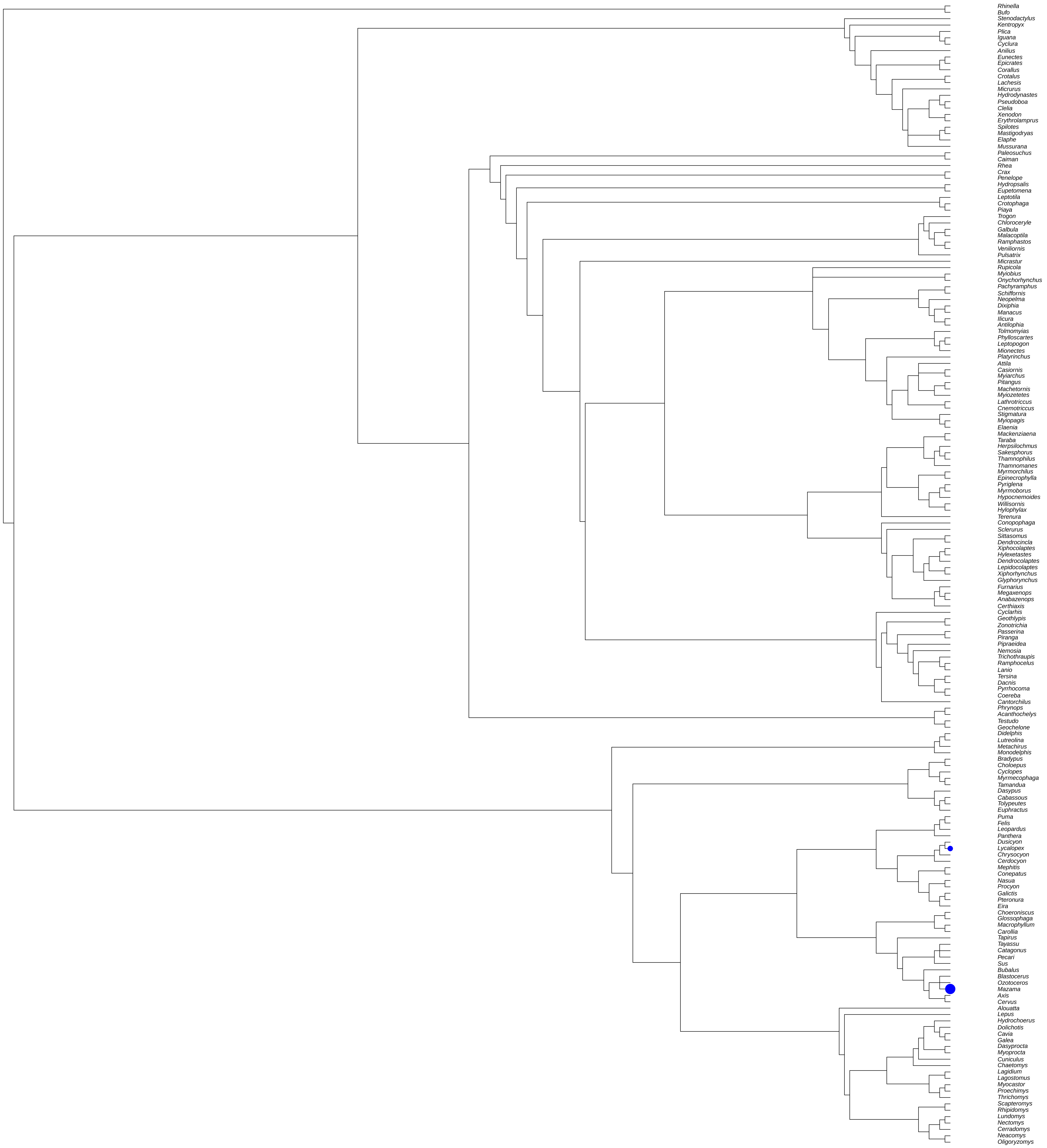

Amblyomma neumanni nymph

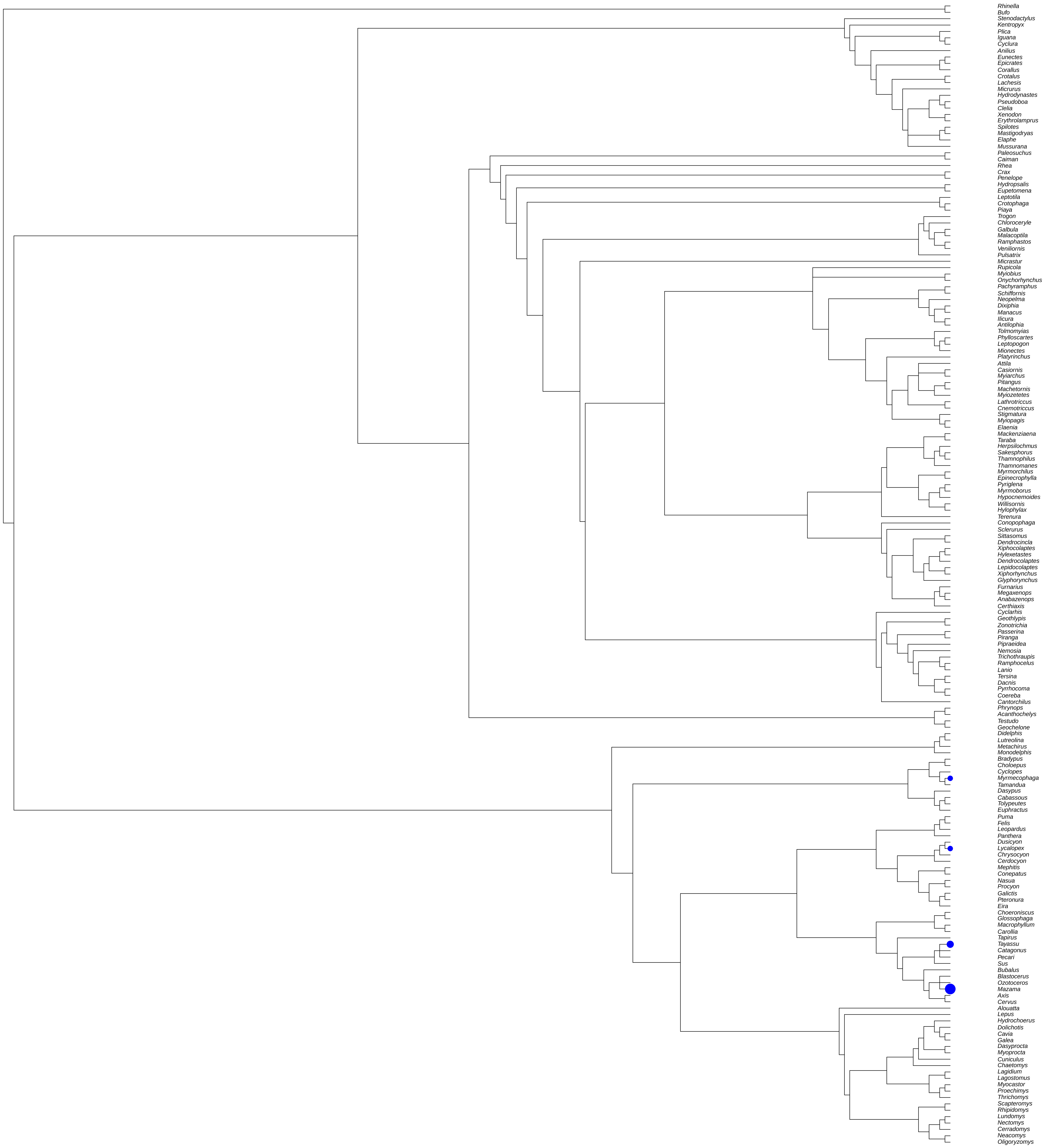

Amblyomma nodosum adult

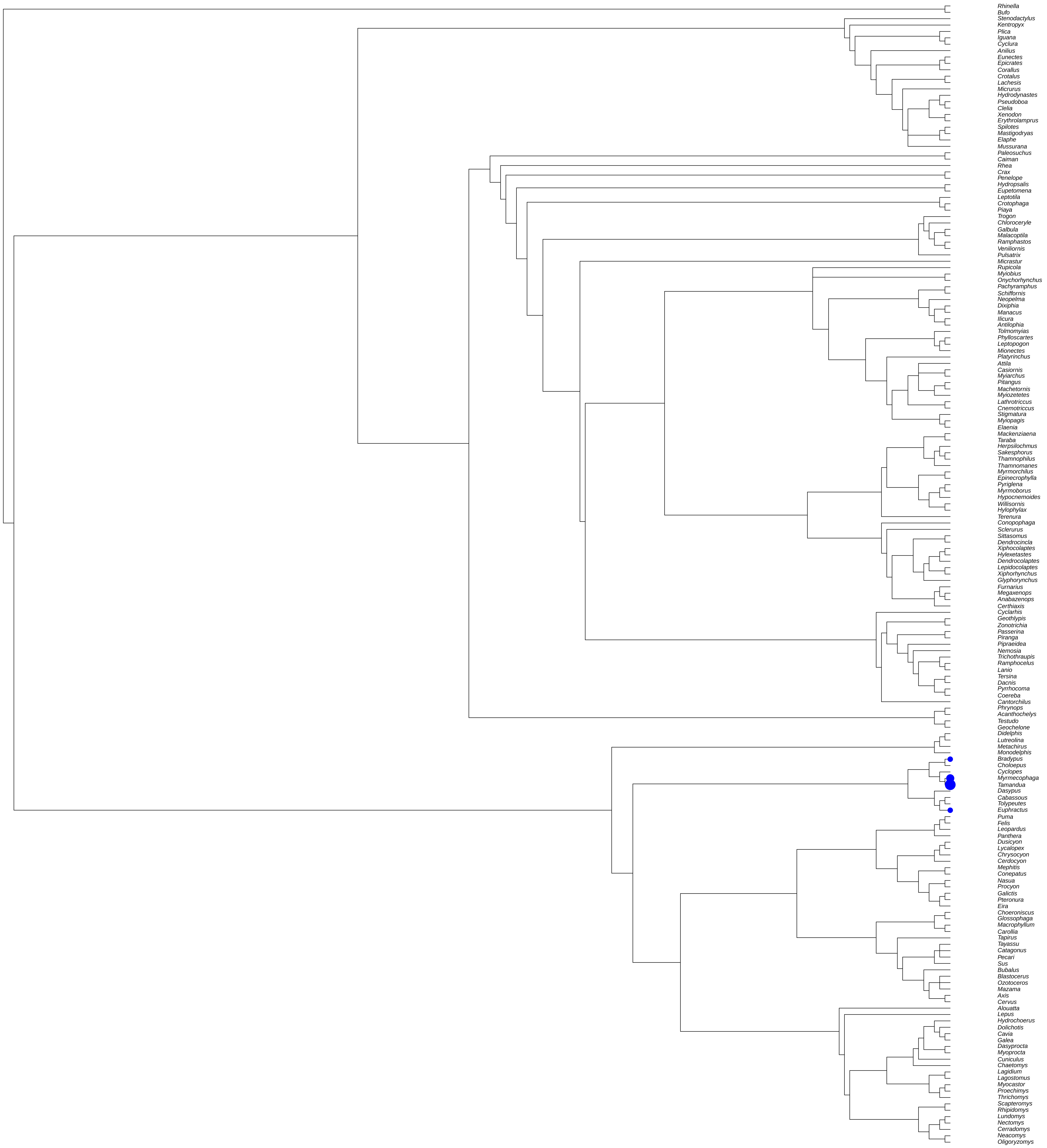

Amblyomma nodosum larva

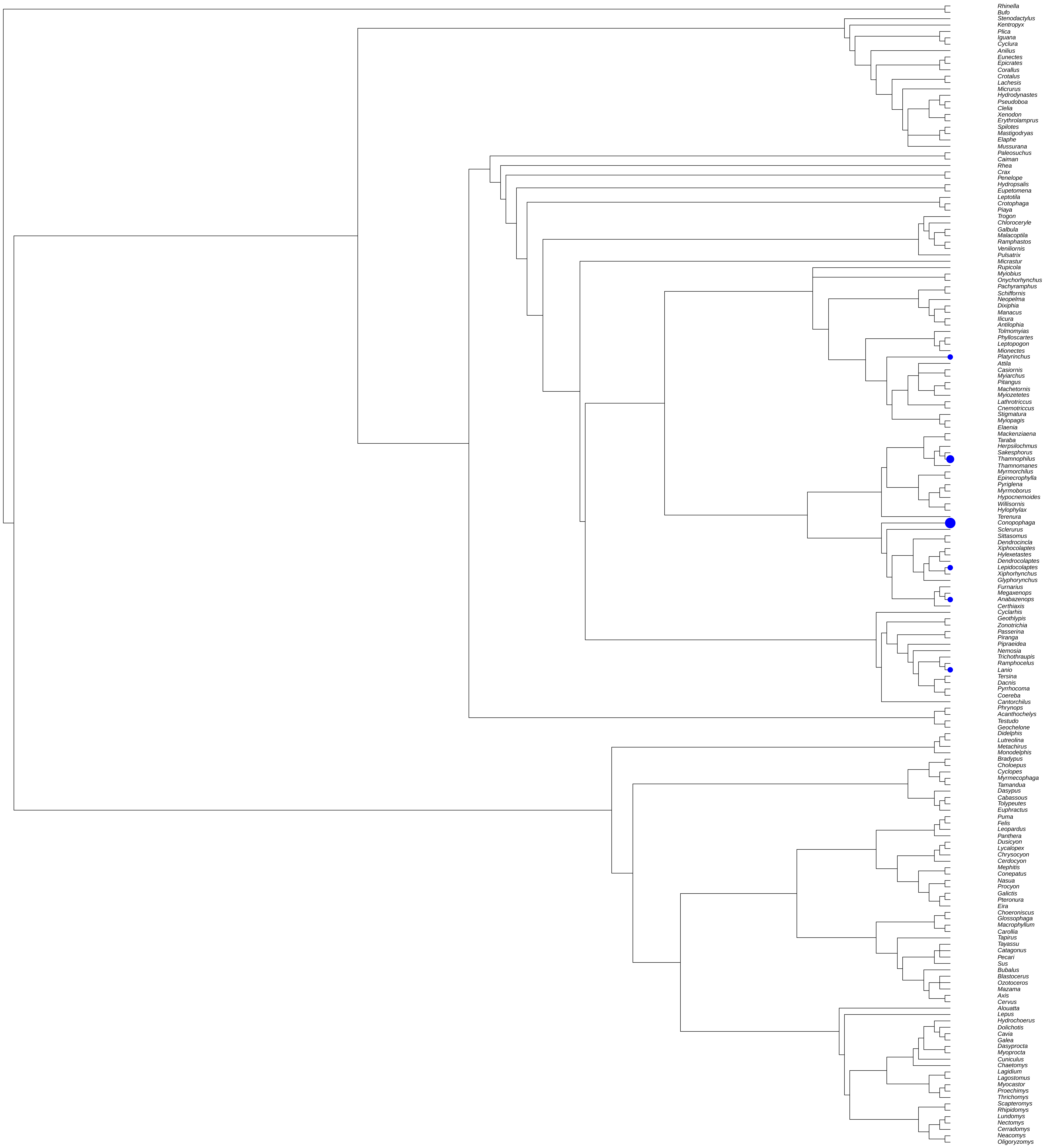

Amblyomma nodosum nymph

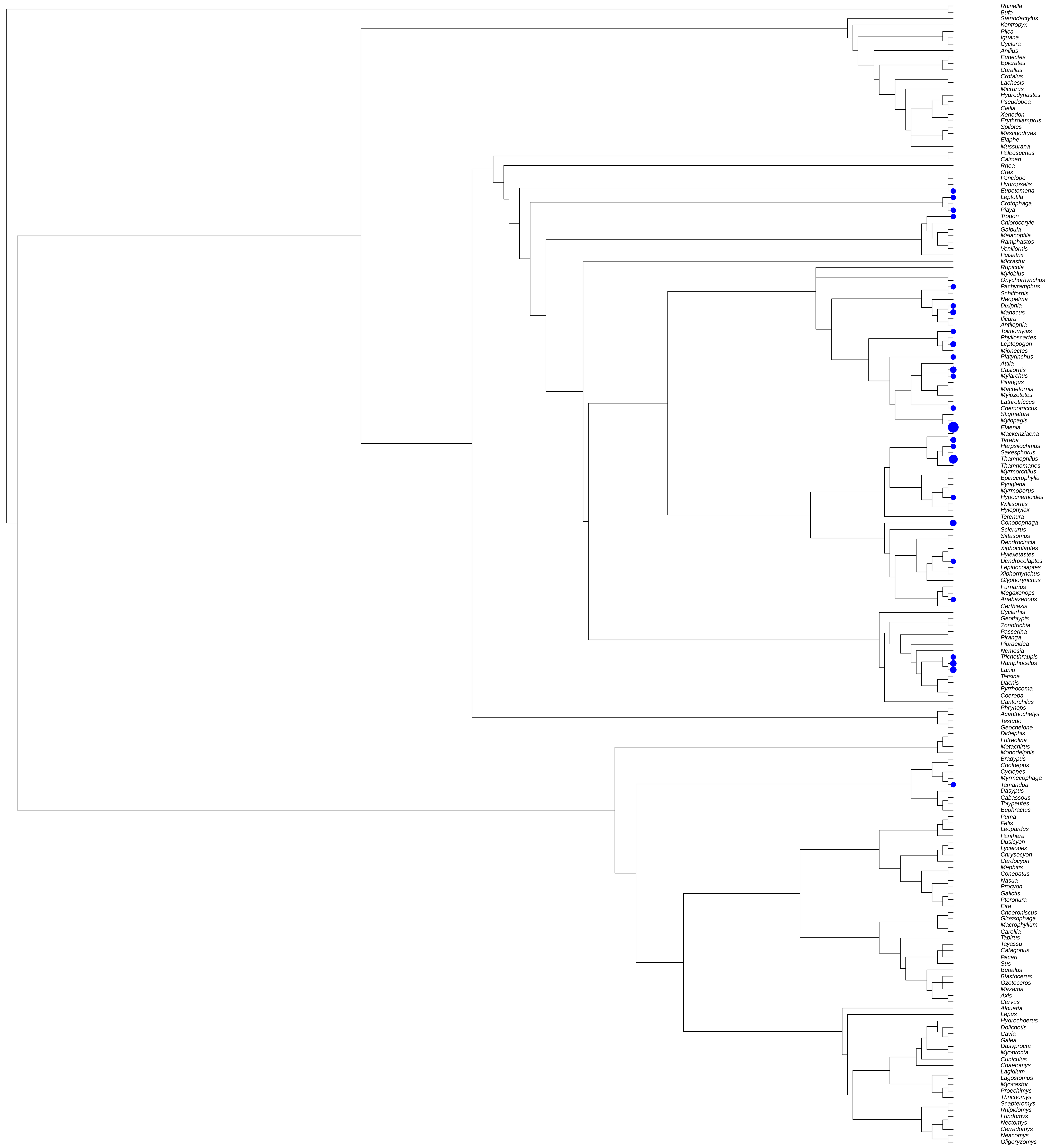

Amblyomma oblongoguttatum adult

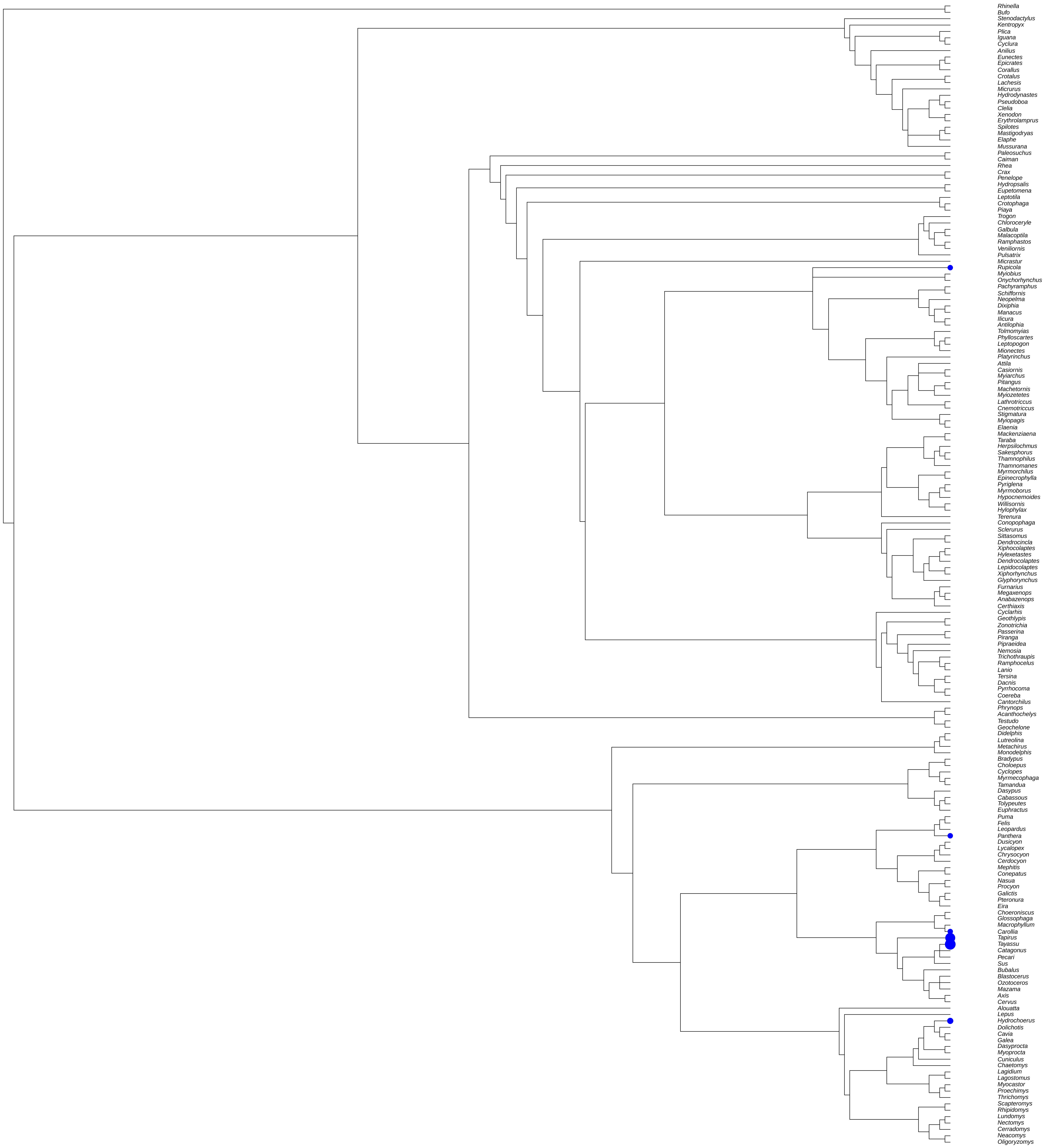

Amblyomma oblongoguttatum nymph

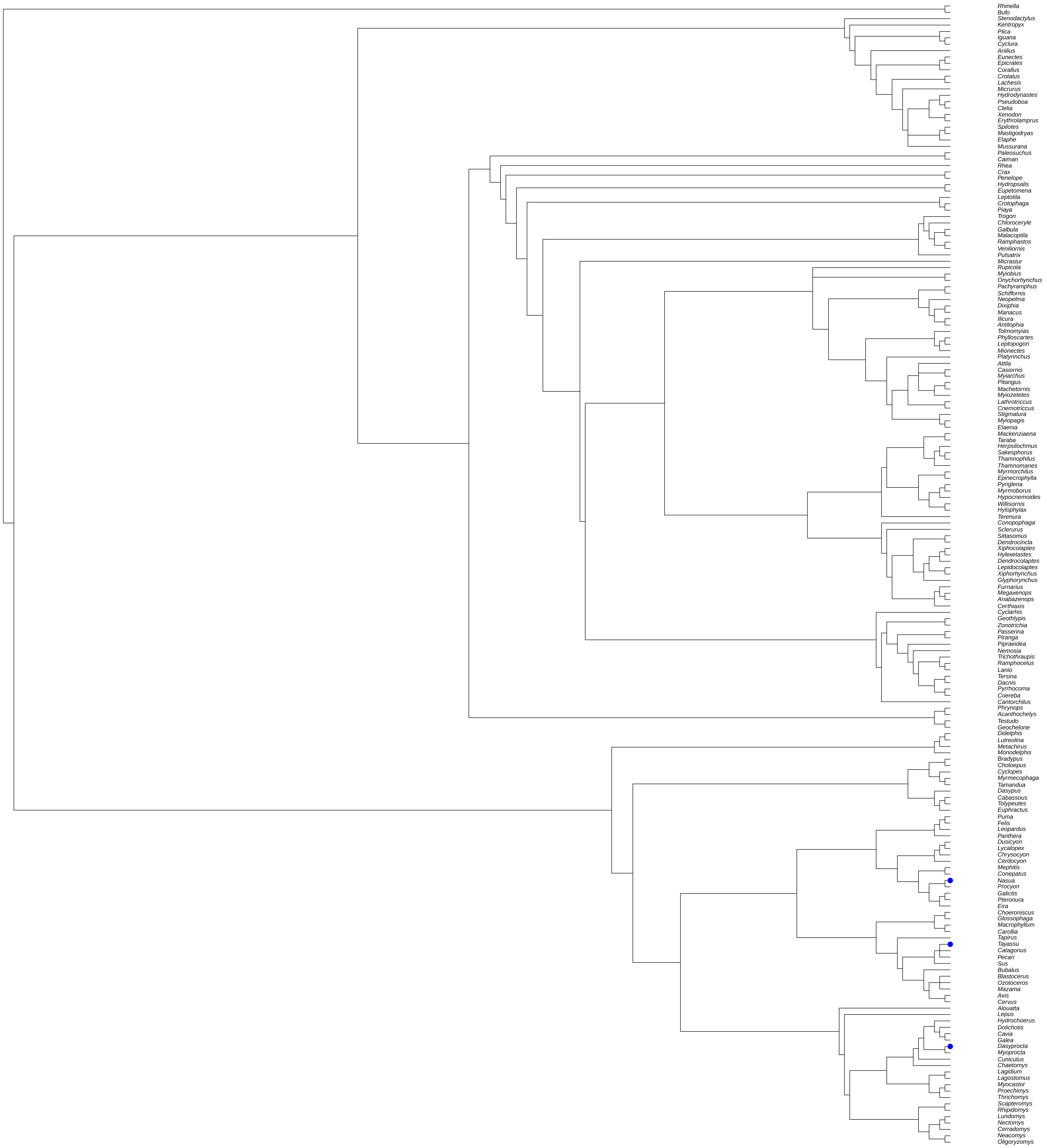

Amblyomma ovale adult

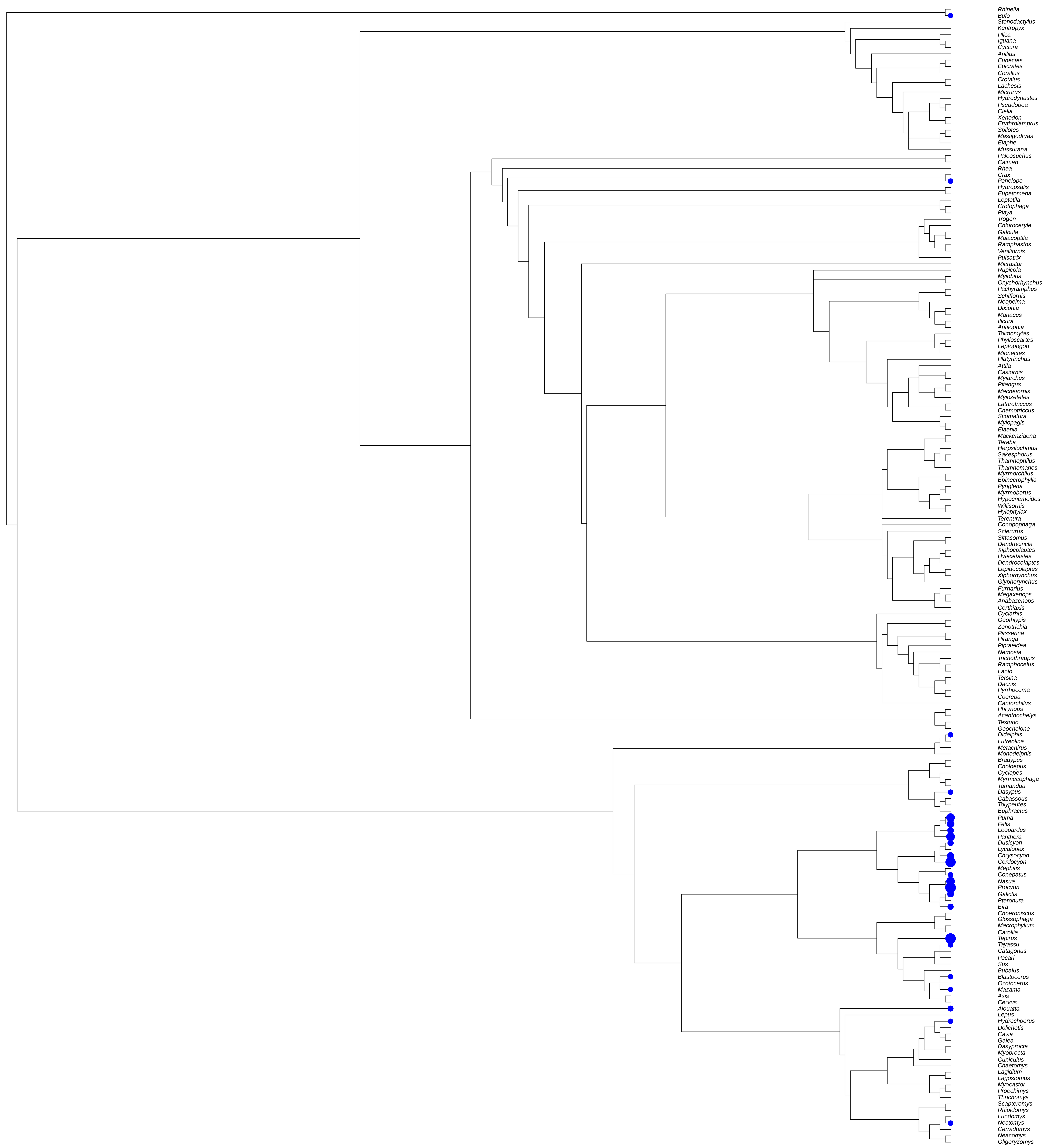

Amblyomma ovale larva

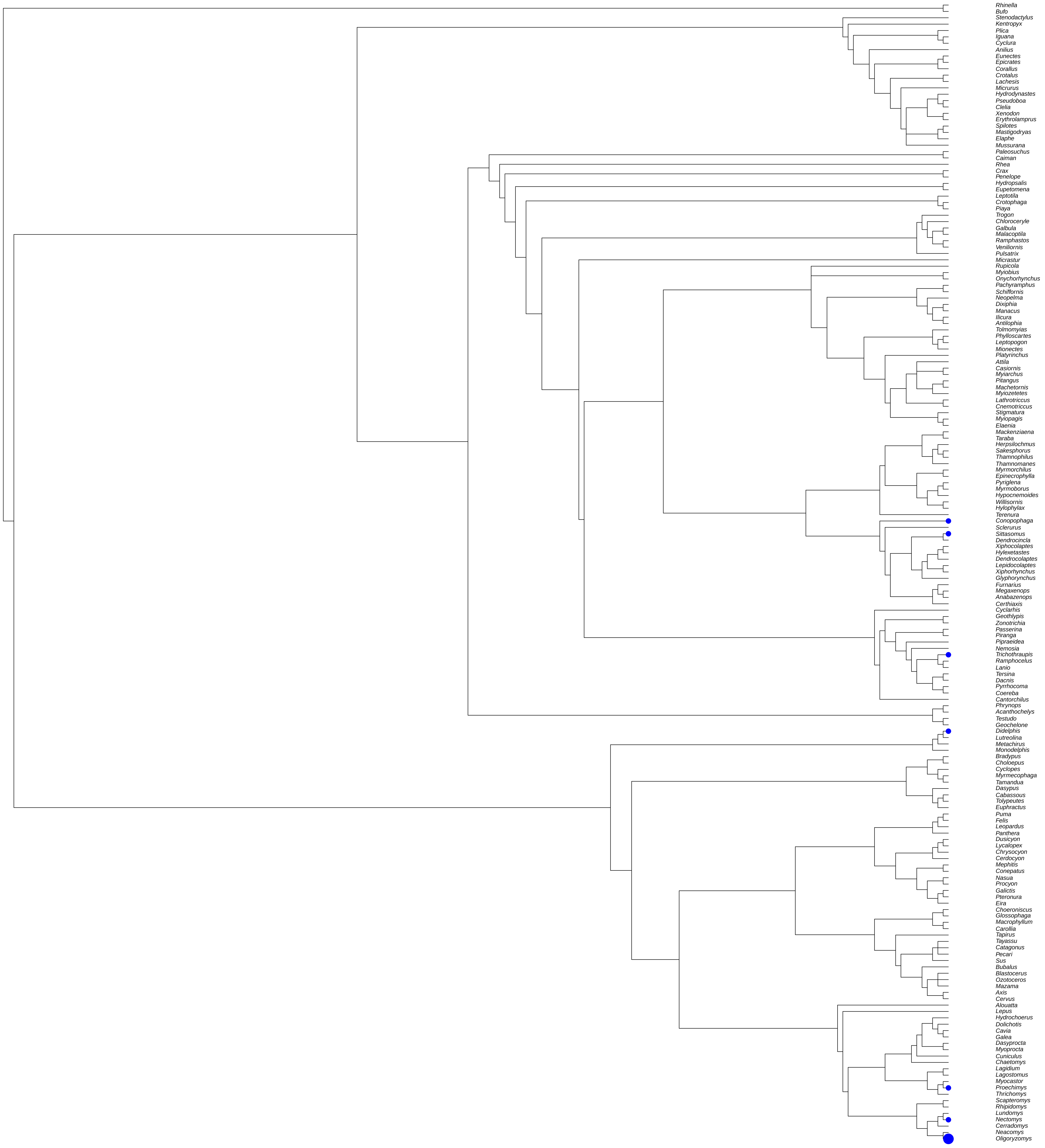

Amblyomma ovale nymph

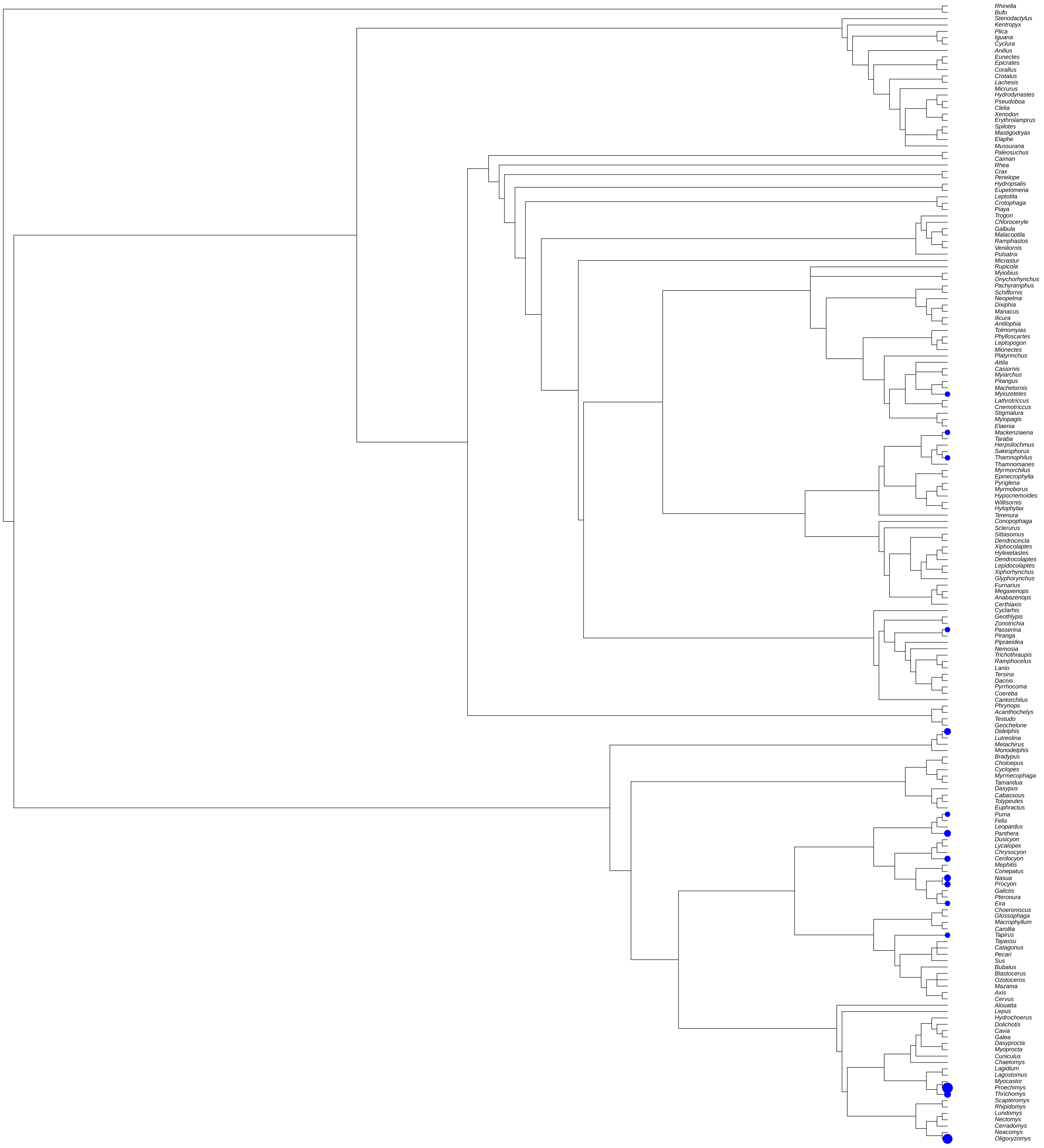

Amblyomma pacae adult

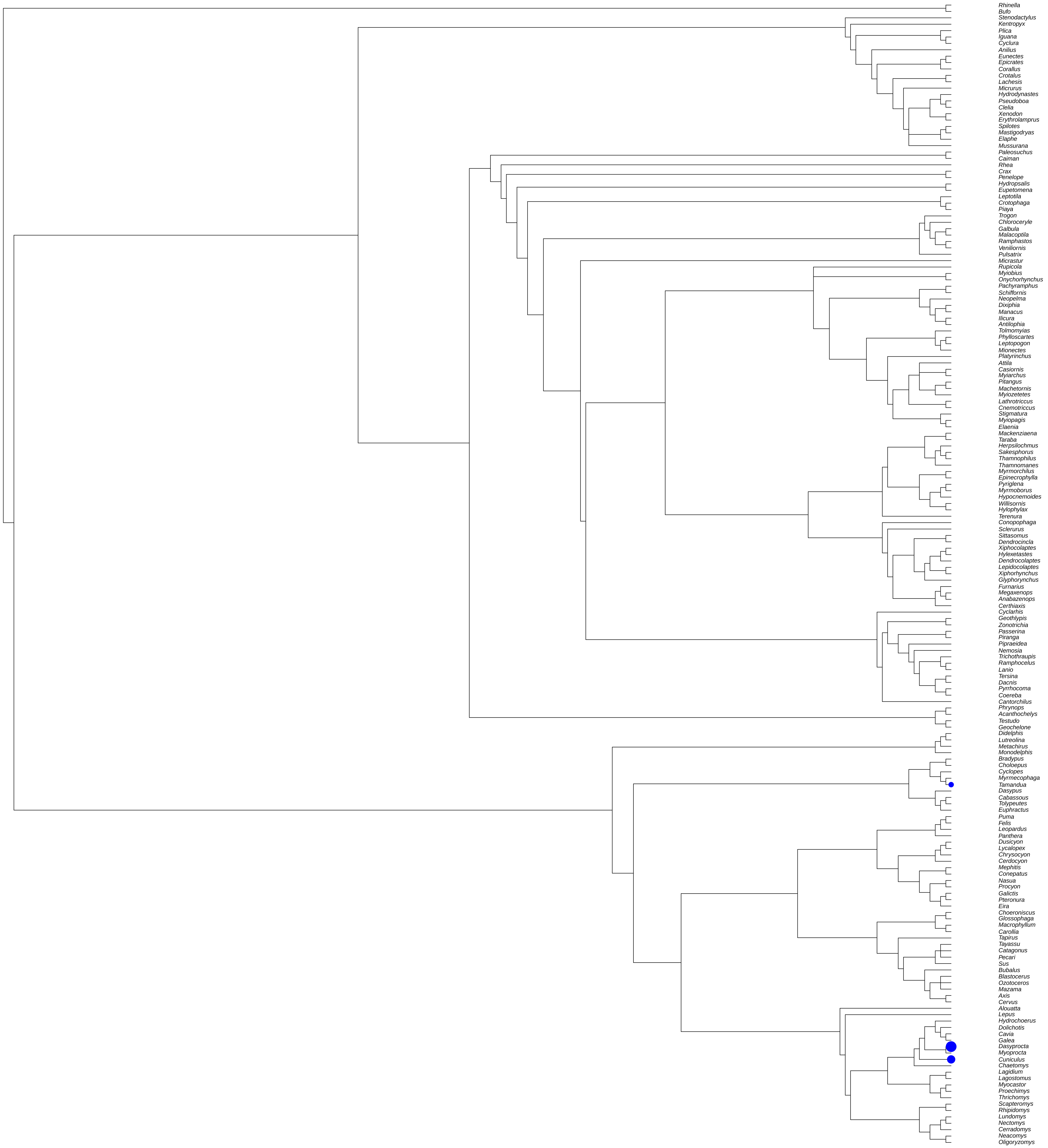

Amblyomma pacae nymph

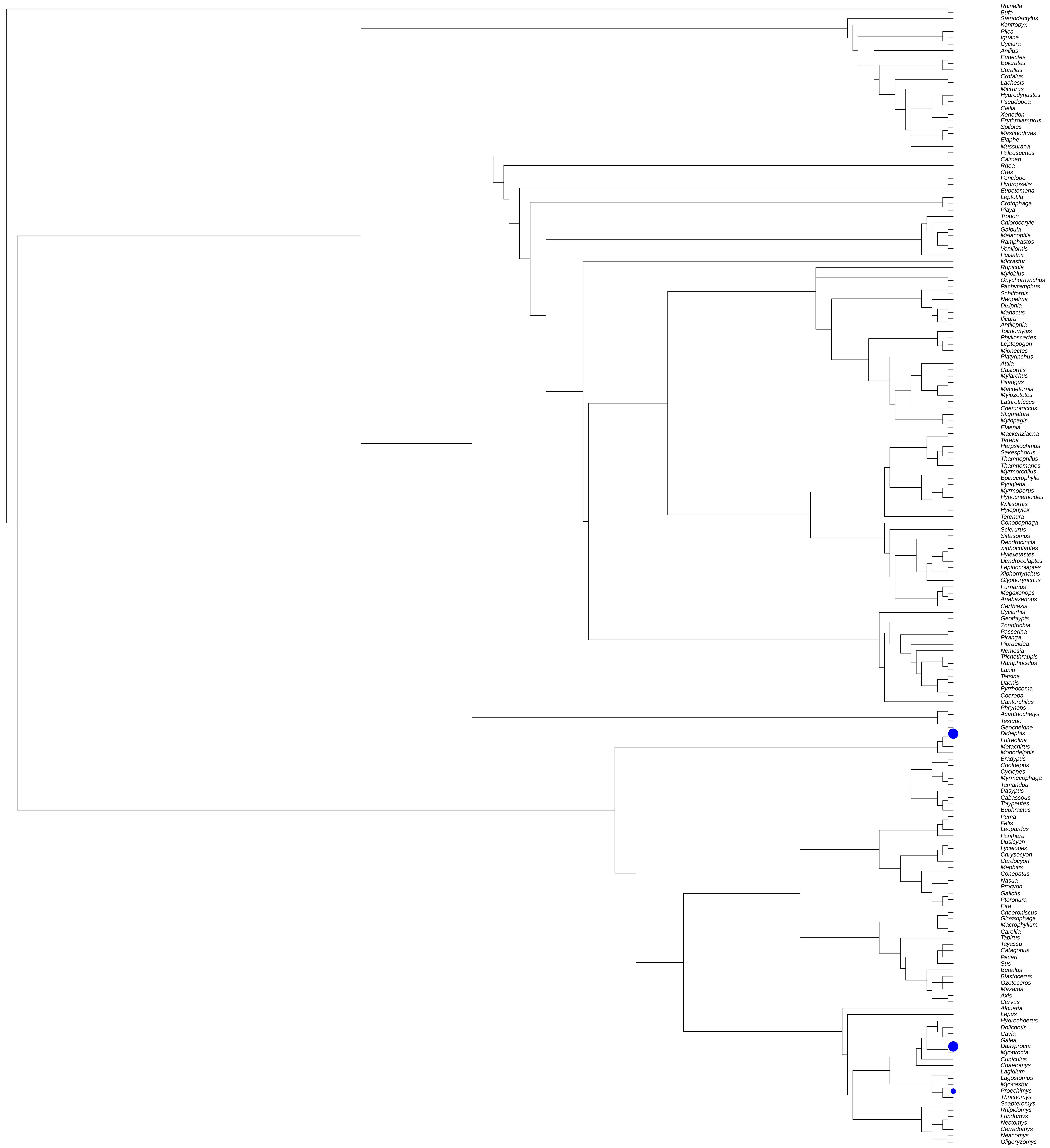

Amblyomma parkeri adult

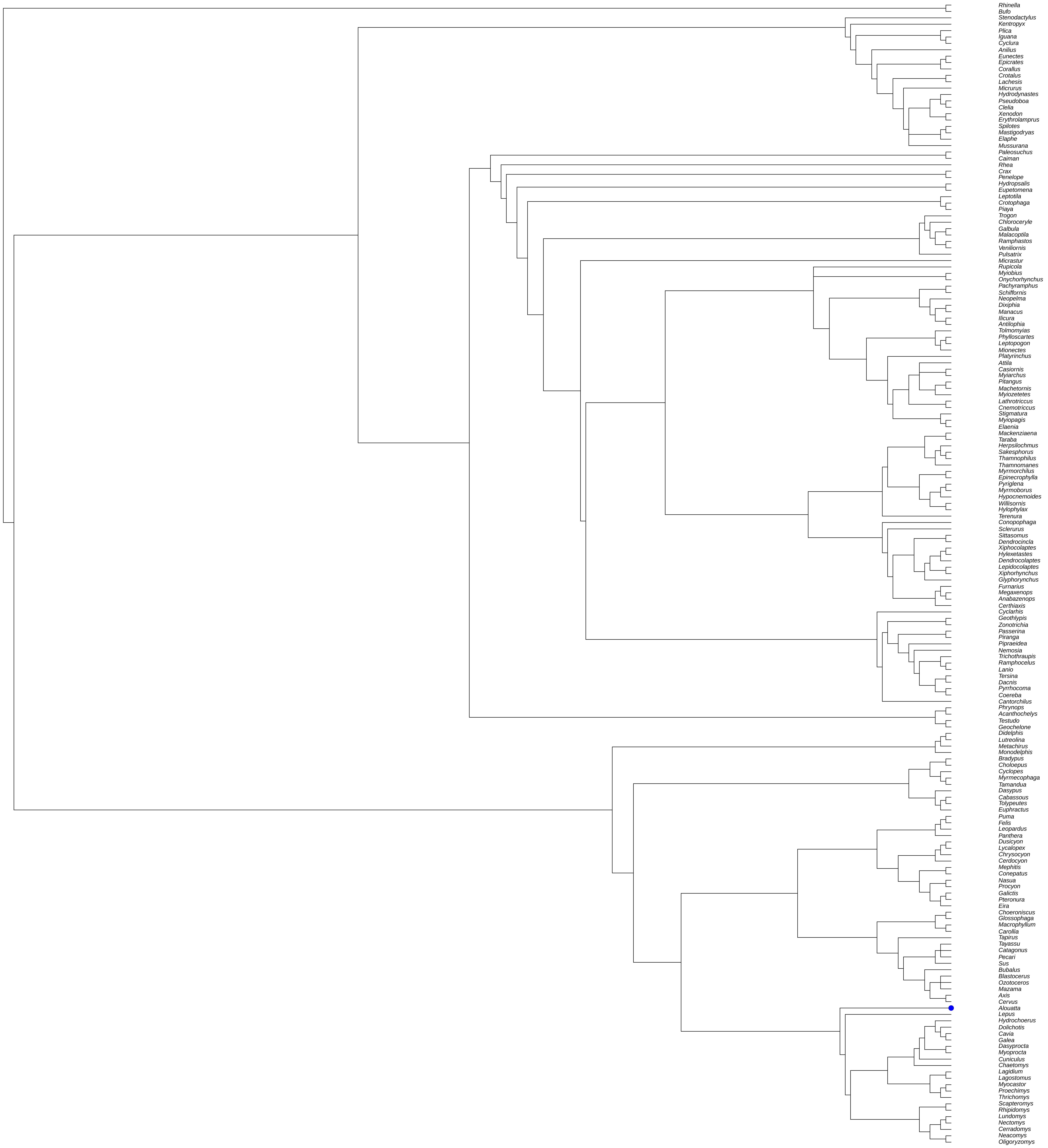

Amblyomma parkeri larva

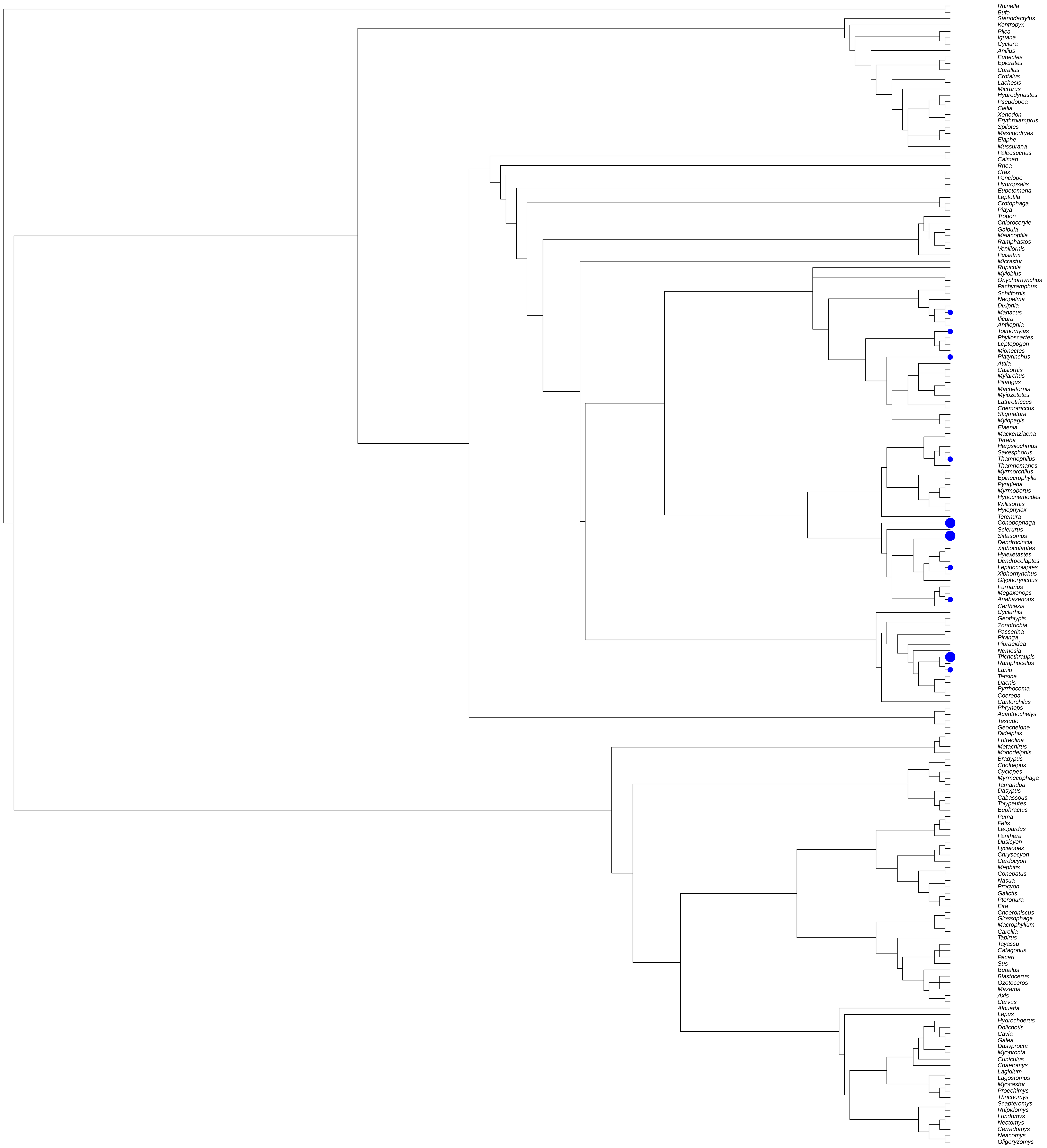

Amblyomma parkeri nymph

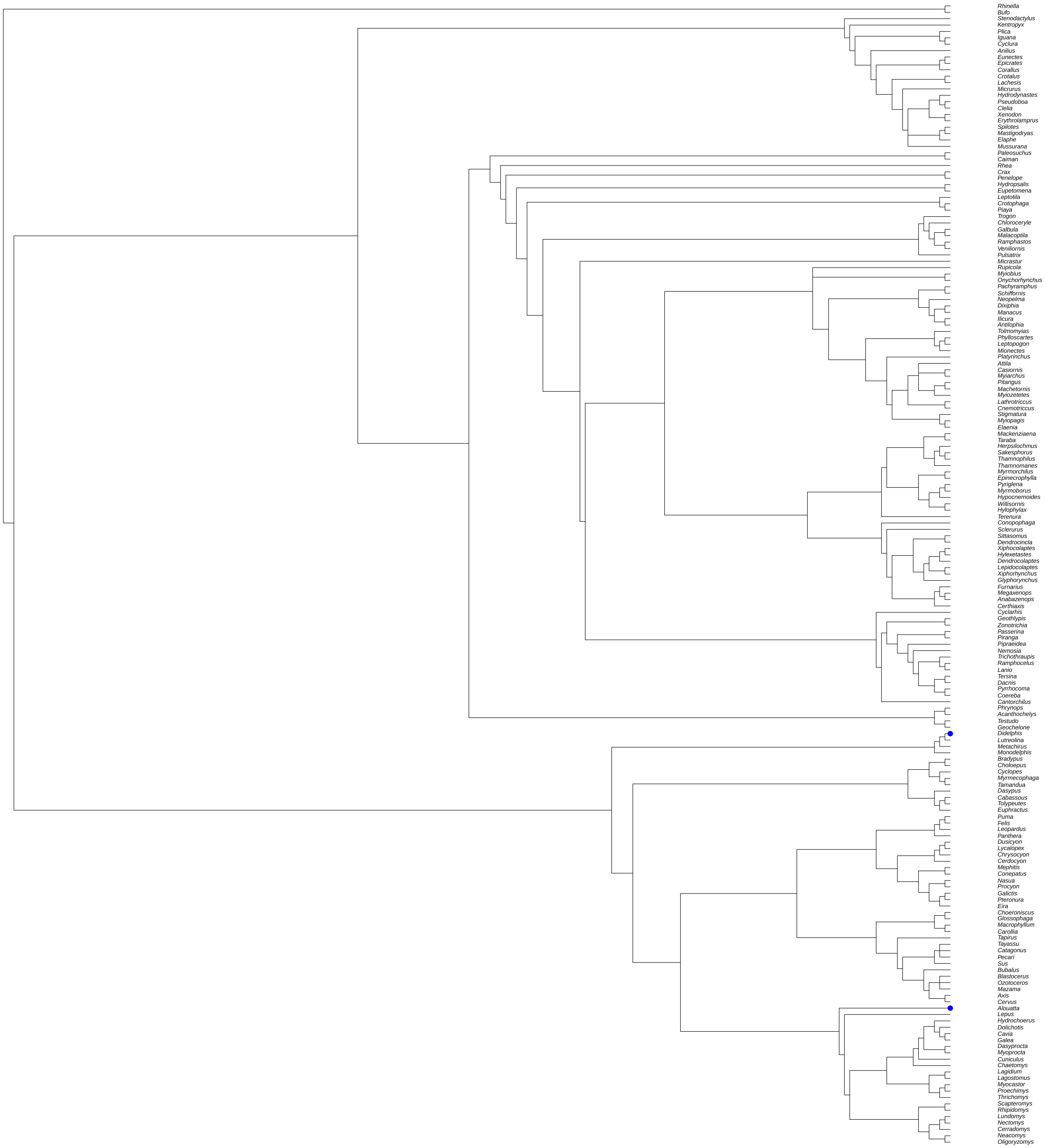

Amblyomma parvum adult

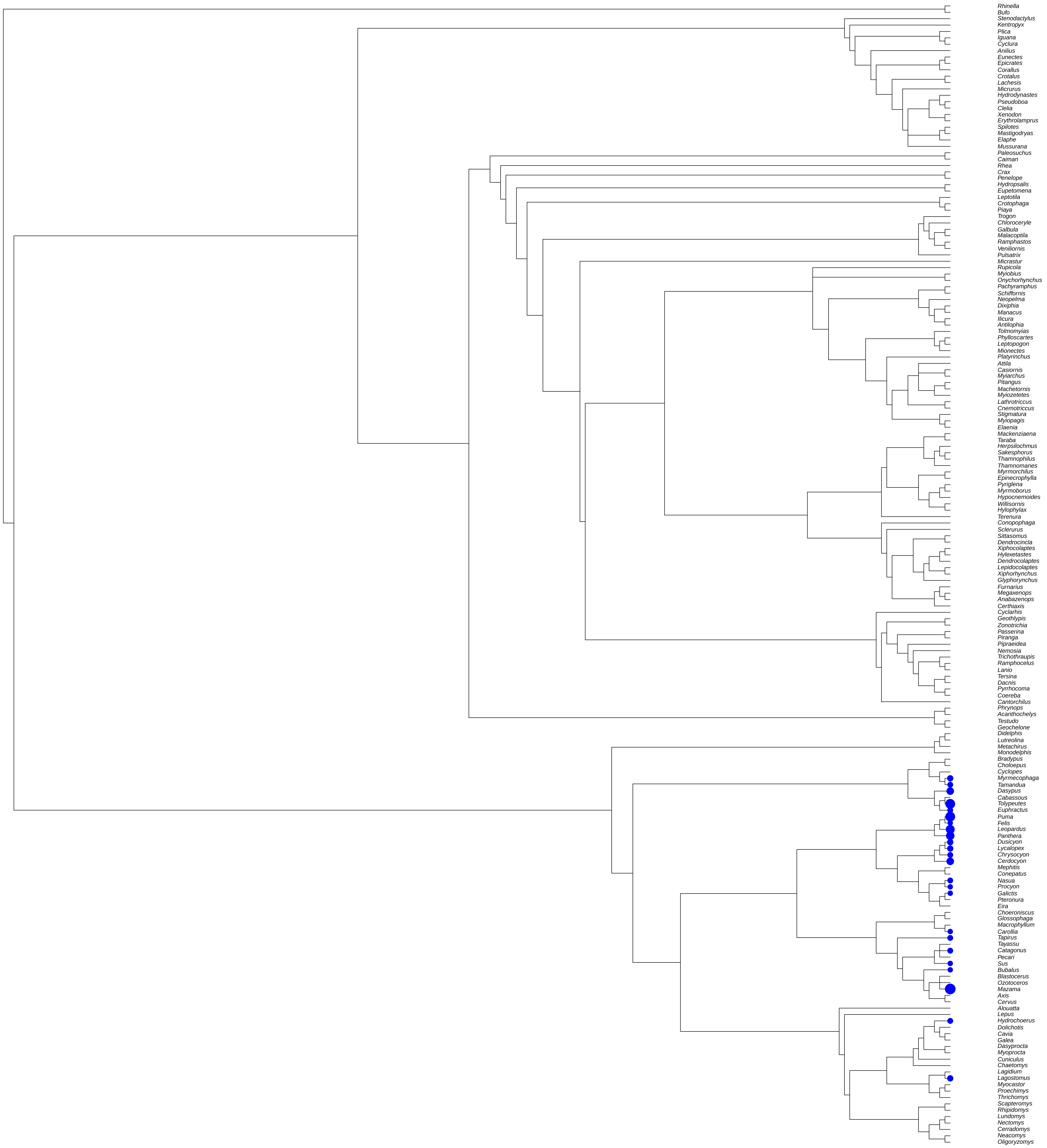

Amblyomma parvum larva

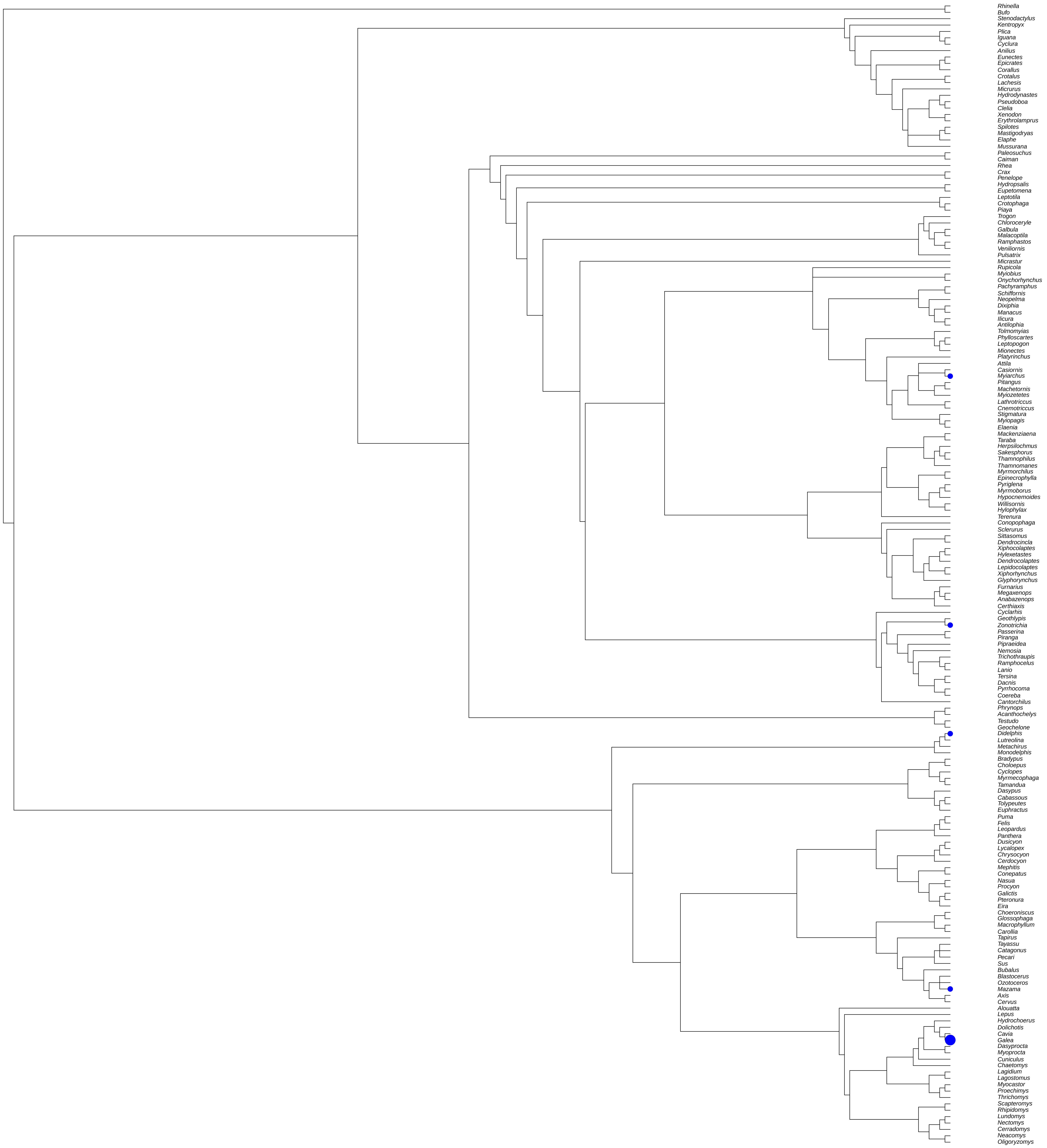

Amblyomma parvum nymph

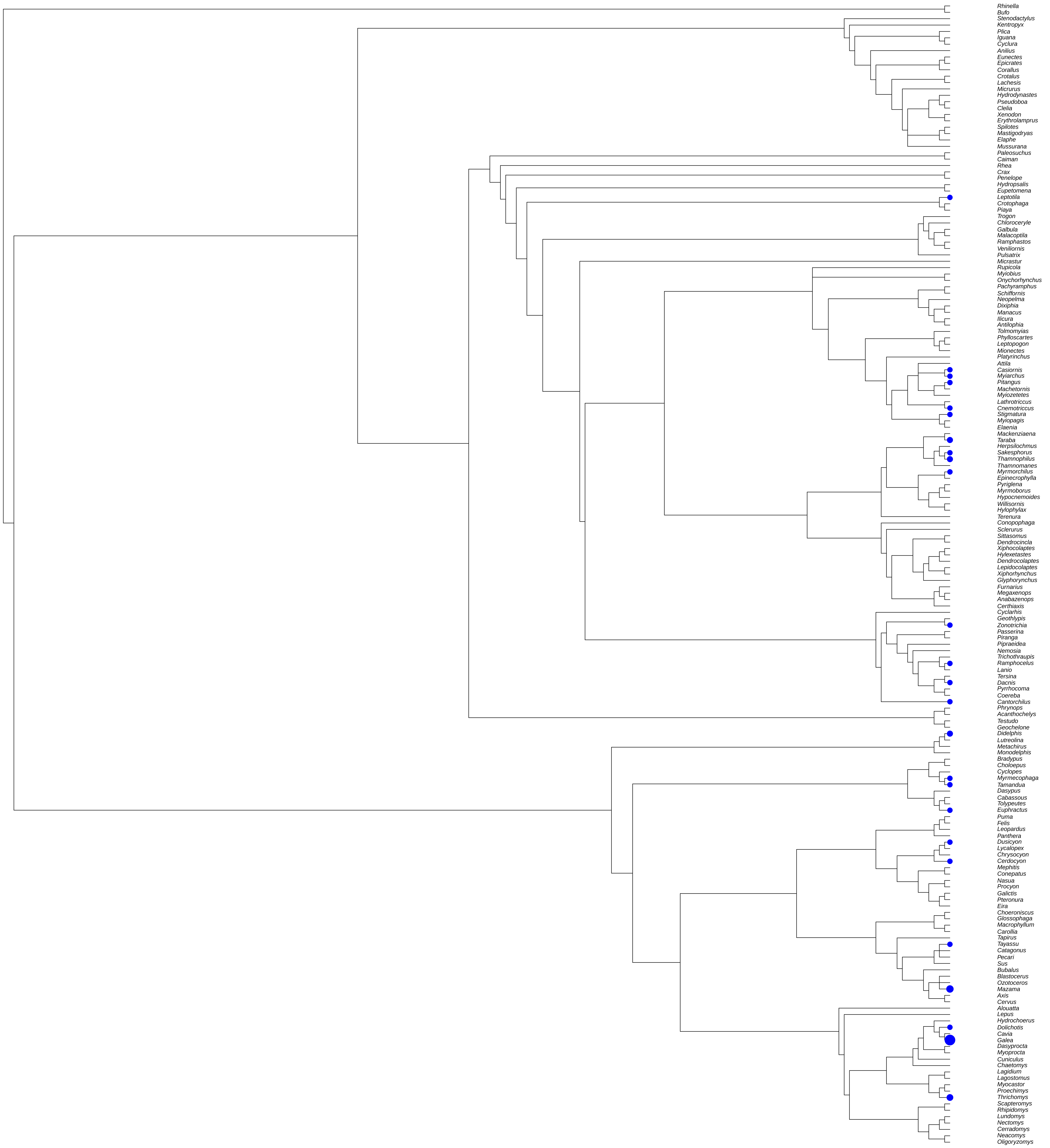

Amblyomma pseudoconcolor adult

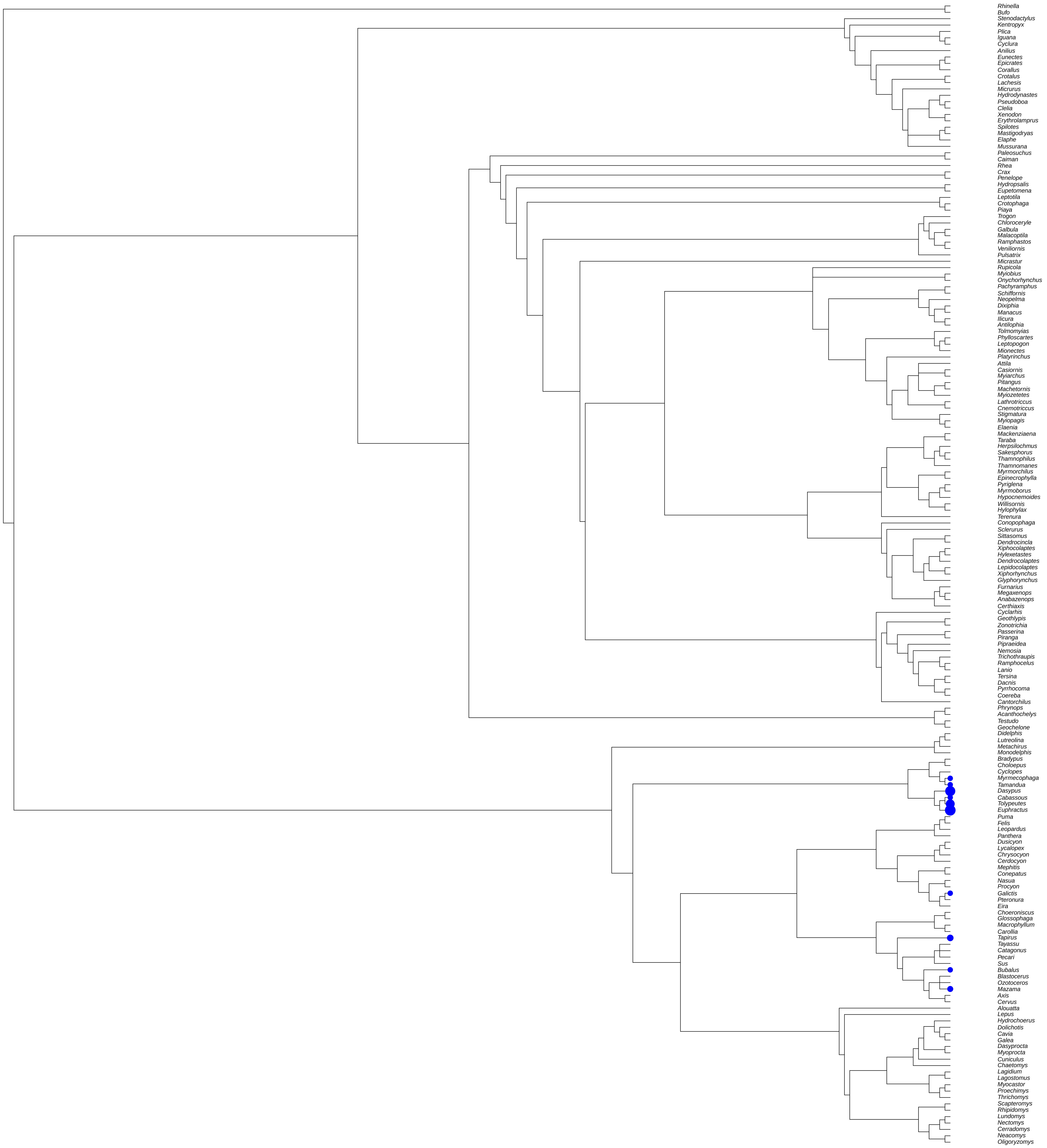

Amblyomma pseudoconcolor larva

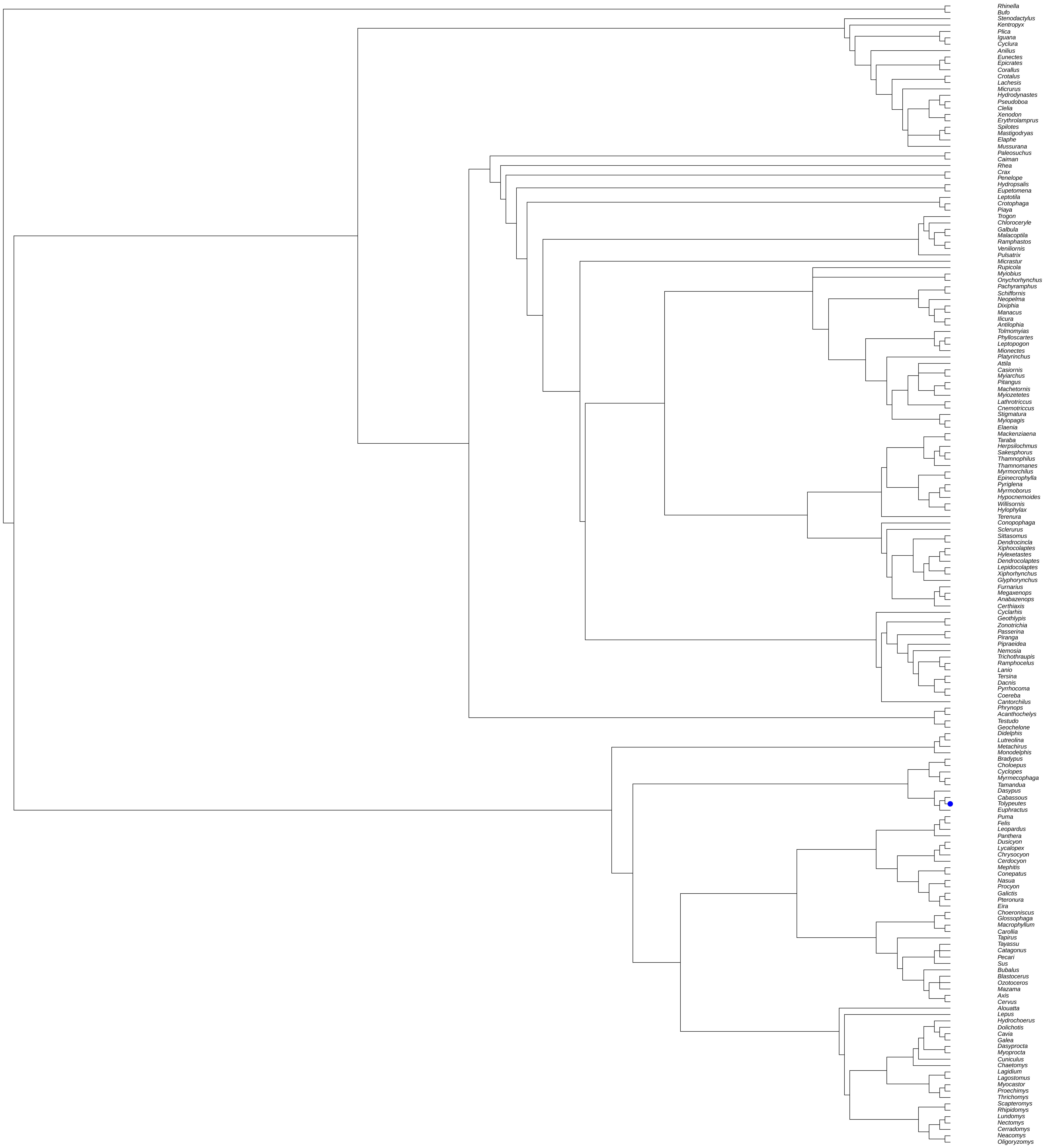

Amblyomma pseudoconcolor nymph

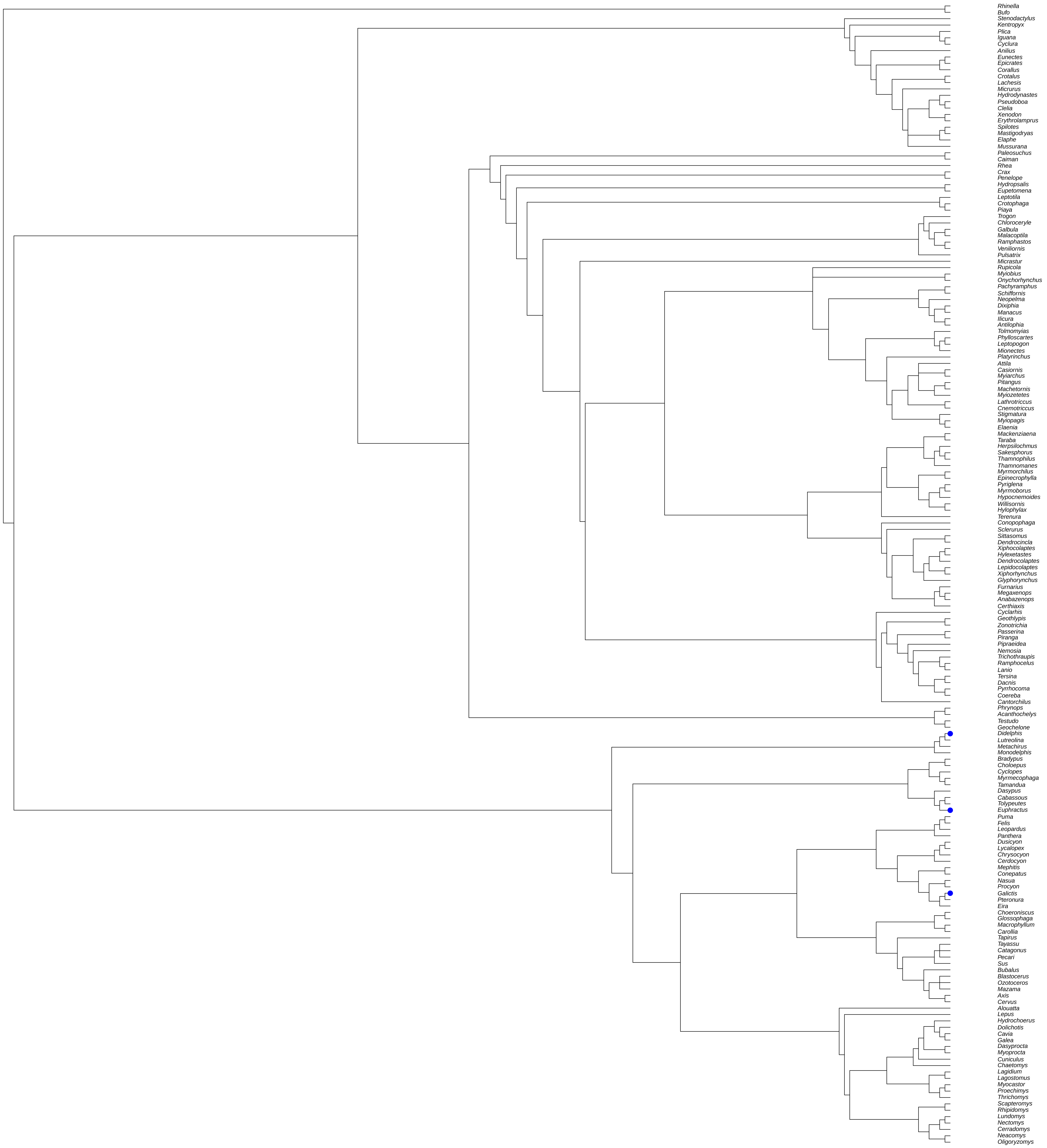

Amblyomma pseudoparvum adult

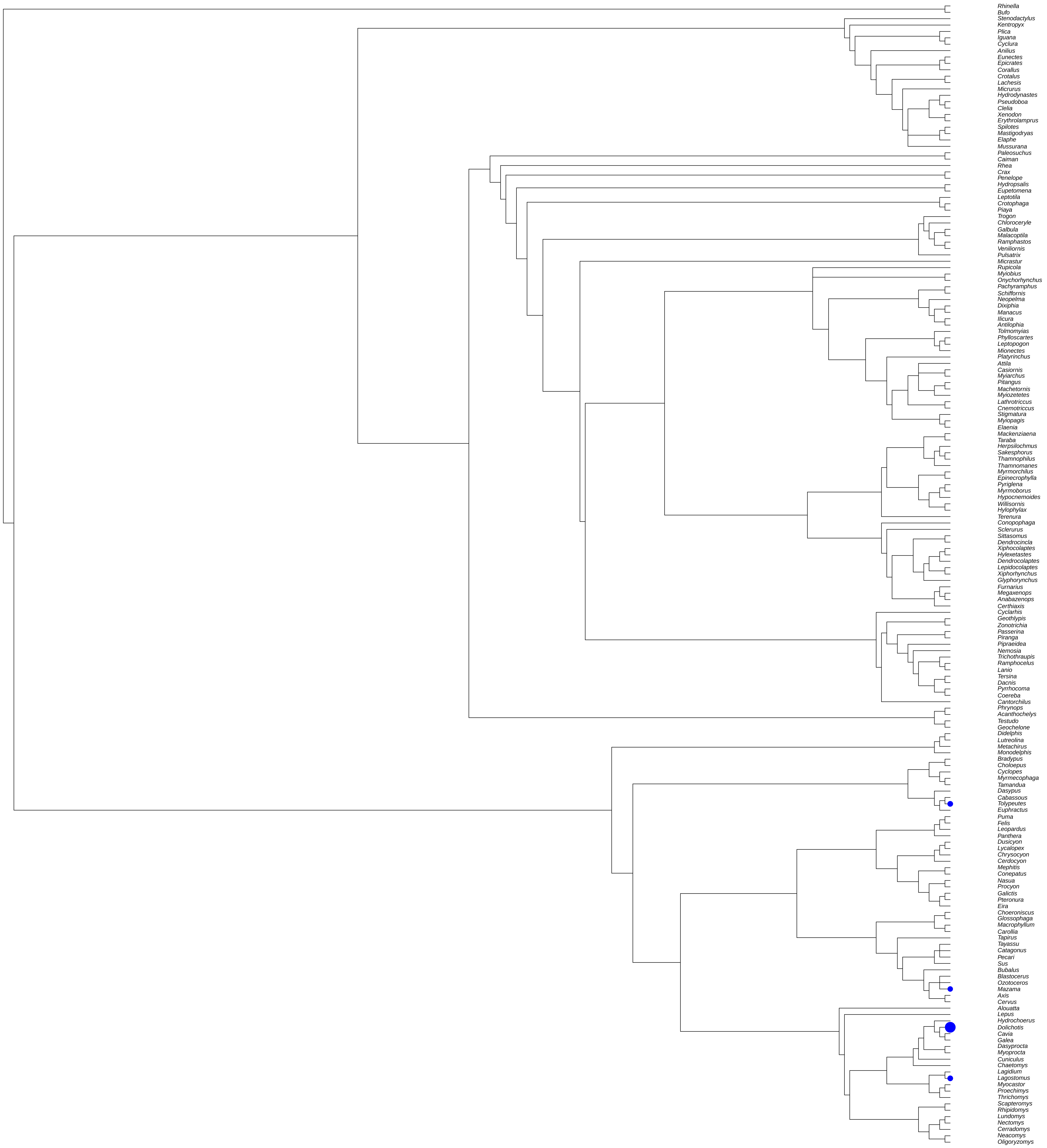

Amblyomma pseudoparvum larva

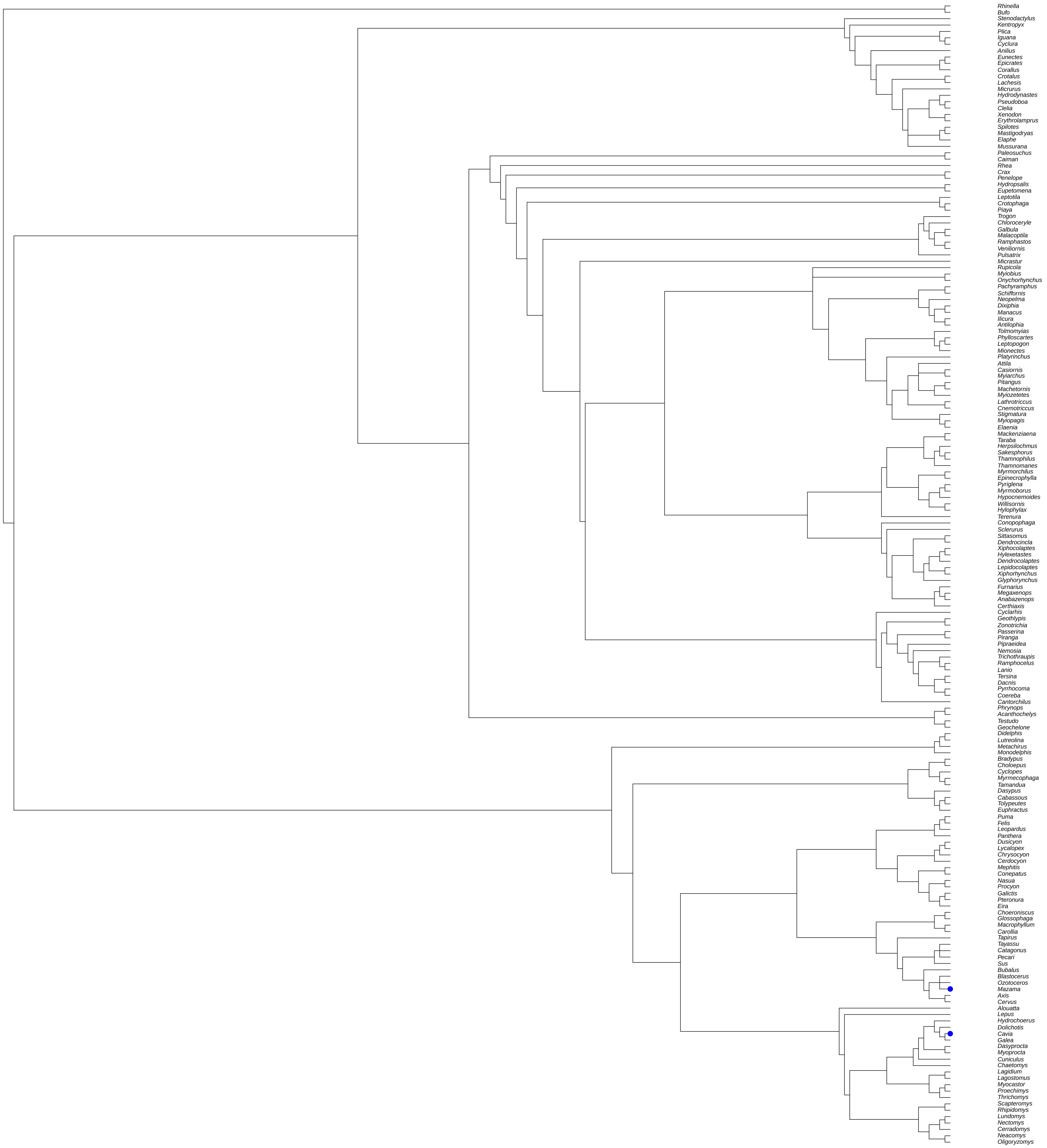

Amblyomma pseudoparvum nymph

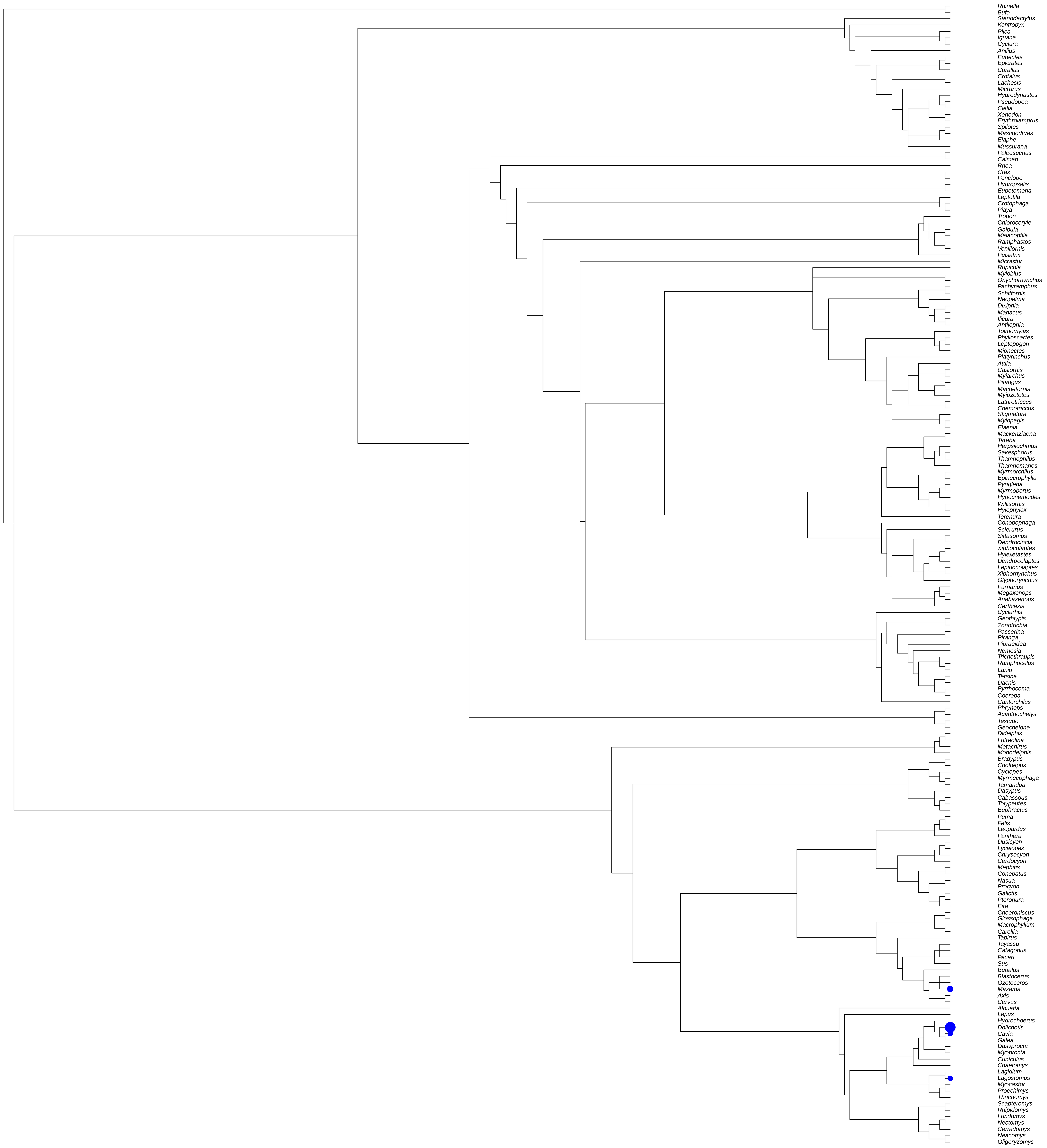

Amblyomma romitti adult

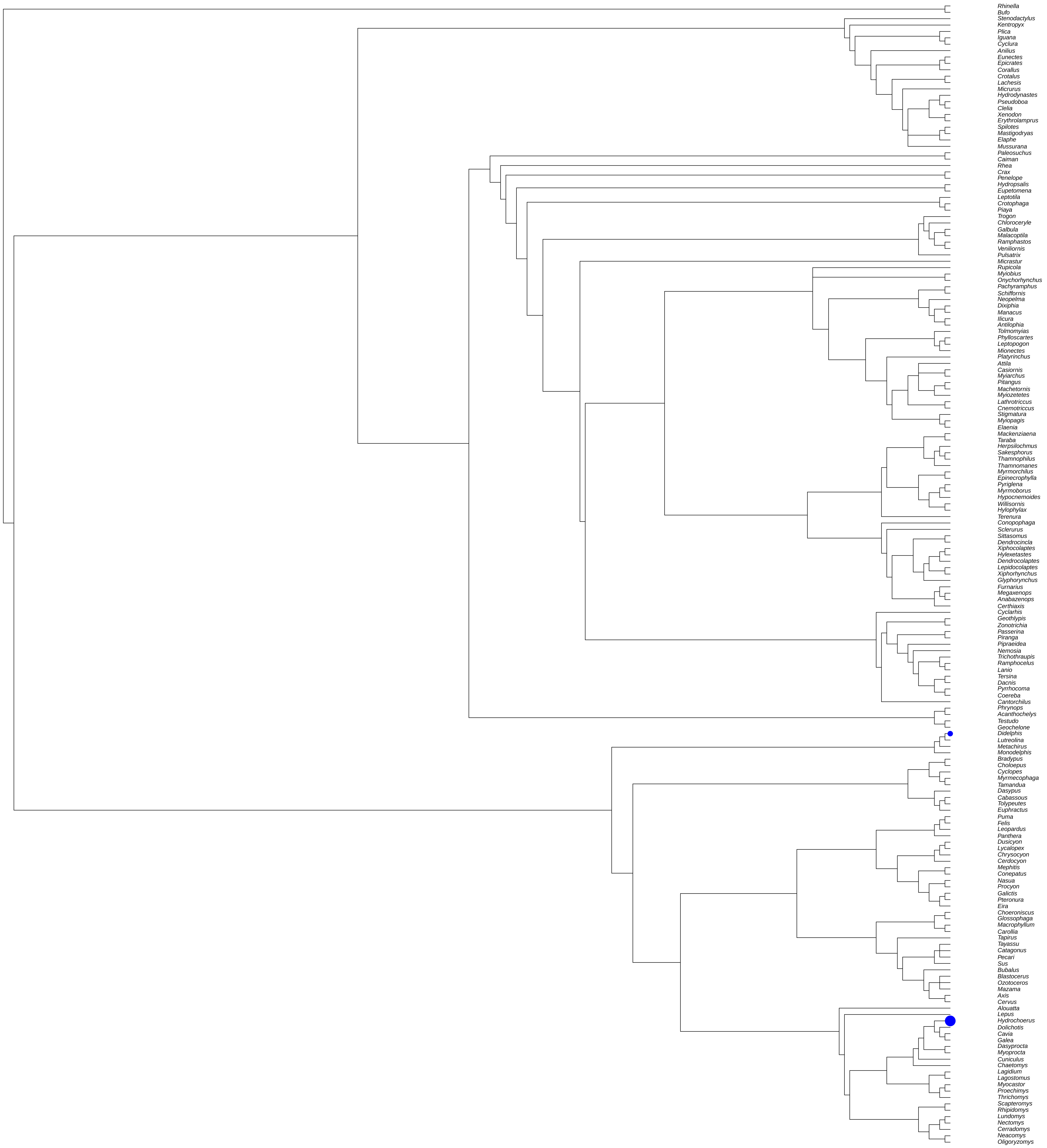

Amblyomma romitti nymph

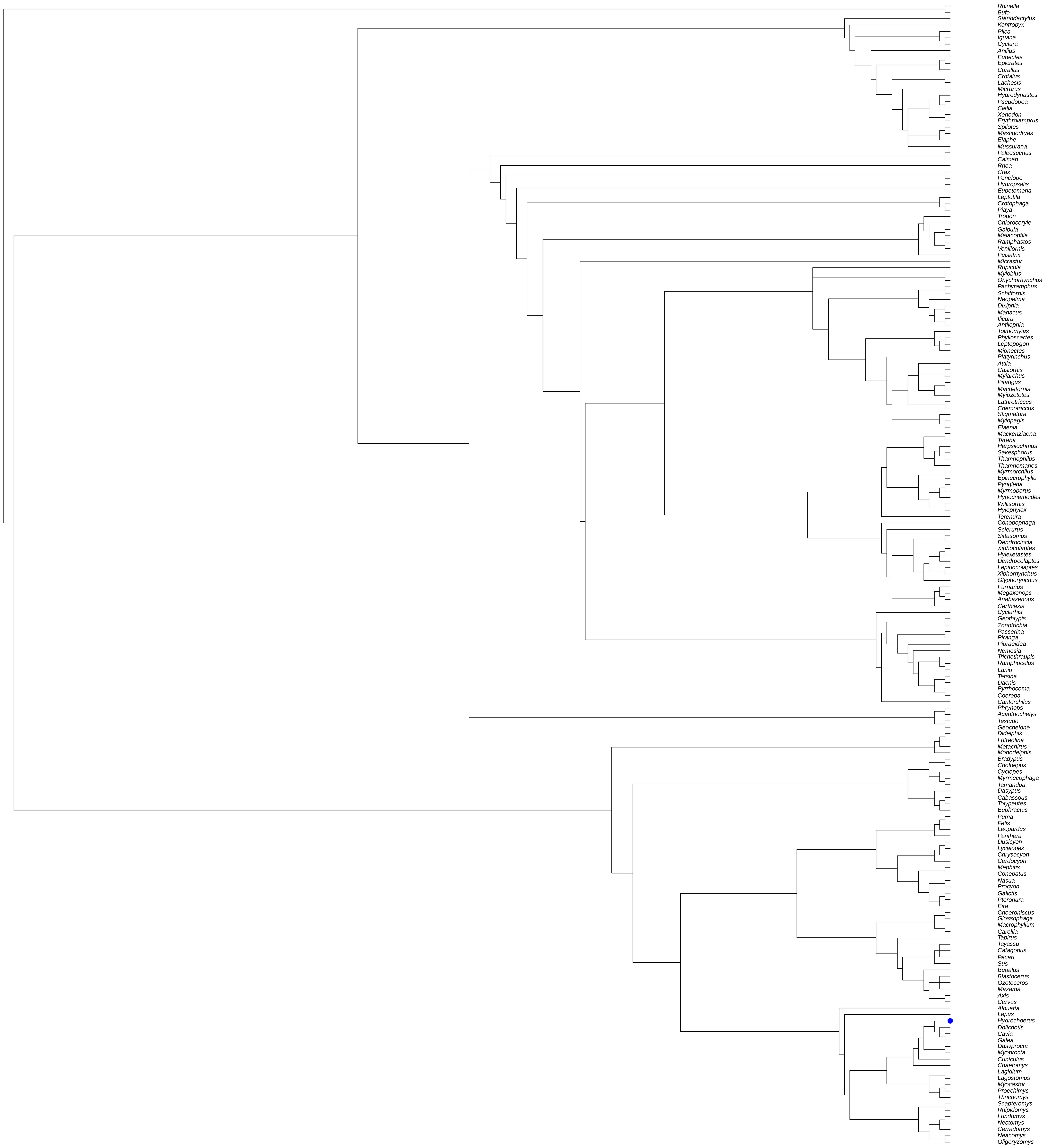

Amblyomma rotundatum adult

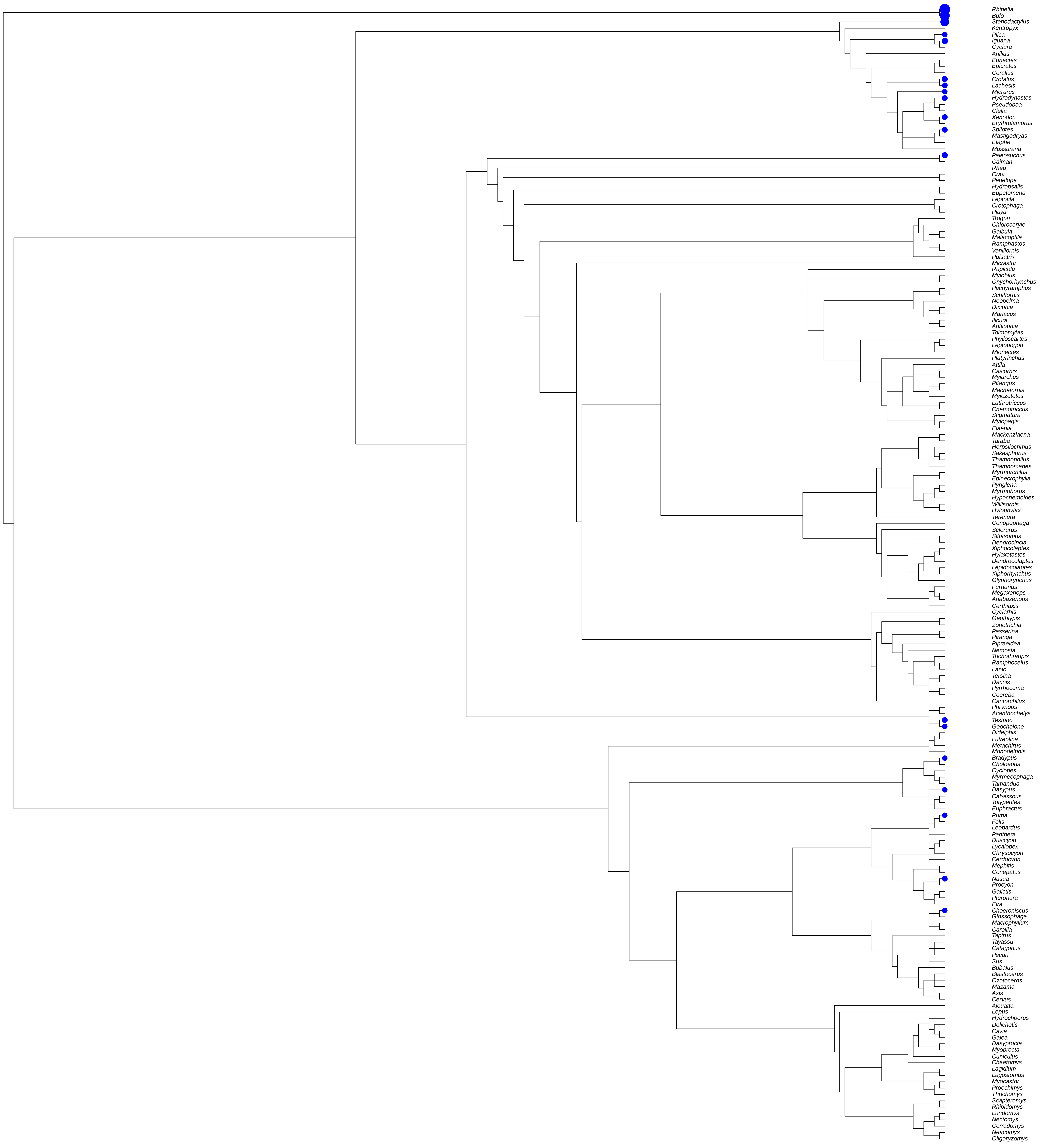

Amblyomma rotundatum larva

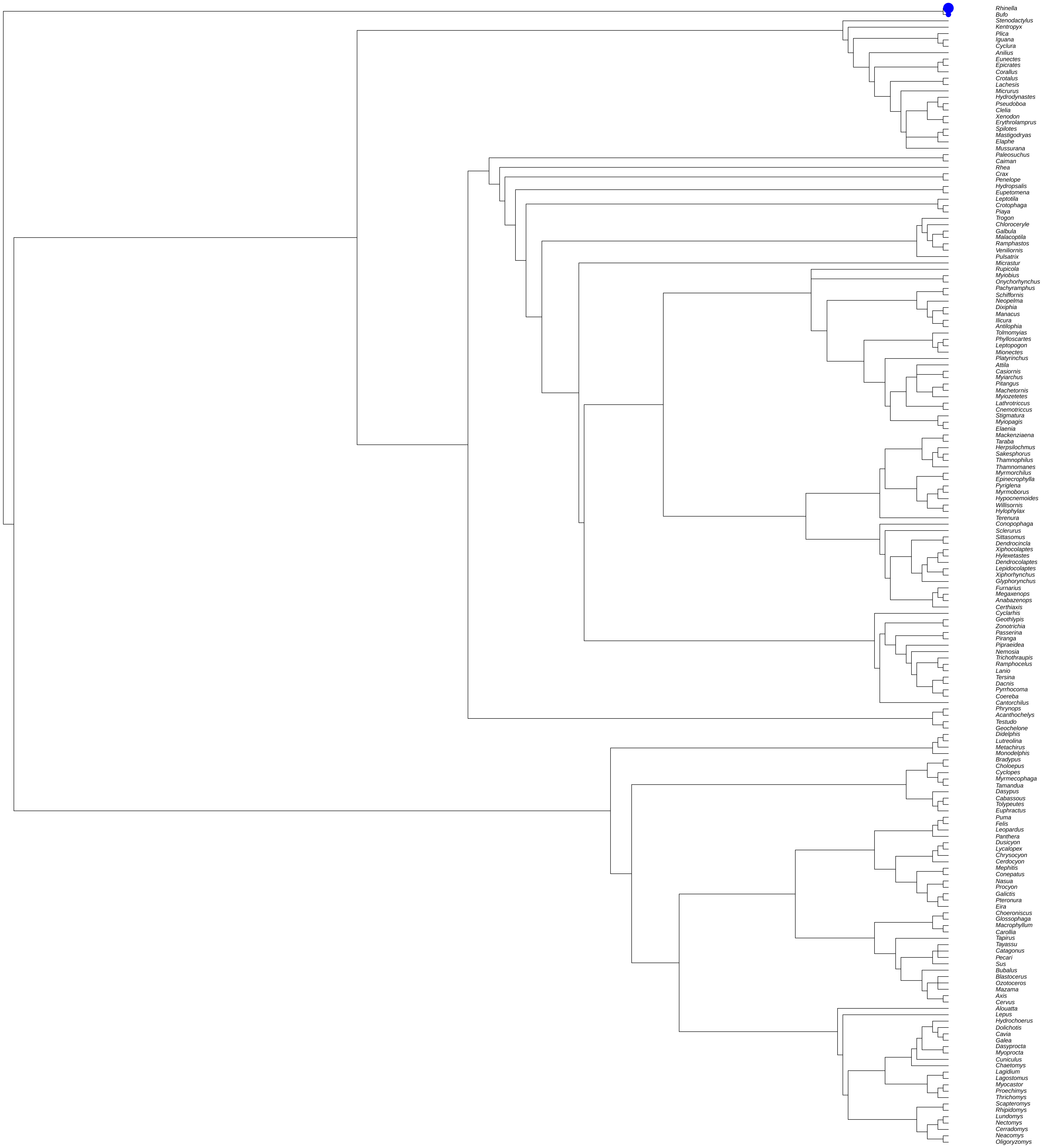

Amblyomma rotundatum nymph

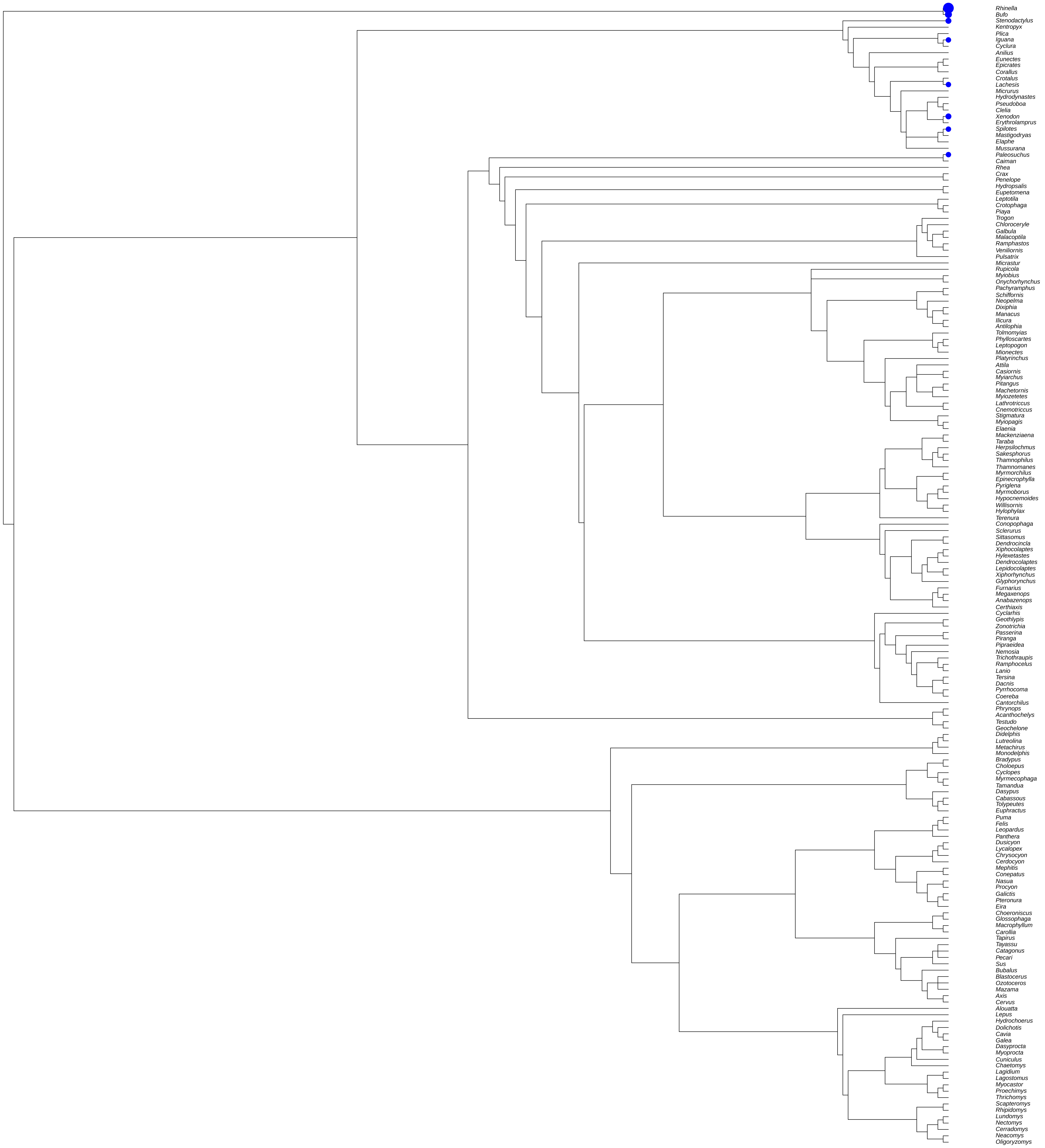

Amblyomma scalpturatum adult

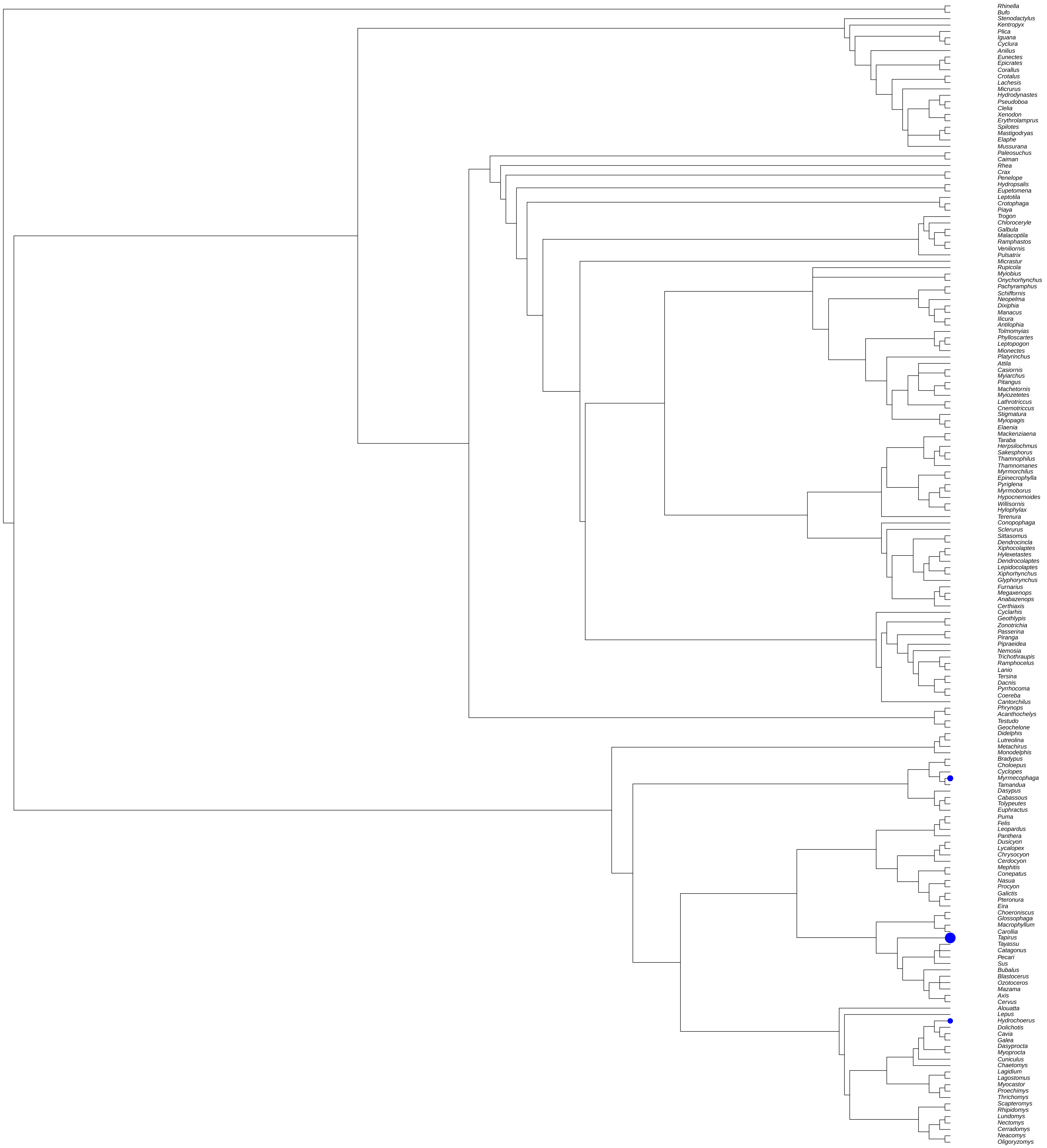

Amblyomma scalpturatum nymph

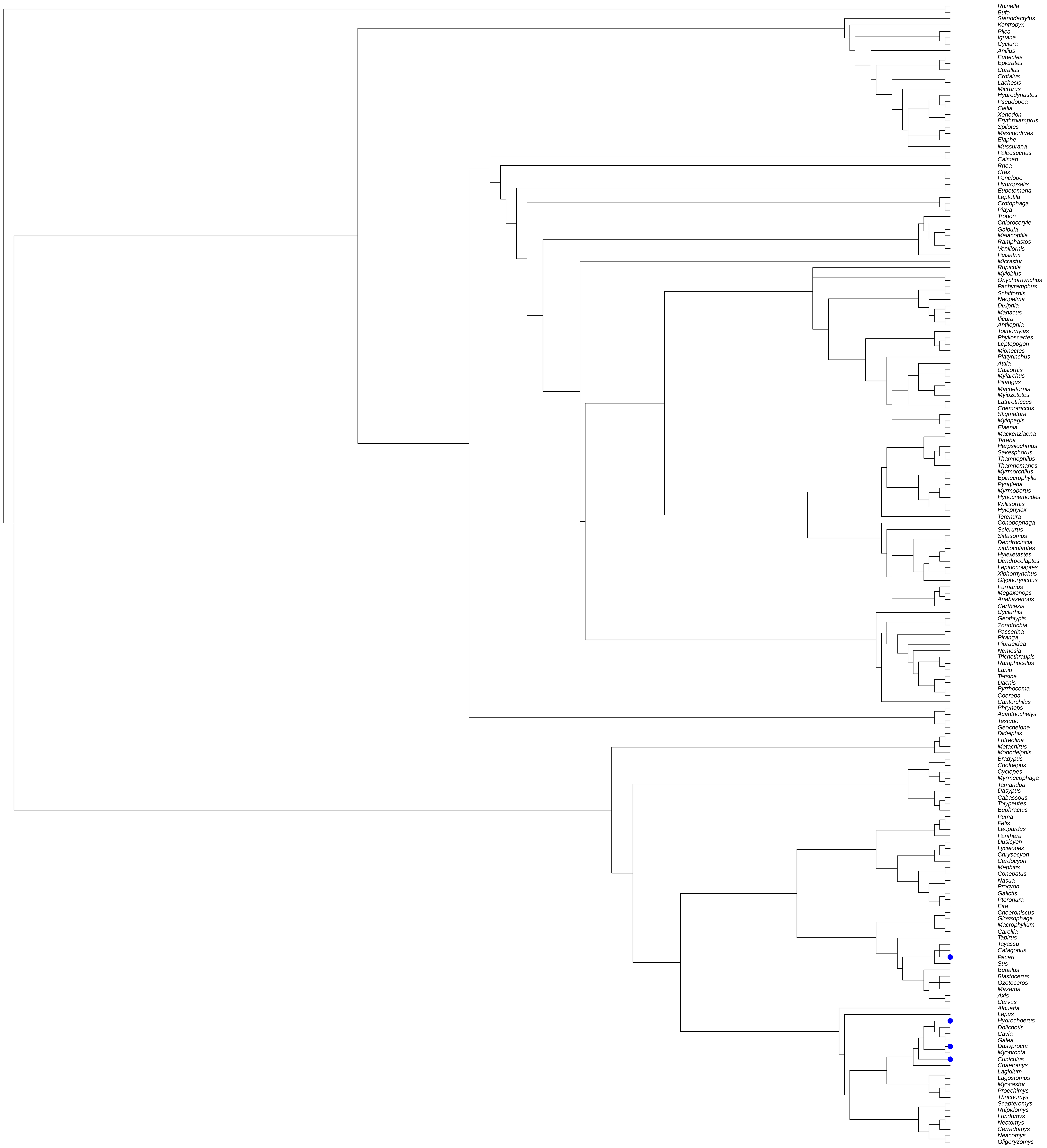

Amblyomma sculptum adult

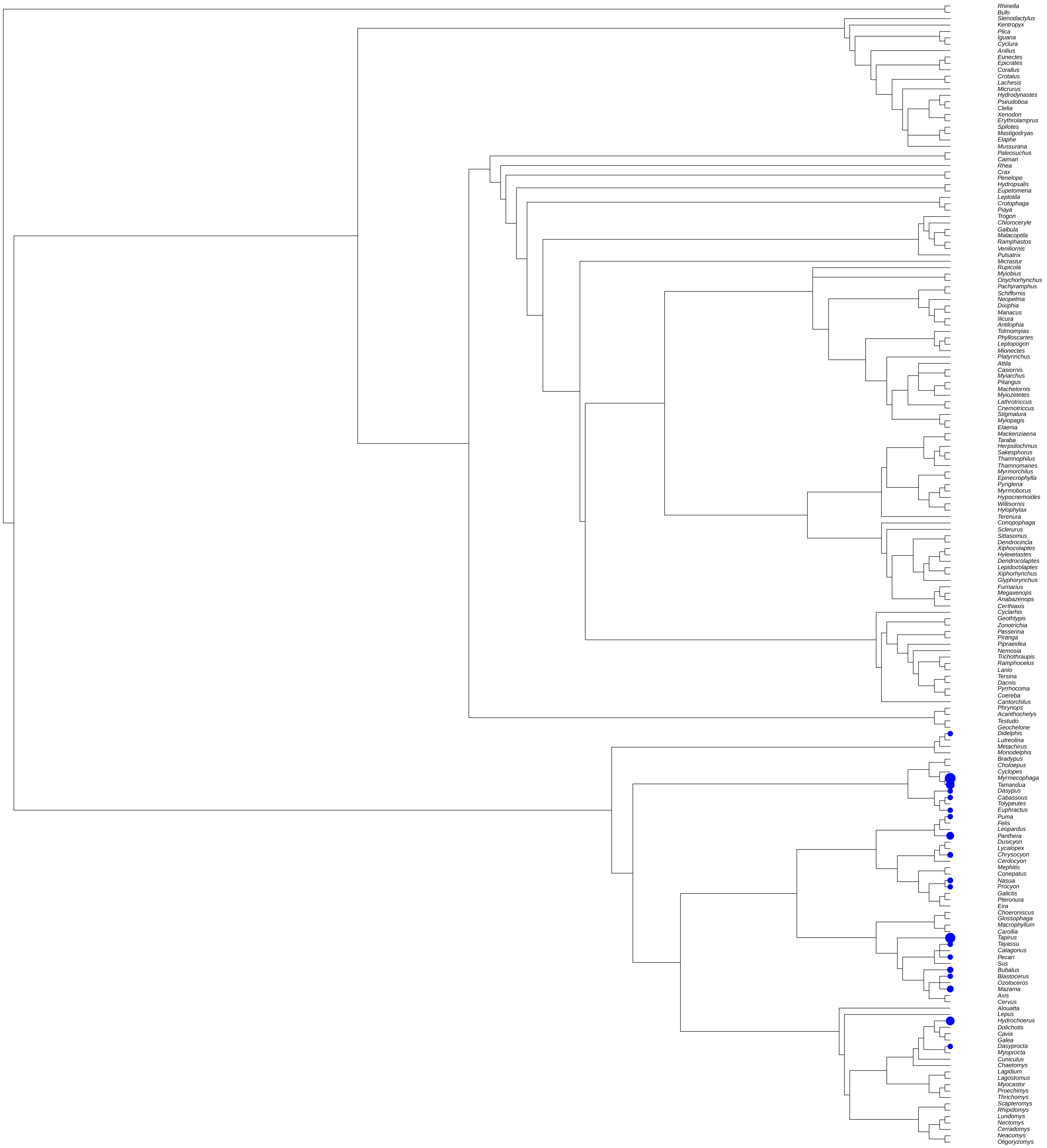

Amblyomma sculptum larva

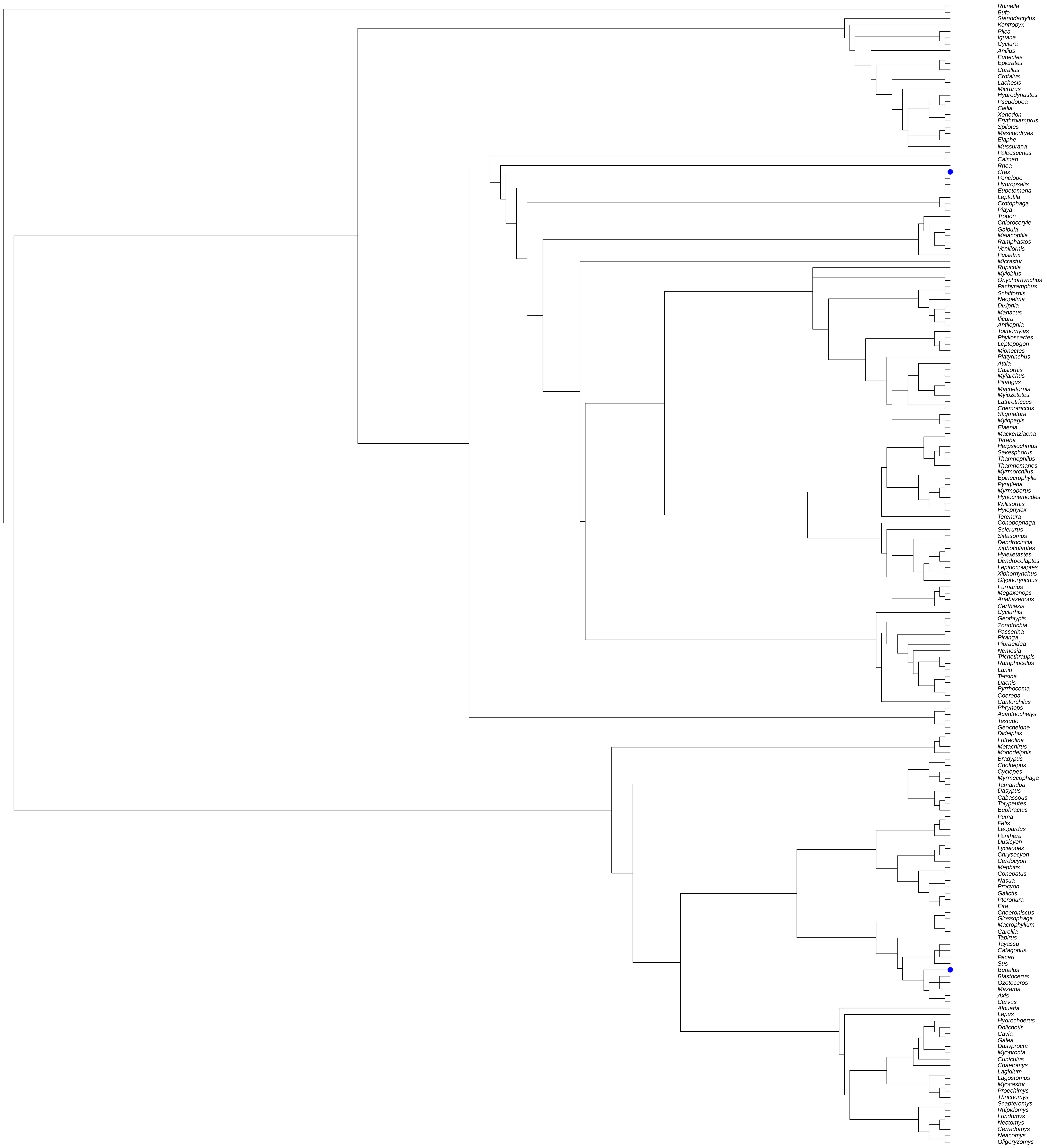

Amblyomma sculptum nymph

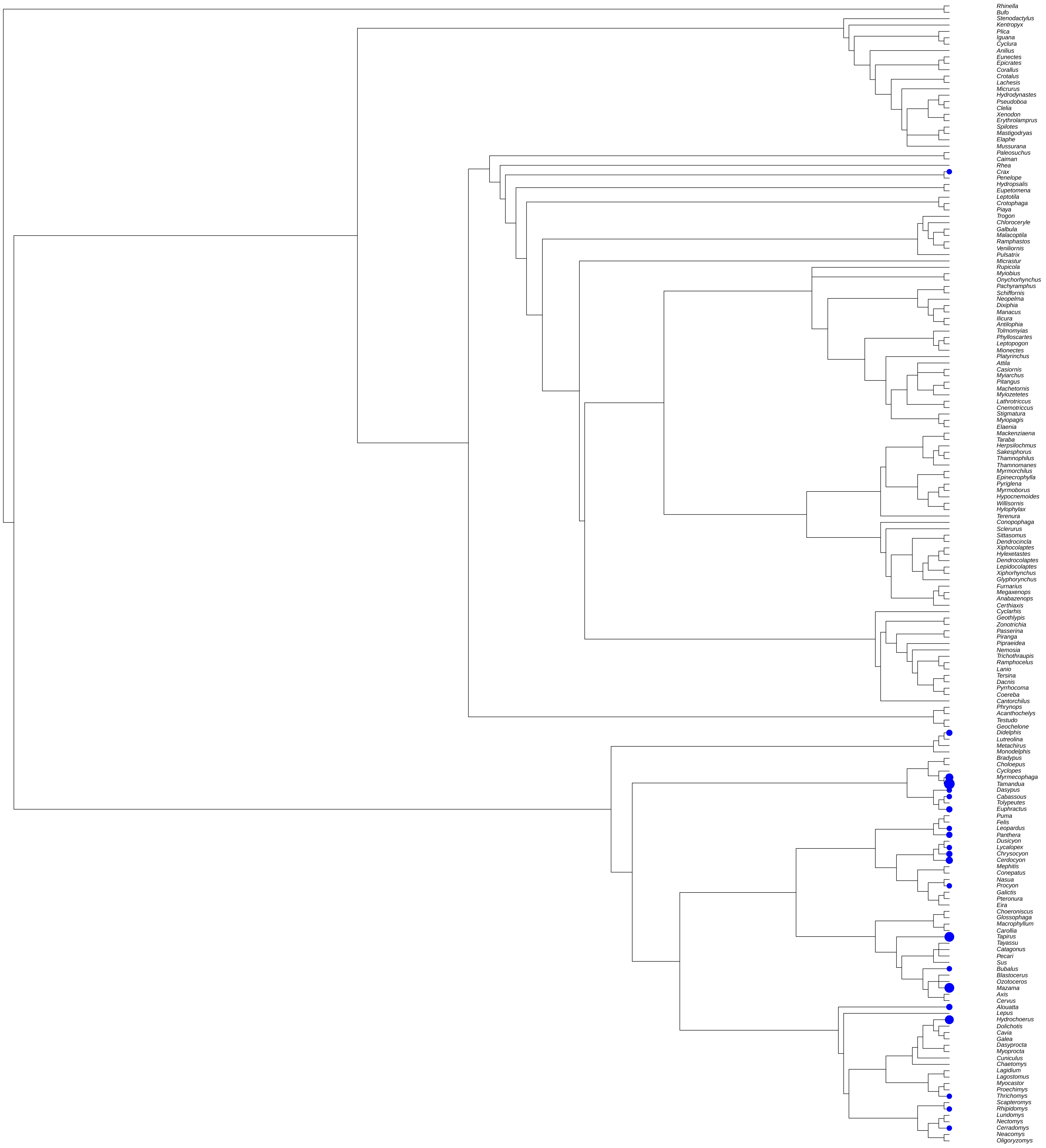

Amblyomma tigrinum adult

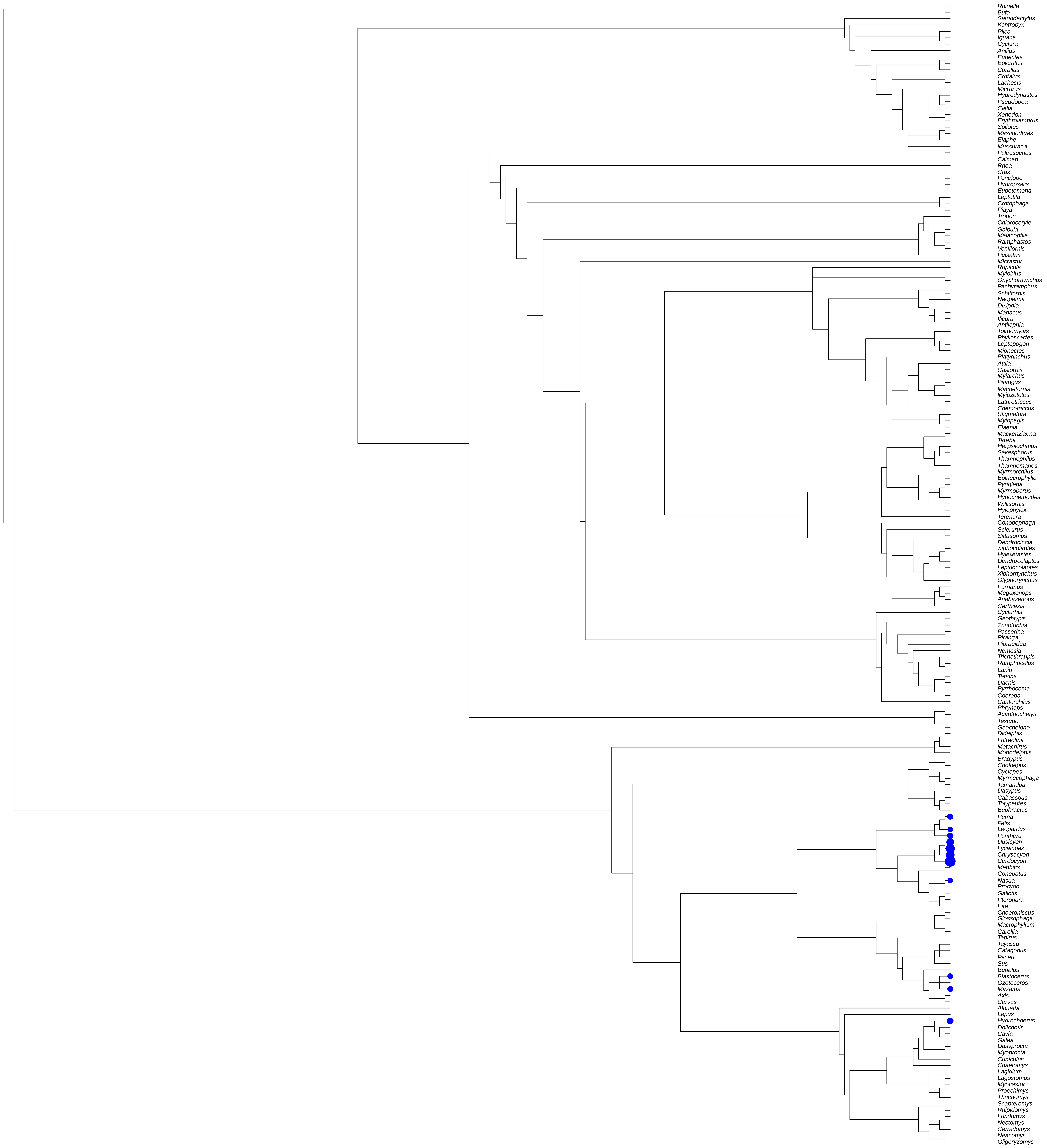

Amblyomma tigrinum larva

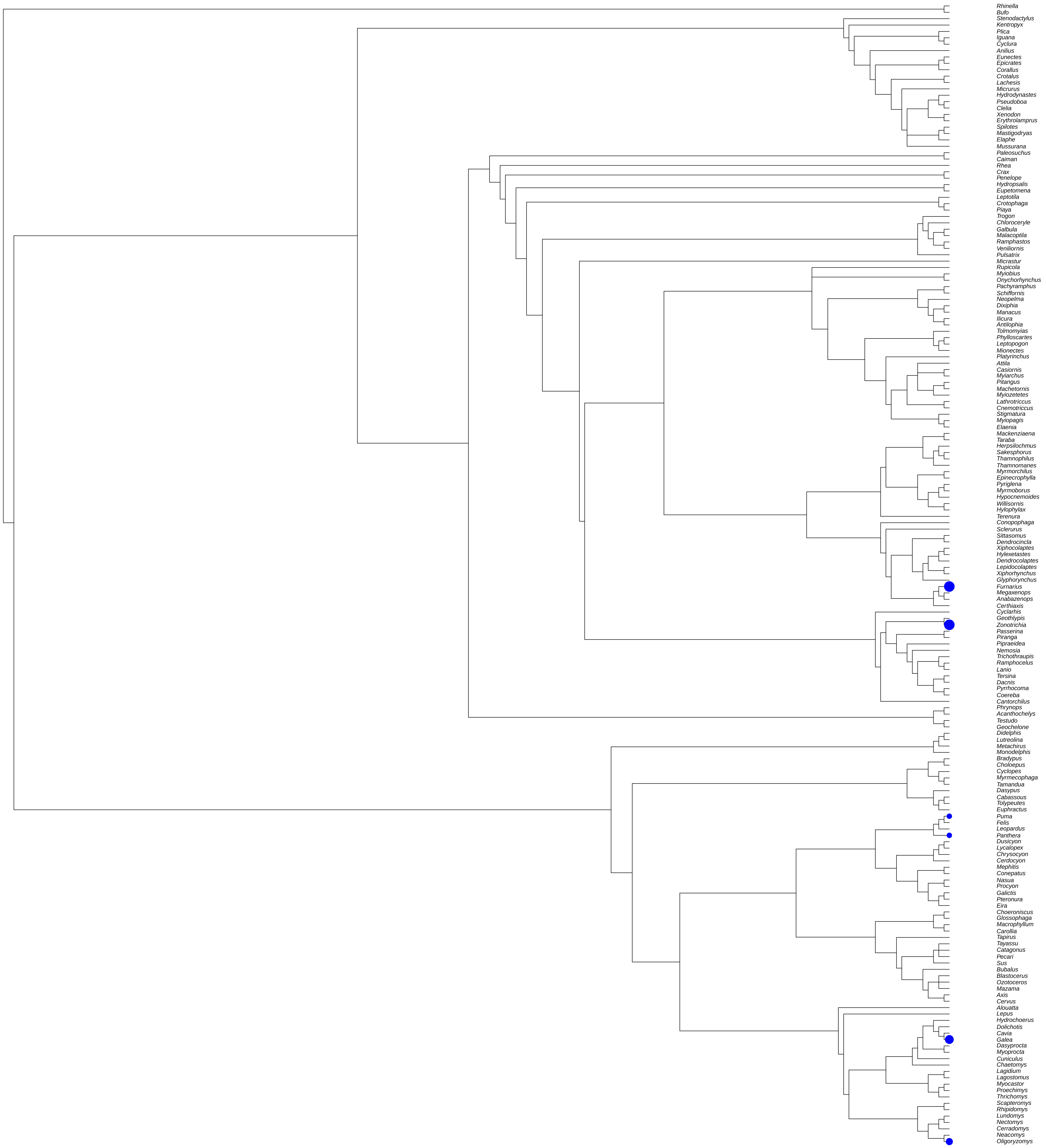

Amblyomma tigrinum nymph

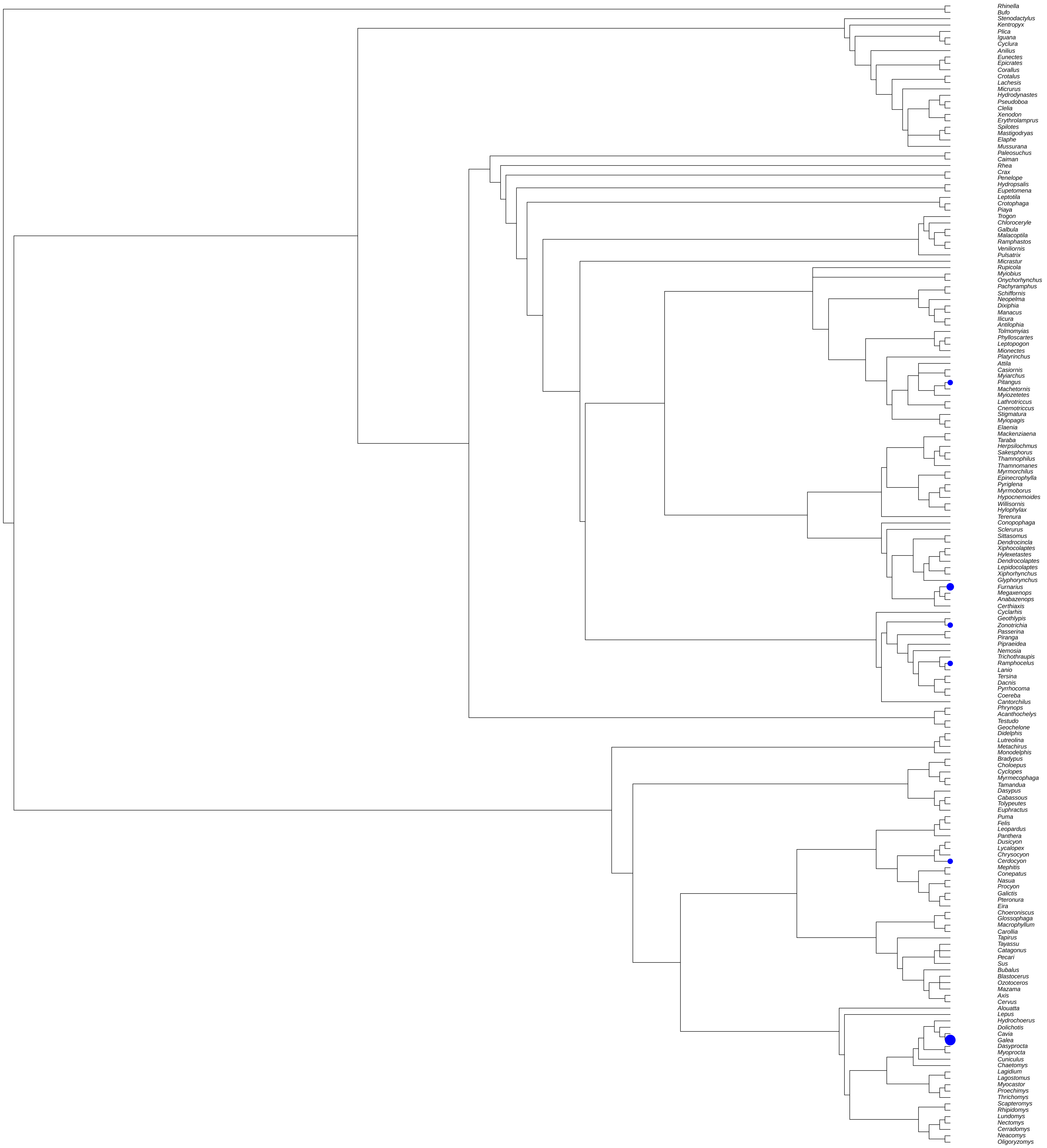

Amblyomma triste adult

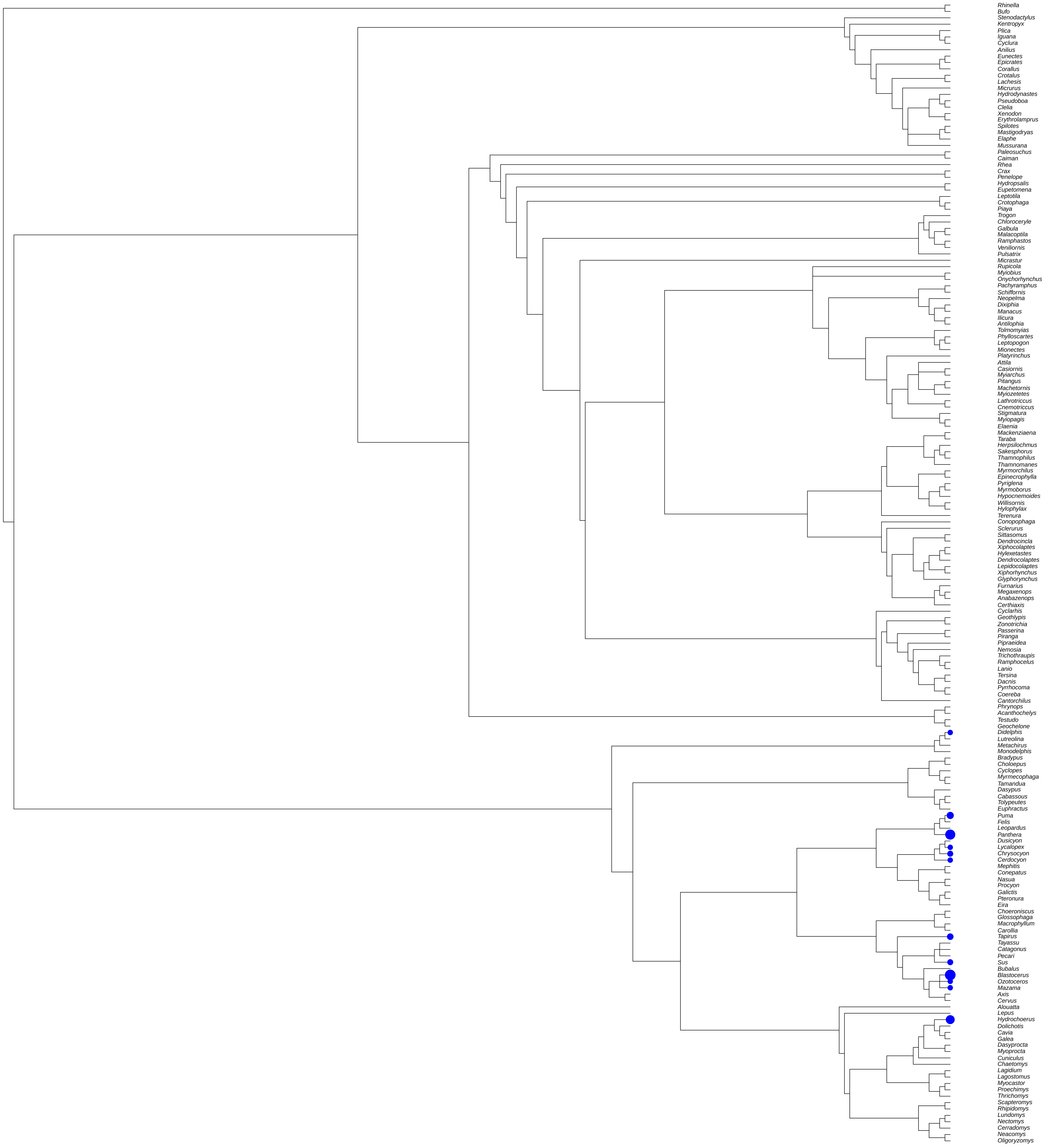

Amblyomma triste larva

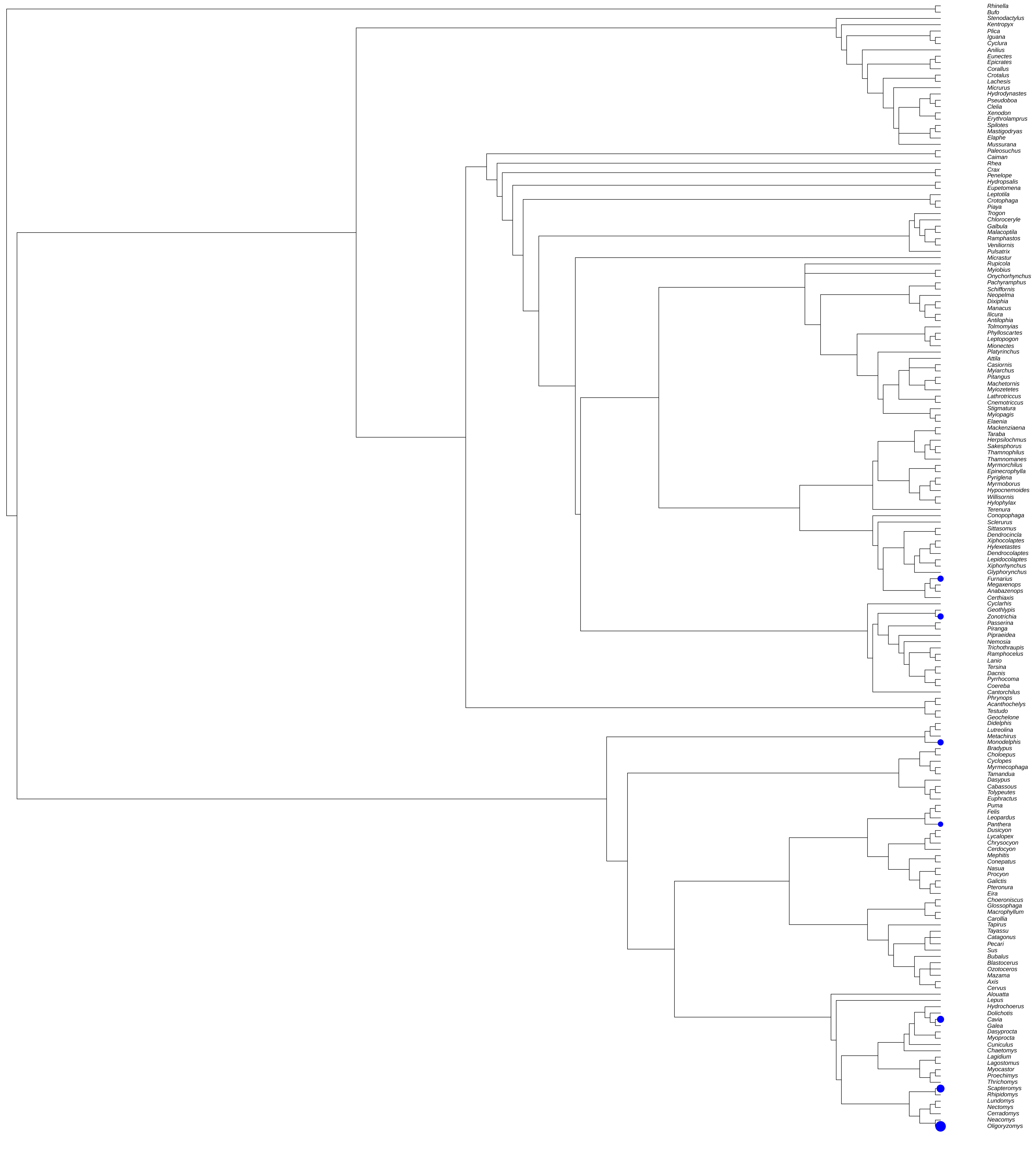

Amblyomma triste nymph

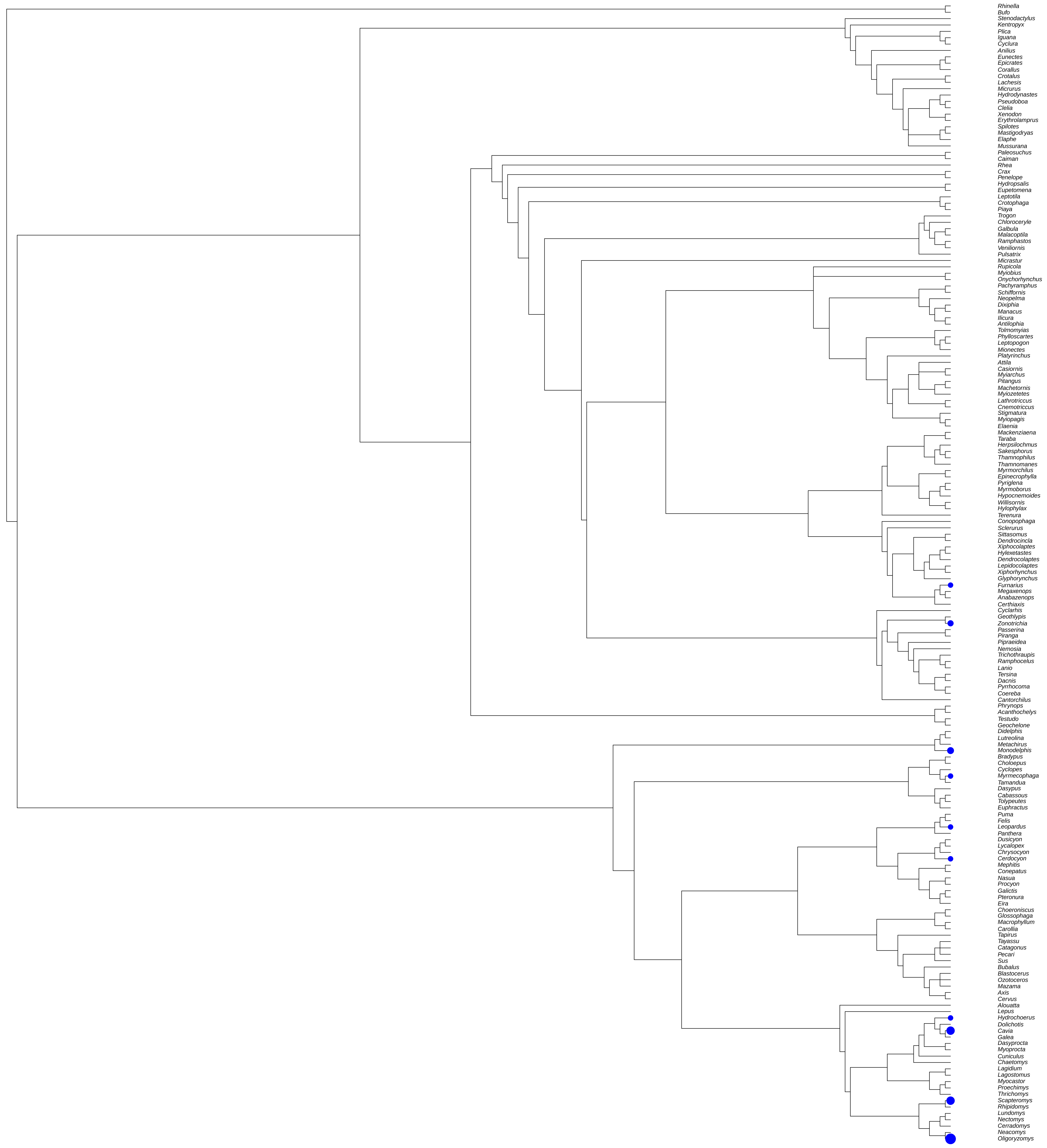

Amblyomma varium adult

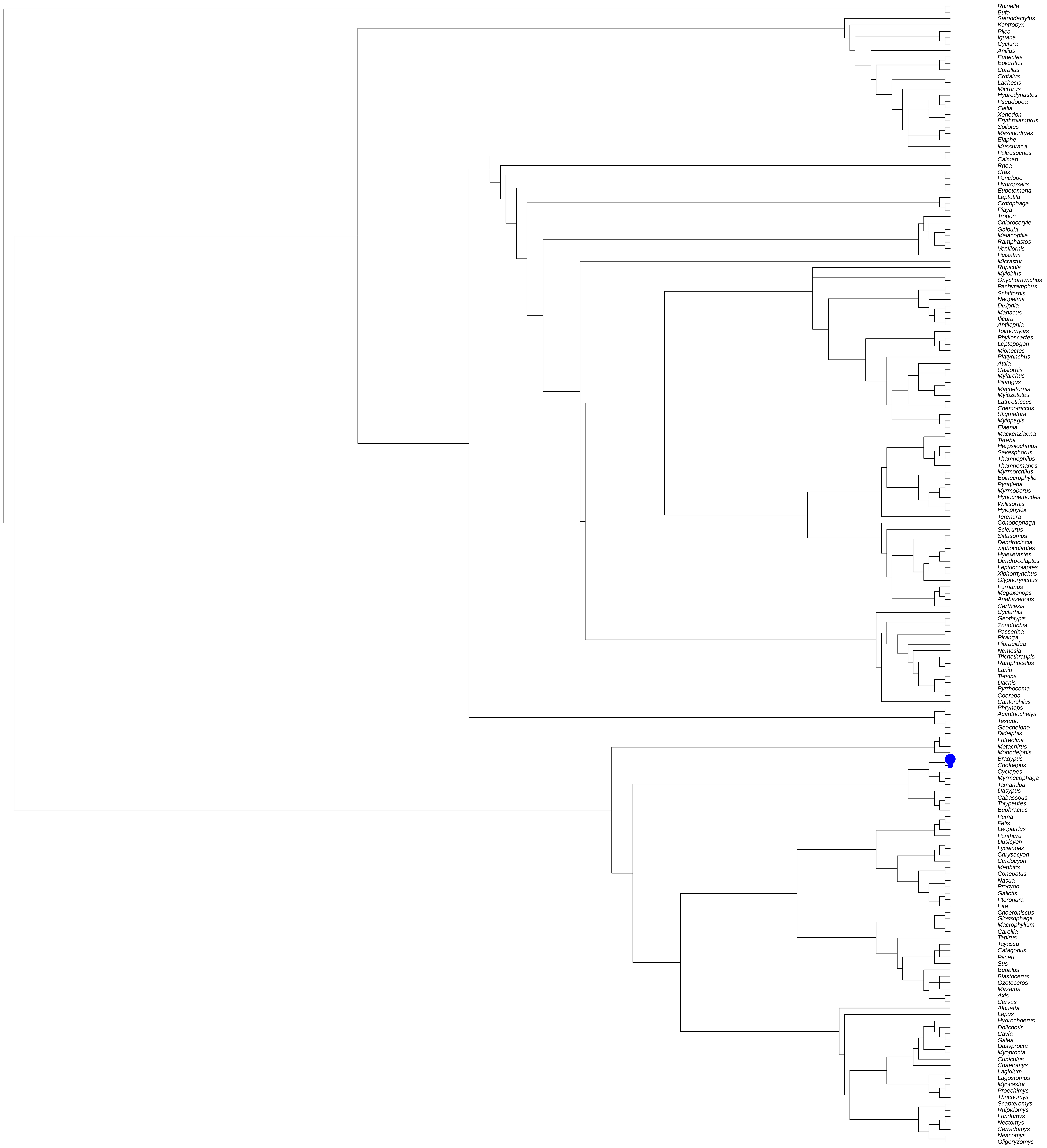

Amblyomma varium nymph

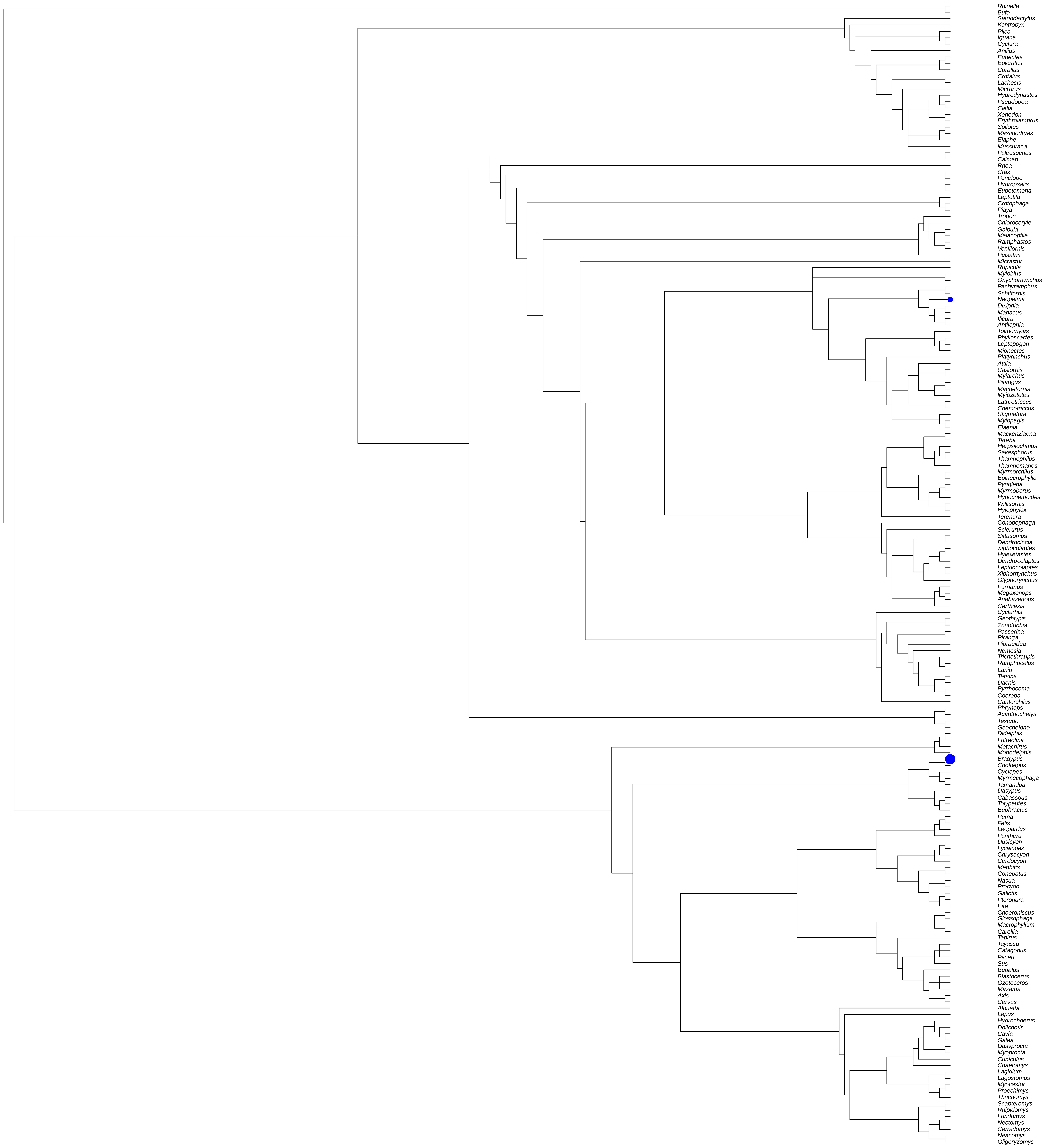

Haemaphysalis juxtakochi adult

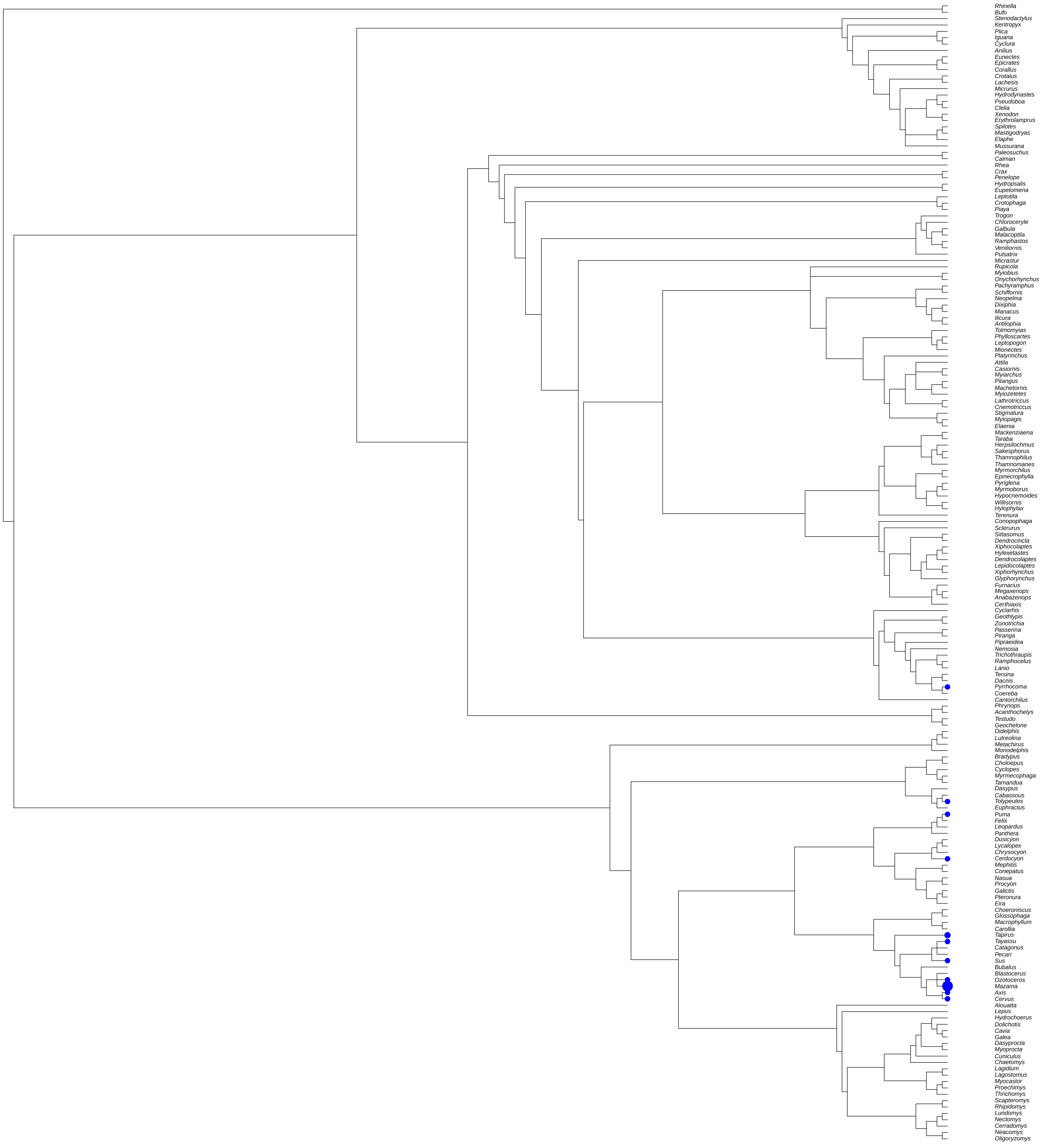

Haemaphysalis juxtakochi larva

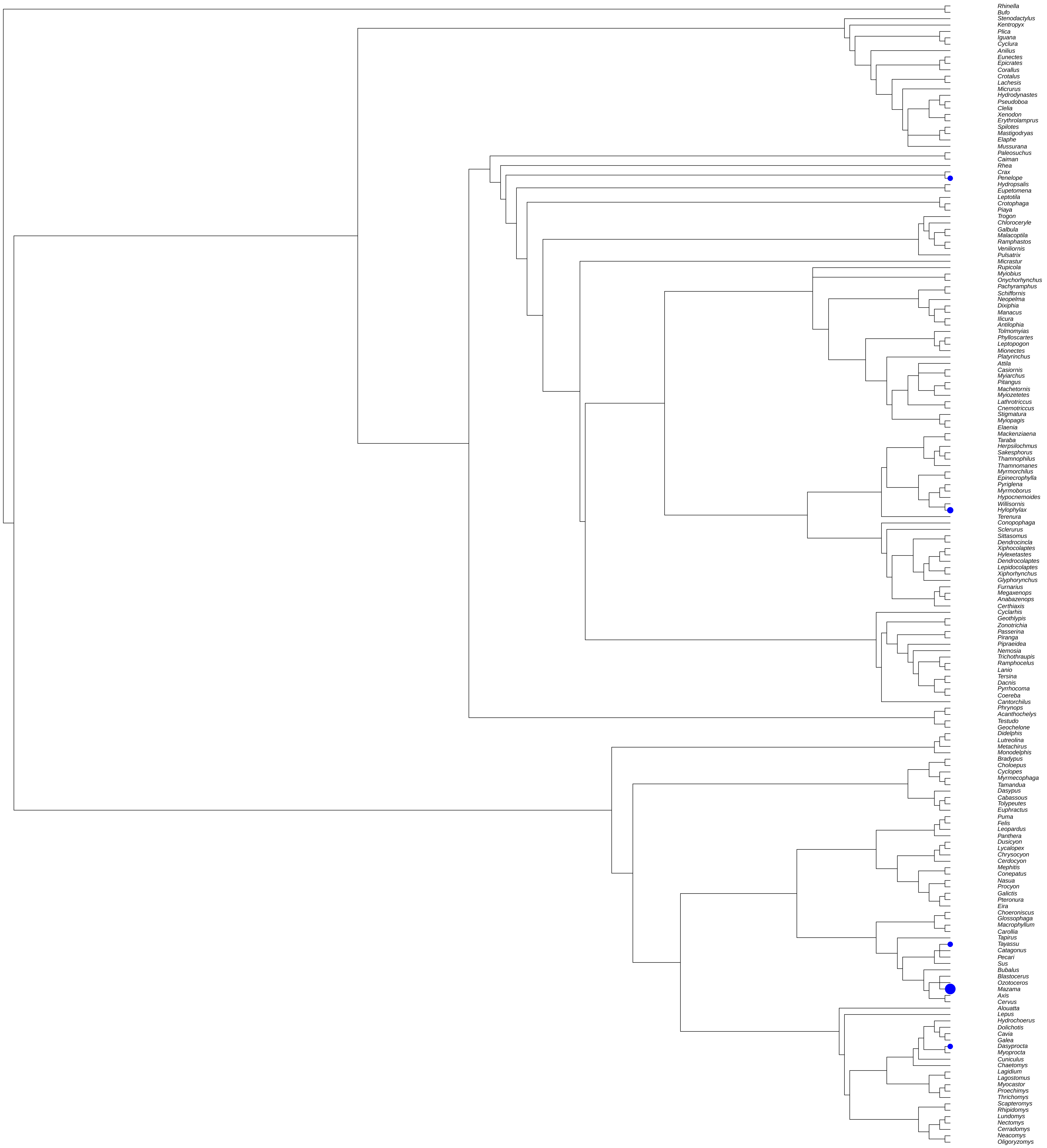

Haemaphysalis juxtakochi nymph

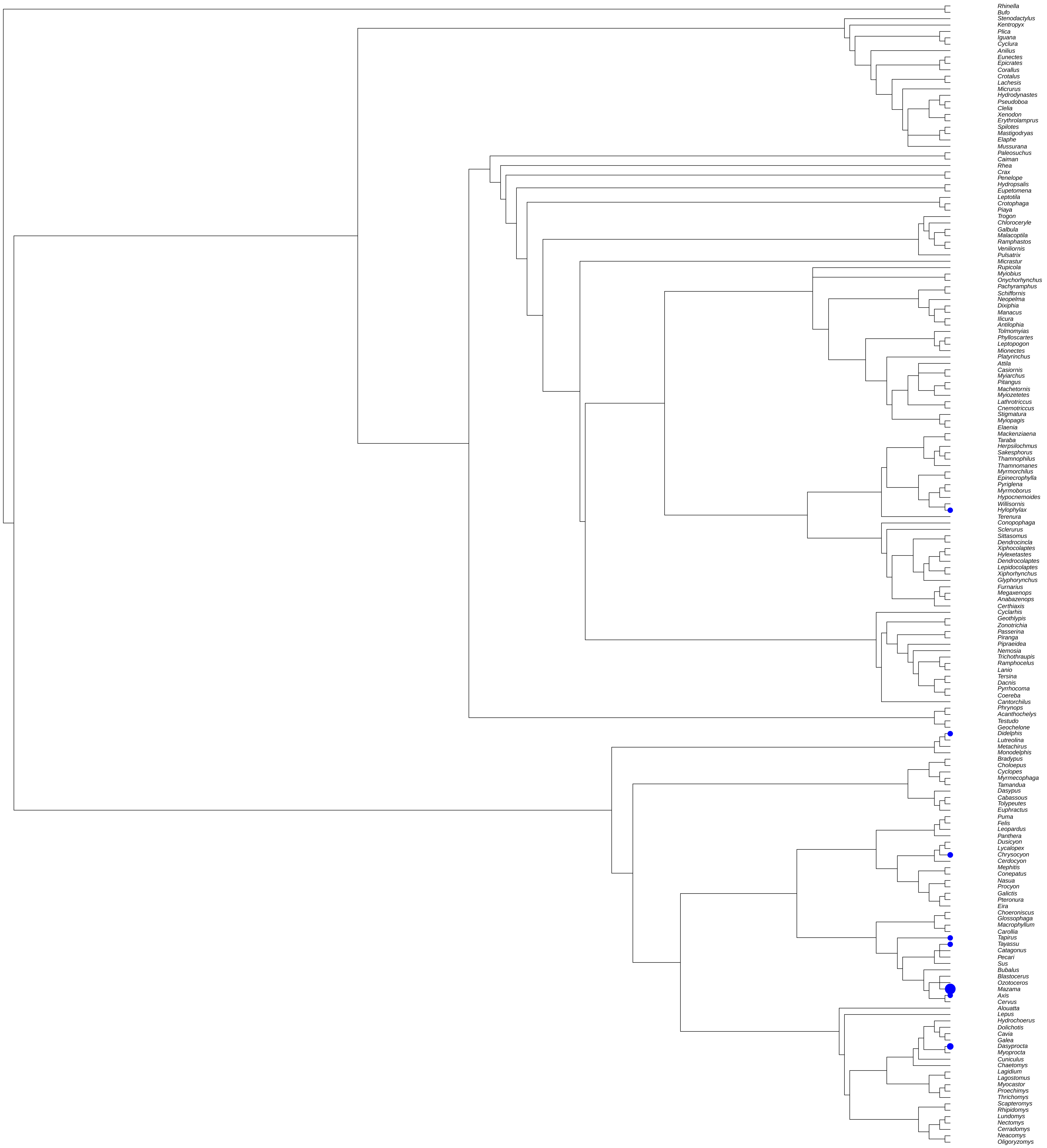

Haemaphysalis leporispalustris adult

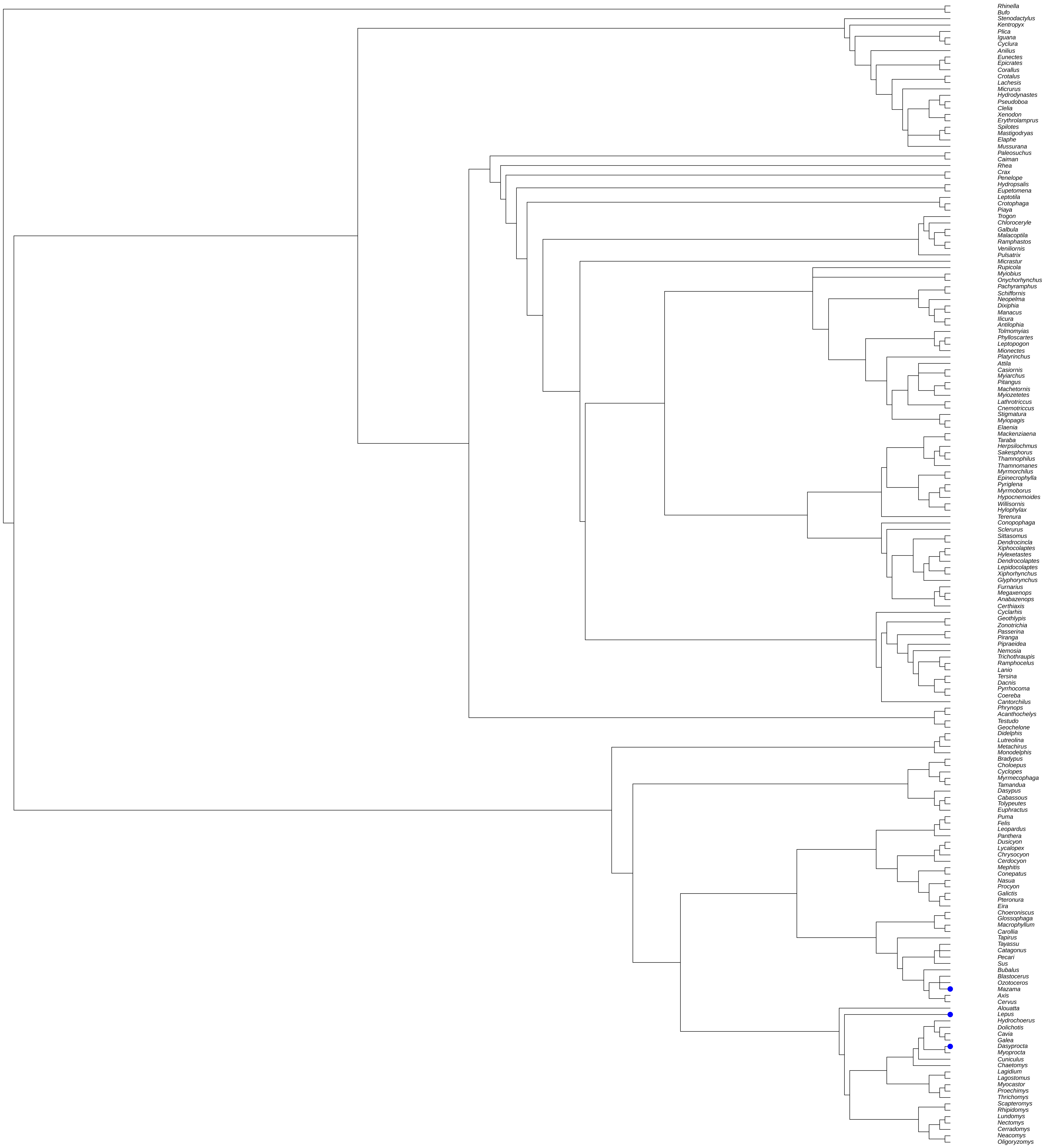

Haemaphysalis leporispalustris larva

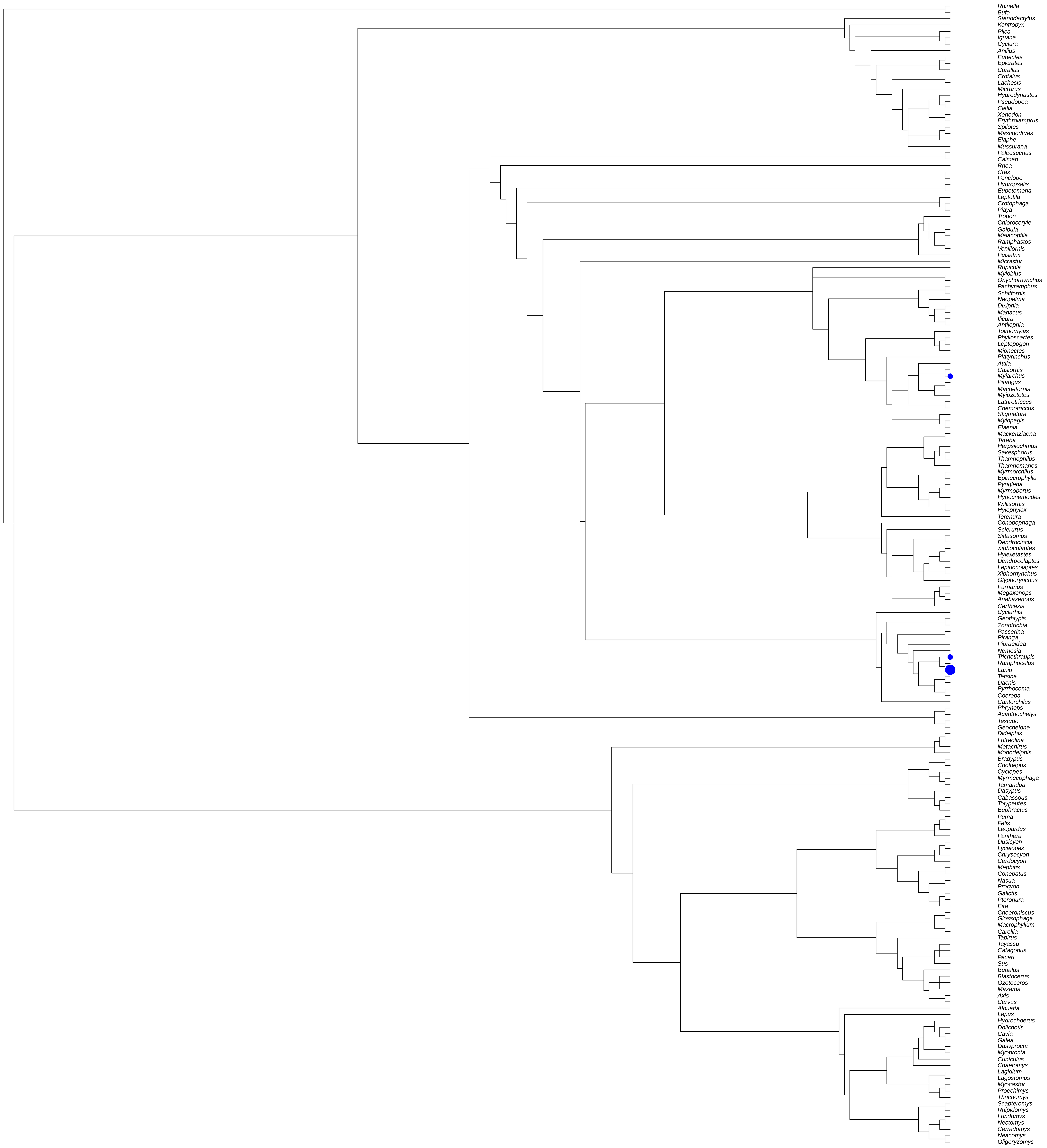

Haemaphysalis leporispalustris nymph

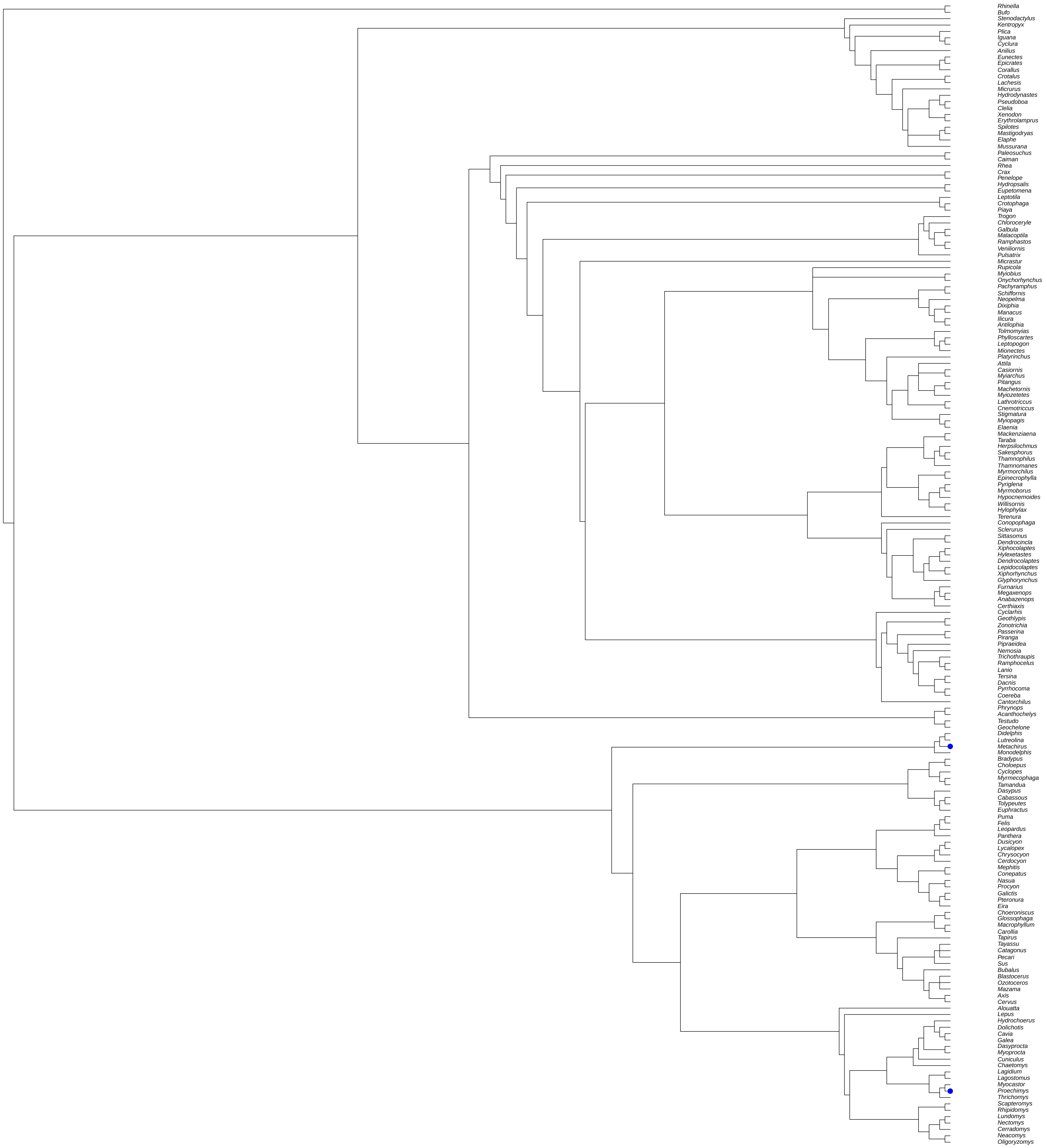

Ixodes amarali adult

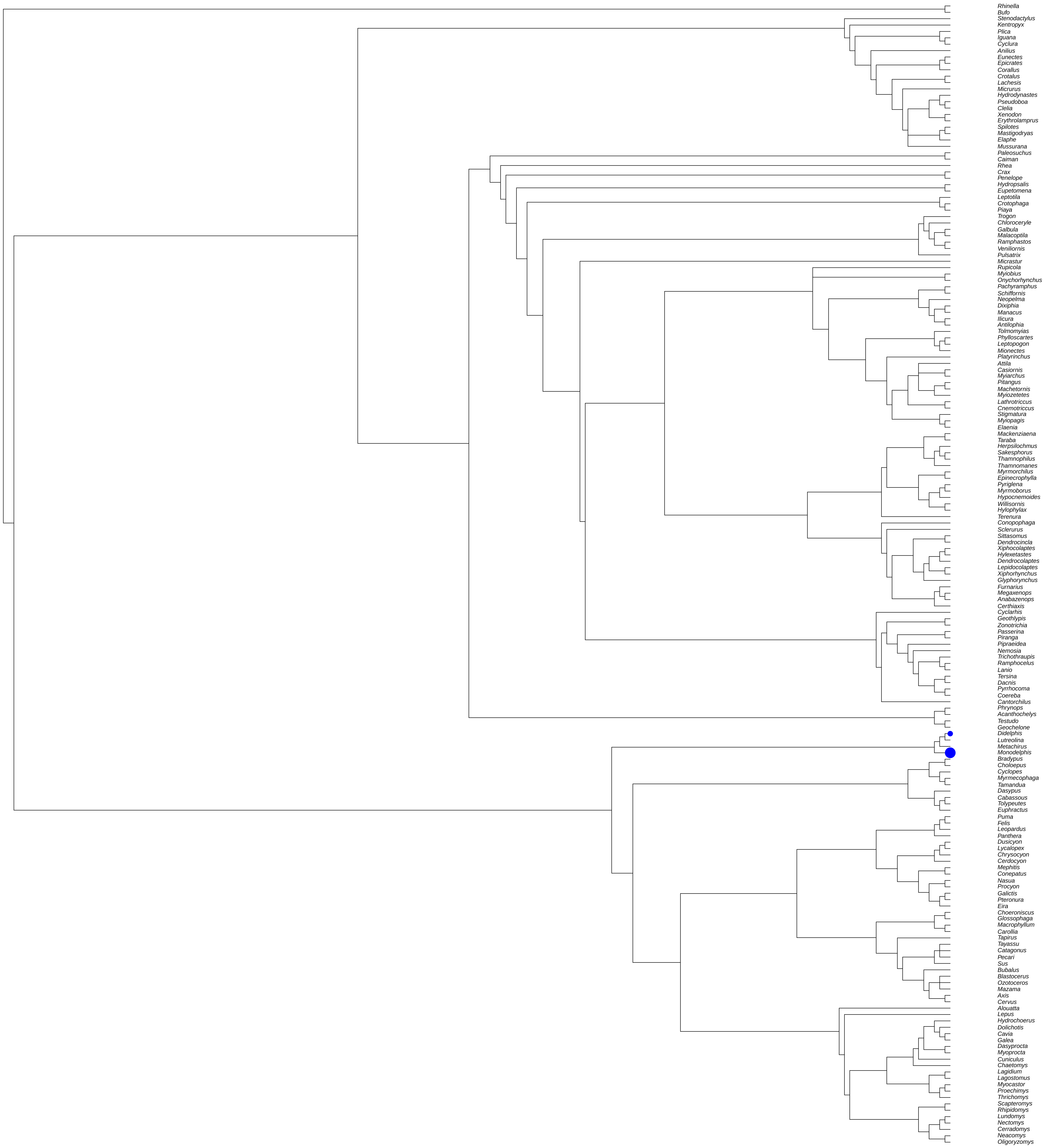

Ixodes amarali larva

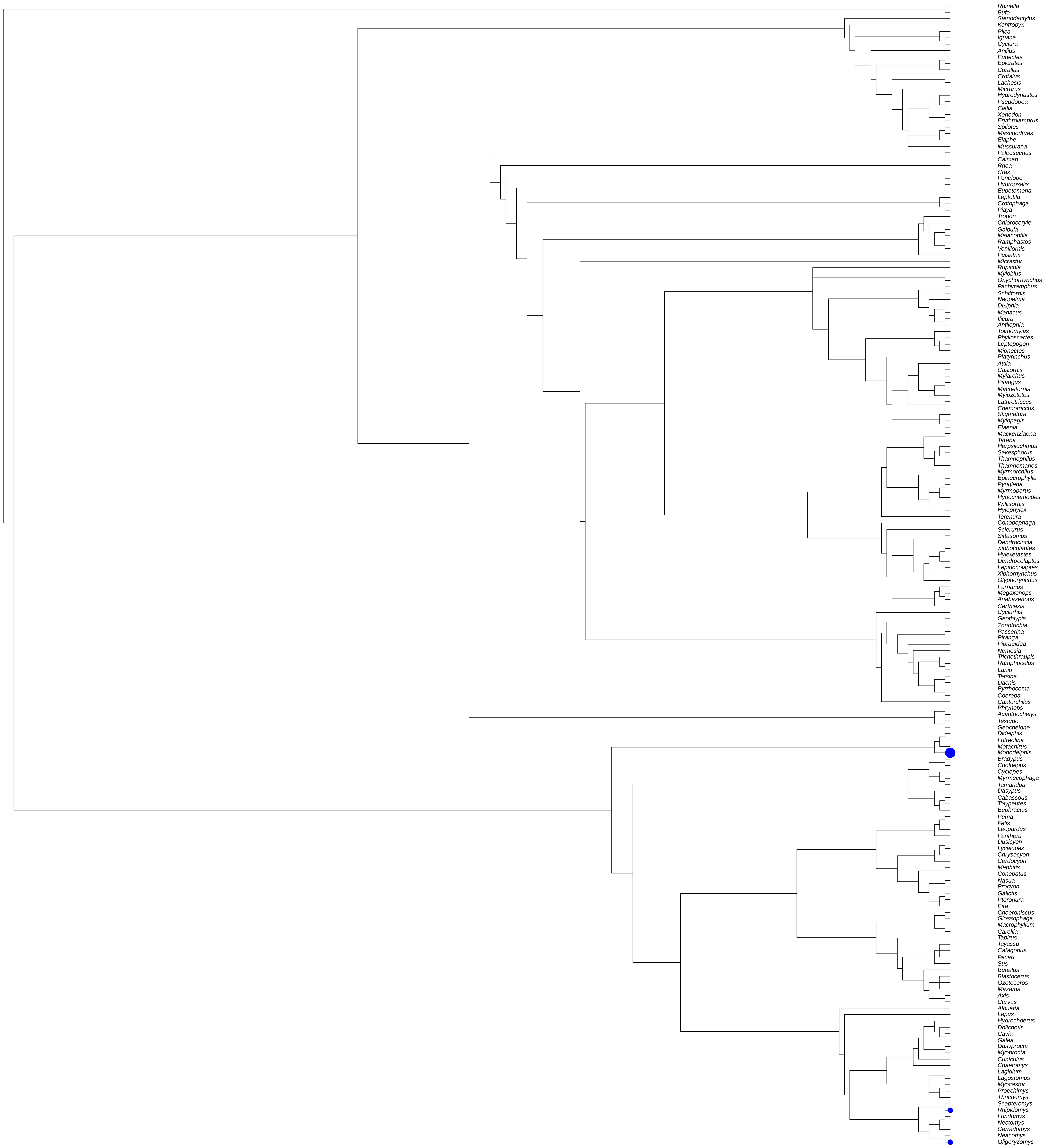

Ixodes amarali nymph

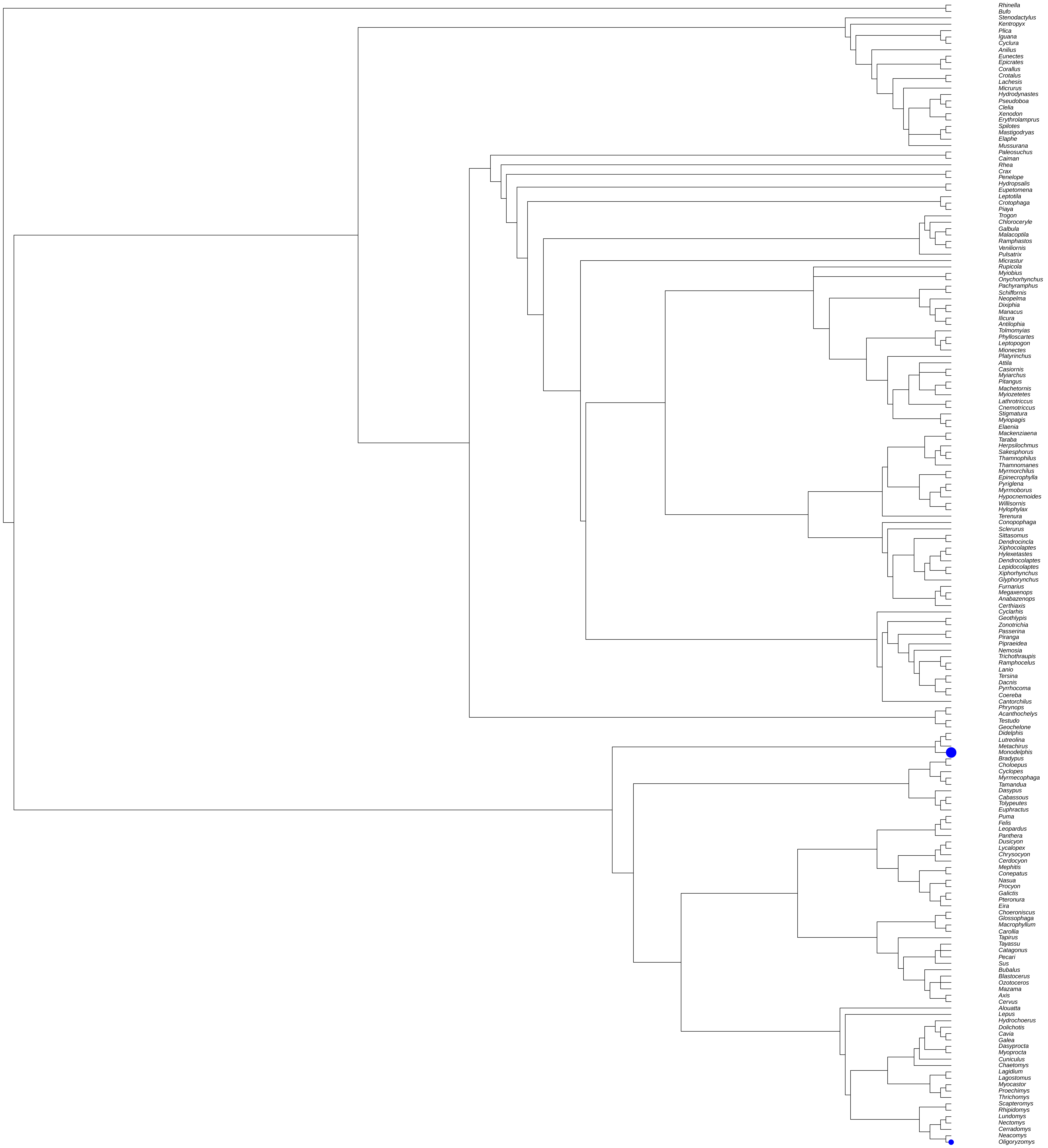

Ixodes aragaoi adult

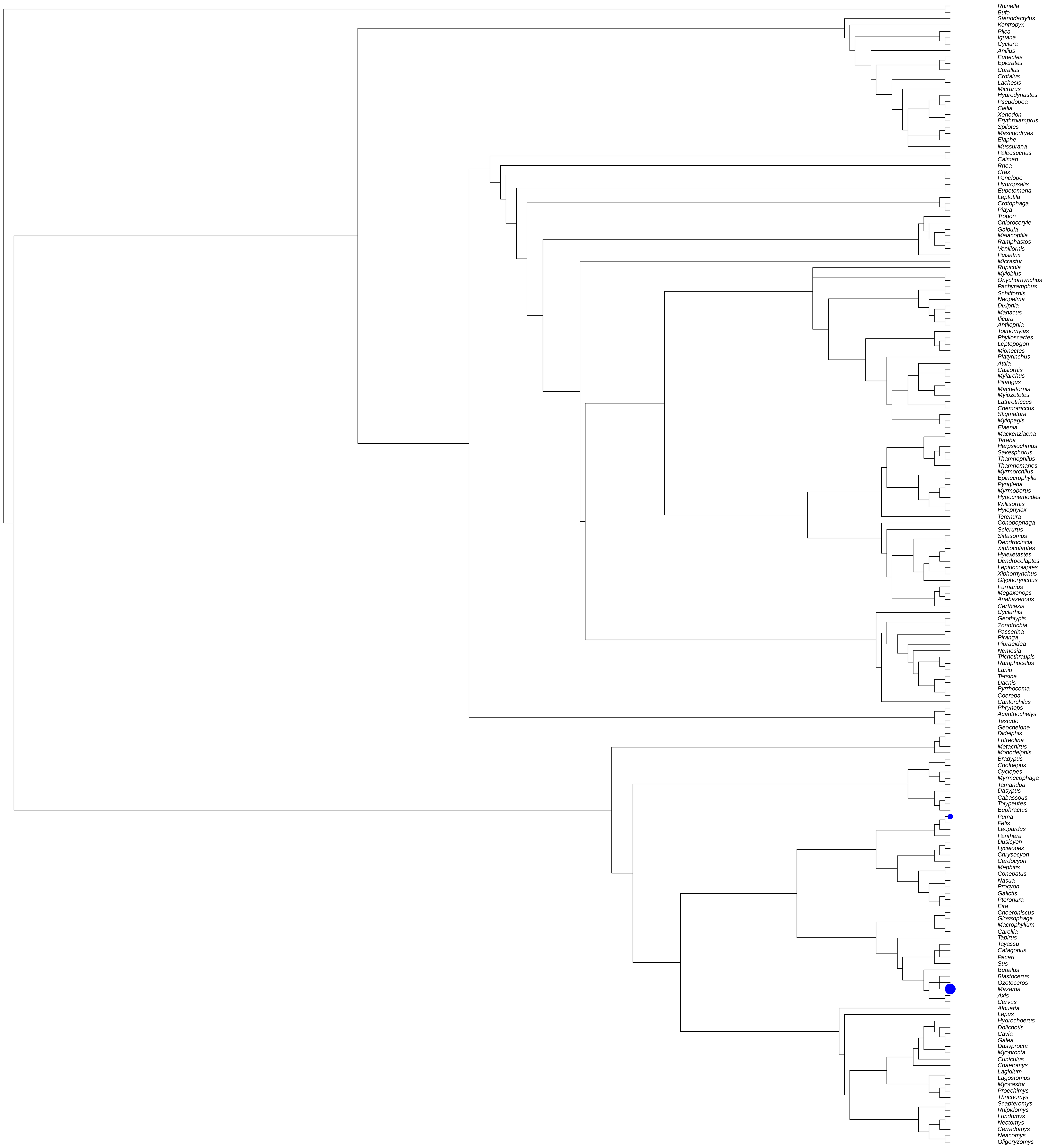

Ixodes aragaoi nymph

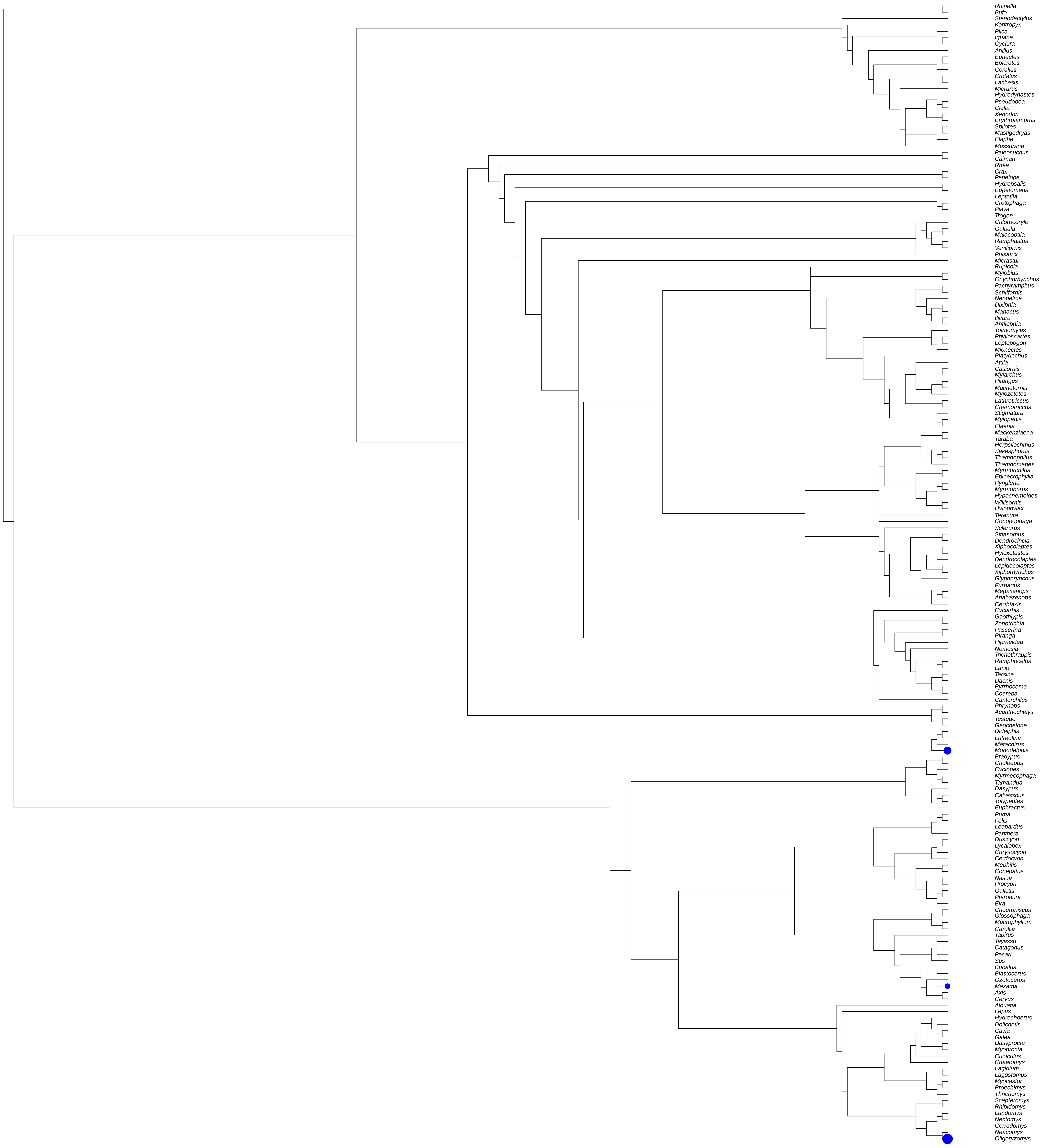

Ixodes auritulus adult

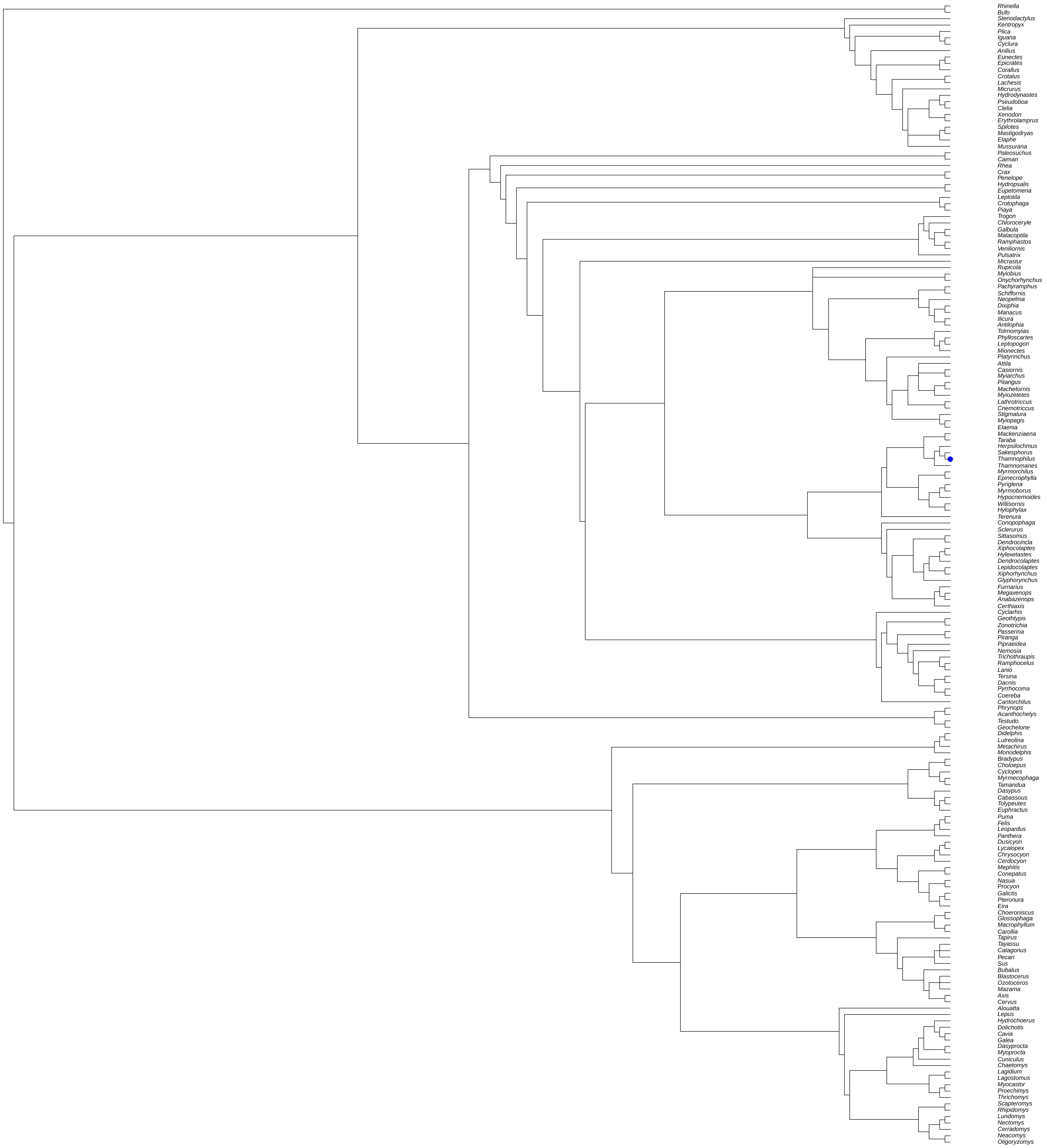

Ixodes auritulus larva

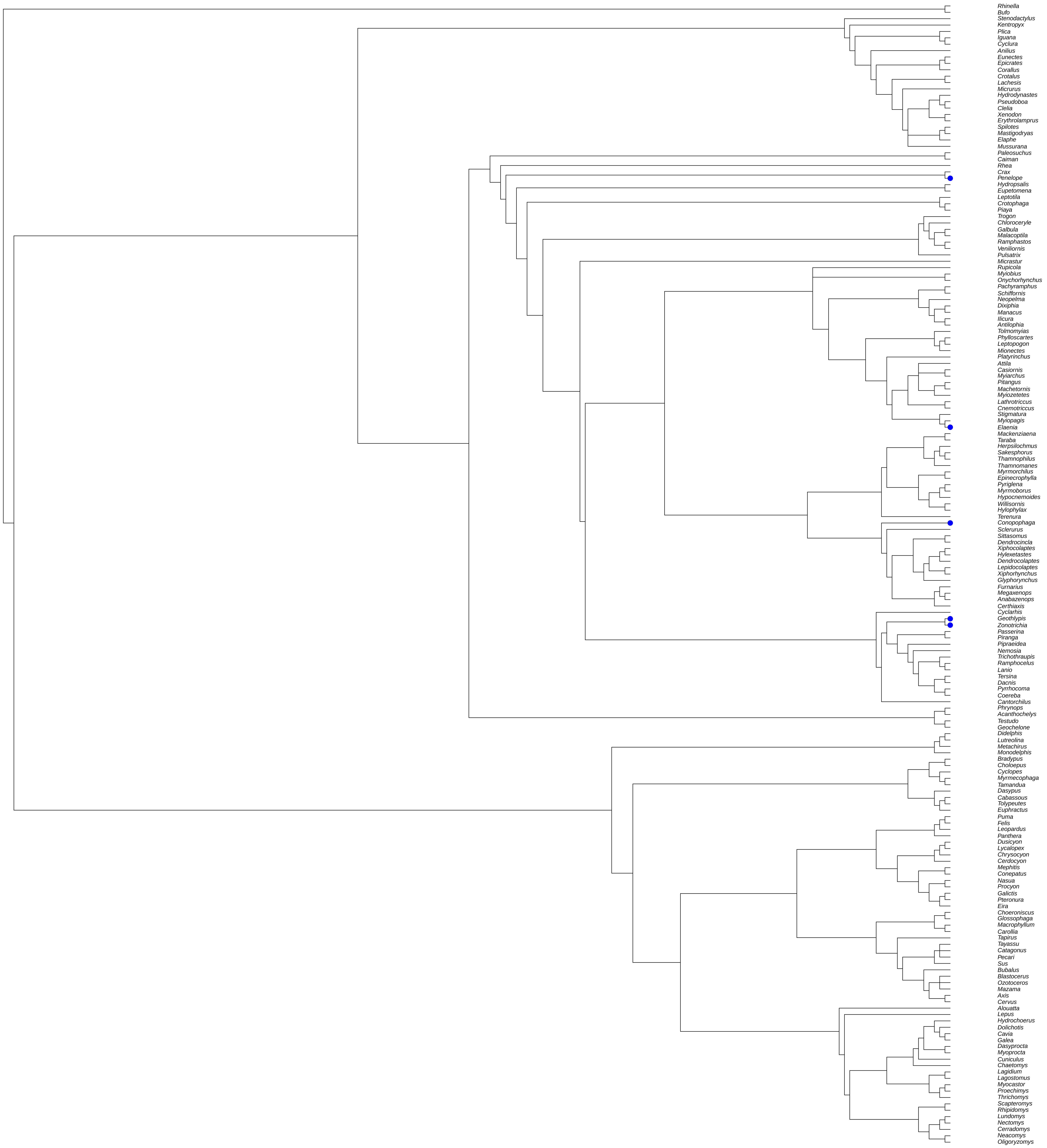

Ixodes auritulus nymph

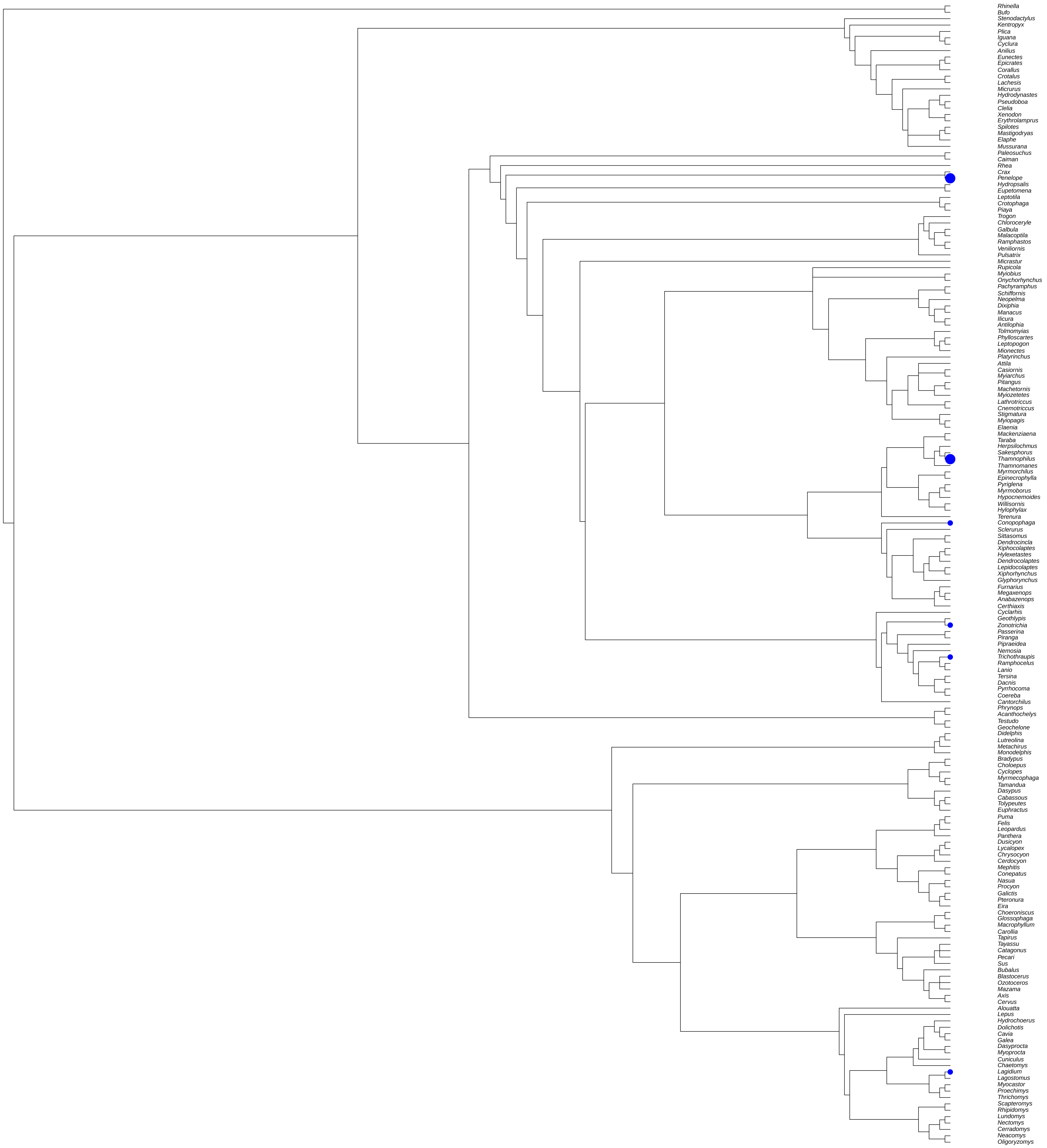

Ixodes fossulatus adult

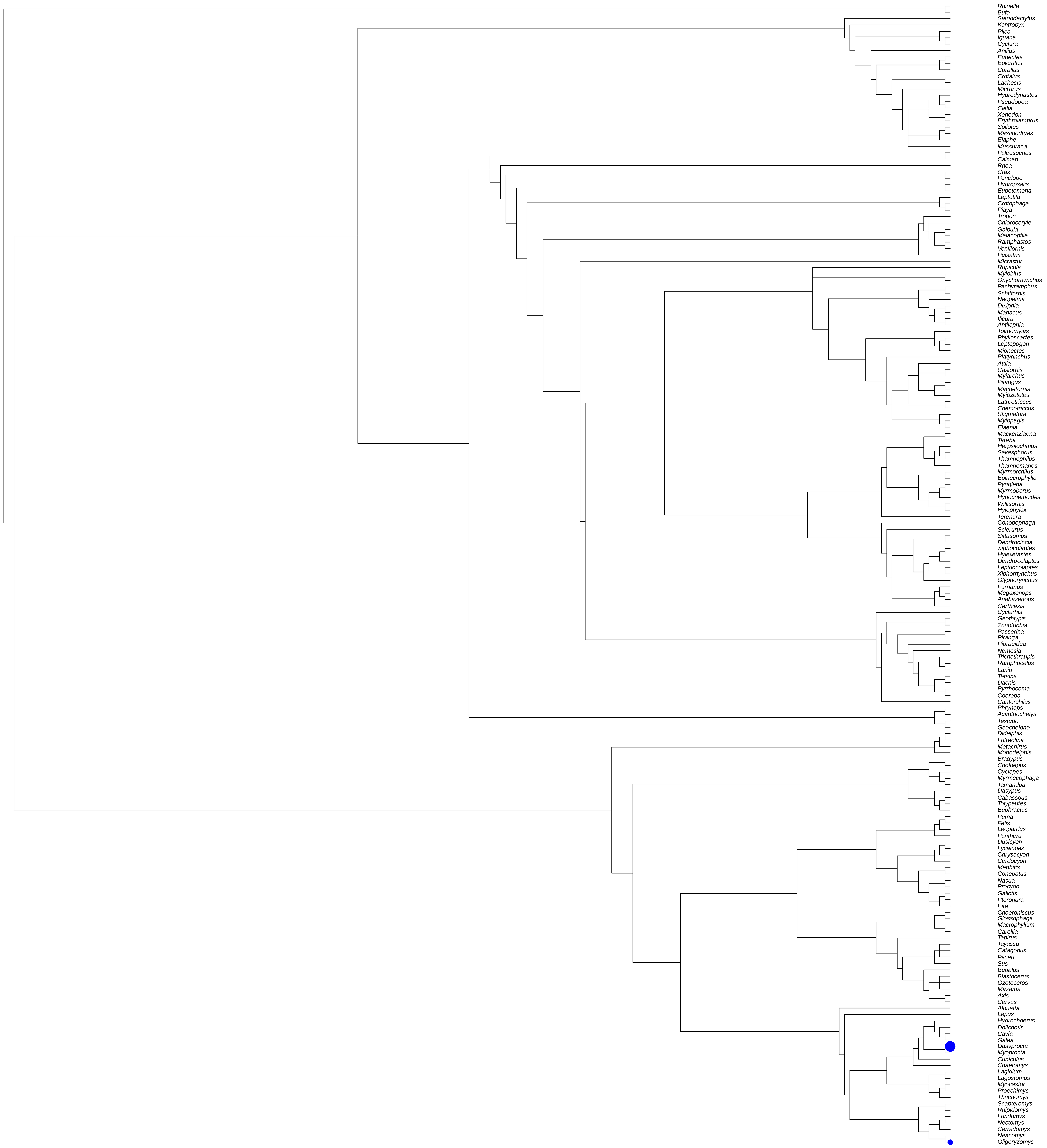

Ixodes fossulatus larva

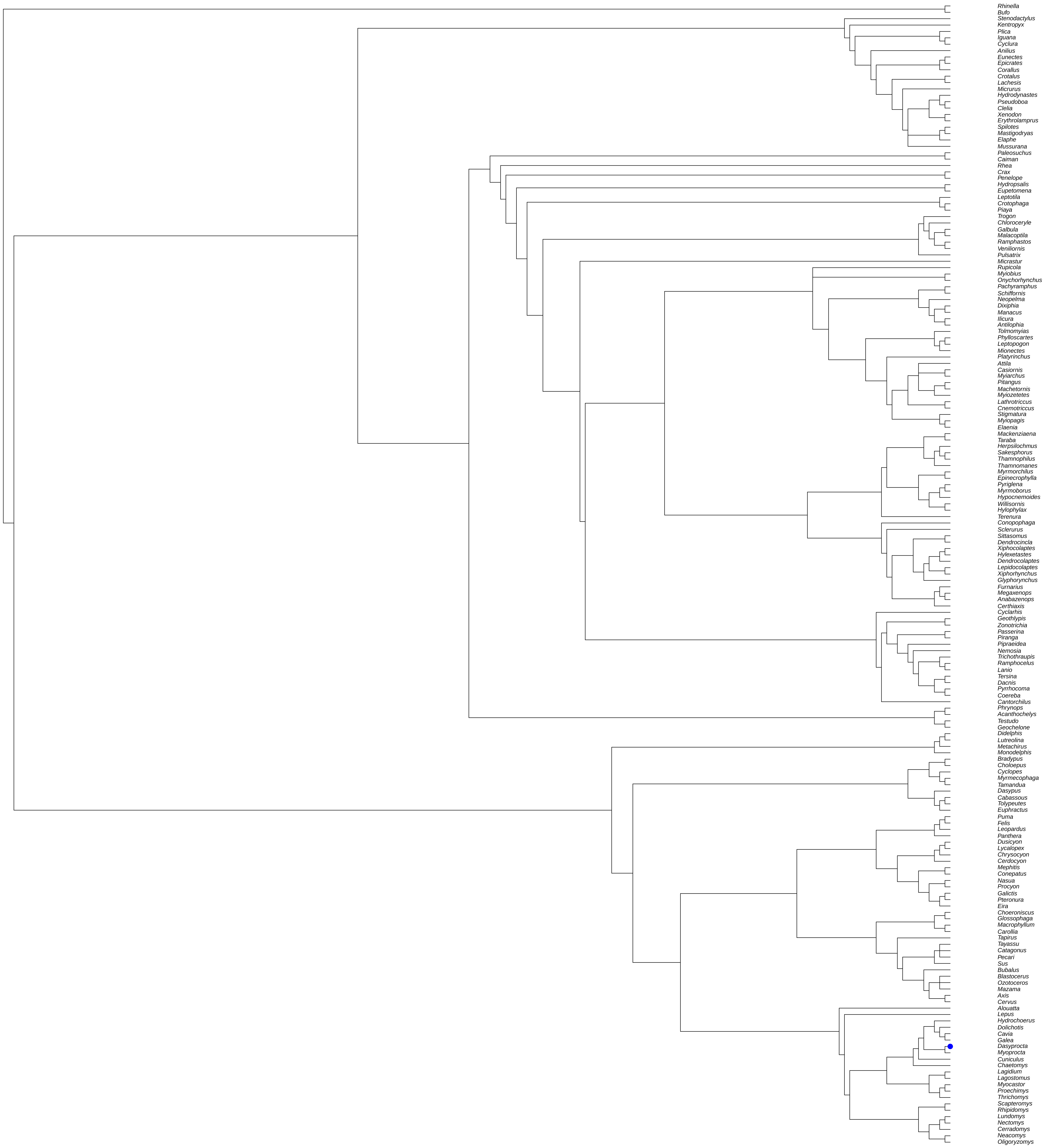

Ixodes fossulatus adult

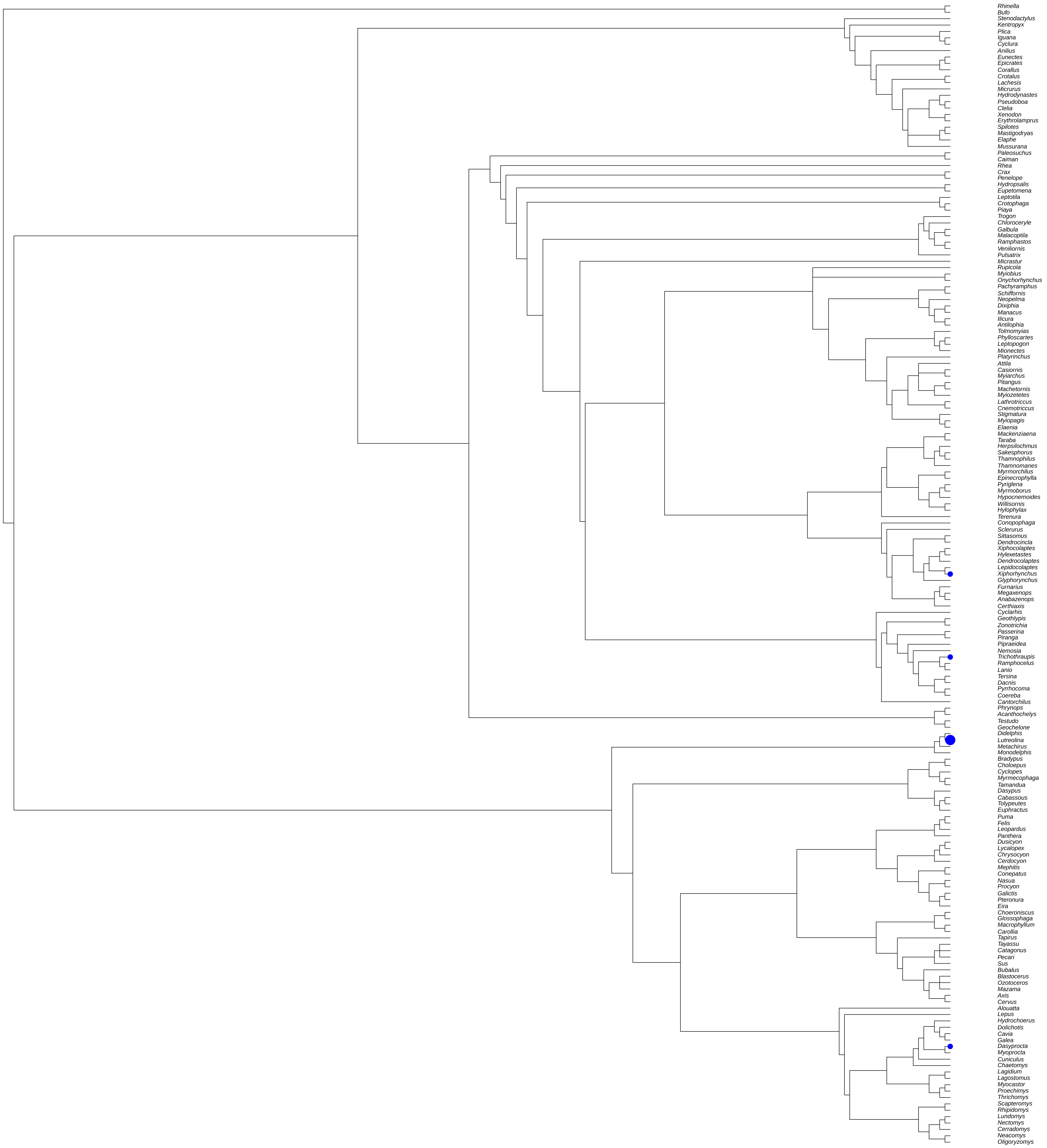

Ixodes longiscutatus adult

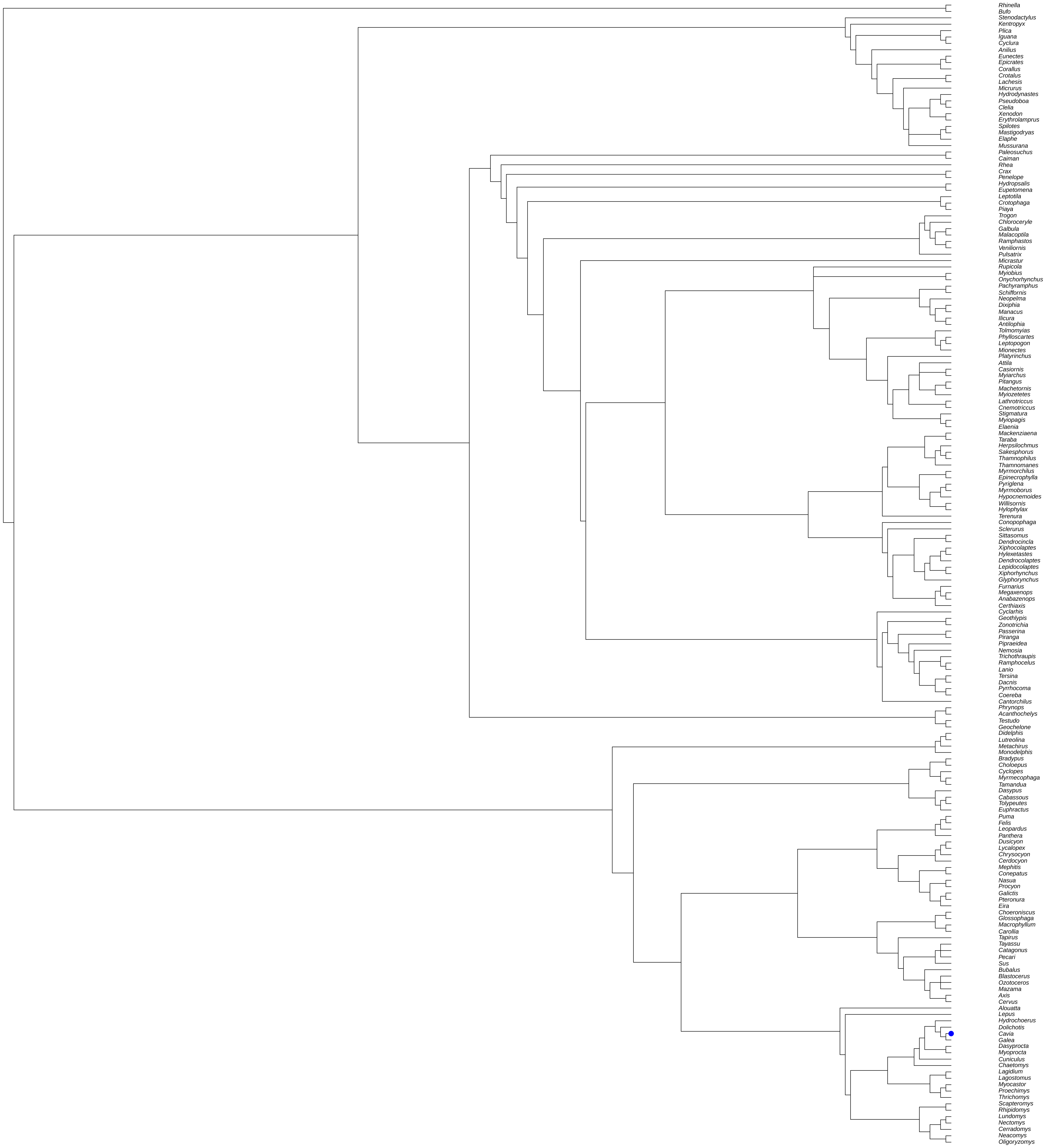

Ixodes longiscutatus larva

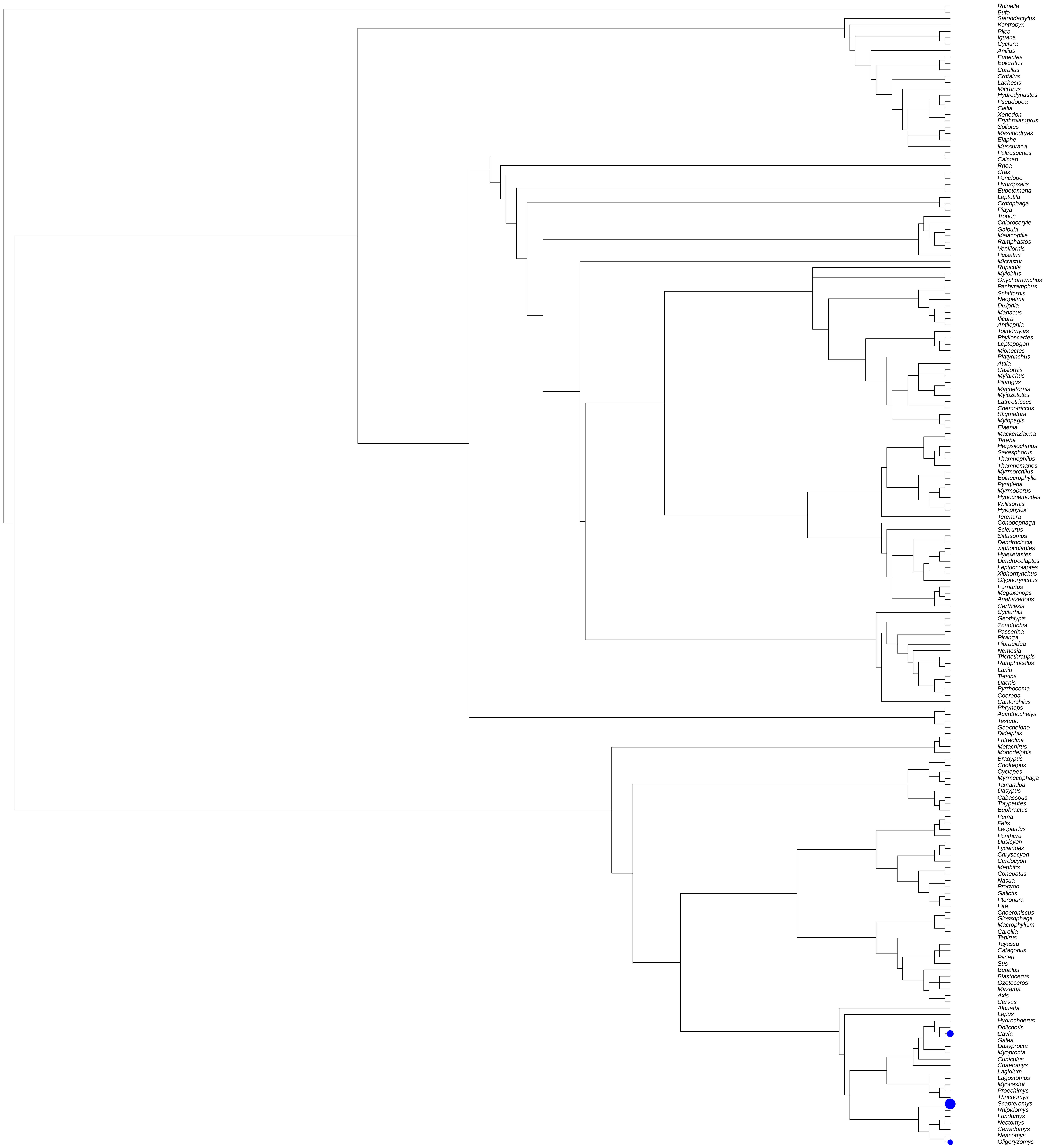

Ixodes longiscutatus nymph

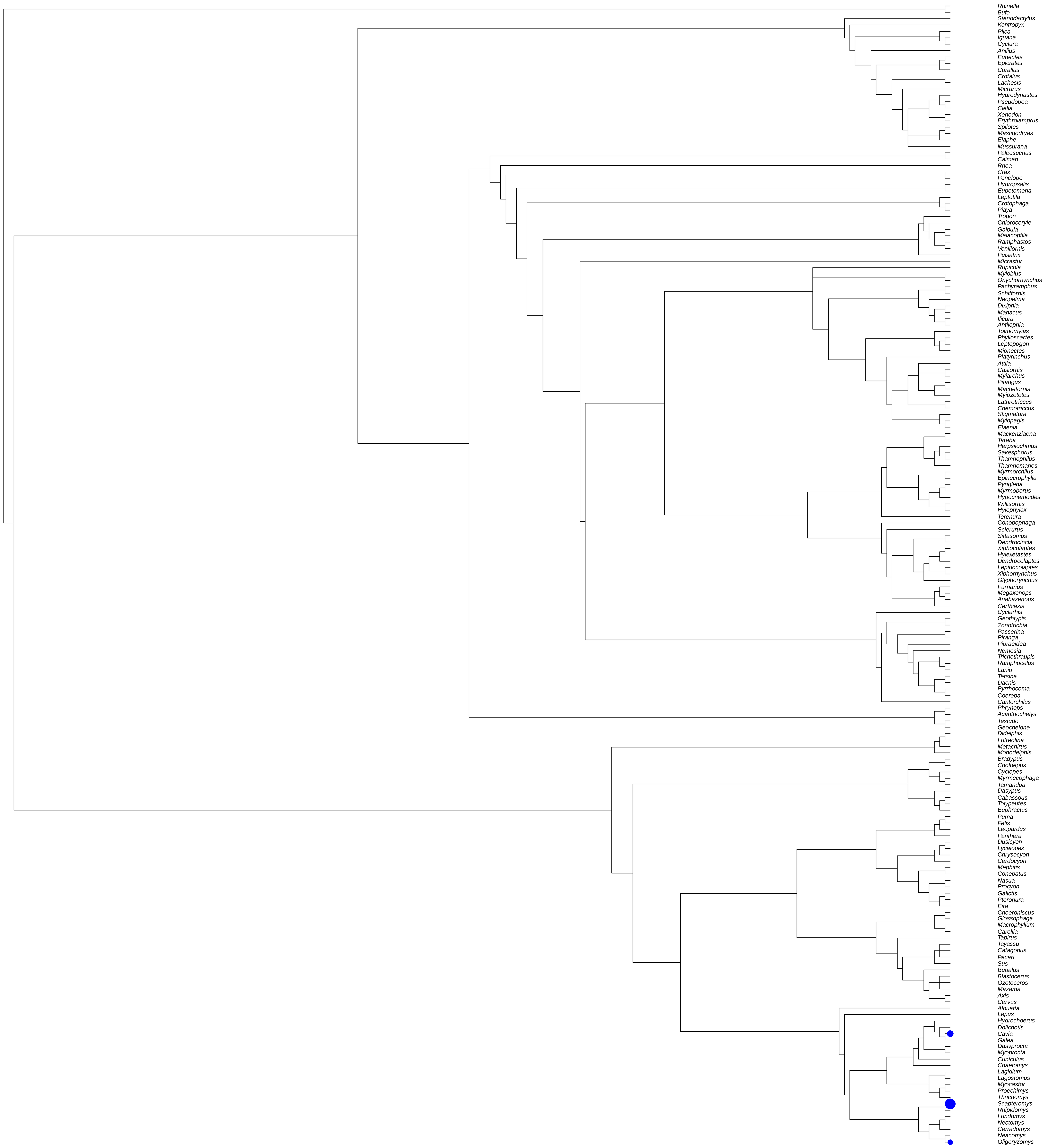

Ixodes loricatus adult

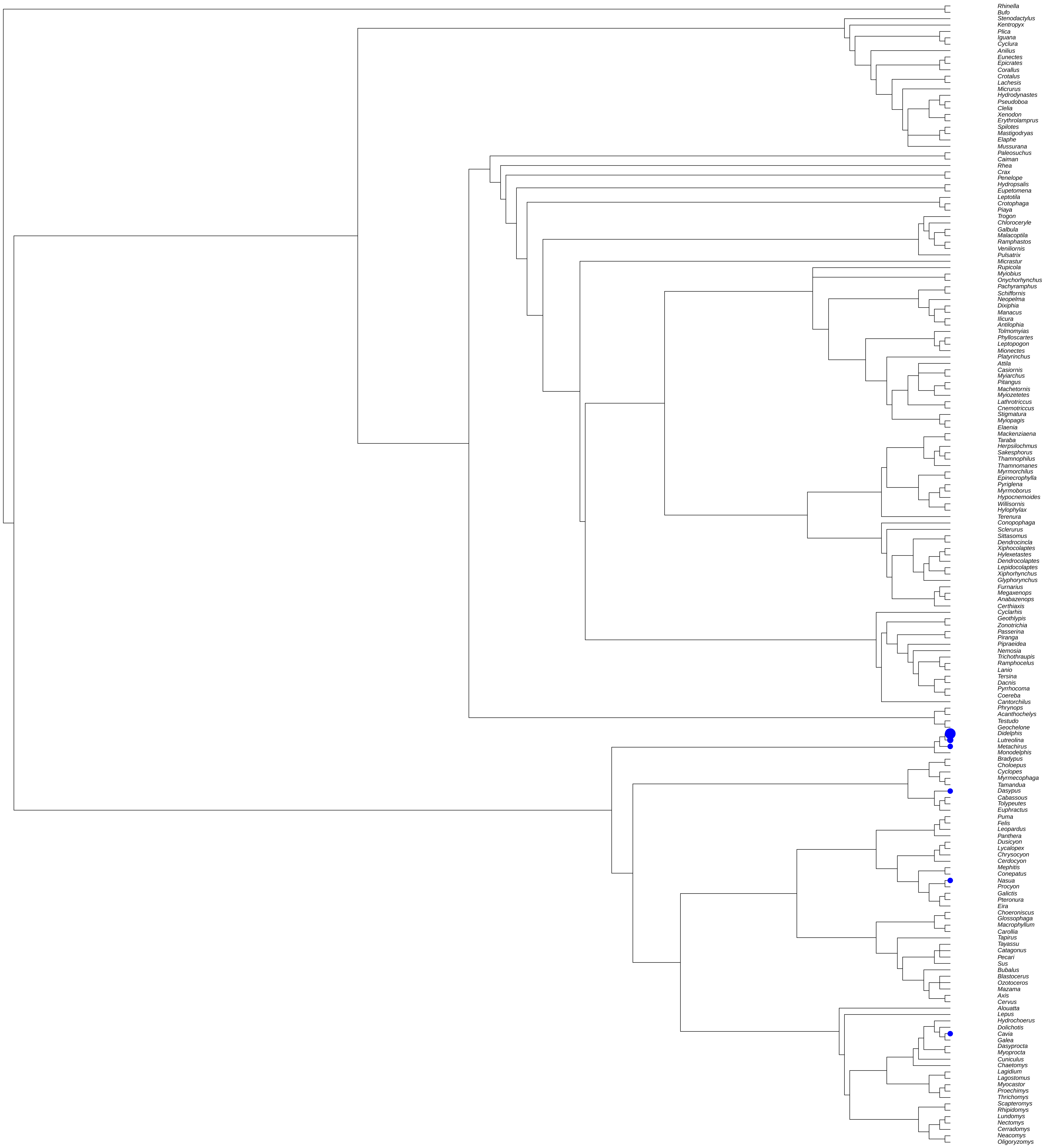

Ixodes loricatus larva

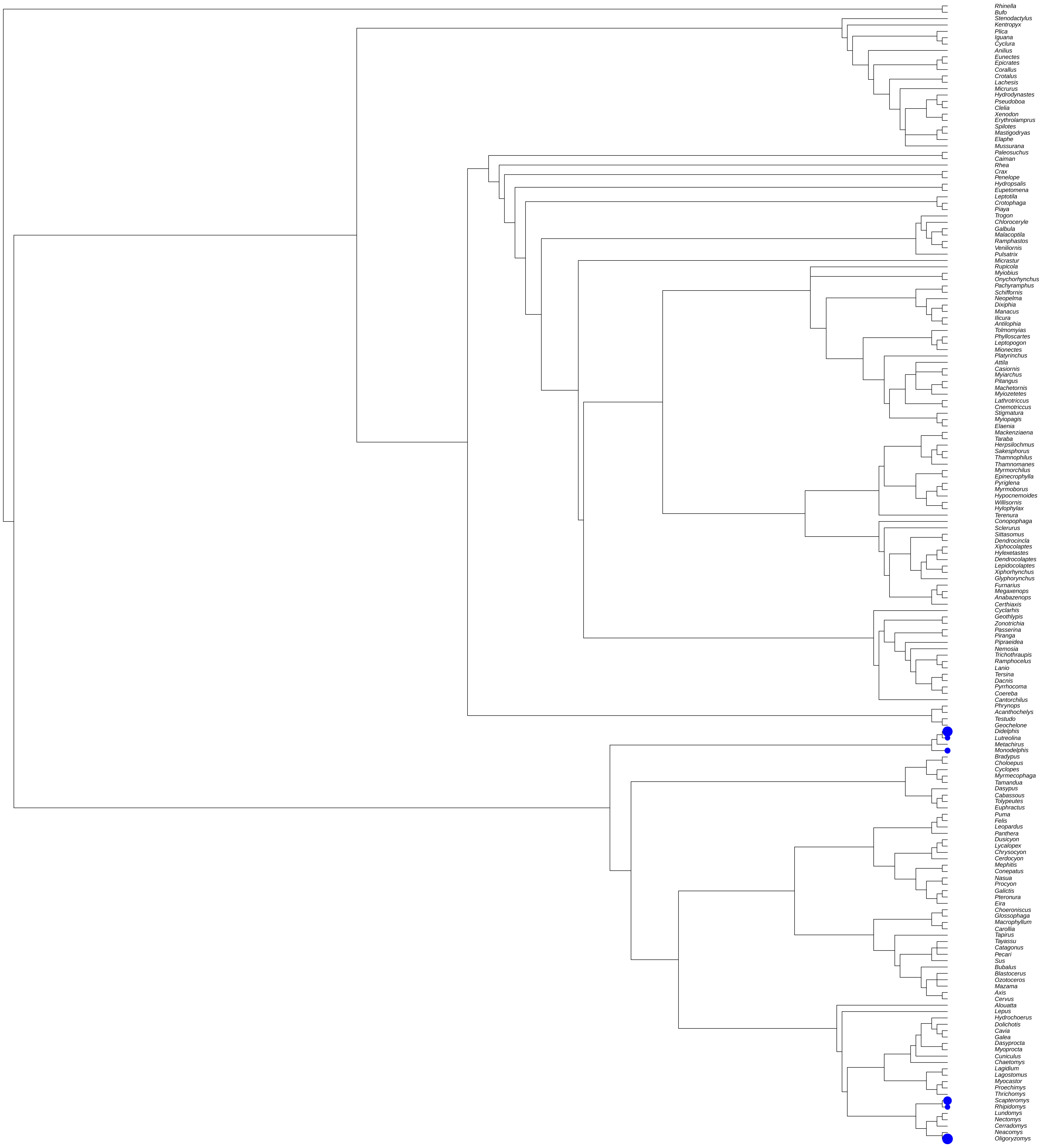

Ixodes loricatus adult

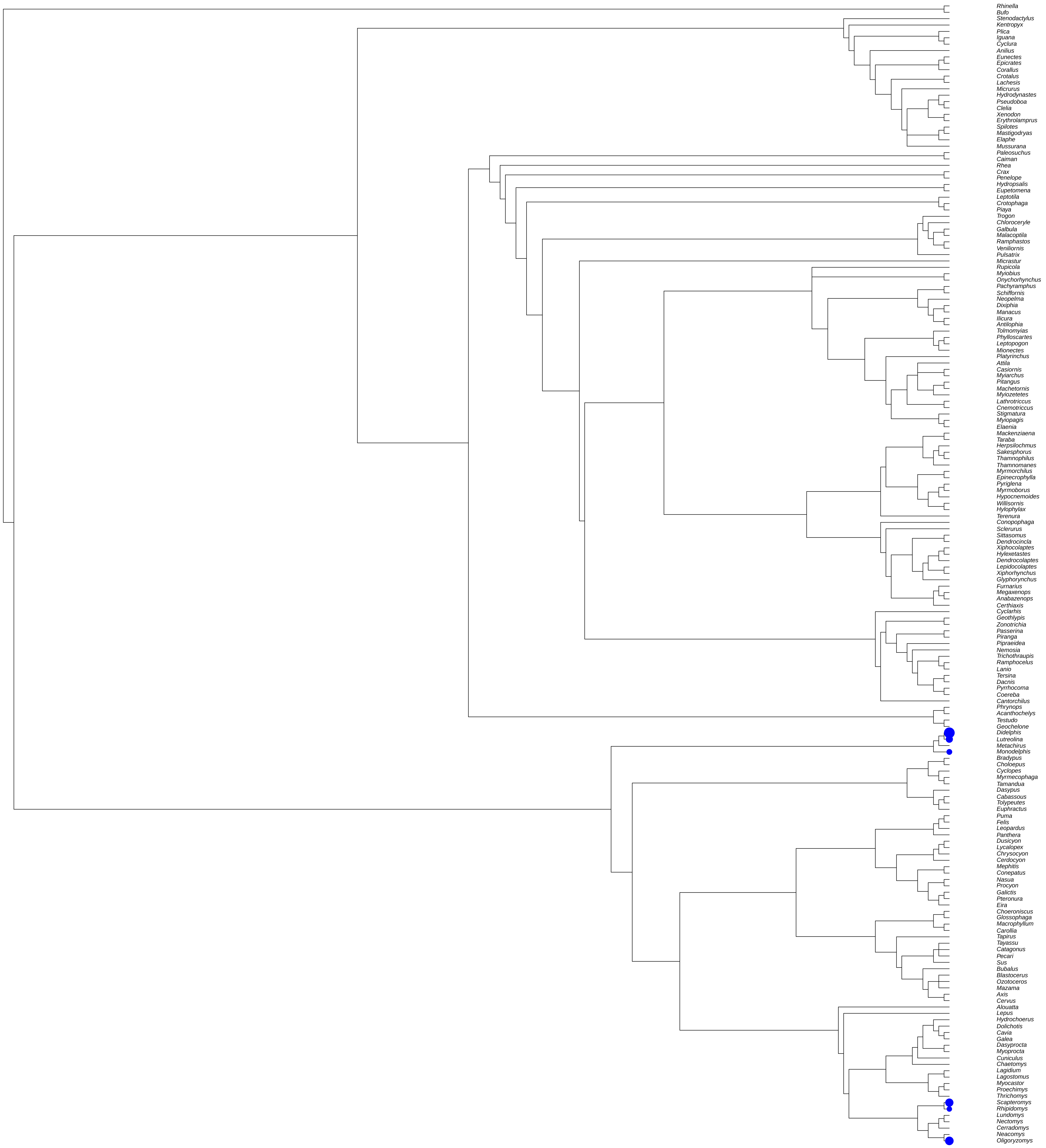

Ixodes loricatus nymph

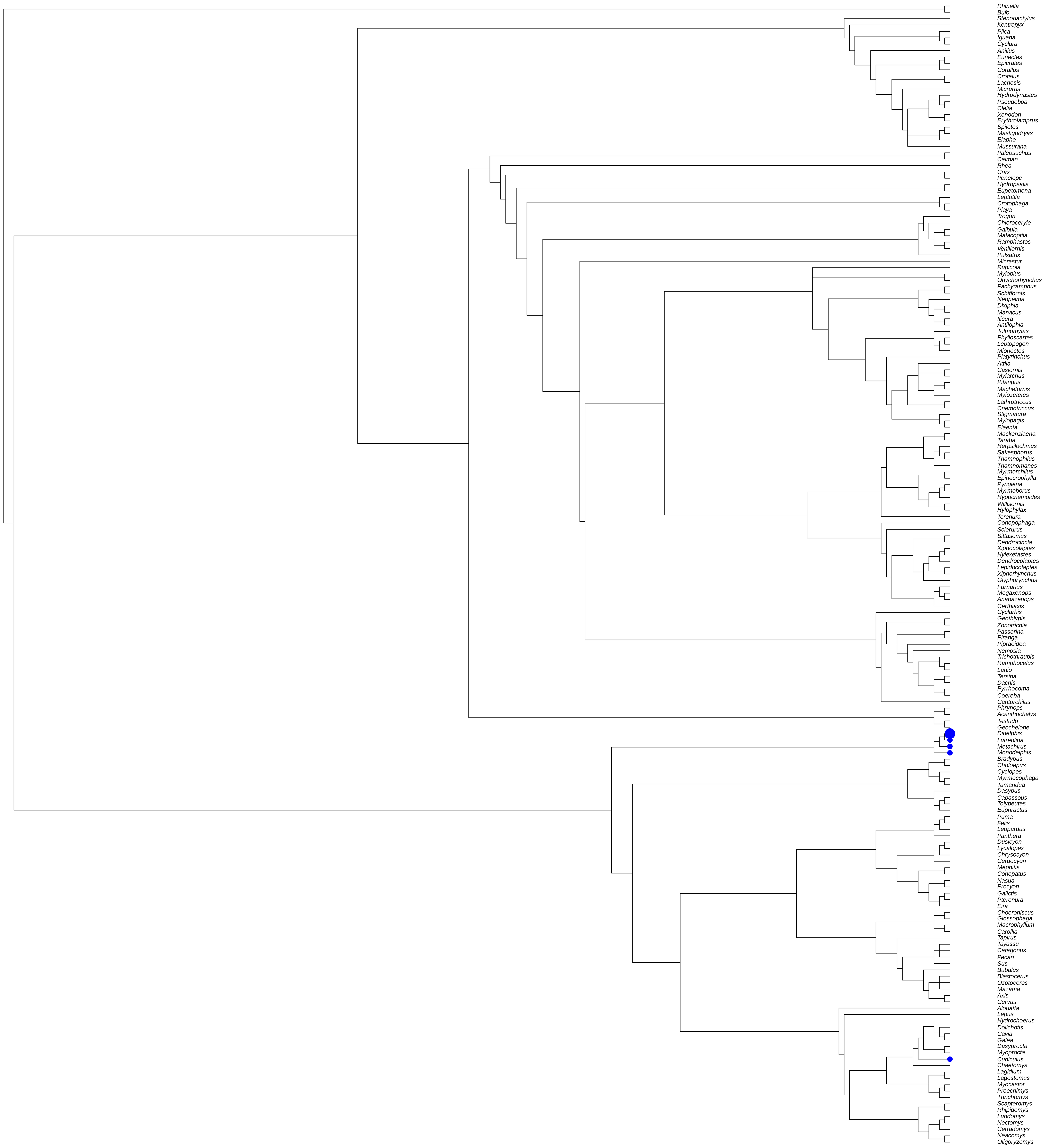

Ixodes luciae larva

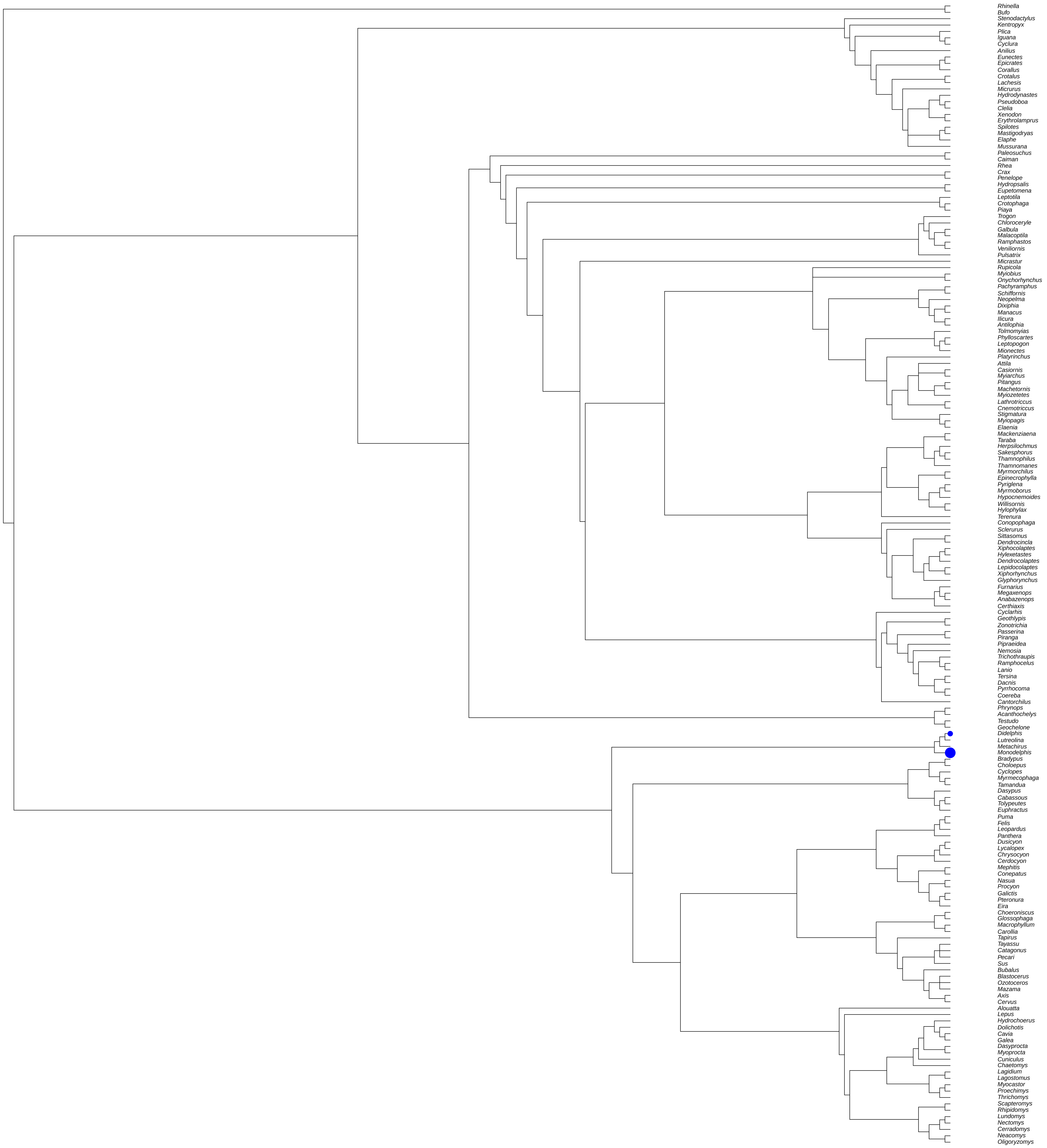

Ixodes luciae nymph

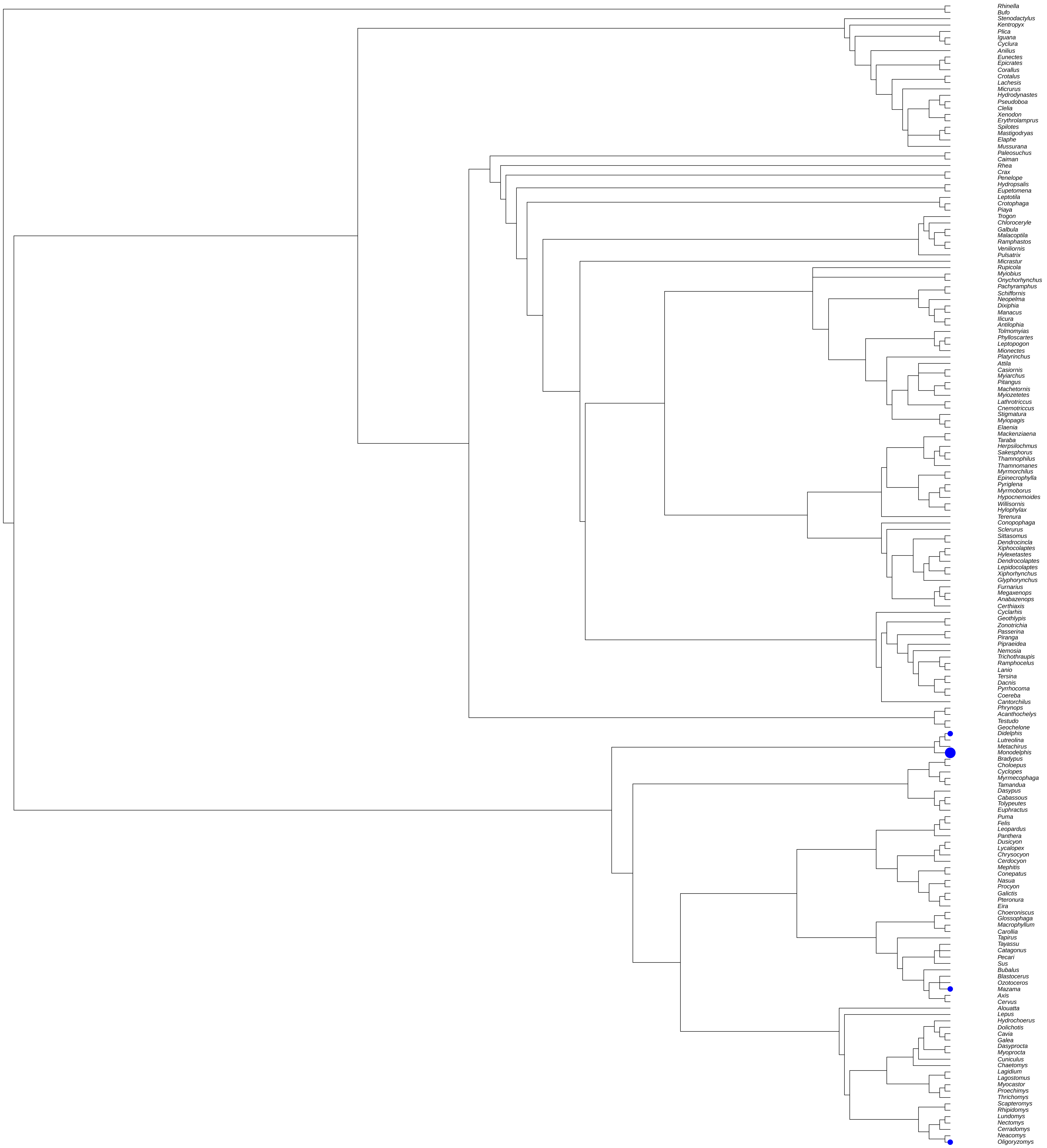

Ixodes pararicinus adult

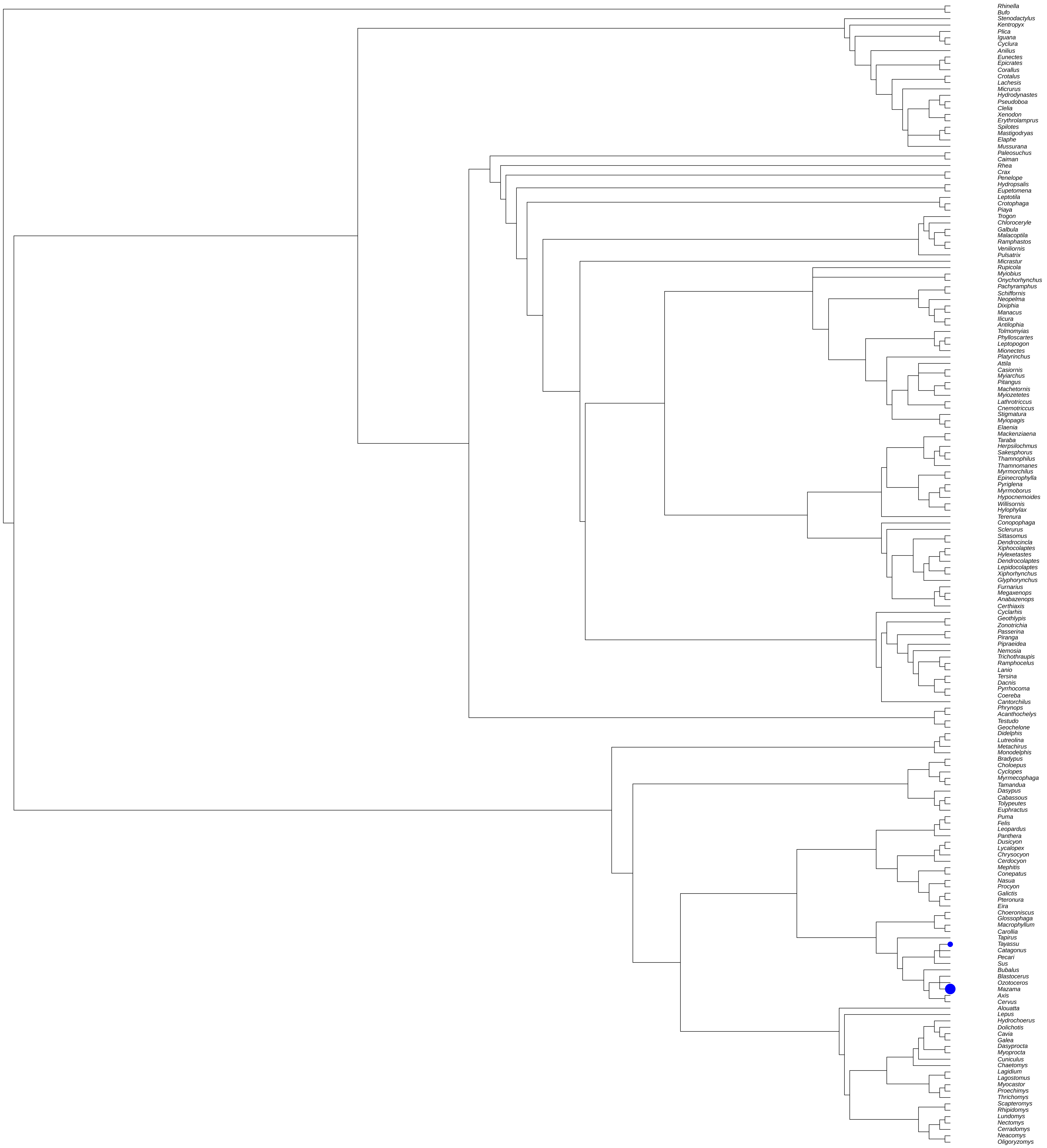

Ixodes pararicinus larva

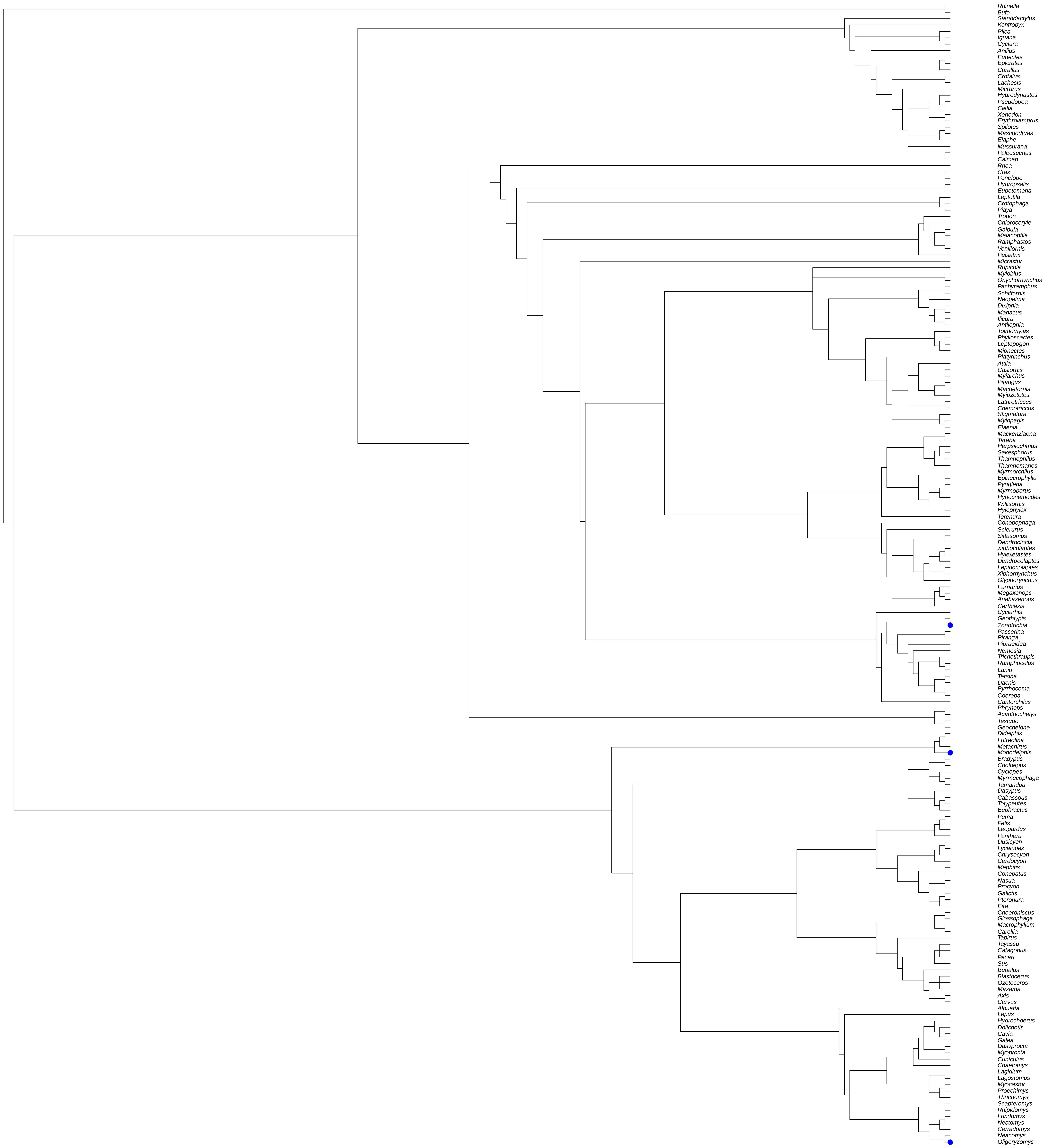

Ixodes sigelos adult

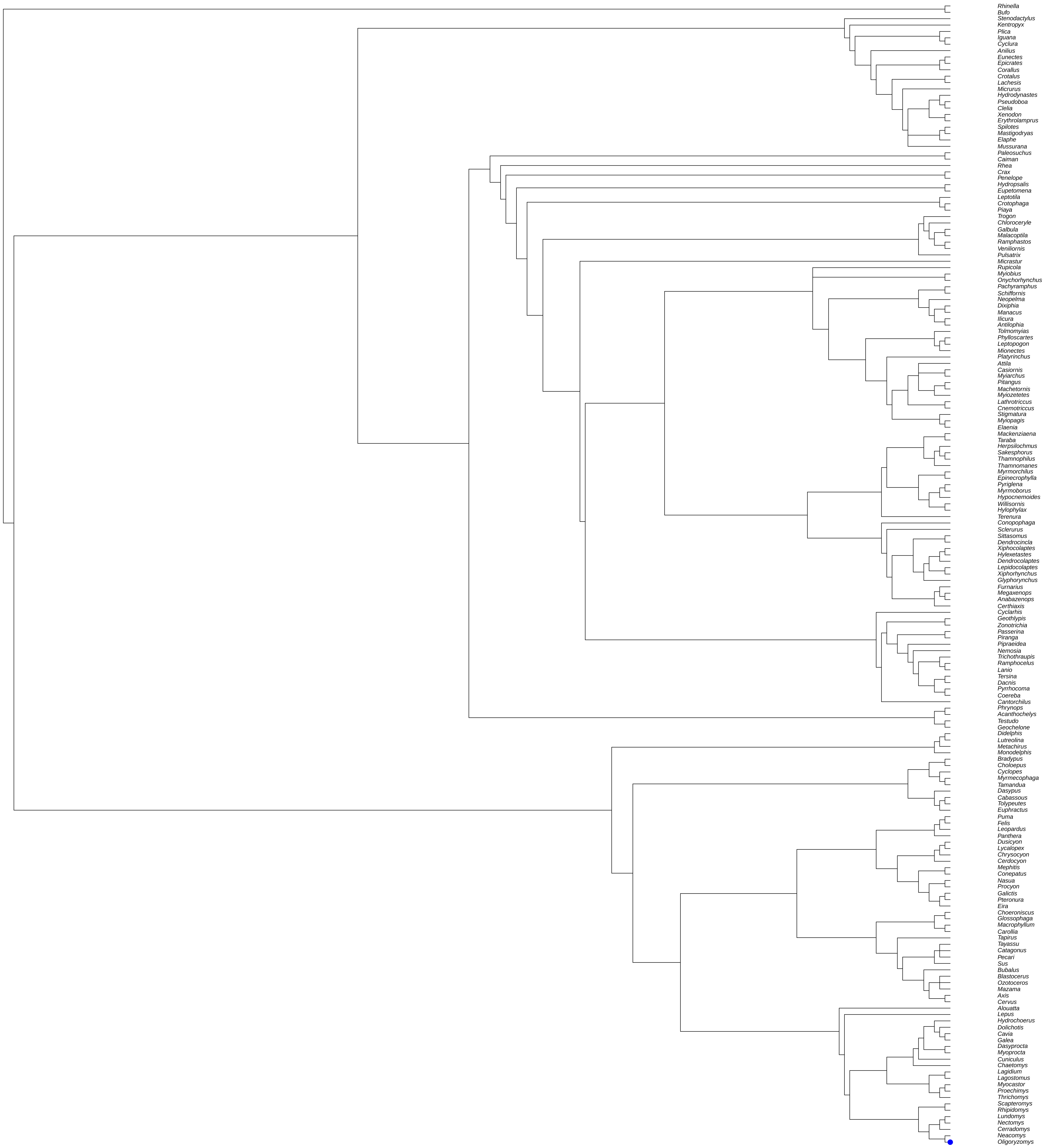

Ixodes sigelos larva

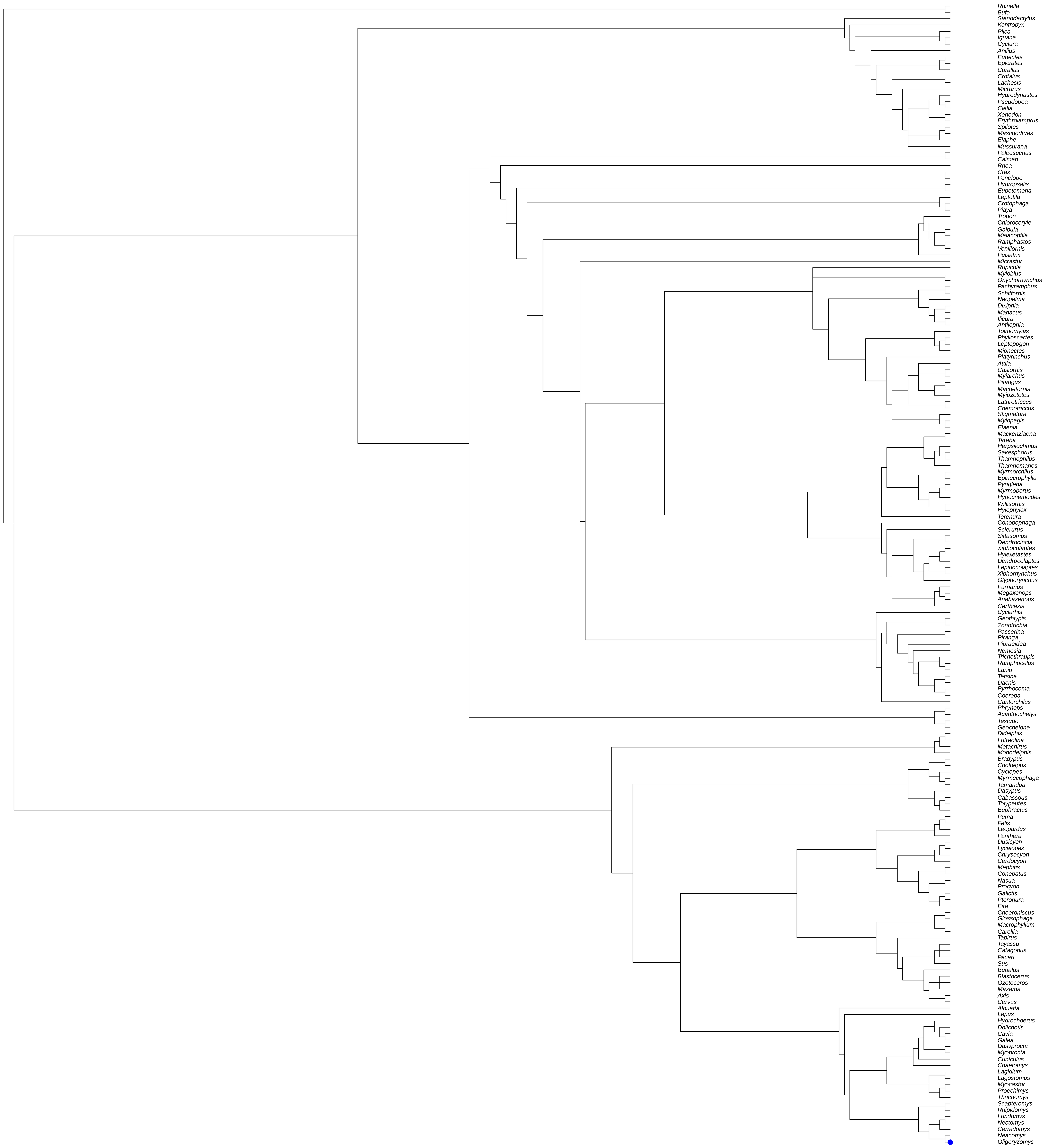

Ixodes sigelos nymph

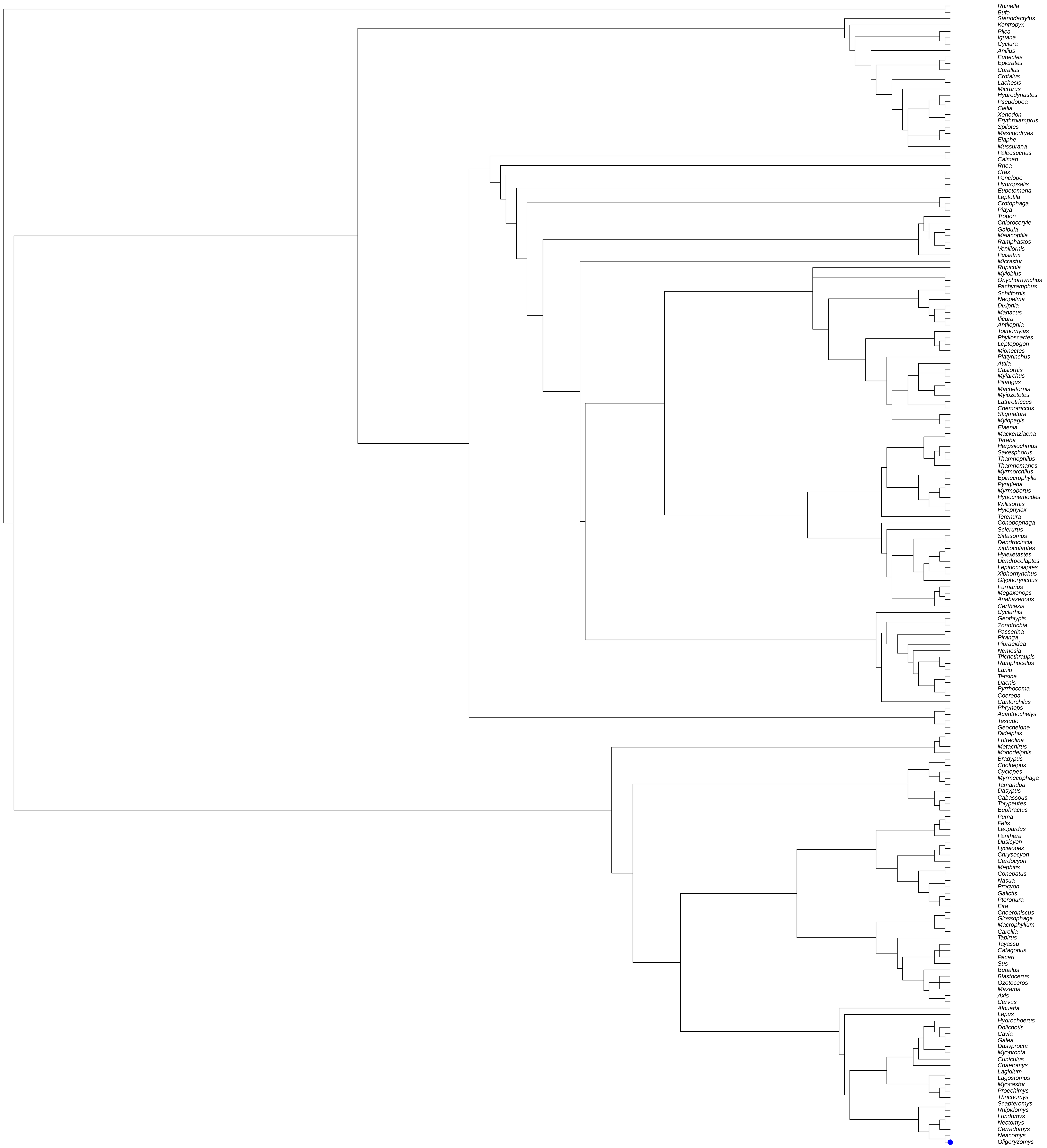

Ixodes stilesi nymph

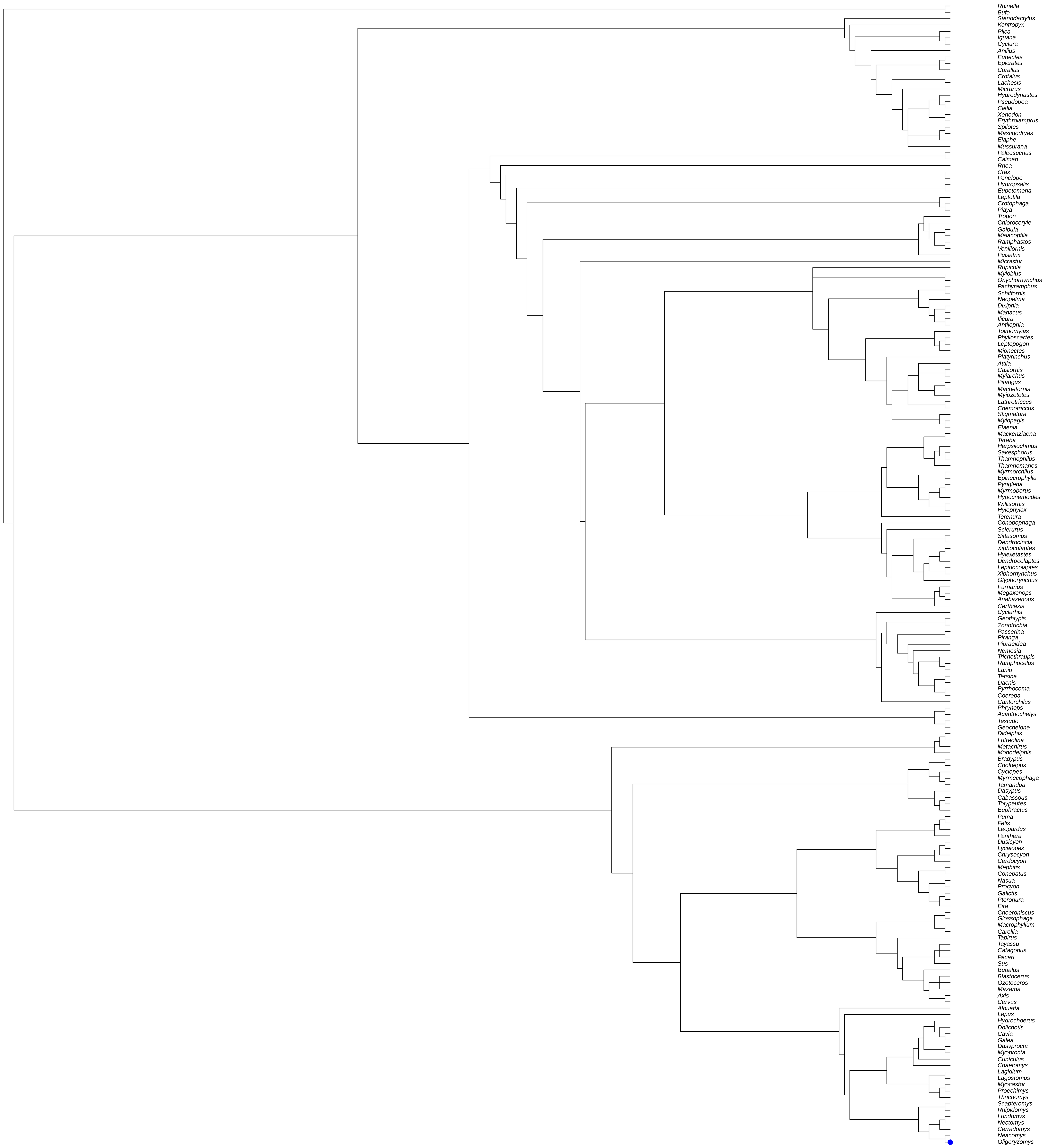

Ixodes tapirus adult

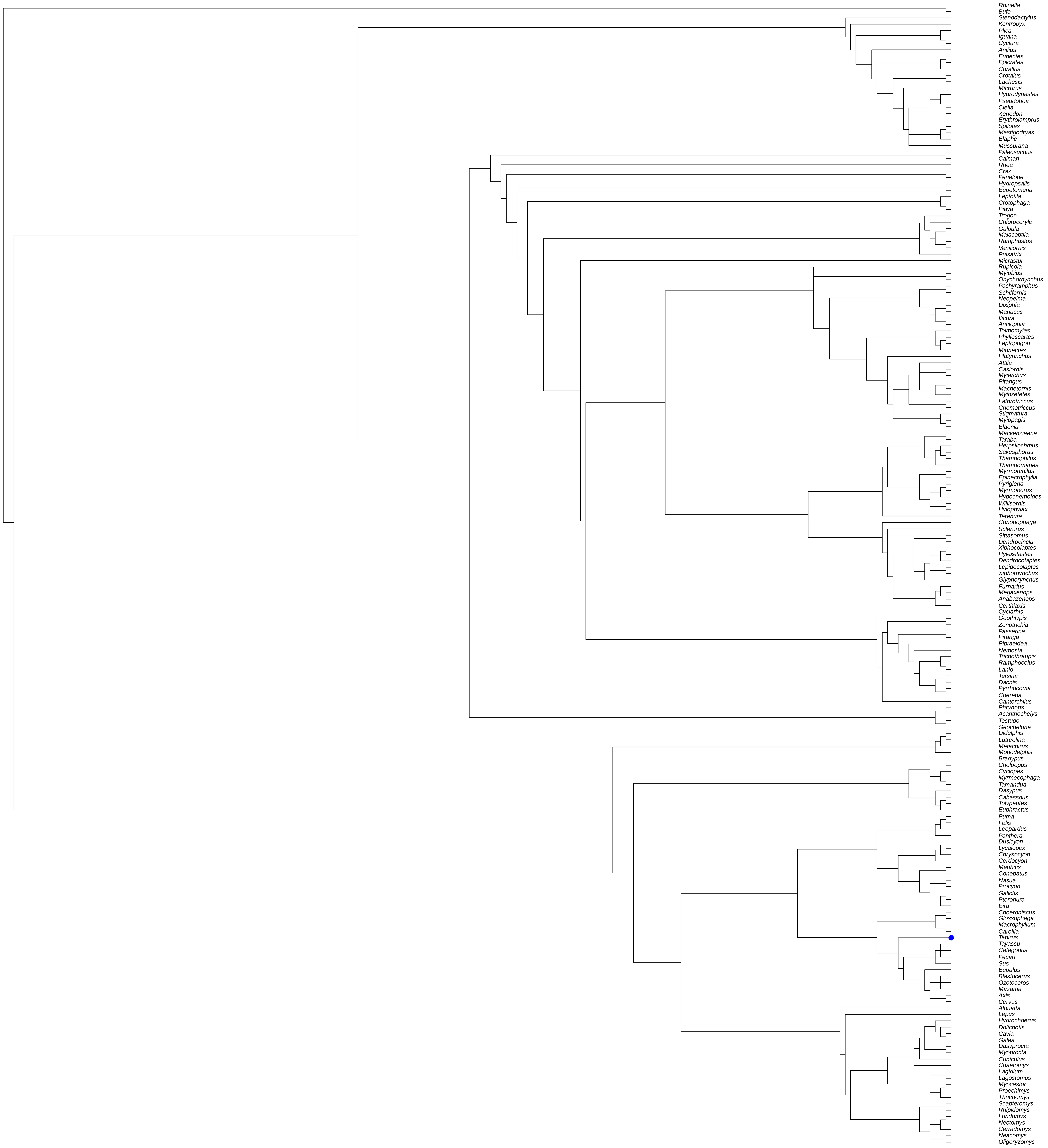

Ixodes venezuelensis adult

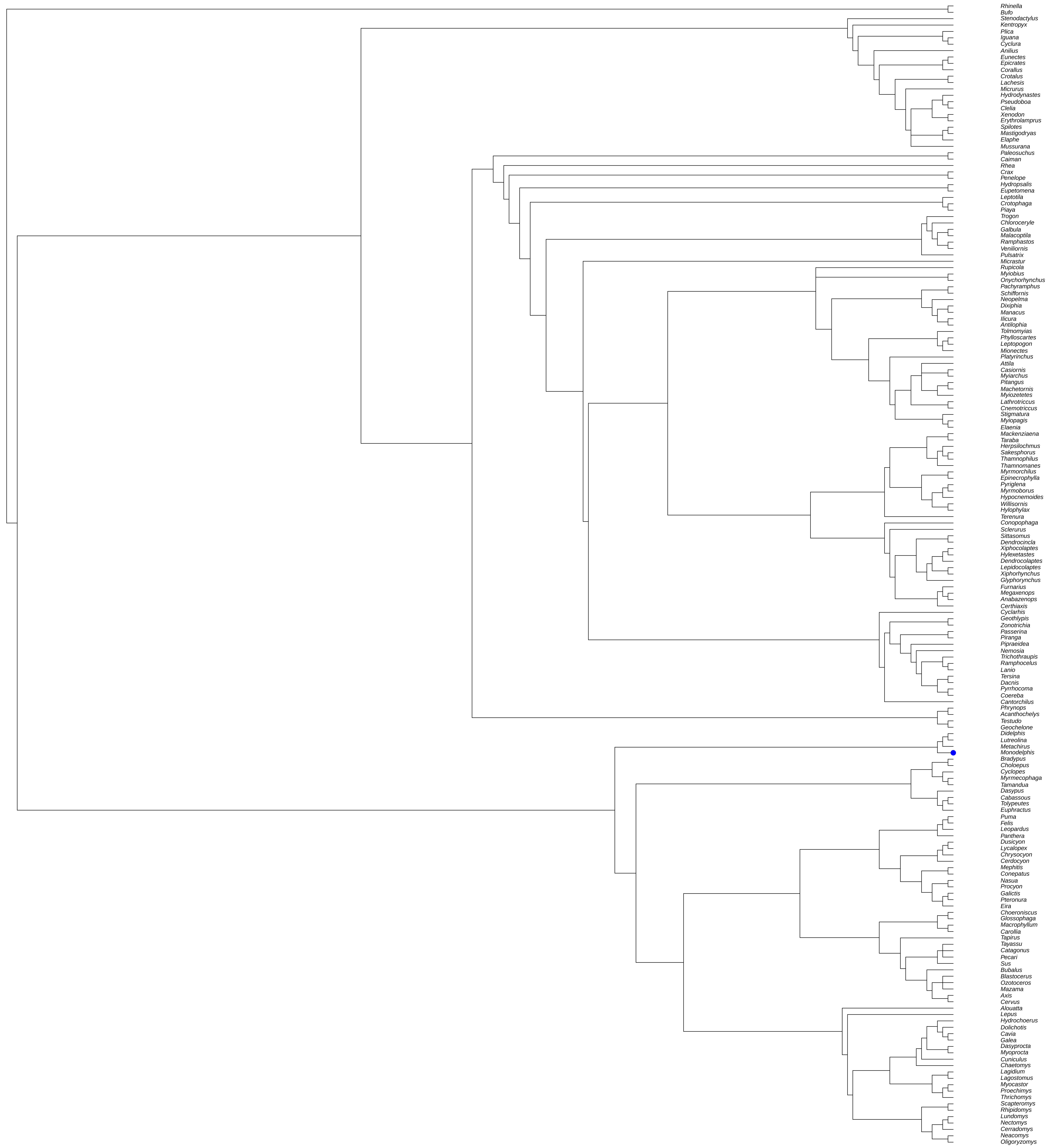

Ixodes venezuelensis larva

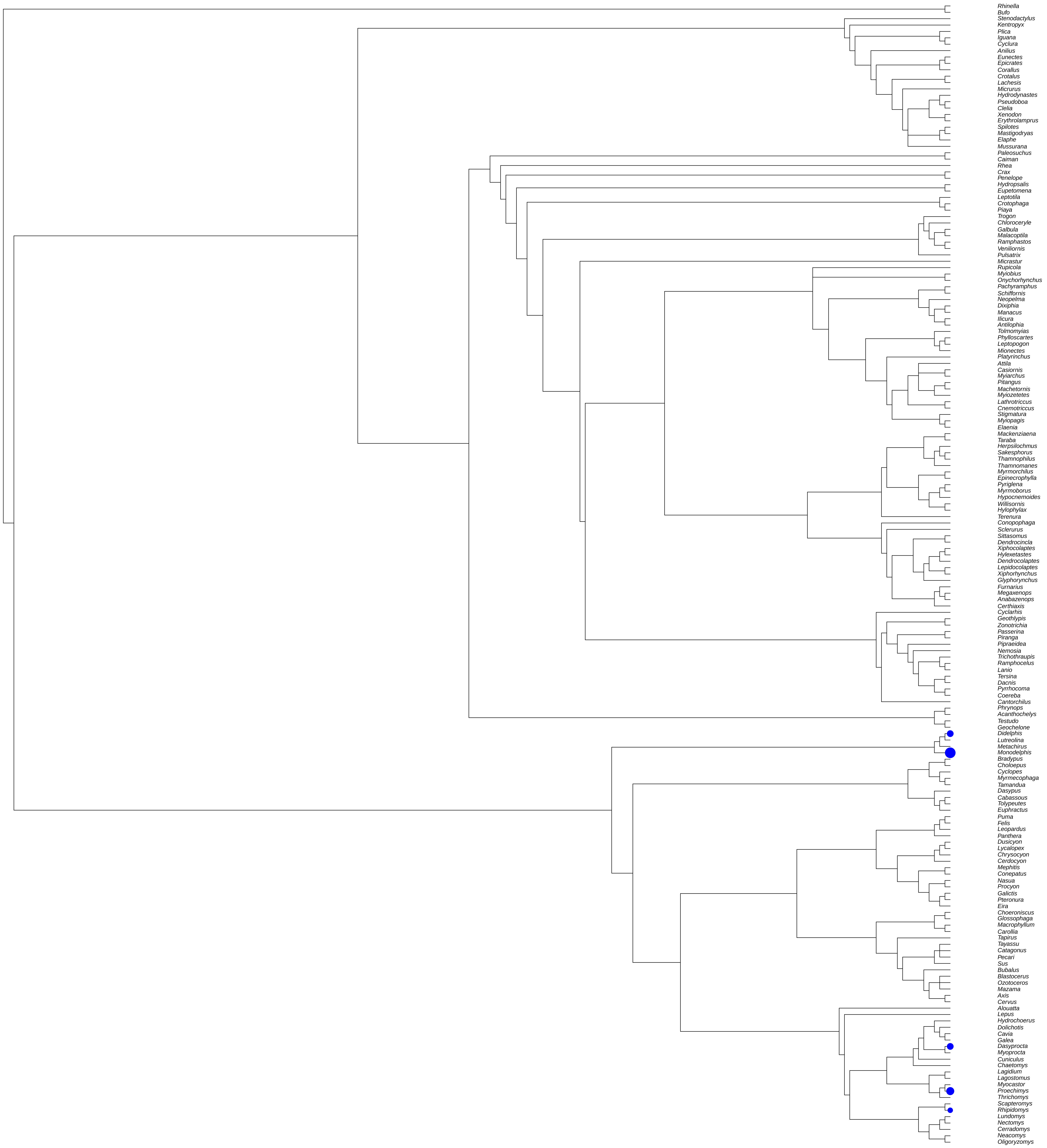

Ixodes venezuelensis adult

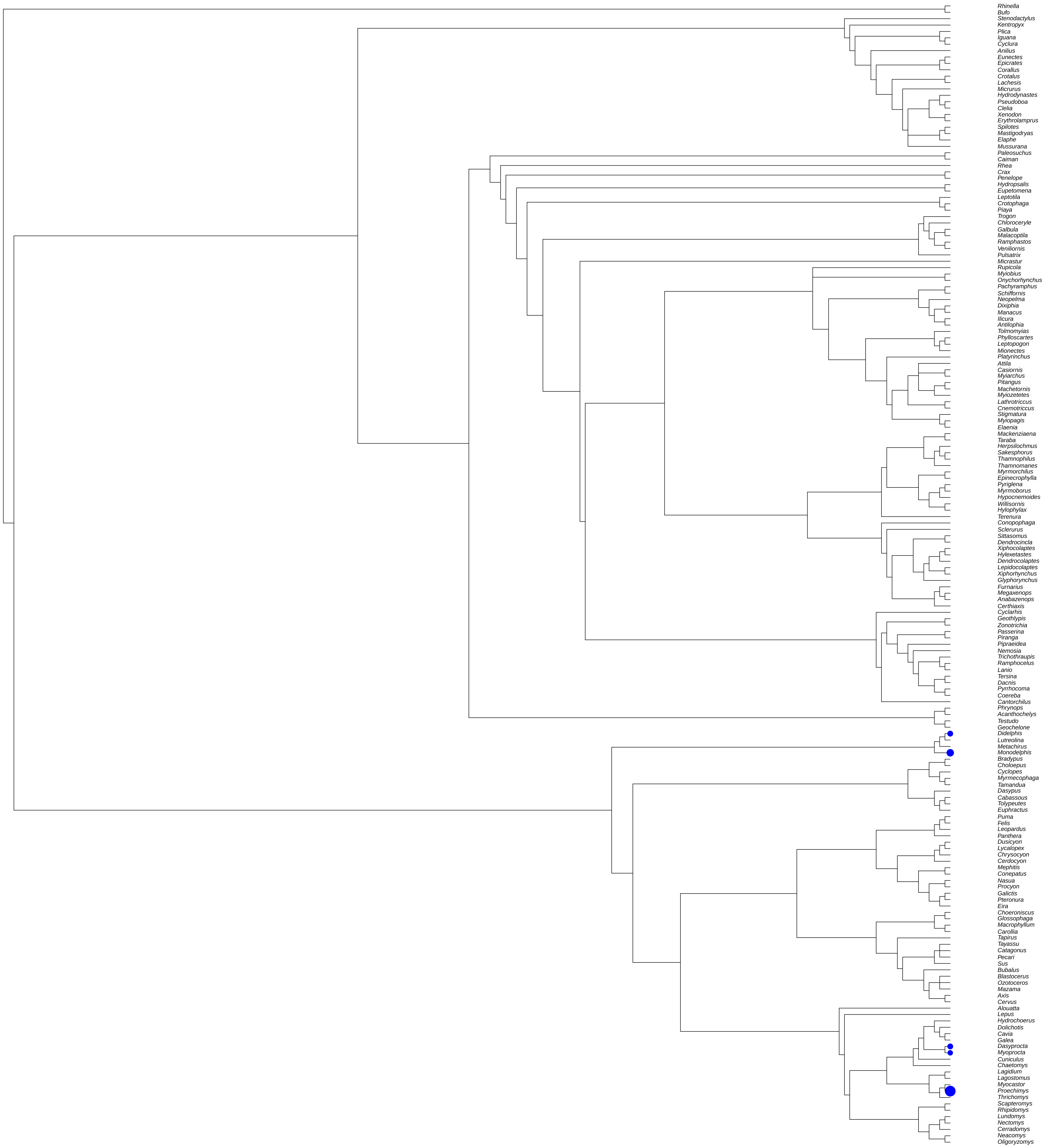

Supplement: Supplementary file 1 — Supplementary Information. [file Supplementary Figure S3.pdf › Supplementary Figure S3.pdf]
