# Supplementary material for: A community approach to the Neotropical ticks-hosts interactions
Source: Sci Rep. 2020 Jun 9;10:9269. doi: 10.1038/s41598-020-66400-3 (PMC7283479; doi:10.1038/s41598-020-66400-3)
Supplement: Supplementary file 1 — Supplementary Information. [file Supplementray Figure S1.pdf › Supplementray Figure S1.pdf]

Genera of hosts

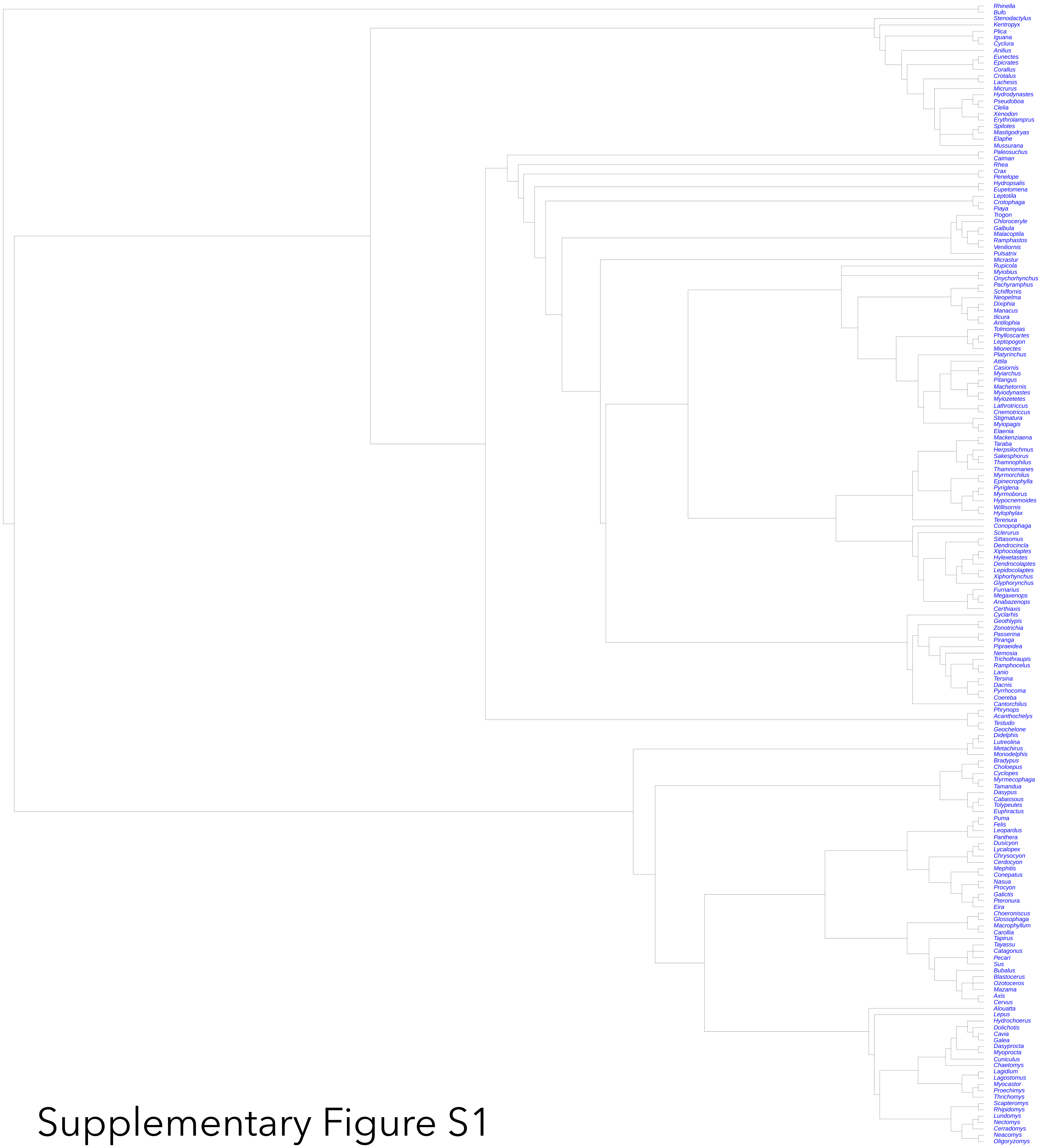

Supplementary Figure S1
